# Enantioselective Single and Dual α-C–H Bond Functionalization of Cyclic Amines via Enzymatic Carbene Transfer

**DOI:** 10.1021/jacs.2c10775

**Published:** 2022-12-21

**Authors:** Xinkun Ren, Bo M. Couture, Ningyu Liu, Manjinder S. Lall, Jeffrey T. Kohrt, Rudi Fasan

**Affiliations:** † Department of Chemistry, University of Rochester, Rochester, New York 14627, United States; ‡ Pfizer Inc., Medicine and Design, Groton, Connecticut 06340, United States

## Abstract

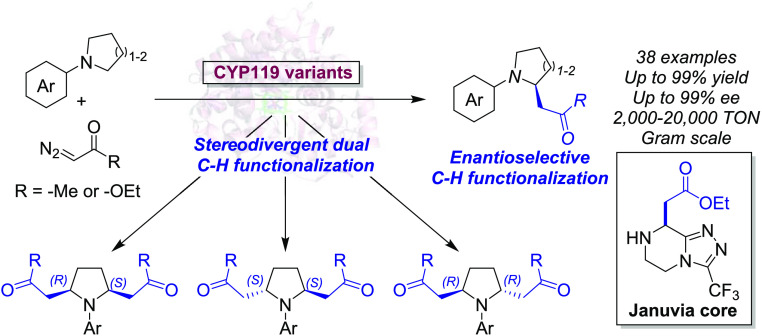

Cyclic amines are ubiquitous structural motifs found in pharmaceuticals and biologically active natural products, making methods for their elaboration via direct C–H functionalization of considerable synthetic value. Herein, we report the development of an iron-based biocatalytic strategy for enantioselective α-C–H functionalization of pyrrolidines and other saturated *N*-heterocycles via a carbene transfer reaction with diazoacetone. Currently unreported for organometallic catalysts, this transformation can be accomplished in high yields, high catalytic activity, and high stereoselectivity (up to 99:1 e.r. and 20,350 TON) using engineered variants of cytochrome P450 CYP119 from *Sulfolobus solfataricus*. This methodology was further extended to enable enantioselective α-C–H functionalization in the presence of ethyl diazoacetate as carbene donor (up to 96:4 e.r. and 18,270 TON), and the two strategies were combined to achieve a one-pot as well as a tandem dual C–H functionalization of a cyclic amine substrate with enzyme-controlled diastereo- and enantiodivergent selectivity. This biocatalytic approach is amenable to gram-scale synthesis and can be applied to drug scaffolds for late-stage C–H functionalization. This work provides an efficient and tunable method for direct asymmetric α-C–H functionalization of saturated *N*-heterocycles, which should offer new opportunities for the synthesis, discovery, and optimization of bioactive molecules.

## Introduction

**Figure 1:**
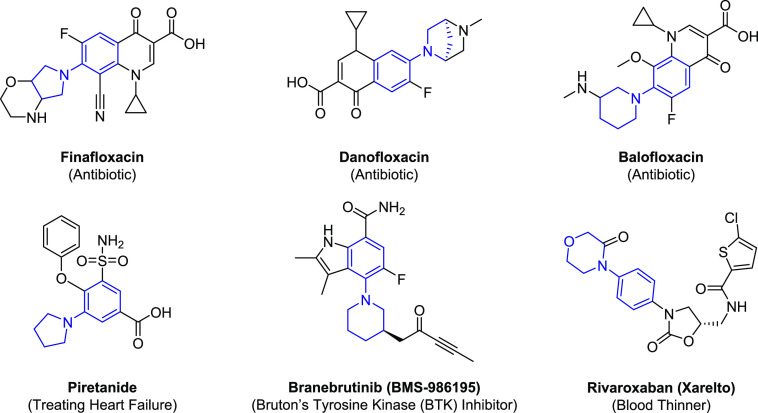
Representative drugs and bioactive natural products containing *N*-aryl pyrrolidines, piperidines, and other saturated *N*-heterocycles.

**Figure 2:**
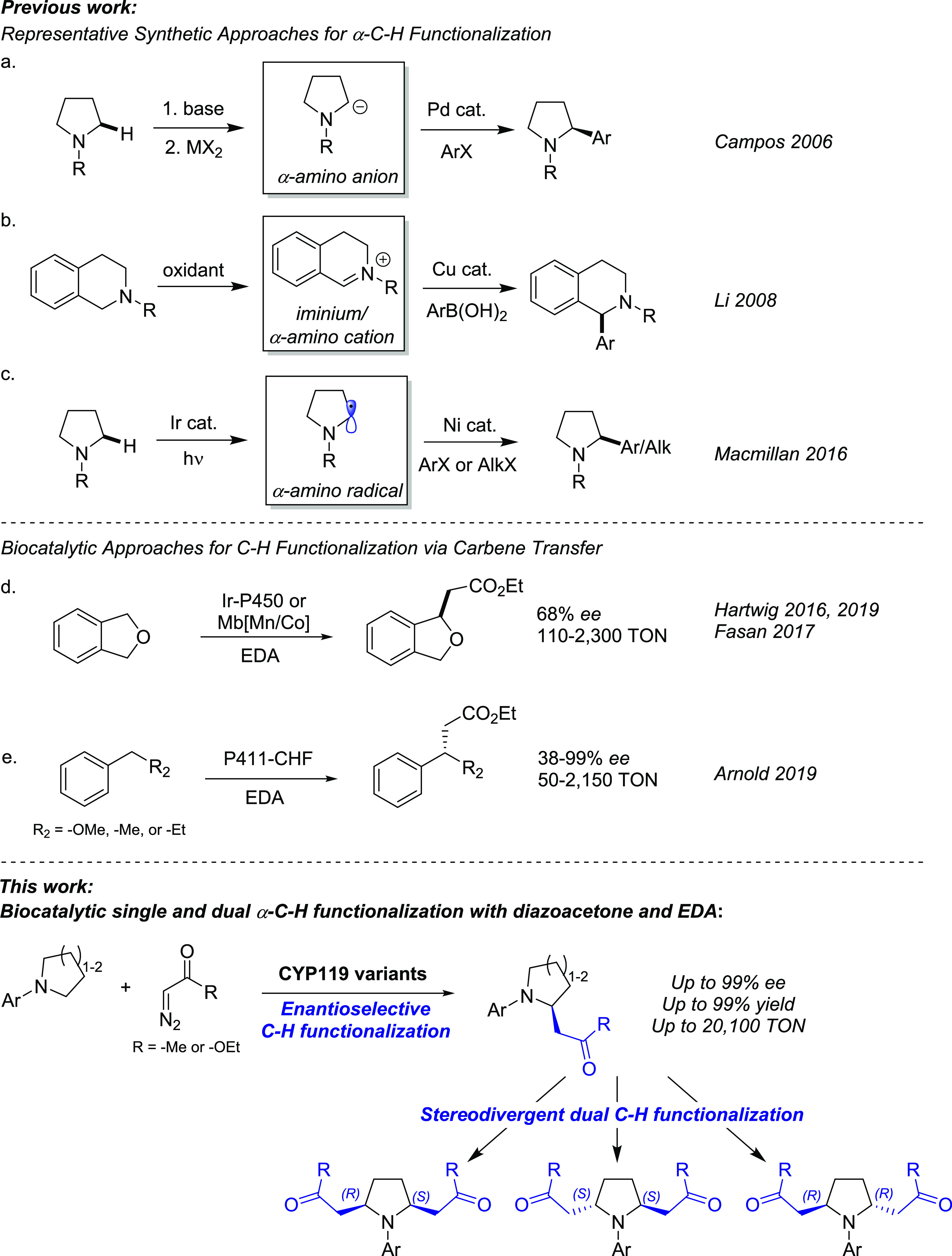
Representative chemocatalytic methods for α-C–H functionalization (a–c). Previous (d, e) and present biocatalytic strategy for C–H functionalization via enzyme-catalyzed carbene transfer.

Saturated *N*-containing heterocycles (e.g., pyrrolidine, piperidine, morpholine) are key components of many pharmaceuticals and biologically active natural products (Figure 1).^1^ Given their value as “privileged” scaffolds in medicinal chemistry, major efforts have been devoted to the development of strategies for the functionalization of these compounds. Among them, C(sp^3^)–H functionalization strategies resulting in the formation of new chiral carbon–carbon bonds are of particular interest.^2,3^ Current chemical strategies for α-C(sp^3^)–H functionalization of saturated *N*-heterocycles include multistep sequences in which strong bases are used to generate reactive α-amino anions, which can undergo transition metal-mediated alkylation/arylation (Figure 2a).^4−9^ Other notable approaches involve the use of oxidative α-C–H functionalization to generate an intermediate iminium ion, which is captured by carbon-based nucleophiles (Figure 2b),^10−14^ directing group mediated C(sp^3^)–H functionalization,^15−17^ and photoredox strategies (Figure 2c).^18−22^ Despite this progress, these protocols require multistep sequences, rare transition metals, and/or they lack stereoselectivity.

Enzymes are attractive alternatives for C–H functionalization because of their inherent chemoselectivity, sustainability, and the potential to be optimized via protein engineering for tuning activity and stereoselectivity.^23−28^ While natural enzymes capable of forging new carbon–carbon bonds via C(sp^3^)–H functionalization are rare and largely limited to specific substrates (e.g., methyl transfer reactions catalyzed by *S*-adenosylmethionine-dependent enzymes),^29−33^ recent advances in protein engineering have expanded the repertoire of enzyme-catalyzed C–H functionalization via carbene transfer chemistry (Figure 2d,e).^23,34^ Traditionally, C–H functionalization via metal–carbenoid insertion has been pursued through small molecule organometallic catalysts, including complexes with rhodium,^35−37^ iridium,^38,39^ and other metals.^40−43^ However, the realization of asymmetric intermolecular C(sp^3^)–H carbene insertions has represented a major challenge, with viable strategies being largely limited to rhodium-based systems and “donor–acceptor” carbene transfer reagents.^44^ Building upon key advances in the development of engineered hemoproteins as “carbene transferases” for olefin cyclopropanation and carbene heteroatom–hydrogen insertion,^25^ the reaction scope of these systems has been recently extended to the functionalization of C–H bonds.^23,34^ Using artificial metalloenzymes containing metallo-substituted hemoproteins, we and the Hartwig group reported the C(sp^3^)–H alkylation in phthalan substrates (Figure 2d)^45−47^ and C–H functionalization in indoles^48^ using ethyl diazoacetate (EDA) as carbene precursor.^45−47^ More recently, Arnold and co-workers reported an engineered biocatalyst derived from P450_BM3_ (“P411-CHF”) for the enantioselective insertion of EDA into benzylic and allylic C(sp^3^)–H bonds (Figure 2e).^49^ This biocatalyst was later optimized for the α-C–H alkylation of secondary anilines in the presence of a diazolactone reagent or trifluorodiazoethane.^50,51^ Despite this progress, the range of available strategies for biocatalytic C(sp^3^)–H functionalization via carbene transfer remains scarce. Here, we describe the development of a versatile biocatalytic platform for the efficient and enantioselective α-C(sp^3^)–H functionalization of cyclic amines using diazoacetone and ethyl diazoacetate as carbene donors (Figure 2). This approach offers a simple, scalable, and sustainable route to the preparation of enantioenriched α-functionalized cyclic amines amenable to further diversification using different chemistries. In addition, we show that these methodologies can be combined to afford difunctionalized products with diastereodivergent selectivity (Figure 2) and applied to late-stage functionalization of a drug precursor, which highlights their value for asymmetric synthesis and medicinal chemistry, respectively.

## Results and Discussion

### Development of CYP119 Catalysts for α-C–H Functionalization of *N*-Phenylpyrrolidine with Diazoacetone

**Figure 3:**
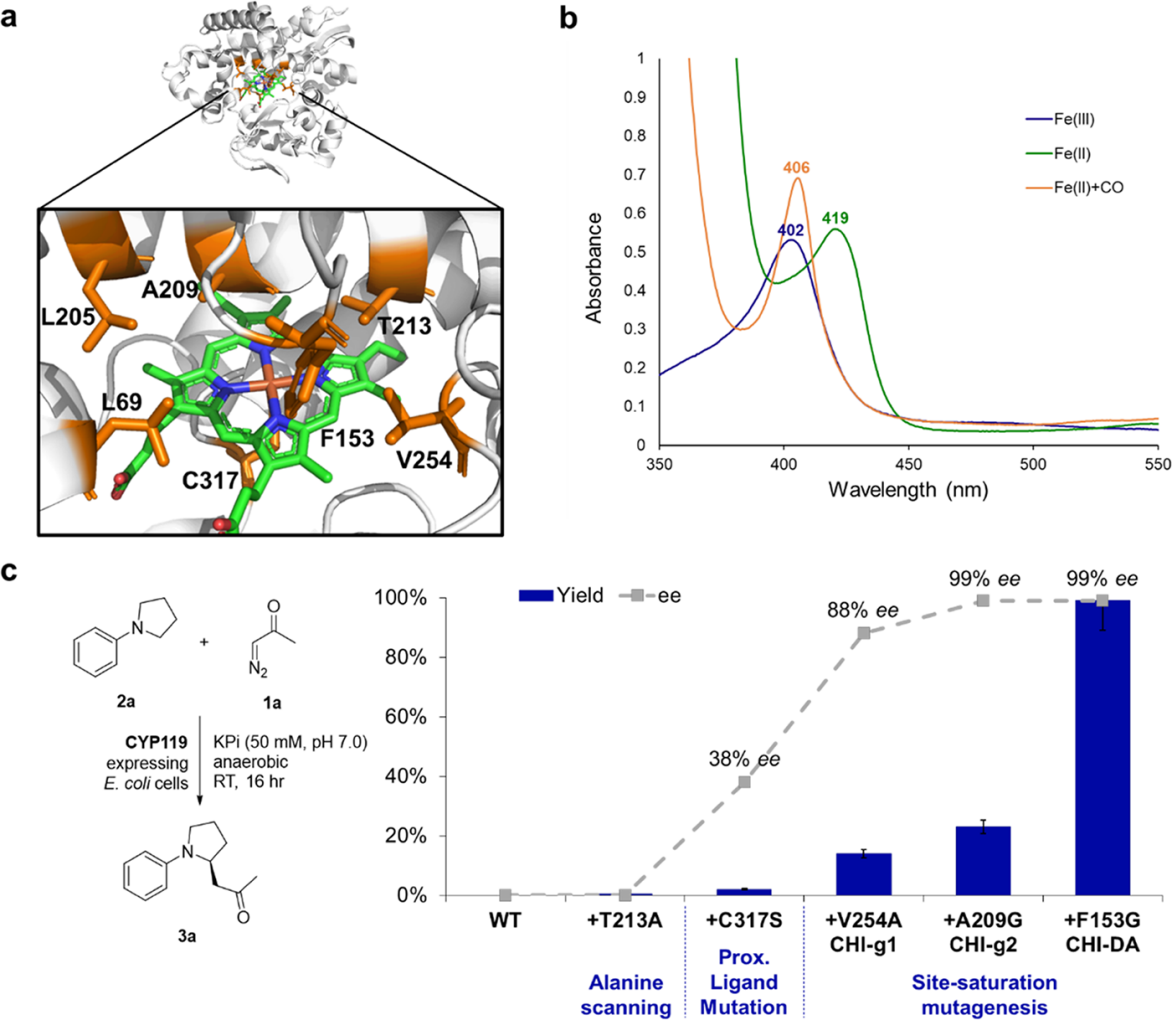
Structure, spectral properties, and directed evolution of CYP119 catalysts for enantioselective C–H functionalization of *N*-phenylpyrrolidine with diazoacetone. (a) X-ray crystal structure of CYP119 from *S. solfataricus* (PDB 1IO7 ^61^). Amino acid residues targeted for mutagenesis are highlighted in orange, and the heme cofactor is shown in green. (b) UV–vis absorption spectra for CYP119 (T213A, C317S) in its ferric state (Fe(III), blue), ferrous state (Fe(II), green), and CO-bound form (Fe(II)+CO, orange). (c) Directed evolution of CYP119 catalysts for enantioselective C–H functionalization of *N*-phenylpyrrolidine (2a) with diazoacetone (1a). Yields and % *ee* as determined under standard reaction conditions with diazoacetone (Table 1).

As an initial goal, we targeted the development of a biocatalyst that can promote the C–H functionalization of *N*-substituted pyrrolidine substrate **2a** in the presence of diazoacetone (**1a**) (Table 1), a reaction previously unreported for both chemical and biological catalysts. While diazoketones are versatile yet underexplored reagents for carbene transfer reaction, we envisioned this reaction would enable C–H functionalization of the cyclic amine with concomitant installation of a keto group, which is amenable to further diversification via known chemistries.^52^ In initial efforts, we screened an in-house library of engineered myoglobin variants that were previously shown to have high activity for a variety of carbene transfer reactions, including the C–H functionalization of indoles^48^ and cyclopropanation with benzyldiazoketones.^52^ However, none of these myoglobin variants show activity in the reaction of **1a** with **2a** either as purified proteins or in whole cells. These results prompted us to consider other hemoprotein scaffolds. Given the success with applying engineered P450_BM3_ variants for benzylic C–H functionalization with ethyl diazoacetate (EDA) but cognizant of their poor reactivity with diazoacetone,^49^ we directed our attention to other members of the cytochrome P450 enzyme family, namely, P450_cam_ (camphor hydroxylase),^53^ the thermophilic P450 CYP119 (Figure 3a) from *Sulfolobus solfataricus*,^54^ and the explosive-degrading P450 XplA,^55^ which was recently shown to possess non-native C–H amination reactivity via nitrene transfer.^56^ However, none of these enzymes show detectable activity in the target reaction either as purified proteins or in whole cells (Table 1; Supporting Information, SI Tables S1 and S2). The highly evolved P411-CHF,^49^ along with transition metal catalysts known to catalyze carbene C–H insertion reactions^57^ (SI Table S1), also failed to produce the desired C–H insertion product, further highlighting the challenges associated with this transformation.

**Table 1:**
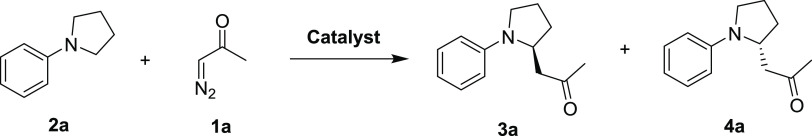
Intermolecular C–H Functionalization of *N*-Phenylpyrrolidine (**2a**) with Diazoacetone Using Hemoproteins and Variants Thereof^a^

entry	catalyst	yield (%)^b^	TON^c^	e.r.^d^ (**3a**:**4a**)
1	Hemin	0	0	-
2	Mb (WT)	0	0	-
3	Mb (H64G, V68A)	0	0	-
4	P411-CHF	0	0	-
5	CYP119 (WT)	0	0	-
6	CYP119 (T213A)	0.4	35	nd
7	CYP119 (T213A, C317S)	2	170	69:31
8	CYP119 (T213A, V254A, C317S) **CHI-g1**	14	200	94:6
9	CYP119 (A209G, T213A, V254A, C317S) **CHI-g2**	23	3,100	99.5:0.5
10	CYP119 (F153G, A209G, T213A, V254A, C317S) **CHI-DA**	99	12,900	99.5:0.5
11^e^	CYP119 (F153G, A209G, T213A, V254A, C317S) **CHI-DA**	99	500	99.5:0.5
12^f^	CYP119 (F153G, A209G, T213A, V254A, C317S) **CHI-DA**	53	20,350	99.5:0.5

a Standard reaction conditions: protein expressing C41(DE3) *Escherichia coli* cells, OD_600_ = 40, 10 mM **2a**, 20 mM diazoacetone (**1a**), in KPi buffer (50 mM, pH 7), room temperature, 16 h, in an anaerobic chamber.

b Assay yields as determined by gas chromatography (GC) using calibration curves with the isolated product.

c TON as calculated based on the protein concentration measured from cell lysate.

d Enantiomeric ratio (e.r.) for **3a**:**4a** as determined by chiral supercritical fluid chromatography (SFC).

e Using 20 μM purified protein and 10 mM Na_2_S_2_O_4_.

f OD_600_ = 10. nd = not determined.

Given the lack of activity of wild-type P450 XplA, P450_cam_, and CYP119, we generated a set of active site “alanine scanning” libraries in which amino acid residues lying in close proximity to heme cofactor are substituted for alanine (5 sites for XplA, 11 sites for P450_cam_, and 6 sites for CYP119; SI Table S2), with the goal of systematically varying the active site shape of these enzymes and identify mutations that could favor interaction with the non-native substrate. The corresponding variants were expressed in *E. coli* C41(DE3) and tested for their activity in the reaction with **2a** and diazoacetone (**1a**) as whole cells. While the large majority of these enzyme variants showed no activity in the reaction (SI Table S2), CYP119 variant T213A was found to exhibit basal activity (0.4% yield) toward the formation of the C–H functionalization product **3a**/**4a** (Table 1, entry 6). Using CYP119 (T213A) as the starting point, we next evaluated the effects of mutating the heme axial ligand as a means to tune the reactivity of the enzyme, since this approach has proven valuable in the context of other hemoprotein-catalyzed carbene transfer reactions.^46,58−60^ Accordingly, the heme coordinating Cys317 residue in CYP119 (T213A) was mutated to each of the other proteinogenic nucleophilic amino acid residues such as His, Ser, Thr, Tyr, Arg, Lys, Asp, and Glu. Among these variants, CYP119 (T213A, C317S) showed slightly improved activity compared to the parent enzyme, producing **3a**/**4a** in 2% yield and 69:31 enantiomeric ratio (Table 1, entry 7). The CO-bound ferrous form of this serine-ligated CYP119 variant displays a Soret peak at 406 nm (Figure 3b). A similar blue shift in the Soret band has been observed upon an axial Cys→Ser substitution in P450_BM3_,^58^^58^ although the λ_max_ of the ferrous CO-bound form of these enzymes clearly depends on the type of P450 under investigation (e.g., 411 nm for P450_BM3_,^58^ 416 nm for P450_cam_ (C353S) variants in Table S2).

To improve its reactivity toward C–H functionalization of **2a**, CYP119 (T213A, C317S) was then subjected to iterative rounds of active site mutagenesis and screening (Figure 3c). Specifically, active site residues L69, F153, L205, A209, and V254 were individually randomized using a combination of partial amino acid alphabets (KBG, WDC, MHG degenerate codons in 1:1:1 ratio) comprising (mostly) uncharged residues of variable size (Gly, Ala, Ser, Val, Leu, Trp, Thr, Asn, Gln, Cys, Pro, Ile, Met, Phe, Thr). The libraries were expressed in *E. coli* C41 (DE3) and screened as whole cells in 96-well plates. Using this strategy, a dramatic improvement in both activity and stereoselectivity of the enzyme for the synthesis of **3a** could be achieved after three rounds of directed evolution, as summarized in Figure 3c. In particular, accumulation of a beneficial mutation at position 254 (V254A) led to a tripled mutant variant (called “CHI-g1”) that exhibits improved yield (2 → 14%) and enantioselectivity (94:6 vs 69:31 e.r.) compared to CYP119 (T213A, C317S) (Table 1, entry 8). Using CHI-g1 as the parent, introduction of another space-creating mutation at the level of active site residue 209 (Ala209 → Gly) gave a variant (called “CHI-g2”) featuring excellent enantioselectivity (99.5:0.5 er) as well as dramatically (>15-fold) improved TON for the conversion of **2a** into **3a** (200 → 3100 TON; Table 1, entry 9). Despite the high TON, the product yield obtained in whole-cell reactions using this variant was still moderate (23%). CHI-g2 was thus subjected to another round of KBG/WDC/MHG-based mutagenesis at the yet unaltered active site positions (i.e., L69, F153, L205), leading to identification of an improved variant carrying a F153G mutation called “CHI-DA” (Table 1, entry 10). Using whole-cell reactions at a cell density (OD_600_) of 40, the CYP119 variant CHI-DA delivers **3a** in quantitative yield as well as with excellent enantioselectivity (>99% *ee*), supporting 12,900 turnovers (Table 1, entry 10). The same yield and enantioselectivity were also obtained for reactions with purified protein at 0.2 mol % (Table 1, entry 11). The (*S*)-absolute configuration of **3a** was assigned based on crystallographic analysis of the related product **3e** (SI Figure S4).

From time course experiments, the CHI-DA-catalyzed conversion of **2a** into **3a** was determined to proceed with an initial turnover frequency (TOF) of 150 turnovers/min and to reach completion within 12 h (SI Figure S1). Furthermore, under catalyst-limited conditions (OD_600_ = 10), CHI-DA was determined to catalyze the C–H functionalization of **2a** with over 20,000 turnovers (Table 1, entry 12). Thus, in addition to offering excellent enantioselectivity, the catalytic activity (TON) of this evolved CYP119-based carbene transferase is one to two orders of magnitude higher than those previously achieved with engineered P411s on other C–H carbene insertion reactions (Figure 2e).^49−51^

### Substrate Scope of CHI-DA with Pyrrolidine Derivatives

**Figure 4:**
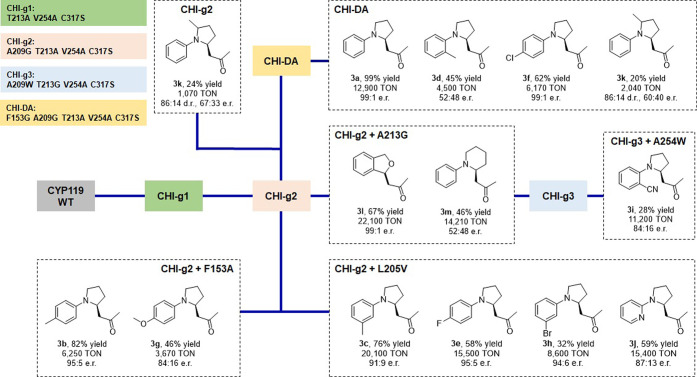
Activity and selectivity of CYP119-based biocatalysts for α-C–H functionalization of *N*-aryl-pyrrolidines with diazoacetone. Yields, TON, and enantioselectivity as determined from whole-cell reactions under standard reaction conditions with diazoacetone, as described in Table 1. The lines indicate the relationship among the enzyme variants along the evolutionary lineage.

To explore the substrate scope of CHI-DA, this enzyme was challenged with a panel of variously substituted *N*-aryl pyrrolidine substrates (**2b**–**l**) in the presence of diazoacetone (**1a**) (SI Table S3). Among these compounds, *para*-substituted *N*-phenyl-pyrrolidine derivatives such as **2b** (*p*-Me), **2e** (*p*-F), and **2f** (*p*-Cl) were efficiently converted (5,150–9,340 TON) into the desired products **3b**, **3e**, and **3f** in good to high yields (51–93%) and enantioselectivity (86:14 to 91:9 e.r.) (SI Table S3). The *para*-methoxy derivative **3g** was also afforded in good yield and high TON (5,240), albeit with more moderate enantioselectivity (75:25 e.r.). In contrast, lower levels of activity and/or enantioselectivity were observed in the presence of the *ortho*- and *meta*-substituted *N*-phenyl-pyrrolidine derivatives (SI Table S3), indicating a lower tolerance of the enzyme to substitutions other than at the para position. To overcome this limitation, the most active members from the enzyme libraries derived from CHI-g1, CHI-g2, and CHI-g3 were screened against these substrates, resulting in the identification of catalysts with improved activity and enantioselectivity for the synthesis of **3c** (*m*-Me), **3h** (*m*-Br), **3i** (*o*-CN), as well as the pyridyl derivative **3j** (Figure 4). Using CHI-g2(L205V), the latter product (**3j**) could be obtained in a 3-fold higher yield (59 vs 20%), 10-fold higher TON, and higher enantioselectivity (87:13 vs 60:40 er) compared to CHI-DA. In general, CHI-g2 (L205V) shows significantly improved activity and enantioselectivity across multiple substrates not well accepted by CHI-DA (e.g., **3h**: 32 vs 5% yield and 94:6 vs 85:15 e.r.; Figure 4 and Table S3), thus offering a useful complementarity to the latter enzyme in terms of substrate scope. A racemic substrate bearing α methyl group to the pyrrolidine nitrogen (**2k**) was also prepared and tested. Notably, the corresponding C–H insertion product **3k** was obtained in 86:14 diastereomeric ratio using either CHI-DA or CHI-g2 (Figure 4), suggesting a potential utility of these enzymes also for kinetic resolution of racemic substrates upon further optimization of the biocatalyst for this application. In contrast, no enantioselectivity could be achieved with **3d** (Figure 4). In addition to the pyrrolidine-based substrates, efficient and highly enantioselective α-C–H functionalization could be achieved in the presence of other heterocyclic substrates, such as piperidine, to give **3m** in 46% yield (14,210 TON; 52:48 e.r.), and phthalan, whose transformation delivered **3l** in 67% yield (22,210 TON) and excellent enantioselectivity (99:1 e.r.) using CHI-g2 (A213G) as the catalyst (Figure 4). Collectively, these CYP119-based biocatalysts are capable of processing a broad range of *N*-aryl pyrrolidines and other saturated heterocycles with high TON and, in most cases, high stereoselectivity (Figure 4).

### C–H Functionalization of *N*-Phenyl-pyrrolidine with EDA

Given the high activity and selectivity of the engineered CYP119 catalysts in the α-C–H functionalization with diazoacetone, we extended our investigations to the C–H functionalization of *N*-phenyl-pyrrolidine in the presence of ethyl diazoacetate (EDA; **1b**). This reaction was previously investigated using P411-CHF, but its scope was limited to a few substrates (3).^49^ On the other hand, other hemoproteins and heme enzymes showed no activity (SI Table S4). Following a similar strategy as described above for the diazoacetone reaction, the CYP119-based active site alanine scanning library was initially screened in whole cells by targeting C–H functionalization of **2a** with EDA as a model reaction. Unlike the diazoacetone counterpart, wild-type CYP119 and most of the alanine mutants showed detectable activity in this reaction (3–6% yields; 130–250 TON; SI Table S5) with the L69A, T213A, or V254A single mutation, each providing a 2-fold improvement in yield compared to the wild-type enzyme. A combination of the most beneficial alanine mutation (T213A) with the axial ligand mutation C317S led to a 3-fold improvement in yield (6 → 18%) and a 6-fold improvement in TON (135 → 812), furnishing **6a** with moderate enantioselectivity (71:29 e.r.; Table 2, entry 4). Using CYP119 (T213A, V254A) as background, systematic mutagenesis of the heme coordinating cysteine residue (Cys317) established that while all of these axial ligand variants remain functional, the Cys→Ser mutation is again most beneficial for supporting this carbene transfer reaction (**SI**Figure S3). Notably, the corresponding triple mutant variant CYP119 (T213A, V254A, C317S) (called CHI-g1) was found to catalyze the formation of **6a** in quantitative yield (99%) and slightly improved enantioselectivity (79:21 e.r.) compared to CYP119 (T213A, C317S) (Table 2, entry 5). To further enhance the enantioselectivity for this reaction, the CHI-g1-derived libraries generated via KBG/WDC/MHG-mutagenesis of F153, L205, A209, and G210 were screened in the presence of **2a** and EDA. This process led to the identification of an improved catalyst for this reaction, CYP119 (F153G, T213A, V254A, C317S) (called “CHI-EDA”), which is capable of delivering the desired C–H insertion product **6a** in 89% yield, 87:13 e.r. with 8,920 TON in whole-cell reactions (Table 2, entry 6). Similar results (99% yield; 87:13 e.r.) were obtained using this enzyme in purified form (Table 2, entry 7). From a preparative-scale reaction with CHI-EDA expressing cells, 182 mg of **6a** was readily obtained in 82% isolated yield, demonstrating the scalability of this reaction. Notably, this biocatalytic system was found to furnish **6a** in good yield (51%) with a TON of 5,090 in the presence of air after 2 h of reaction time (Table 2, entry 9). This activity corresponds to a mere 2-fold decrease in activity compared to that measured under anaerobic conditions (Table 2, entry 9 vs 8), further highlighting the efficiency of this biocatalyst. To our knowledge, this is the first example of a biocatalytic C–H carbene insertion reaction achieved under aerobic conditions.

**Table 2:**
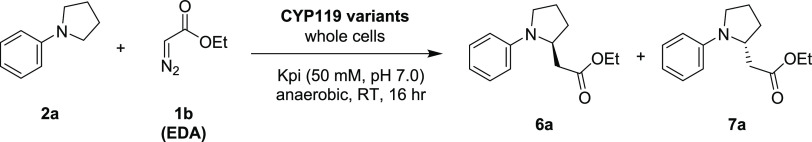
Activity and Selectivity of Representative Engineered CYP119 Variants for the Intermolecular C–H Functionalization of *N*-Phenylpyrrolidine with EDA^a^

entry	enzyme variant	yield (%)^b^	TON^c^	e.r. (**6a**:**7a**)
1	CYP119 (WT)	3	76	nd
2	CYP 119 (C317S)	5	110	nd
3	CYP119 (T213A)	6	135	nd
4	CYP119 (T213A, C317S)	18	812	71:29
5	CYP119 (T213A, V254A, C317S)(**CHI-g1**)	99	4,400	79:21
6	CYP119 (F153G, T213A, V254A, C317S) (**CHI-EDA**)	89 (64)^d^	8,920	87:13
7^e^	CYP119 (F153G, T213A, V254A, C317S) (**CHI-EDA**)	99	500	87:13
8^f^	CYP119 (F153G, T213A, V254A, C317S) (**CHI-EDA**)	87	8,710	87:13
9^f^,^g^	CYP119 (F153G, T213A, V254A, C317S) (**CHI-EDA**)	51	5,090	87:13
10	CYP119 (A209W, T213G, V254A, C317S)	71	6,750	19:81

a Standard reaction conditions: CYP119-expressing C41 (DE3) *E. coli* cells, OD_600_ = 40, 10 mM **1a**, 20 mM EDA, in KPi buffer (50 mM, pH 7), room temperature, 16 h, in an anaerobic chamber.

b Assay yields as determined by GC using calibration curves generated with the isolated product.

c TON as calculated based on the protein concentration measured from cell lysate.

d Isolated yield.

e Using 20 μM purified protein and 10 mM Na_2_S_2_O_4_.

f Reaction time: 2 h.

g Under aerobic conditions. nd = not determined.

While CHI-EDA catalyzes the C–H alkylation of **2a** with (*S*)-enantioselectivity, enantiodivergent selectivity in this transformation could be achieved using CYP119 (A209W, T213G, V254A, C317S), which produces the (*R*)-configured product **7a** in 71% yield and 81:19 e.r. (Table 2, entry 10). In addition, kinetic experiments showed that the CHI-EDA-catalyzed reaction with **2a** and EDA is remarkably fast, proceeding with an initial turnover frequency (TOF) of 2,900 turnovers per minute and reaching completion within only 2 h (SI Figure S2). This reaction is about 20-fold faster than the CHI-DA-catalyzed C-H functionalization of **2a** with diazoacetone, which proceeds with a TOF of 150 turnovers min^–1^ (SI Figure S1).

### C–H Functionalization of Cyclic Amines with EDA

**Figure 5:**
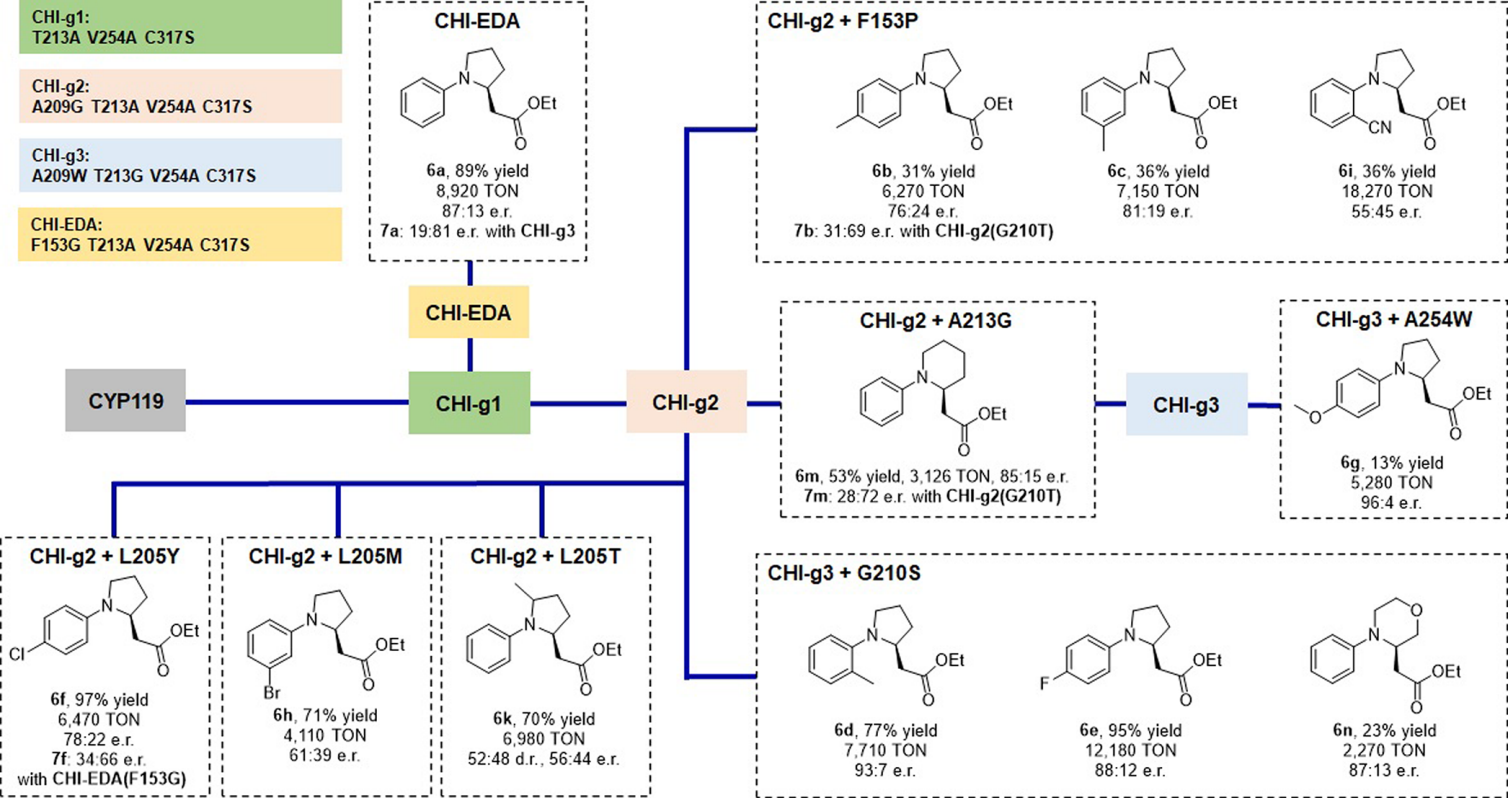
Activity and selectivity of CYP119 biocatalysts for α-C–H functionalization of cyclic amines with EDA. Yields, TON, and enantioselectivity were determined from whole-cell reactions under standard reaction conditions with EDA, as described in Table 2. The lines indicate the relationship among the enzyme variants along the evolutionary lineage.

Upon challenging CHI-EDA with other *N*-aryl-pyrrolidine derivatives (**2b**–**n**), lower levels of activity were generally observed compared to **6a** (e.g., 27–34% yield for **6e** and **6f**), indicating a more pronounced substrate specificity for this biocatalyst and reaction as opposed to broader substrate scope of the CYP119 biocatalysts for the reactions with diazoacetone (e.g., CHI-DA; SI Table S3). This behavior is reminiscent of that of engineered cytochrome P450s in native (monooxygenation) reactions or non-native reactions.^62^ This finding prompted us to pursue a substrate vs library approach for identifying better catalysts for these target substrates. Accordingly, the latter were screened in parallel against the most active CYP119 variants identified during the evolution of CHI-DA (Figure 3c). Among them, the CHI-g2-derived variants proved most effective for the enantioselective C–H functionalization of substituted *N*-aryl-pyrrolidines, giving **6b**, **6c**, **6f**, **6h**–**k**, and **6m** in up to 97% yield and 81:19 enantiomeric ratio (Figure 5). On the other hand, products **6d**, **6e**, **6g**, and **6n** were most efficiently afforded using CHI-g3 derived variants, with up to 95% yield and 96:4 e.r. (Figure 5). Notably, using their respective optimal catalysts, **6e** (p-F) and **6i** (o-CN) were obtained in nearly quantitative yields with over 12,000–18,000 TON, which corresponds to 5–10-fold higher TON values than that previously reported for P411-CHF in a related reaction.^49^ Furthermore, along with the previously mentioned enantiodivergent synthesis of **7a** using CHI-g3 (Table 2), catalysts useful for the synthesis of **7b**, **7f**, and **7m** with inverted enantioselectivity were also discovered (e.g., 31:69 e.r. for **7b** (*p*-Me) with CHI-g2(G210T) vs 76:24 e.r. with CHI-g2 (G210T); Figure 5).

Compared to the diazoacetone-driven reactions, the CYP119-catalyzed reactions in the presence of EDA were determined to proceed with lower activity but significantly higher enantioselectivity with *N*-phenyl-piperidine as the substrate (**6m**, 53% yield, 85:15 e.r.), and they could be further extended to the α-C–H functionalization of a morpholine substrate (**6n**, 23% yields, 87:13 e.r.; Figure 5), thus demonstrating the scope of this methodology across other important types of cyclic amine scaffolds. Furthermore, both stereoisomers of the piperidine product **6m** and **7m** were obtained in enantioenriched form (85:15 and 28:72 e.r.) using stereodivergent variants (Figure 5). Notably, these reactions can be readily scaled up to obtain the desired C–H insertion products on a semi-preparative scale (60–100 mg).

### Stereodivergent Dual C–H Functionalization with Diazoacetone and EDA

**Figure 6:**
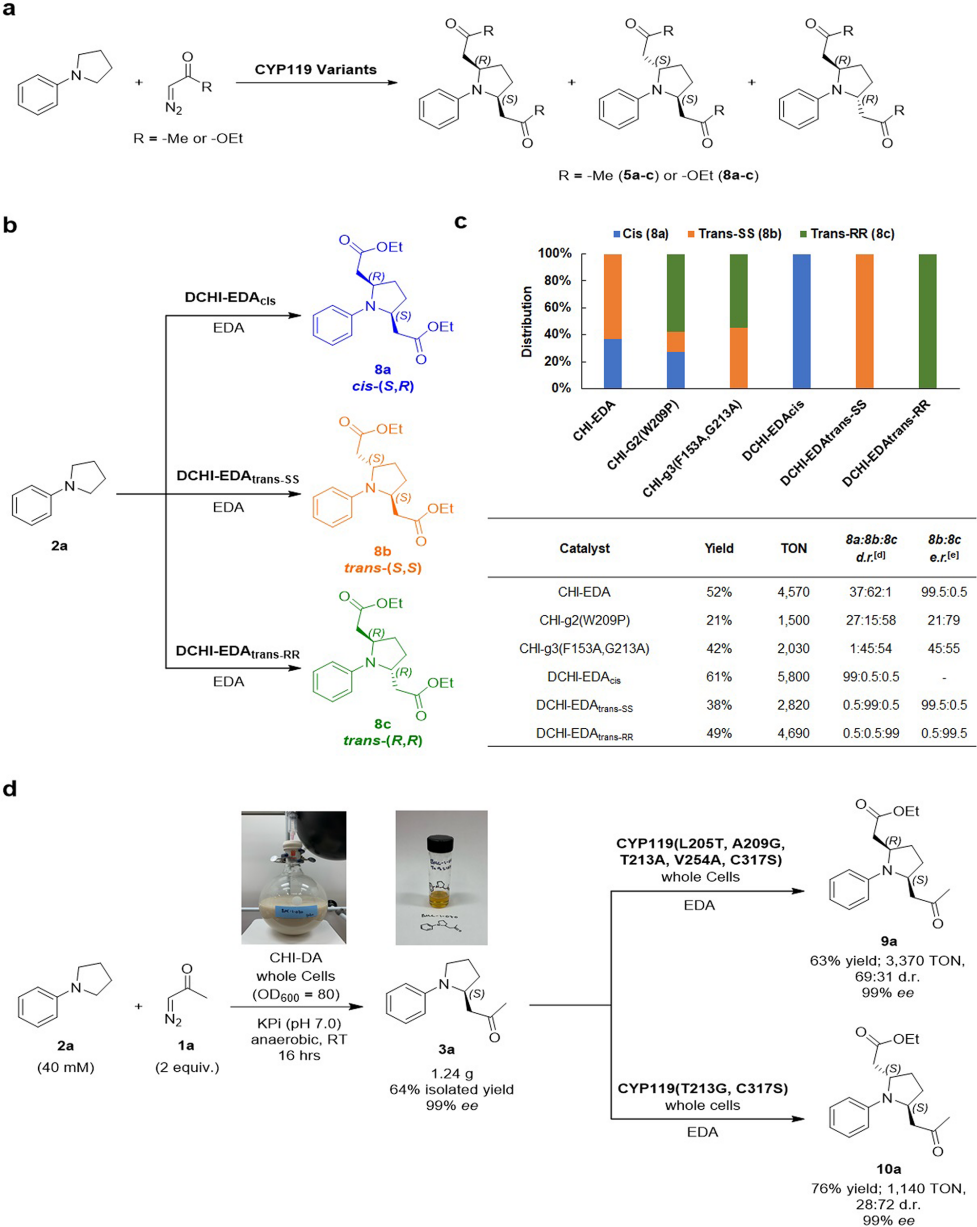
Stereodivergent dual α-C–H functionalization. (a) General scheme for dual C–H carbene insertion with diazoacetone and ethyl diazoacetate in *N*-phenylpyrrolidine. (b, c) One-pot dual C–H functionalization of **2a** with EDA using CYP119 with stereodivergent selectivity. Whole-cell reactions were carried out under standard reaction conditions with EDA, as described in Table 2 but using 40 mM EDA. The graph reports the relative distribution of products **8a**–**c** for the three DCHI-EDA variants, along with three other representative CYP119 variants. Assay yields (GC) and TON values reported in the table correspond to the double C–H functionalization product. (d) Tandem dual C–H functionalization of **2a** with diazoacetone and EDA. The EDA reaction was carried out using standard reaction conditions, as described in Table 2. Diastereomeric and enantiomeric ratios were determined by chiral GC and SFC.

During the screening of the engineered CYP119 libraries, we noticed the presence of highly active variants capable of catalyzing a double C–H insertion in *N*-phenylpyrrolidine with both diazoacetone and EDA. These double insertion products were isolated and determined by NMR spectroscopy to correspond to 2,5-disubstituted products as both *cis* and *trans*-isomers (**5a**–**c** and **8a**–**c**; Figure 6a). We further noticed that the ratio between the *cis* and *trans*-isomers varied among different CYP119 variants, suggesting that the stereoselectivity of the double insertion reaction can be tuned via protein engineering. Accordingly, we sought to extend the scope of the present methods to the stereodivergent dual C–H functionalization through both a one-pot/single diazo reagent reaction and via a tandem process with two different diazo reagents.

Toward the former goal, the engineered CYP119 libraries were screened for the functionalization of **2a** with EDA in the presence of excess diazo compound (4 equiv). From this screening, diastereo- and enantiodivergent CYP119 variants capable of selectively yielding each of the three possible stereoisomeric products, namely, *cis*-(*S*,*R*)-**8a**, *trans*-(*S*,*S*)-**8b**, and *trans*-(*R*,*R*)-**8c**, were identified (Figure 6b,c). For the formation of meso compound *cis*-(*S*,*R*)-**8a**, CYP119 (T213A, C317S) (named “DCHI-EDA_*cis*_”) showed excellent diastereoselectivity (99:0.5:0.5 d.r.) along with high activity (61% yield) (Figure 6c). On the other hand, the two *trans*-enantiomers *trans*-(*S*,*S*)-**8b**, and *trans*-(*R*,*R*)-**8c** could be obtained with excellent diasteroselectivity and enantioselectivity (99% *ee*) using two related variants, i.e., CYP119 (F153Y, T213G, V254A, C317S) (called “DCHI-EDA_*trans-SS*_”) and CYP119 (F153A, T213G, V254A, C317S) (named “DCHI-EDA_*trans-RR*_”) (Figure 6c). Interestingly, a complete inversion in the enantiopreference in these enzymes can be ascribed to a single mutation in position 153 (Ala vs Tyr). Albeit with lower diastereoselectivity than DCHI-EDA_*trans*-*SS*_, CHI-EDA shows also high activity and excellent enantioselectivity for the formation of *trans*-(*S*,*S*)-**8b** in the presence of excess EDA (99% *ee*, Figure 6c).

These findings then prompted us to pursue a strategy for achieving a stereoselective dual C–H functionalization of the cyclic amine substrate via a combination of the two methodologies for C–H insertion with diazoacetone and with EDA described above. To this end, a gram-scale synthesis of the diazoacetone insertion product **3a** was carried out via whole-cell biotransformation of **2a** with diazoacetone with *E. coli* cells expressing variant CHI-DA, resulting in the isolation of 1.24 g of **3a** in high enantiopurity (>99% *ee*) and 64% isolated yield (Figure 6d). The enzymatic product was then applied to screen the CYP119 libraries for variants capable of catalyzing the α-C–H functionalization of this substrate to produce the difunctionalized products **9** and **10** with diastereodivergent selectivity (Figure 6d). While multiple variants accepted **3a** to produce the desired difunctionalized products, CYP119 (L205T, A209G, T213A, V254A, C317S) was found to catalyze the efficient and selective formation of the *cis* product **9** (69:31 d.r., 63% yield), whereas CYP119 (T213G, C317S) offered complementary diastereoselectivity in this reaction by favoring the formation of *trans*-product **10** (72:28 d.r., 76% yield). Both products were obtained in high enantiopurity (99% *ee*) owing to the excellent enantioselectivity stereocontrol of the prior CHI-DA catalyzed step.

Beside representing first examples of a dual enzyme-catalyzed C–H carbene insertion on a single substrate, these results also illustrate the value of the present strategies toward enabling the synthesis of stereoisomeric and enantioenriched compounds decorated with multiple functional groups (e.g., ester/keto group) that are readily amenable for further functionalization. Among other applications, these types of compounds can be valuable building blocks for generating stereoisomeric libraries in drug discovery campaigns.^63,64^

### Late-Stage C–H Functionalization of Advanced Pharmaceutical Intermediate

**Figure 7:**
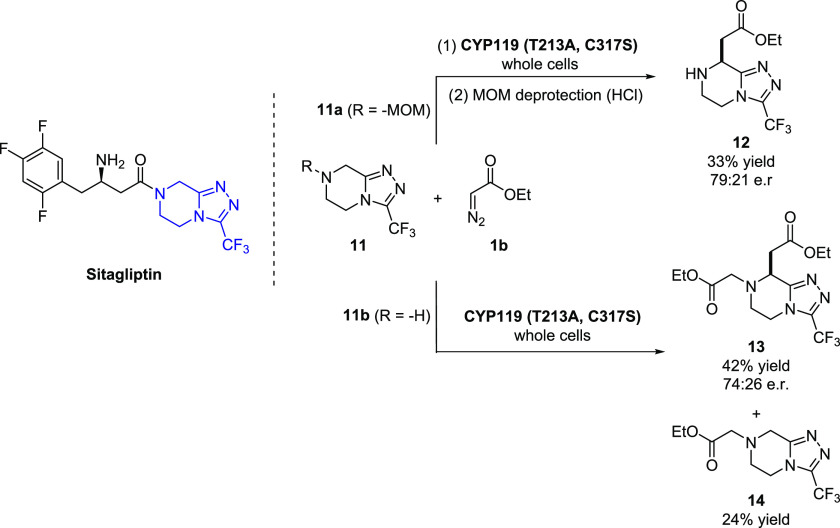
Late-stage enzymatic C–H functionalization of sitagliptin core with engineered CYP119 catalysts.

In the interest of determining whether the present methodologies could be extended to late-stage functionalization of drug scaffolds, we targeted the core structure of the antidiabetic drug sitagliptin (Januvia) (Figure 7) for C–H functionalization. In addition to the presence of a metal coordinating group (triazole), this molecule features a fused piperazine ring that contains multiple α-C–H bonds of similar reactivity, thus presenting a challenge in terms of regioselectivity for late-stage functionalization via chemical means. To identify catalysts for its modification, the MOM-protected compound **11a** was screened against the library of engineered CYP119 variants in the presence of EDA. Gratifyingly, CYP(T213A, C317S) was found to catalyze the C(sp^3^)–H functionalization of the drug core molecule to give **12** in 79:21 e.r. and 25% yield after the removal of the MOM protecting group (Figure 7). Notably, the same enzyme was able to accept the unprotected core (**11b**) to produce the dual N–H/C–H insertion product **13** with similar enantioselectivity (74:26 e.r.), along with the N–H insertion product **14** (Figure 7). Altogether, these results provide a proof-of-principle demonstration of the value of the present biocatalytic strategy for late-stage functionalization of drug scaffolds.

## Conclusions

In summary, we have reported the development of a highly enantioselective, biocatalytic strategy for the asymmetric α-C–H functionalization of pyrrolidines with diazoacetone as the carbene donor reagent. In particular, CYP119-derived biocatalysts were evolved to catalyze this challenging reaction with high efficiency and high enantioselectivity across multiple substrates (up to 99:1 e.r.), supporting over 20,000 catalytic turnovers. The latter corresponds to the highest catalytic activity for an enzyme-catalyzed intermolecular carbene C–H insertion reaction reported to date, and it compares well with the highest TON values reported for native monooxygenation reactions catalyzed by engineered P450s.^65−73^ These biocatalytic reactions can be performed in whole cells and are readily scalable, as illustrated by gram-scale synthesis of **3a** (Figure 6). This methodology was further extended to the enantioselective α-C–H functionalization of a range of cyclic amine substrates, including pyrrolidine, piperidine, and morpholine scaffolds, using EDA. Furthermore, by combining the two methodologies, it was possible to accomplish both a one-pot and a tandem dual α-C–H functionalization with diastereo- and enantiodivergent selectivity, providing access to valuable polyfunctionalized building blocks in different stereochemical configurations. Finally, this approach was successfully applied to the selective late-stage C–H functionalization of the core structure of a drug molecule (sitagliptin), providing a direct path for its functionalization or diversification. Complementing other biocatalytic strategies,^74−78^ these biocatalysts are expected to expand opportunities for the synthesis and diversification of bioactive molecules containing saturated *N*-heterocycles, and we anticipate that iron-based CYP119-derived catalysts can be leveraged for other types of synthetically useful carbene C–H insertion reactions.

## Experimental Procedures

### General Information

All chemicals were purchased from commercial sources or provided by Pfizer and used without further purification. Substrates **2b**–**d** were synthesized according to the reported procedure. Unless stated otherwise, all column purification were performed on a Biotage Selekt Flash Chromatography, eluted with ethyl acetate: hexane, 0–15% gradient, detected by UV absorption at 254 nm. Thin layer chromatography (TLC) was carried out using Merck Millipore TLC silica gel 60 F254 glass plates. ^1^H, ^13^C, and ^19^F NMR spectra were measured on a Bruker DPX-400 instrument (operating at 400 MHz for ^1^H, 100 MHz for ^13^C, and 375 MHz for ^19^F) or a Bruker DPX-500 instrument (operating at 500 MHz for ^1^H and 125 MHz for ^13^C).

### Protein Expression

Wild-type and engineered CYP119 variants were expressed in *E. coli* C41(DE3) cells as follows. After transformation, cells were grown in LB medium (ampicillin, 100 mg L^–1^) at 37 °C (200 rpm) until OD_600_ reached 1 to 1.2. Cells were then induced with 0.25 mM isopropyl-β-d-1-thiogalactopyranoside (IPTG) and 0.3 mM δ-aminolevulinic acid (δ-ALA). After induction, cultures were shaken at 180 rpm and 27 °C and harvested after 20 h by centrifugation at 4000 rpm at 4 °C. The cells were resuspended in 20 mL of Ni-NTA Lysis Buffer (50 mM KPi, 250 mM NaCl, 10 mM histidine, pH 8.0). Resuspended cells were frozen and stored at −80 °C until purification. Cell suspensions were thawed at room temperature, lysed by sonication, and clarified by centrifugation (14,000 rpm, 50 min, 4 °C). The clarified lysate was transferred to a Ni-NTA column equilibrated with Ni-NTA Lysis Buffer. The resin was washed with 50 mL of Ni-NTA Lysis Buffer and then 50 mL of Ni-NTA Wash Buffer (50 mM KPi, 250 mM NaCl, 20 mM histidine, pH 8.0). Proteins were eluted with Ni-NTA Elution Buffer (50 mM KPi, 250 mM NaCl, 250 mM histidine, pH 7.0). After elution from the Ni-NTA column, the protein was buffer-exchanged against 50 mM KPi buffer (pH 7.0) using 10 kDa Centricon filters. The concentration of the CYP119 variants was determined from CO-binding assays (difference spectra) using ε_406_ = 100 mM^–1^ cm^–1^ as the extinction coefficient.

### Protein Engineering

Protein evolution was conducted through iterative rounds of site saturation mutagenesis based on active site residues, which showed different product profiles with their alanine scanning libraries. In each round, the mutagenesis was conducted using the Quickchange method.^79^ A mixture of DNA degenerate primers (KBG/WDC/MHG = 1:1:1) encoding (mostly) uncharged amino acid residues of variable size (Gly, Ala, Ser, Val, Leu, Trp, Thr, Asn, Gln, Cys, Pro, Ile, Met, Phe, Thr) were used, and the PCR products were transformed into *E. coli* DH5a cells after digestion with *Dpn*I restriction enzyme. The colonies were collected in LB medium (ampicillin, 100 mg L^–1^), and plasmids were extracted by QIAprep Spin Miniprep Kit (Cat No.27104). Library coverage was then assessed by DNA sequencing to confirm the incorporation of desired mutations. The library of CYP119 variants was then transformed into *E. coli* DH5α cells and the proteins expressed in 96-well plates under the conditions described above. After expression, the cells were pelleted by centrifugation and resuspended in KPi buffer (50 mM, pH 7). The reactions were initiated by adding substrate into each well of the plate in an anaerobic chamber, followed by shaking for 12 h. The reaction mixtures were extracted with dichloromethane (DCM) and analyzed by chiral GC-FID or chiral SFC. The CYP119 variant that showed improved activity and enantioselectivity was sequenced and used as the template for the next round of mutagenesis and protein evolution.

### Enzymatic Reactions

Analytical scale enzymatic reactions with purified proteins were carried out at a 500 μL scale using the CYP119 variant (or other protein), cyclic amine substrate, diazoacetone (or ethyl diazoacetate), and sodium dithionite Na_2_S_2_O_4_ at the concentrations indicated in the tables and legends. In a typical procedure, a solution of Na_2_S_2_O_4_ in potassium phosphate buffer (50 mM, pH 7.0) was degassed by bubbling argon into the mixture for 3 min in a sealed vial. A buffered solution containing the CYP119 variant was carefully degassed in a similar manner in a separate vial. The two solutions were then mixed via cannula transfer. Reactions were initiated by addition of *N*-phenylpyrrolidine derivative (from a 0.5 M stock solution in ethanol), followed by the addition of diazoacetone/ethyl diazoacetate (from a 0.5 M stock solution in ethanol) with a syringe, and the reaction mixture was stirred for 16 h at room temperature under positive argon pressure. For whole-cell experiments, reactions were carried out at a 500 μL scale using *E. coli*. whole cells expressing the CYP119 variant, cyclic amine substrate, and diazoacetone (or ethyl diazoacetate) at the concentrations indicated in the tables and legends. In a typical procedure, a sealed vial containing whole cells was degassed with argon for 3 min. The reactions were initiated by addition of *N*-phenylpyrrolidine derivative (from a 0.5 M stock solution in ethanol), followed by the addition of diazo compound (from a 0.5 M stock solution in ethanol) with a syringe. The reaction mixture was stirred for 16 h at room temperature under positive argon pressure. The TON for the whole-cell reactions was calculated based on CYP119 concentration in the reaction mixture as measured via UV–vis spectroscopy after cell lysis.

### Reaction Analysis

The reactions were analyzed by adding 25 μL of internal standard (benzodioxole, 50 mM in methanol) to a 500 μL aliquot of the reaction mixture, followed by extraction with 500 μL dichloromethane (DCM) and centrifugation at 14,000 rpm. The organic layer was collected and analyzed by GC for yield, and by chiral SFC for enantioselectivity. The TON for the whole-cell reactions was calculated based on CYP119 concentration in the reaction mixture as measured via UV–vis using the CO-binding assay (ε_406_ = 100 mM^–1^ cm^–1^) after cell lysis. Calibration curves of the different products were constructed using authentic standards from the whole-cell reactions (General Procedure A). Enantioselectivity was determined by SFC using a chiral column, as described in the analytical methods section (Supporting Information).

#### General Procedure A: Whole-Cell Biocatalytic Reactions for C–H Functionalization on a Preparative Scale

These reactions were carried out on a 40 mL scale using C41(DE3) *E. coli* cells expressing the CYP119 variant, 10 mM cyclic amine substrate, 20 mM diazo reagent (diazoacetone or EDA). In a typical procedure, the substrate (0.4 mmol in 1 mL of ethanol) was added slowly to a 125 mL Erlenmeyer flask containing a stirring suspension of CYP119-expressing cells (OD_600_ = 40 in KPi, pH 7) in an anaerobic chamber. After stirring for 5 min, 1.6 mL of 500 mM diazo solution (2 equiv) in ethanol was added into the Erlenmeyer flask, and then the flask was sealed with a rubber septum. The reaction mixture was stirred at room temperature overnight. The reaction mixture was extracted with ethyl acetate (100 mL × 3), and the combined organic layers were dried over MgSO_4_ and concentrated under reduced pressure. The TON for the whole-cell reactions was calculated based on CYP119 concentration in the reaction mixture as measured via UV–vis spectroscopy using the CO-binding assay (ε_406_ = 100 mM^–1^ cm^–1^) after cell lysis. The crude product was purified by flash column chromatography using silica gel and ethyl acetate/hexanes as the eluent to isolate the product. The purified product was characterized by NMR, gas chromatography–mass spectrometry (GC–MS), and chiral SFC for stereoselectivity determination, and they were used as authentic standards for the construction of the calibration curves (TON and % conversion determination). Functionalization of the sitagliptin core was carried out in a similar manner using CYP119 (T213A, C317S) expressing *E. coli* C41(DE3) cells (OD_600_ = 120), 2.5 mM **11a** or **11b**, 40 mM EDA, in KPi buffer (50 mM, pH 7), room temperature, 16 h, in an anaerobic chamber. Product yields and characterization data are provided in the Supporting Information.

#### General Procedure B: Whole-Cell Biocatalytic Reactions for Dual C–H Functionalization with EDA on a Preparative Scale

The same procedure was followed as General Procedure A, with the only difference being that 4 equiv of the EDA (3.2 mL of 0.5 M stock solution) were used instead of 2 equiv. The crude product was purified by flash column chromatography using silica gel and ethyl acetate/hexanes as the eluent to isolate the product. The purified product was characterized by NMR, GC–MS, and bychiral SFC for stereoselectivity determination, and they were used as authentic standards for the construction of the calibration curves (TON and % conversion determination). Product yields and characterization data for **8a**–**c** are provided in the Supporting Information.

#### Biocatalytic Synthesis of Difunctionalized Products **9a** and **10a**

These reactions were carried out in a two-step process. In the first step, *N*-phenylpyrrolidine (**2a**) (1.40 g in 2.5 mL of ethanol) was added slowly to a 125 mL Erlenmeyer flask containing a stirring suspension of *E. coli* cells expressing CHI-DA (190 mL, OD_600_ = 40 in KPi, pH 7) in an anaerobic chamber. After stirring for 5 min, 15 mL of 1 M diazoacetone (**1a**) (2 equiv) solution in ethanol was added dropwise into the Erlenmeyer flask, and then the flask was sealed with a rubber septum. The reaction mixture was stirred at room temperature overnight. The reaction mixture was extracted with ethyl acetate (3 × 100 mL), and the combined organic layers were dried over MgSO_4_ and concentrated under reduced pressure. The TON for the whole-cell reactions was calculated based on CYP119 concentration in the reaction mixture as measured via UV–vis spectroscopy using the CO-binding assay (ε_406_ = 100 mM^–1^ cm^–1^) after cell lysis. The crude product was purified by flash column chromatography using silica gel and ethyl acetate/hexanes (1:4) as the eluent to afford enantiopure **3a** (>99% *ee*) in 64% isolated yield (1.93 g). For the second step, **3a** (0.4 mmol in 1 mL of ethanol) was added slowly to a 125 mL Erlenmeyer flask containing a 40 mL solution of *E. coli* cells (OD_600_ = 40 in KPi, pH 7) expressing the desired CYP119 variant (as shown in Figure 7) in an anaerobic chamber. After stirring for 5 min, 1.6 mL of 500 mM EDA (**1b**) (2 equiv) solution in ethanol was added to the cell suspension, and then the flask was sealed with a rubber septum. The reaction mixture was stirred at room temperature overnight. The reaction mixture was extracted with ethyl acetate (100 mL × 3), and the combined organic layers were dried over MgSO_4_ and concentrated under reduced pressure. The TON for the whole-cell reactions was calculated based on CYP119 concentration in the reaction mixture as measured via UV–vis spectroscopy using the CO-binding assay (ε_406_ = 100 mM^–1^ cm^–1^) after cell lysis. The crude product was purified by flash column chromatography using silica gel and ethyl acetate/hexanes (1:4) as the eluent to afford **9a** in 63% isolated yield and **10a** in 76% isolated yield. The purified product was characterized by NMR, GC–MS, and chiral SFC for stereoselectivity determination, and they were used as authentic standards for the construction of the calibration curves (TON and % conversion determination).

## Supporting Information Available

Supporting information includes supplementary tables and figures, chiral GC and SFC chromatograms, synthetic procedures, compound characterization data, NMR spectra, and crystallographic data (PDF)
