# Supplementary material for: Enantioselective Single and Dual α-C–H Bond Functionalization of Cyclic Amines via Enzymatic Carbene Transfer
Source: J Am Chem Soc. 2022 Dec 21;145(1):537–50. doi: 10.1021/jacs.2c10775 (PMC9837850; doi:10.1021/jacs.2c10775)

Supporting Information

Enantioselective Single and Dual α-C–H bond Functionalization of Cyclic Amines via Enzymatic Carbene Transfer

Xinkun Ren[1,3], Bo M. Couture[1], Ningyu Liu[1], Manjinder S. Lall[2], Jeffrey T. Kohrt[2], and Rudi Fasan[1,]*

[1] *Department of Chemistry, University of Rochester, Rochester, NY 14627, United States.*

[2] *Pfizer Inc., Medicine and Design, Groton, CT 06340, United States.*

[3] *Current address: College of Engineering and Applied Sciences, National Laboratory of Solid State Microstructures, Nanjing University, Nanjing, Jiangsu Province 210023, China.*

Table of Contents

Supplementary Tables S1-S6	Pages S2-S11
Supplementary Figures S1-S7	Pages S12-S38
X-Ray Crystallographic Analyses	Pages S39-S40
Analytical Methods	Pages S41-S43
Additional Synthetic Procedures	Pages S43-S44
Compound Characterization Data	Pages S45-S56
NMR Data	Pages S57-S121

Table S1. Activity of metal catalysts, hemin, and hemoproteins in the intermolecular C–H carbene insertion reaction of *N*-phenylpyrrolidine (**2a**) with diazoacetone (**1a**). Reaction conditions: protein (or hemin) at 20 μM, 10 mM **2a**, 20 mM **1a**, 10 mM $Na_2S_2O_4$, in KPi buffer (50 mM, pH 7.0), room temperature, 16 hours, in anaerobic chamber. Chemical catalysts were used at 10 mol% of *N*-phenylpyrrolidine in DCM.

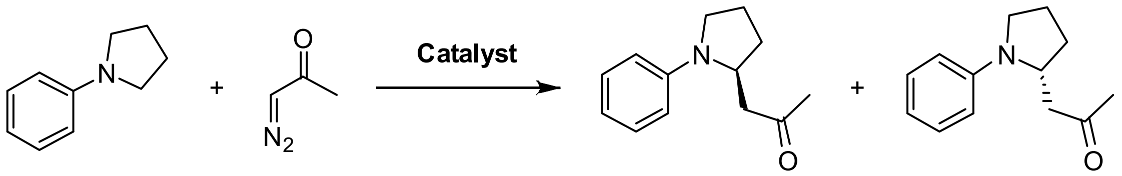

2a **1a** **3a** **4a**

Entry	Catalyst	Yield[a]	TON[b]
1	Hemin	0%	0
2	Sperm whale Mb	0%	0
3	$P450_{BM3}$	0%	0
4	$P450_{cam}$	0%	0
5	P450 XplA	0%	0
6	CYP119 (WT)	0%	0
7	Fe(TPP)Cl in DCM	0%	0
8	CuI in DCM	0%	0
9	$Rh(OAc)_2$ in DCM	0%	0

[a] Conversion as determined by GC. [b] TON as calculated based on the protein concentration measured from cell lysate.

Table S2. Screening of engineered hemoprotein variants for the intermolecular C–H insertion reactions of *N*-phenylpyrrolidine (**2a**) with diazoacetone (**1a**). Reaction conditions: CHI-DA expressing C41(DE3) *E. coli* cells, OD_{600} = 20, 10 mM substrate, 20 mM diazoacetone (**1a**), in KPi buffer (50 mM, pH 7.0), room temperature, 16 hours, in anaerobic chamber.

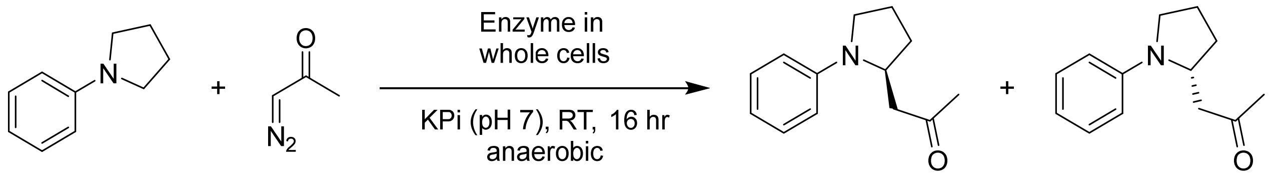

Entry	Enzyme	Yield[a]	TON[b]	e.r.[c] (3a:4a)
1	Mb WT	0%	0	n.d.
2	Mb(H64V, V68A)	0%	0	n.d.
3	Mb(H64G, V68A)	0%	0	n.d.
4	$P450_{BM3}$	0%	0	n.d.
5	P411-CHF	0%	0	n.d.
6	$P450_{cam}$	0%	0	n.d.
7	P450cam(P100A)	0%	0	n.d.
8	P450cam(T101A)	0%	0	n.d.
9	P450cam(F163A)	0%	0	n.d.
10	P450cam(L244A)	0%	0	n.d.
11	P450cam(L245A)	0%	0	n.d.
12	P450cam(T252A)	0%	0	n.d.
13	P450cam(V253A)	0%	0	n.d.
14	P450cam(L294A)	0%	0	n.d.
15	P450cam(V295A)	0%	0	n.d.

16	P450cam(D297A)	0%	0	n.d.
17	P450cam(R299A)	0%	0	n.d.
18	P450cam(C357S)	0%	0	n.d.
19	P450cam(P100A, C357S)	0%	0	n.d.
20	P450cam(T101A, C357S)	0%	0	n.d.
21	P450cam(F163A, C357S)	0%	0	n.d.
22	P450cam(L244A, C357S)	0%	0	n.d.
23	P450cam(L245A, C357S)	0%	0	n.d.
24	P450cam(T252A, C357S)	0%	0	n.d.
25	P450cam(V253A, C357S)	0%	0	n.d.
26	P450cam(L294A, C357S)	0%	0	n.d.
27	P450cam(V295A, C357S)	0%	0	n.d.
28	P450cam(D297A, C357S)	0%	0	n.d.
29	P450cam(R299A, C357S)	0%	0	n.d.
30	XplA WT	0%	0	n.d.
31	XplA(L238A)	0%	0	n.d.
32	XplA(V387A)	0%	0	n.d.
33	XplA(V391A)	0%	0	n.d.
34	XplA(M394A)	0%	0	n.d.
35	XplA(Q438A)	0%	0	n.d.
36	CYP119 WT	0%	0	n.d.
37	CYP119(L69A)	0%	0	n.d.
38	CYP119(F153A)	0%	0	n.d.
39	CYP119(L205A)	0%	0	n.d.
40	CYP119(T213A)	0%	0	n.d.

41	CYP119(V254A)	0%	0	n.d.
42	CYP119(C317S)	0%	0	n.d.
43	CYP119(L69A, C317S)	0%	0	n.d.
44	CYP119(F153A, C317S)	0%	0	n.d.
45	CYP119(L205A, C317S)	0%	0	n.d.
46	CYP119(T213A, C317S)	2%	166	n.d.
47	CYP119(V254A, C317S)	Trace	n.a.	n.d.

[a] Assay yield as determined by GC. [b] TON as calculated based on the protein concentration measured from cell lysate. [c] Enantiomeric ratio (e.r.) for **3a**:**4a** as determined by chiral SFC. N.d.= not determined.

Table S3. Substrate scope of CHI-DA for the α-C–H functionalization of different *N*-aryl-pyrrolidine in the presence of diazoacetone. Reaction conditions: CHI-DA expressing C41(DE3) *E. coli* cells, OD_{600} = 40, 10 mM substrate, 20 mM diazoacetone (**1a**), in KPi buffer (50 mM, pH 7.0), room temperature, 16 hours, in anaerobic chamber.

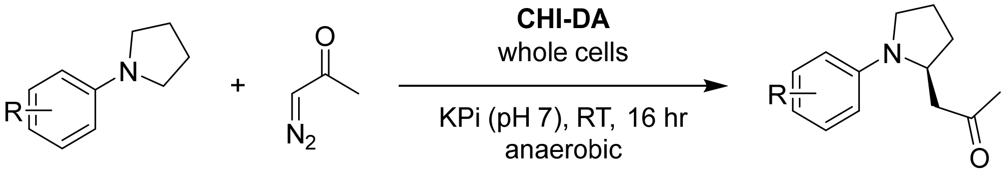

2b-2k **1a** **3b-3k**

**Substrate**	**Product**	**TON[a]**	**Yield[b]**	**e.r.[c] (3a:4a)**
**2b**	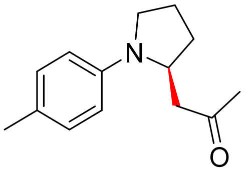 **3b**	9,340	93%	91:9
**2c**	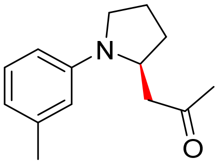 **3c**	8,020	80%	87:13
**2d**	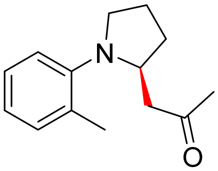 **3d**	4,480	45%	52:48
**2e**	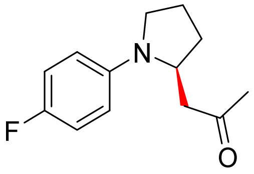 **3e**	5,150	51%	86:14
**2f**	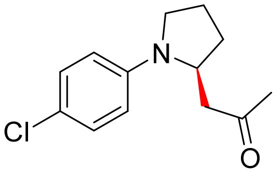 **3f**	6,170	62%	99:1

**2g**	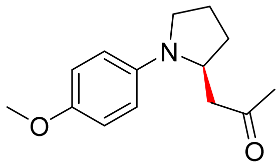 **3g**	5,240	52%	75:25
**2h**	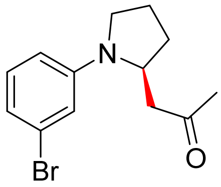 **3h**	497	5.0%	85:15
**2i**	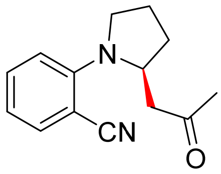 **3i**	574	5.7%	70:30
**2j**	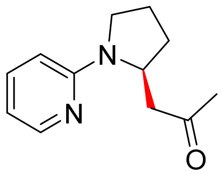 **3j**	1,960	20%	60:40
**2k**	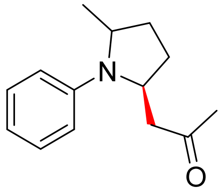 **3k**	2,040	20%	86:14 d.r.<br>60:40 e.r.
**2l**	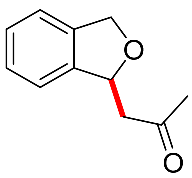 **3l**	5,760	58%	77:23

[a] TON as calculated based on the protein concentration measured from cell lysate. [b] Assay yield as determined by GC using calibration curves generated with isolated product. [c] Enantiomeric ratio (e.r.) for **3a**:**4a** as determined by chiral SFC.

Table S4. Activity of metal catalysts, hemin and hemoproteins in the intermolecular C–H insertion reactions of *N*-phenylpyrrolidine (**2a**) with ethyl diazoacetate (EDA, **1b**)[a]. Reaction conditions: protein (or hemin) at 20 µM, 10 mM **2a**, 20 mM EDA (**1b**), 10 mM $Na_2S_2O_4$, in KPi buffer (50 mM, pH 7.0), room temperature, 16 hours, in anaerobic chamber. Chemical catalysts were used at 10 mol% in DCM.

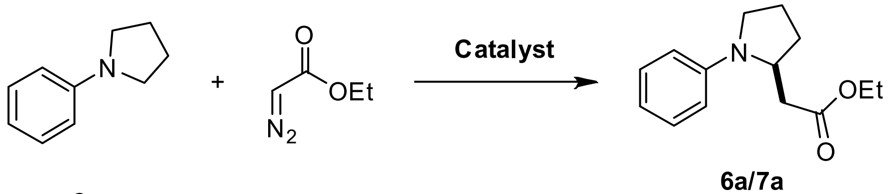

2a **1b** **6a/7a**

Entry	Catalyst	Yield[a]	TON[b]	e.r.
1	Hemin	0%	0	-
2	Mb (sperm whale)	0%	0	-
3	P450 XplA	0%	0	-
4	$P450_{BM3}$	0%	0	-
6	$P450_{cam}$	0%	0	-
7	CuI in DCM	0%	0	-
8	$Rh(OAc)_2$ in DCM	0%	0	-
9	Fe(TPP)Cl in DCM	0%	0	-

[a] Conversion as determined by GC. [b] TON as calculated based on the protein concentration measured from cell lysate.

Table S5. Screening of CYP119 variants for the intermolecular C–H insertion reactions of *N*-phenylpyrrolidine (**2a**) with EDA (**1b**). Reaction conditions: protein expressing C41(DE3) *E. coli* cells, OD_{600} = 20, 10 mM substrate, 20 mM ethyl diazoacetate (**1b**), in KPi buffer (50 mM, pH 7.0), room temperature, 16 hours, in anaerobic chamber.

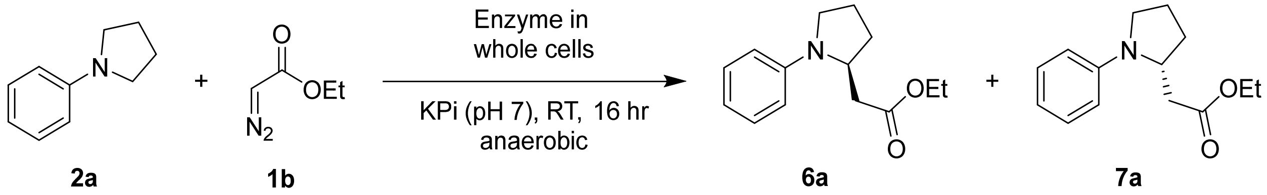

Entry	Catalyst	Yield[a]	TON[b]	e.r.[c] (6a:7a)
1	CYP119	3.0%	133	n.d.
2	CYP119(L69A)	4.5%	200	n.d.
3	CYP119(F153A)	3.1%	137	n.d.
4	CYP119(L205A)	3.7%	164	n.d.
5	CYP119(T213A)	5.8%	256	n.d.
6	CYP119(V254A)	4.1%	181	n.d.
7	CYP119(T257A)	trace	n.a.	n.d.
8	CYP119(C317S)	4.8%	210	n.d.
9	CYP119(L69A, C317S)	3.3%	145	n.d.
10	CYP119(F153A, C317S)	7.6%	336	n.d.
11	CYP119(L205A, C317S)	8.0%	353	n.d.
12	CYP119(T213A, C317S)	18.5%	812	71:29
13	CYP119(V254A, C317S)	1.8%	79	n.d.
14	CYP119(T257A, C317S)	3.6%	157	n.d.

[a] Conversion as determined by GC. [b] TON as calculated based on the protein concentration measured from cell lysate. [c] Enantiomeric ratio (e.r.) for **3a**:**4a** as determined by chiral SFC. N.d.= not determined.

Table S6. Activity of selected CYP119 variants identified during the catalyst optimization process for the intermolecular C–H insertion reaction of *N*-phenylpyrrolidine (**2a**) with EDA (**1b**). Reaction conditions: protein expressing C41(DE3) *E. coli* cells, OD_{600} = 20, 10 mM substrate, 20 mM ethyl diazoacetate (**1b**), in KPi buffer (50 mM, pH 7.0), room temperature, 16 hours, in anaerobic chamber.

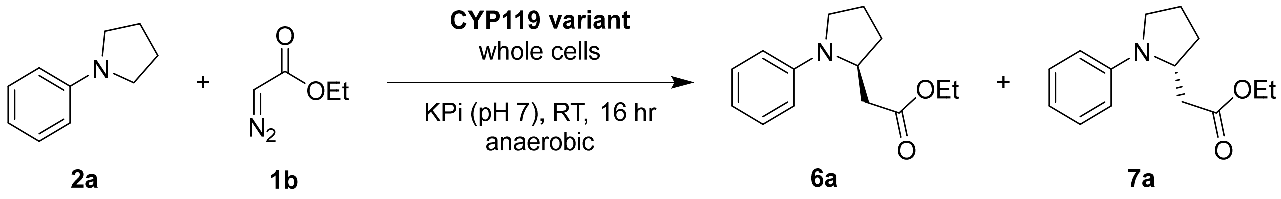

Entry	Mutations vs WT CYP119	Yield[a]	e.r.[b] (6a:7a)
1	-	3.0%	n.d.
2	C317S	4.8%	n.d.
3	T213A	5.8%	n.d.
4	T213A, C317S	18%	71:29
5	T213A, V254A	31%	n.d.
6	T213A, V254A, C317T	4%	n.d.
7	T213A, V254A, C317Y	5%	n.d.
8	T213A, V254A, C317D	6%	n.d.
9	T213A, V254A, C317E	2%	n.d.
10	T213A, V254A, C317K	3%	n.d.
11	T213A, V254A, C317R	7%	n.d.
12	T213A, V254A, C317H	5%	n.d.
13	T213A, V254A, C317S	99%	79:21
14	T213A, V254G, C317S	60%	n.d.
15	F153A, T213A, C317S	32%	n.d.

16	F153V, T213A, C317S	25%	n.d.
17	F153G, T213A, C317S	36%	n.d.
18	F153G, T213A, V254A, C317S	89%	87:13

[a] Conversion as determined by GC. [b] Enantiomeric ratio (e.r.) for **6a**:**7a** as determined by chiral SFC. N.d. = not determined.

Figure S1. Time-course analysis of CHI-DA-catalyzed intermolecular C–H insertion reactions of *N*-phenylpyrrolidine (**2a**) with diazoacetone (**1a**). Conversion was determined by gas chromatography using calibration curves with **3a**. Reaction conditions: CYP119 expressing *E. coli* C41(DE3) cells at OD_{600} = 40, 10 mM **2a**, 20 mM diazoacetone, in oxygen-free potassium phosphate buffer (50 mM, pH 7.0). Mean values and standard errors are derived from experiments performed in duplicate.

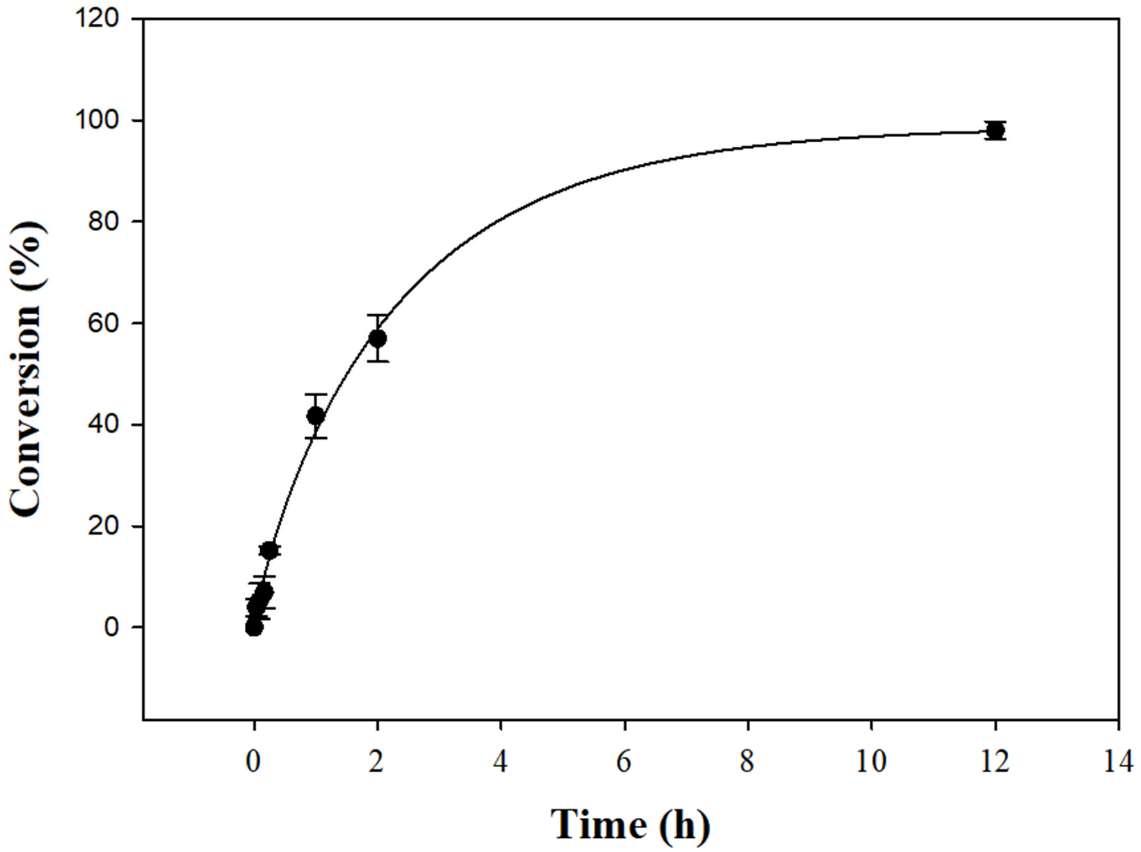

Figure S2. Time-course analysis of CHI-EDA-catalyzed intermolecular C–H insertion reactions of *N*-phenylpyrrolidine (**2a**) with ethyldiazoacetate (**1b**). Conversion was determined by gas chromatography using calibration curves with **6a**. Reaction conditions: CYP119 expressing *E. coli* C41(DE3) cells at OD_{600} = 40, 10 mM **2a**, 20 mM EDA, in oxygen-free potassium phosphate buffer (50 mM, pH 7.0). Mean values and standard errors are derived from experiments performed in duplicate.

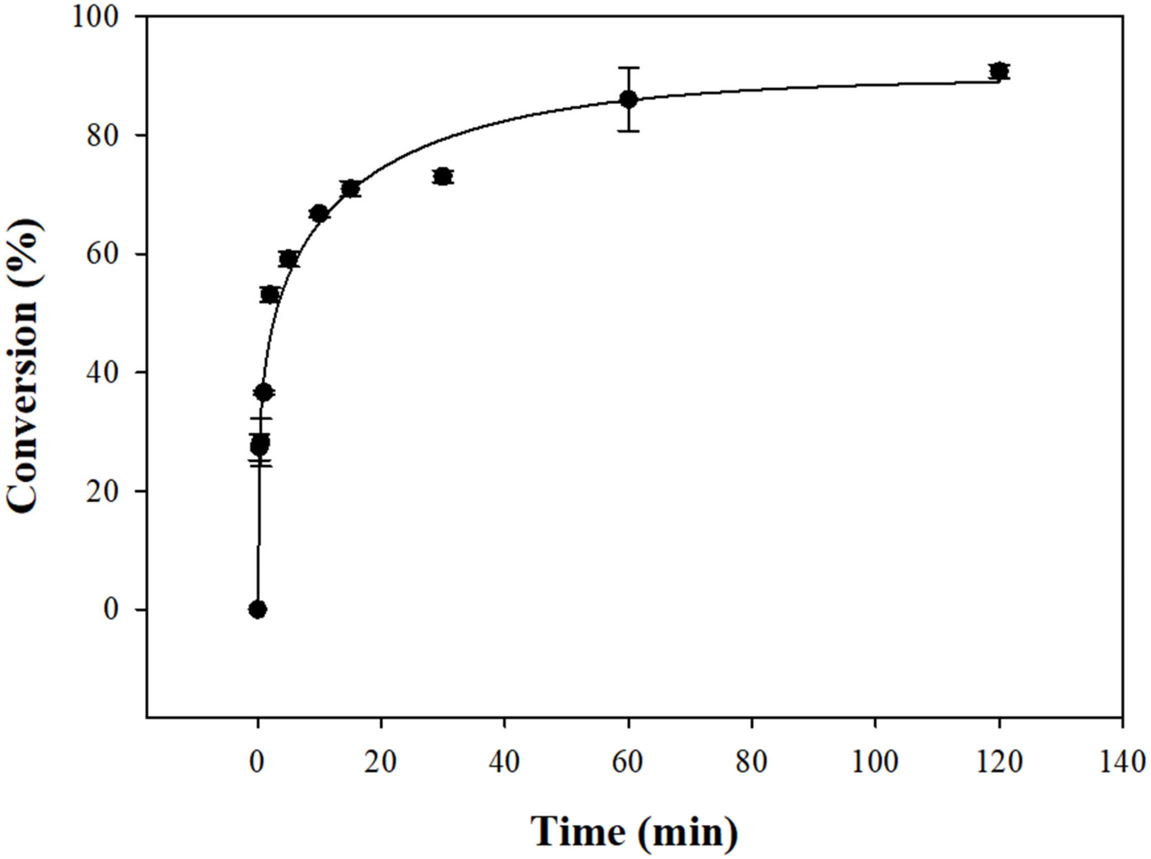

Figure S3. Screening of CYP119(T213A, V254A)-derived axial ligand variants for the intermolecular C–H insertion reaction of *N*-phenylpyrrolidine (**2a**) with EDA (**1b**). Reaction conditions: protein expressing C41(DE3) *E. coli* cells, OD_{600} = 20, 10 mM substrate, 20 mM ethyl diazoacetate (**1b**), in KPi buffer (50 mM, pH 7.0), room temperature, 16 hours, in anaerobic chamber.

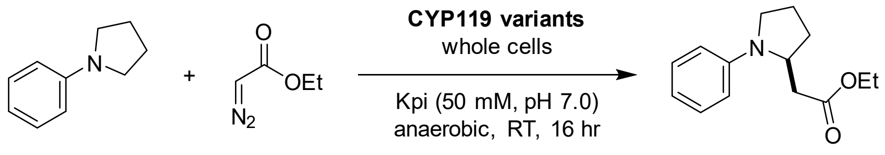

2a **1b** **6a/7a**

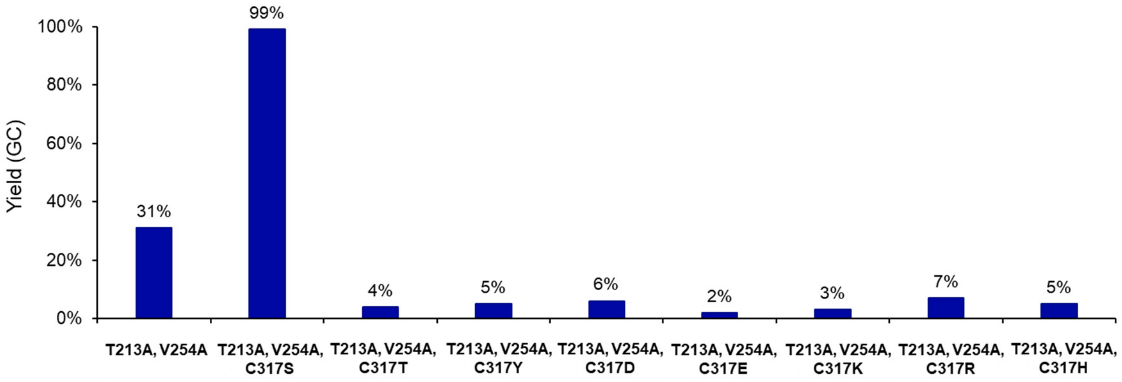

Figure S4. ORTEP for (*S*)-1-(1-(4-fluorophenyl)pyrrolidin-2-yl)propan-2-one (**3e**).

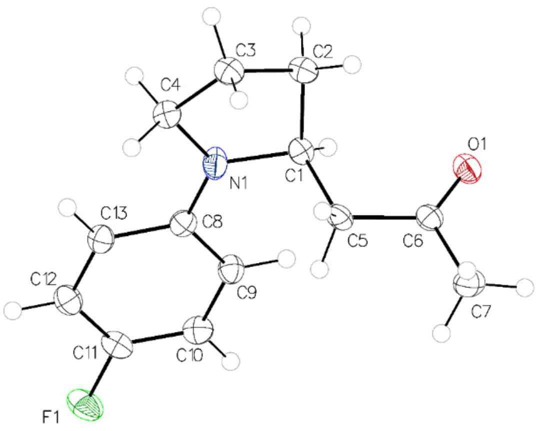

Figure S5. Chiral SFC analysis for the determination of an enantiomeric excess in the CYP119-catalyzed intermolecular C–H insertion reactions with diazoacetone (**1a**). The reference racemic samples were prepared with engineered CYP119 variant as described in the following figures.

- Chiral SFC analysis of racemic **3a** (using CYP119 F153R, T213A, V254A, C317S; *top*) and enantioenriched **3a** product (using CYP119 F153G, A209G, T213A, V254A, C317S; *bottom*):

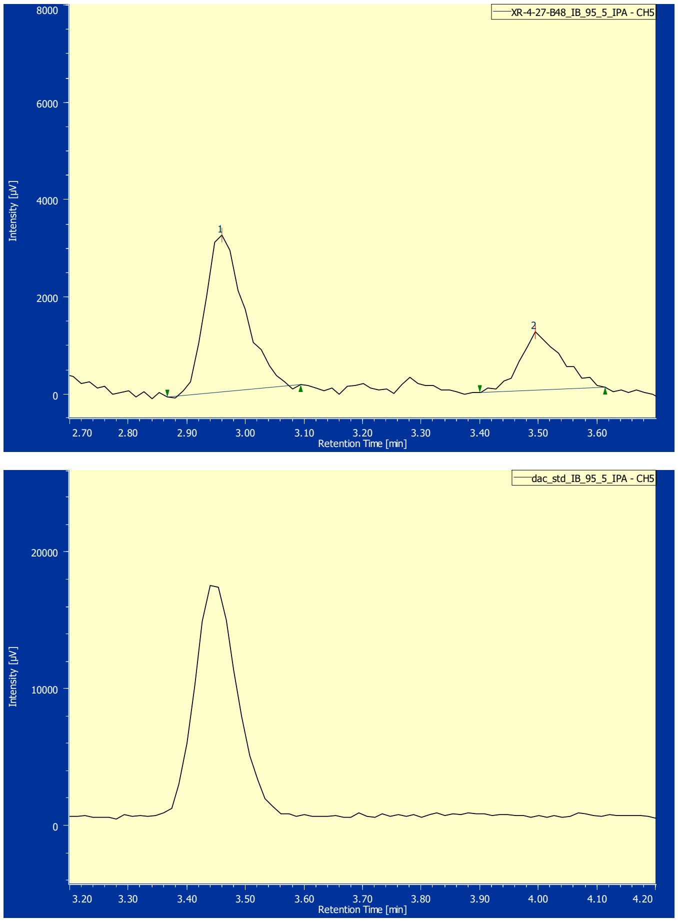

➢ Chiral SFC analysis of racemic **3b** (using CYP119 A209W, T213G, V254W, C317S; *top*) and enantioenriched **3b** product (using CYP119 F153A, A209G, T213A, V254A, C317S; *bottom*):

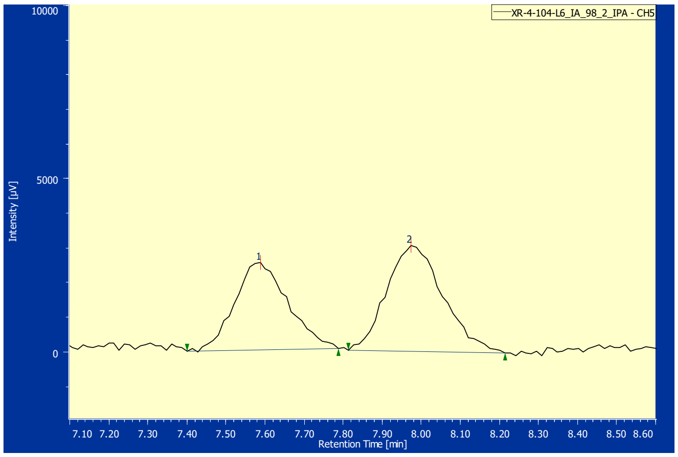

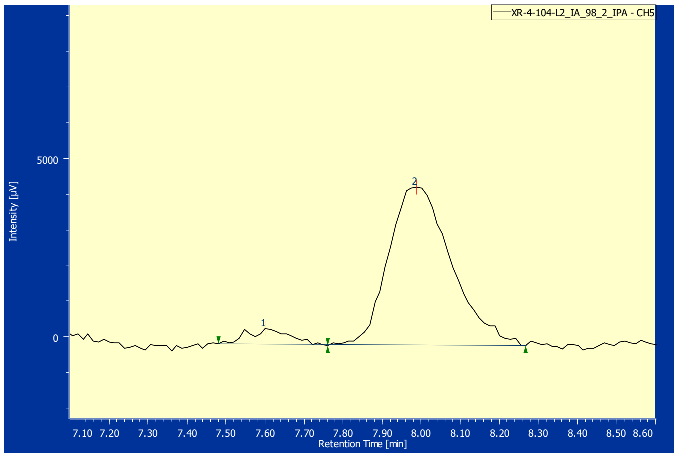

➢ Chiral SFC analysis of racemic **3c** (using CYP119 A209W, T213G, V254W, C317S; *top*) and enantioenriched **3c** product (using CYP119 L205V, A209G, T213A, V254A, C317S; *bottom*):

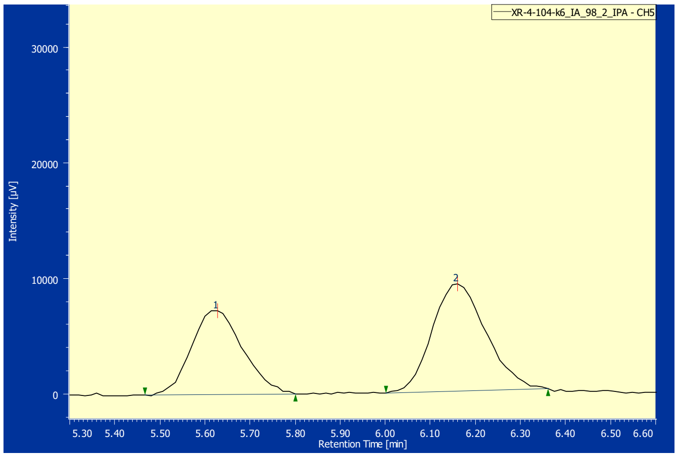

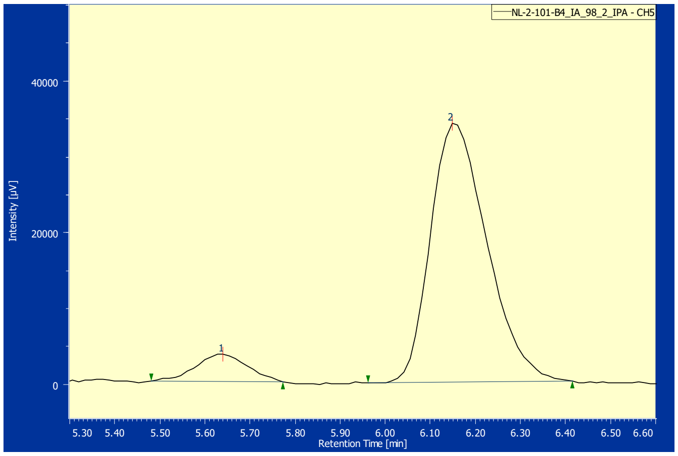

- Chiral SFC analysis of **3d** (using CYP119 F153G, A209G, T213A, V254A, C317S)

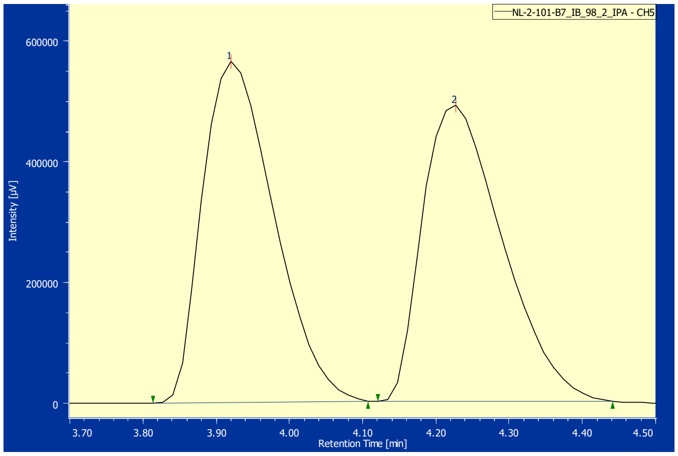

- Chiral SFC analysis of racemic **3e** (using CYP119 L205Y, A209G, T213A, V254A, C317S; *top*) and enantioenriched **3e** product (using CYP119 L205V, A209G, T213A, V254A, C317S; *bottom*):

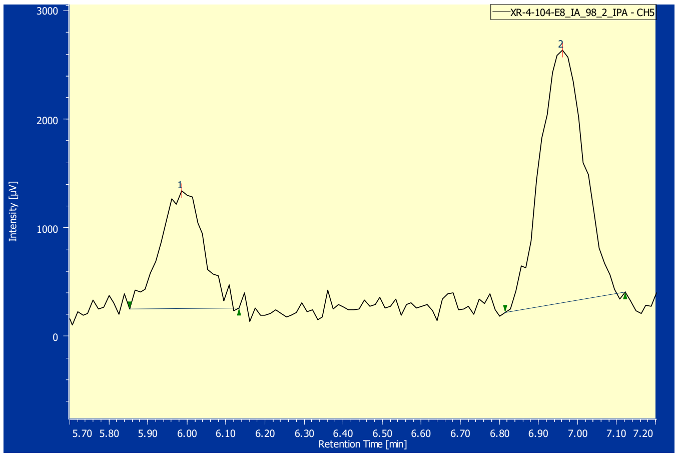

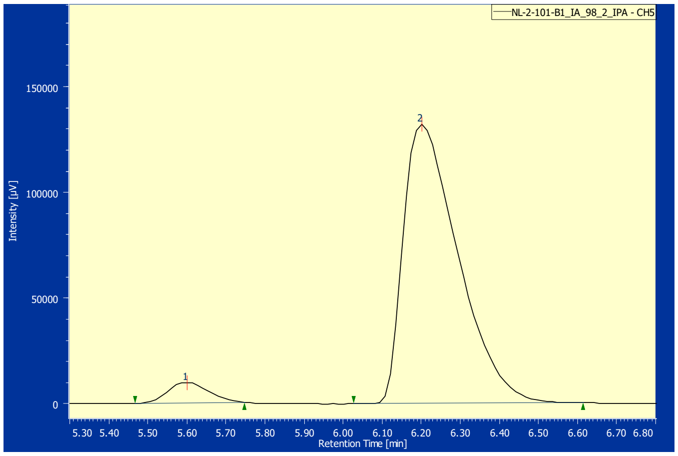

➢ Chiral SFC analysis of racemic **3f** (using CYP119 A209G, G210T, T213A, V254A, C317S; *top*) and enantioenriched **3f** product (using CYP119 F153G, A209G, T213A, V254A, C317S; *bottom*):

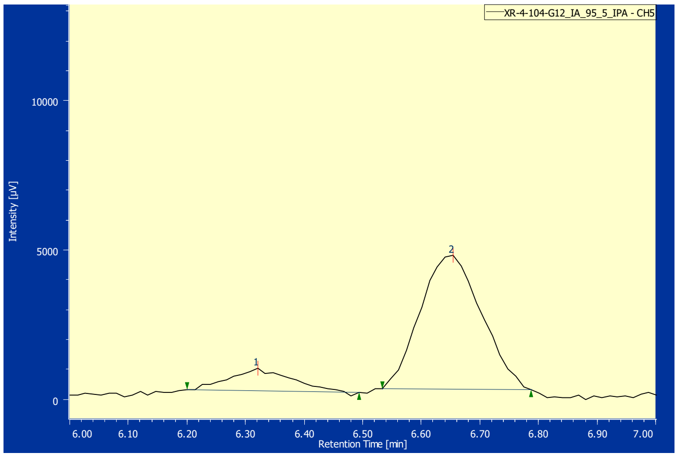

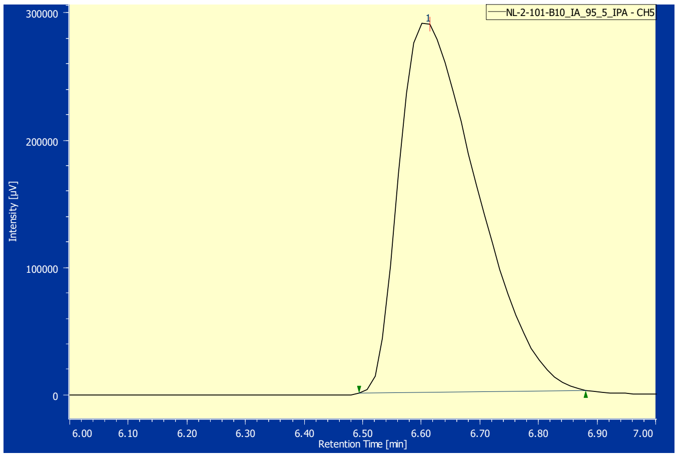

➢ Chiral SFC analysis of racemic **3g** (using CYP119 A209W, G210S, T213G, V254A, C317S; *top*) and enantioenriched **3g** product (using CYP119 F153A, A209G, T213A, V254A, C317S; *bottom*):

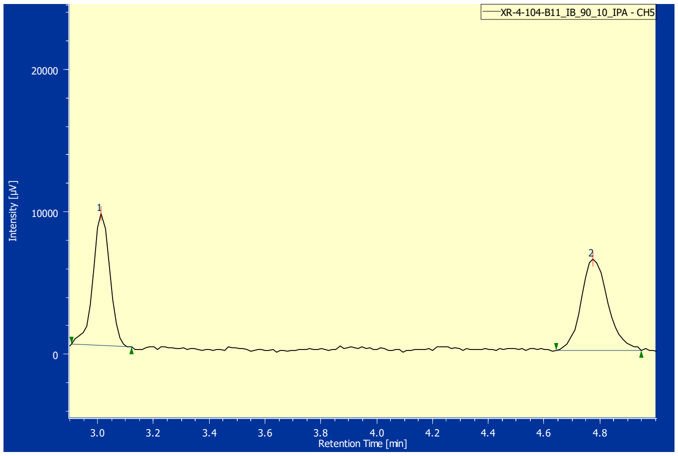

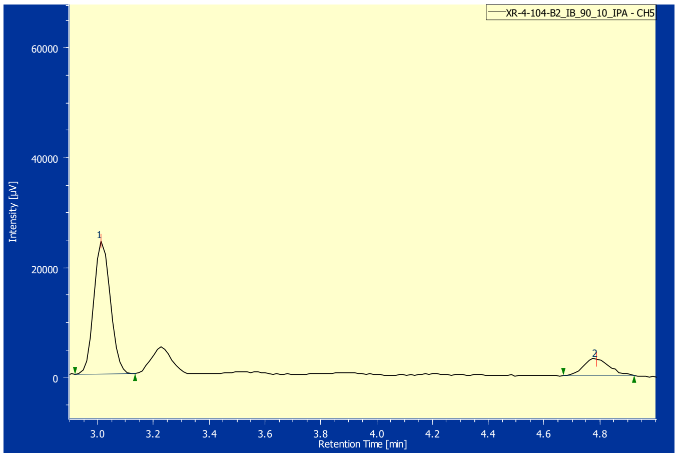

➢ Chiral SFC analysis of racemic **3h** (using CYP119 A209W, T213G, V254W, C317S; *top*) and enantioenriched **3h** product (using CYP119 L205V, A209G, T213A, V254A, C317S; *bottom*):

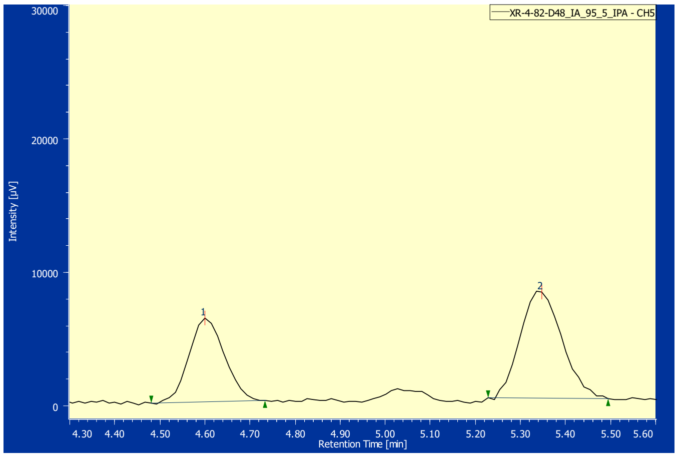

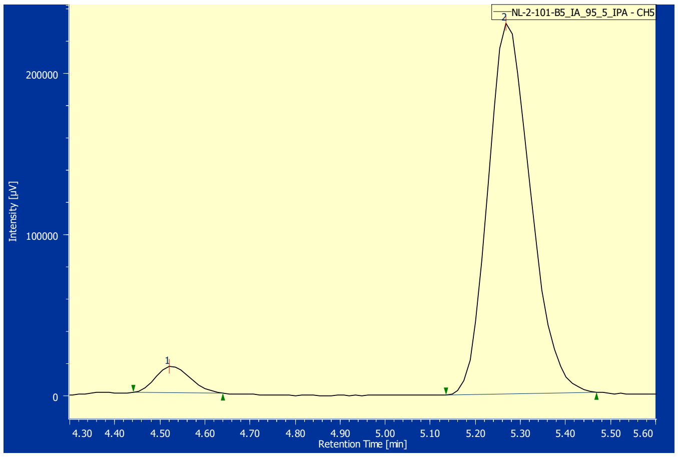

➢ Chiral SFC analysis of racemic **3i** (using CYP119 A209G, G210T, T213A, V254A, C317S; *top*) and enantioenriched **3i** product (using CYP119 A209W, T213G, V254W, C317S; *bottom*):

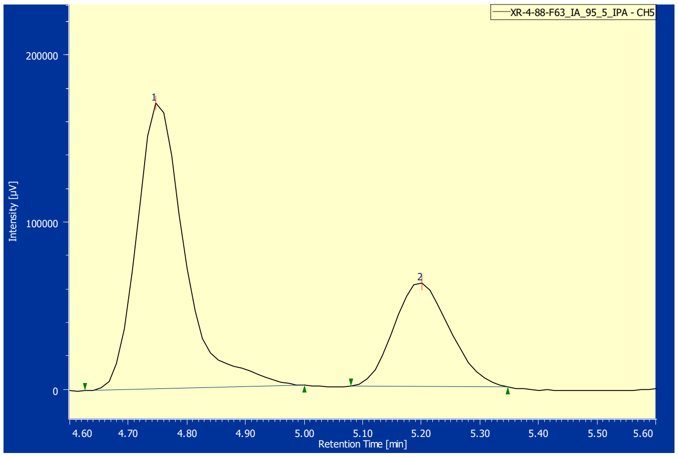

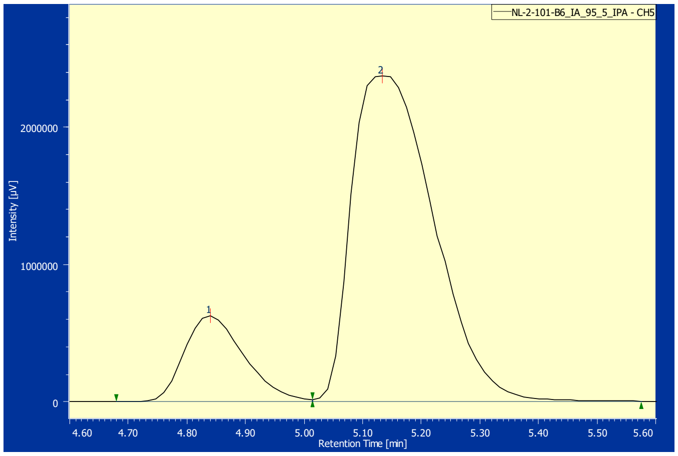

- Chiral SFC analysis of enantioenriched **3j** product (using CYP119 L205V, A209G, T213A, V254A, C317S; *bottom*):

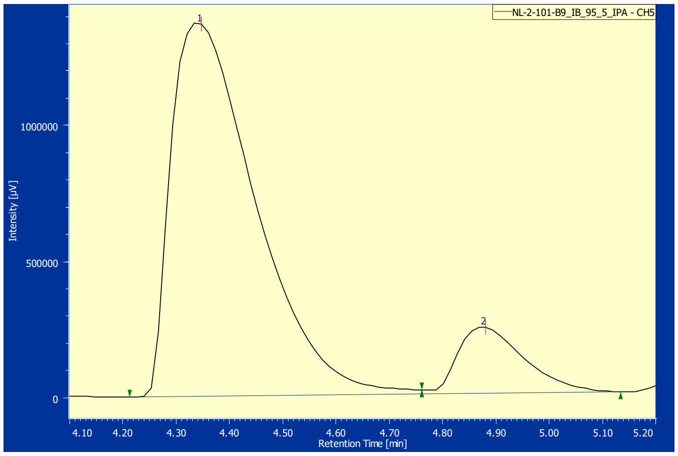

➢ Chiral SFC analysis of racemic **3k** (using CYP119 L205V, A209G, T213A, V254A, C317S; *top*) and enantioenriched **3k** product (using CYP119 A209G, T213A, V254A, C317S; *bottom*):

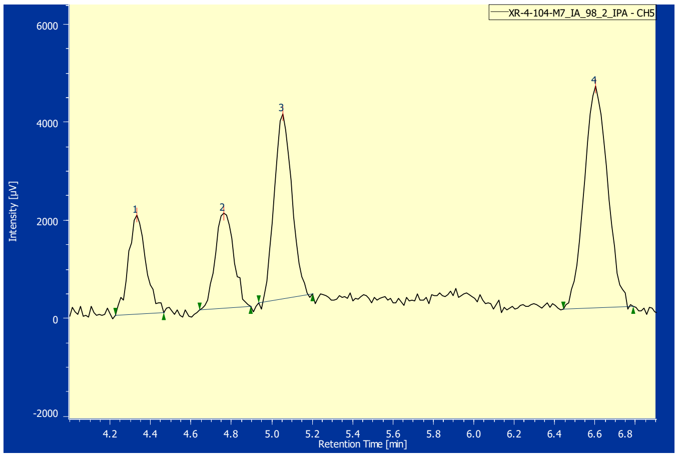

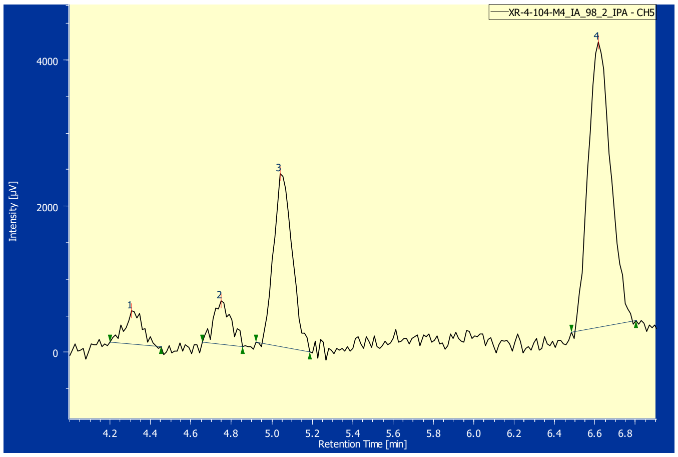

➢ Chiral SFC analysis of racemic **3l** (CYP119 L205Y, A209G, T213A, V254A, C317S; *top*) and enantioenriched **3l** product (using CYP119 A209G, T213G, V254A, C317S; *bottom*):

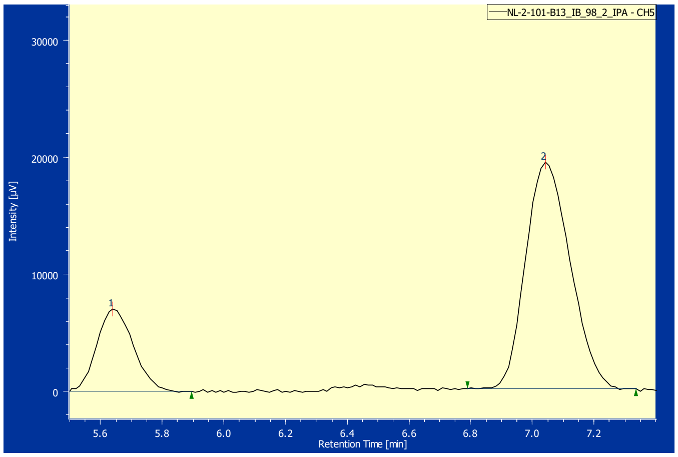

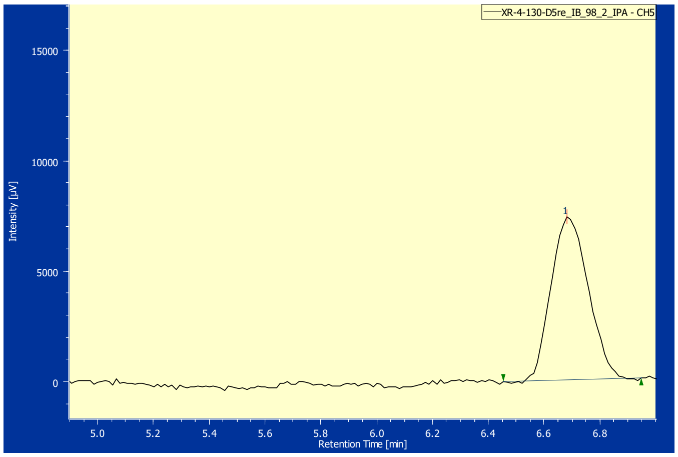

➢ Chiral SFC analysis of racemic **3m** product (using CYP119 A209G, T213G, V245A, C317S):

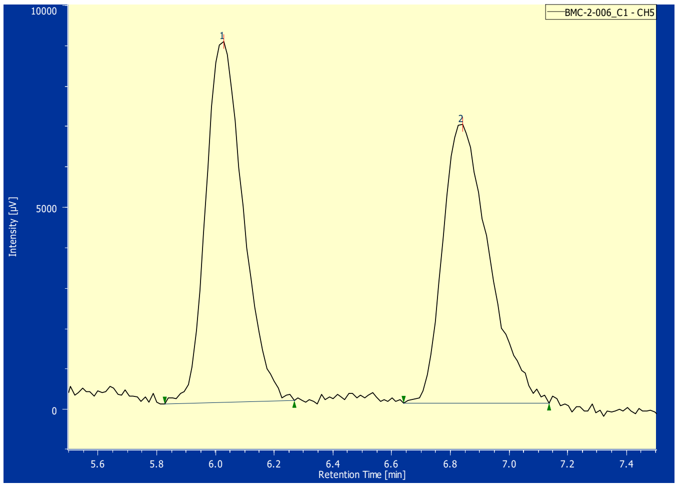

Figure S6. Chiral SFC analysis for the determination of an enantiomeric excess in the CYP119-catalyzed intermolecular C–H insertion reactions with EDA. Reference racemic samples were prepared with alternative engineered CYP119 variants as described in the following figures.

- Chiral SFC analysis of racemic **6a** (using CYP119 F153Y, T213A, V254A, C317S; *top*), enantioenriched **6a** product (using CYP119 F153G, T213A, V254A, C317S; *middle*) and **7a** product (using CYP119 A209W, T213G, V254A, C317S; *bottom*):

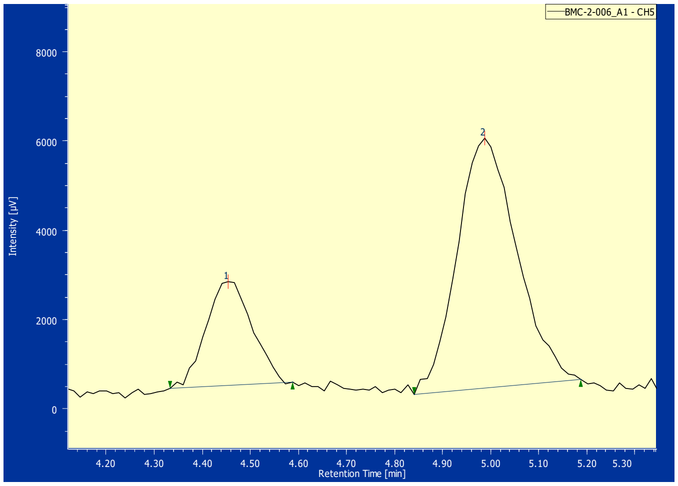

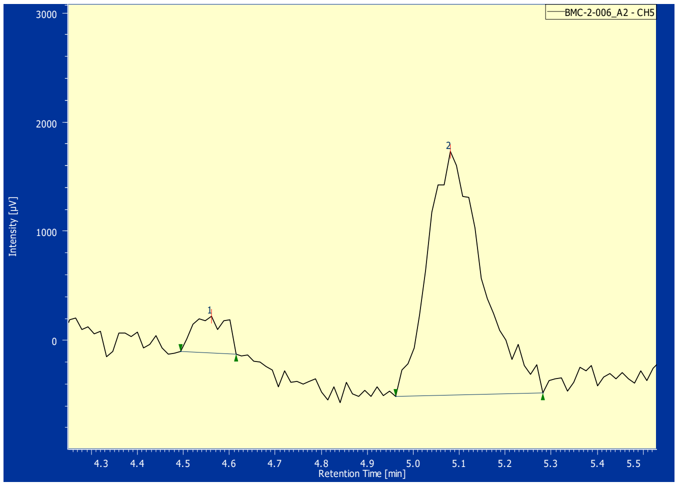

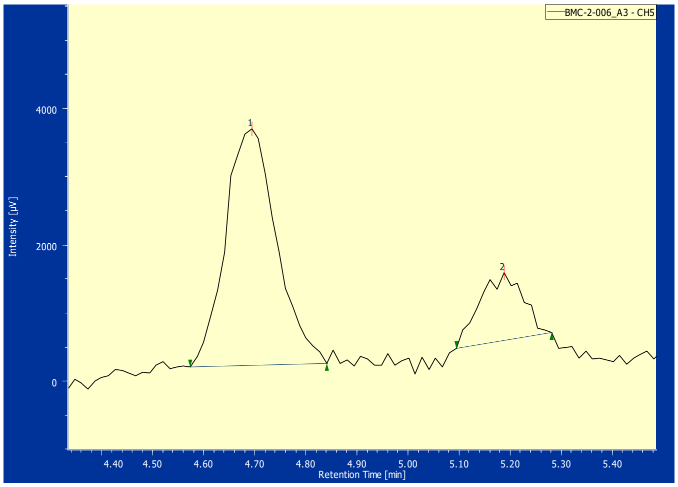

- Chiral SFC analysis of racemic **6b** (using CYP119 L205M, A209G, T213A, V254A, C317S; *top*), enantioenriched **6b** product (using CYP119 A209W, G210S, T213G, V254A, C317S; *middle*) and enantioenriched **7b** product (using CYP119 A209G, G210T, T213A, V254A, C317S; *bottom*):

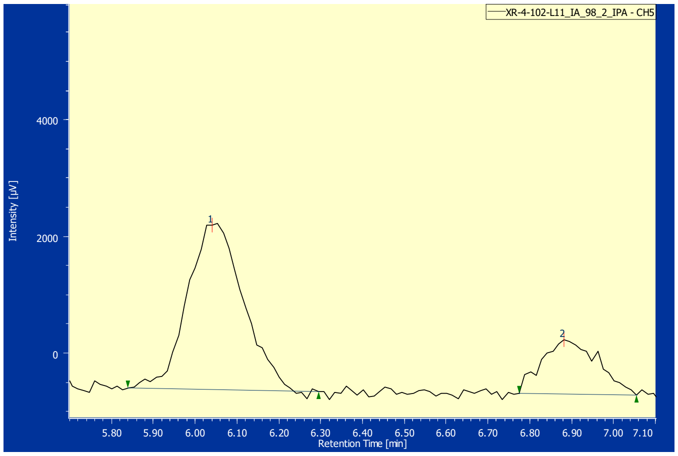

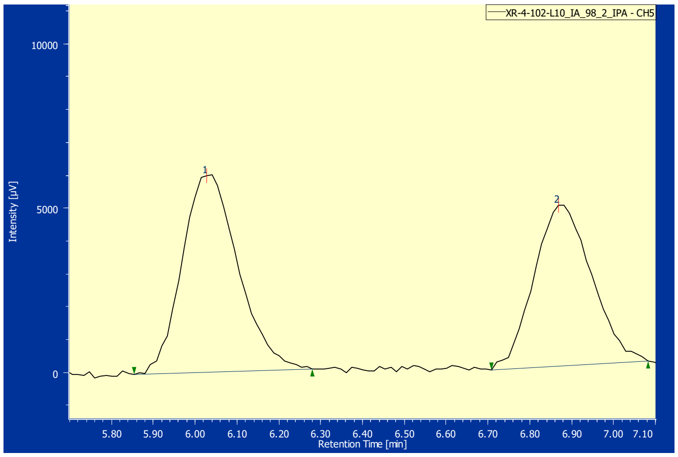

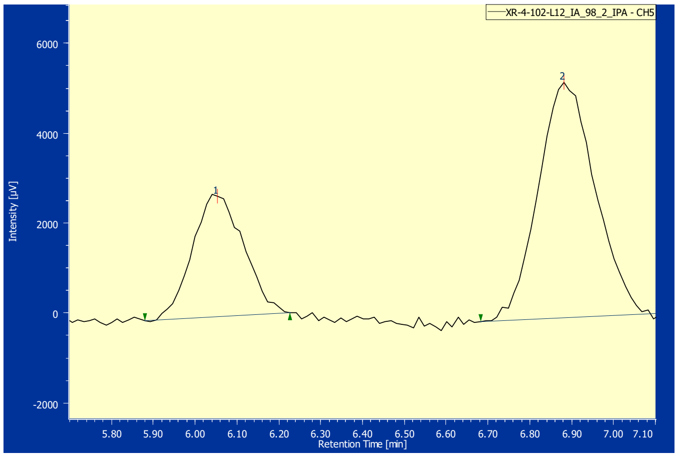

➢ Chiral SFC analysis of racemic **6c** (using CYP119 A209G, G210T, T213A, V254A, C317S; *top*) and enantioenriched **6c** product (using CYP119 A209W, G210S, T213G, V254A, C317S; *bottom*):

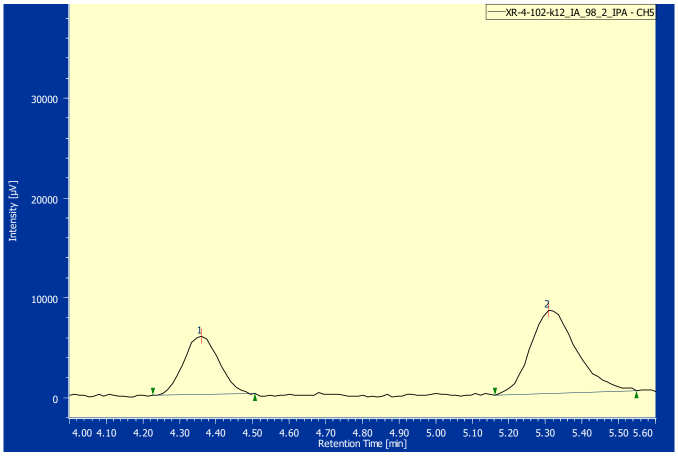

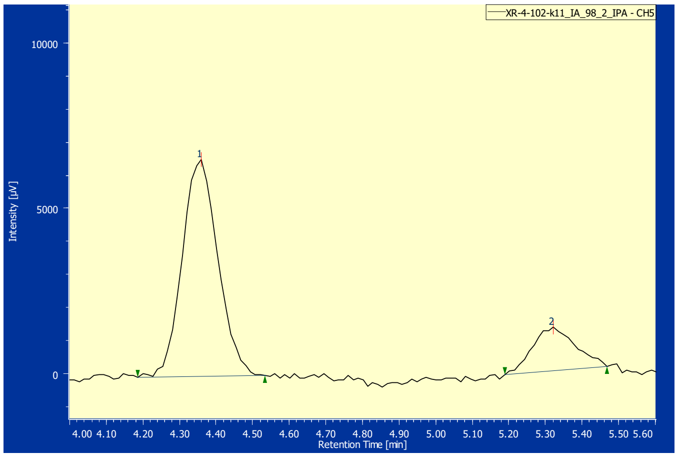

➢ Chiral SFC analysis of racemic **6d** (using CYP119 A209G, G210T, T213A, V254A, C317S; *top*) and enantioenriched **6d** product (using CYP119 F153P, A209G, T213A, V254A, C317S; *bottom*):

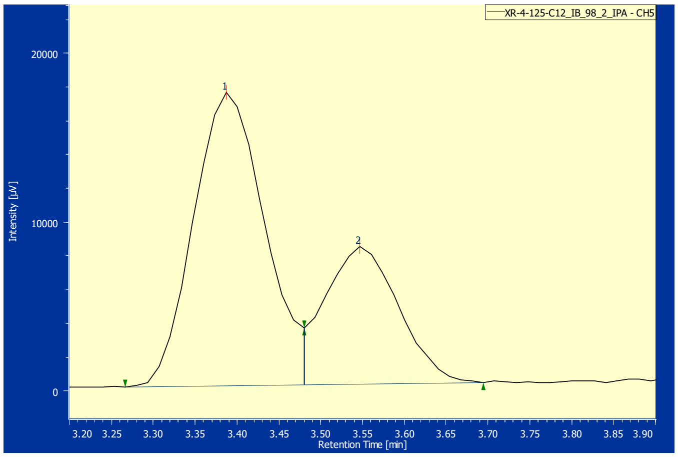

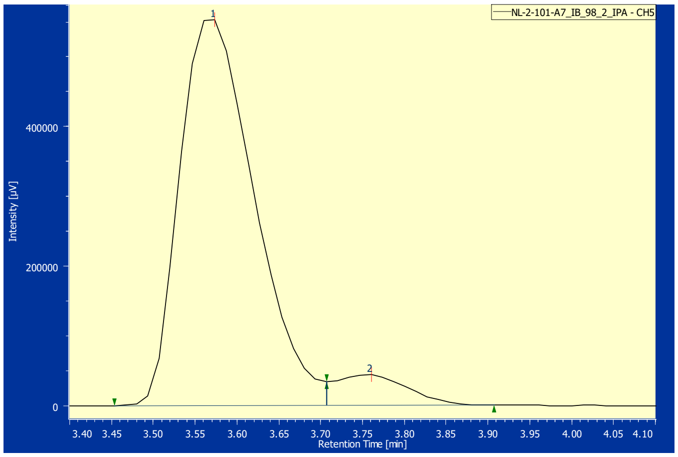

➢ Chiral SFC analysis of racemic **6e** (using CYP119 L205Y, A209G, T213A, V254A, C317S; *top*) and enantioenriched **6e** product (using CYP119 F153P, A209G, T213A, V254A, C317S; *bottom*):

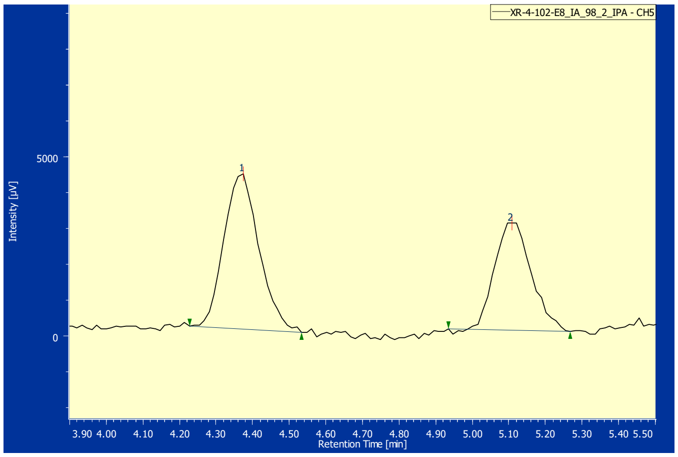

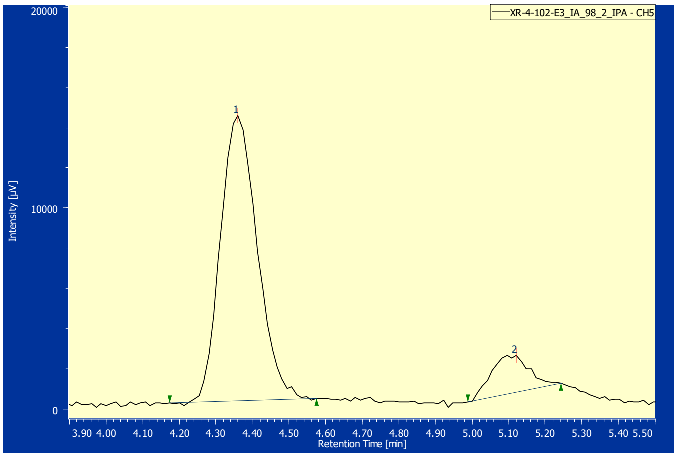

- Chiral HPLC analysis of racemic **6f** (using CYP119 F153Y, A209S, T213A, V254A, C317S; *top*), enantioenriched **6f** product (using CYP119 A209Y, T213G, V254A, C317S; *middle*) and enantioenriched **7f** product (using CYP119 F153G, A209S, T213A, V254A, C317S; *bottom*):

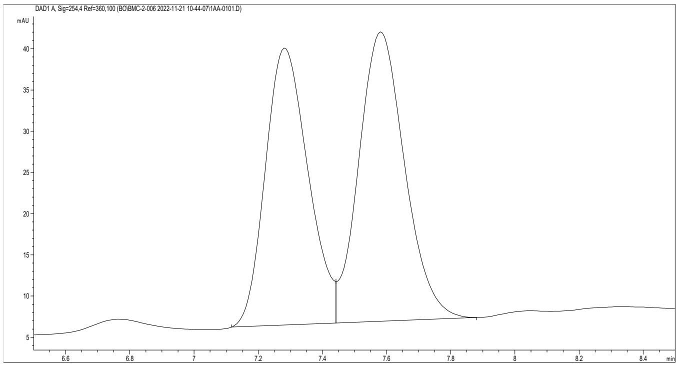

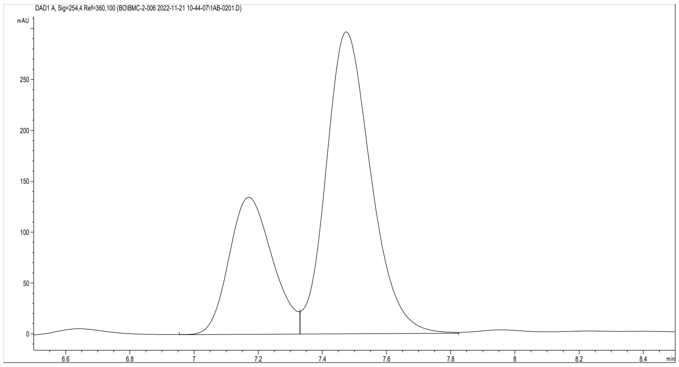

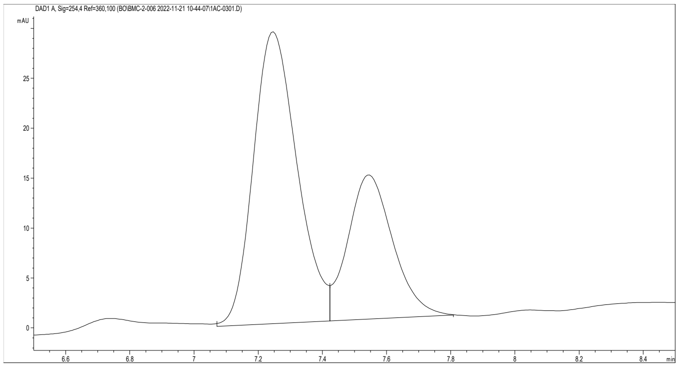

➢ Chiral SFC analysis of racemic **6g** (using CYP119 A209G, T213A, V254A, C317S; *top*) and enantioenriched **6g** product (using CYP119 A209W, T213G, V254W, C317S; *bottom*):

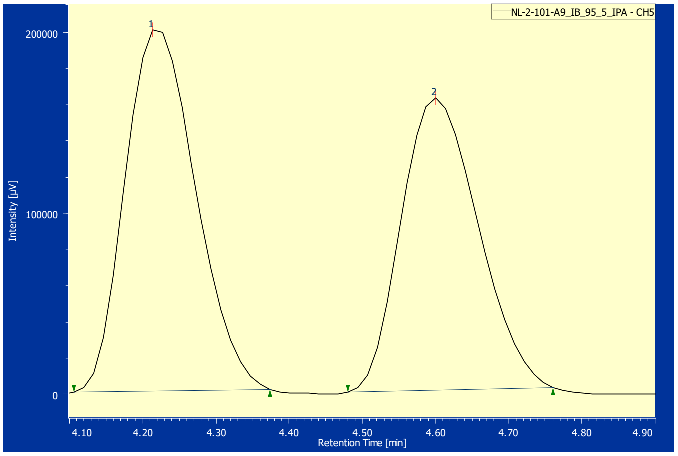

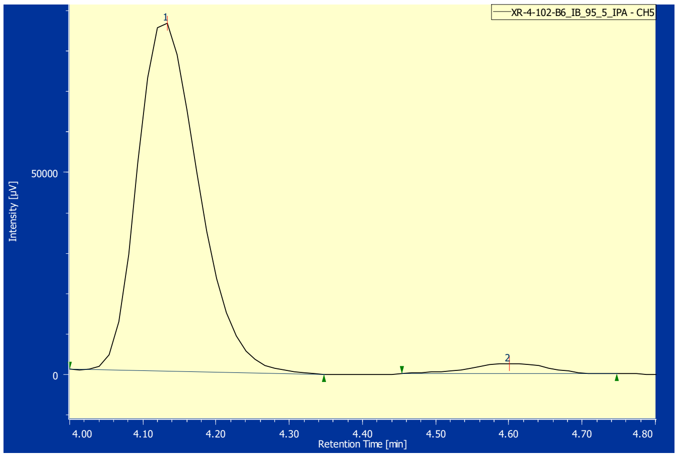

➢ Chiral SFC analysis of racemic **6h** (using CYP119 F153A, A209W, T213A, V254A, C317S; *top*) and enantioenriched **6h** product (using CYP119 L205M, A209G, T213A, V254A, C317S; *bottom*):

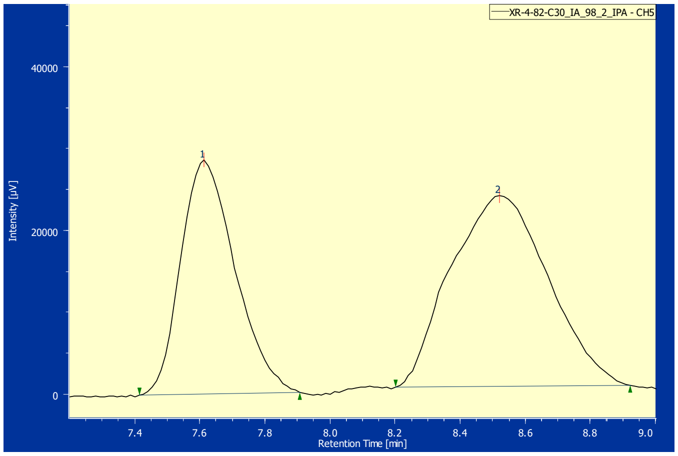

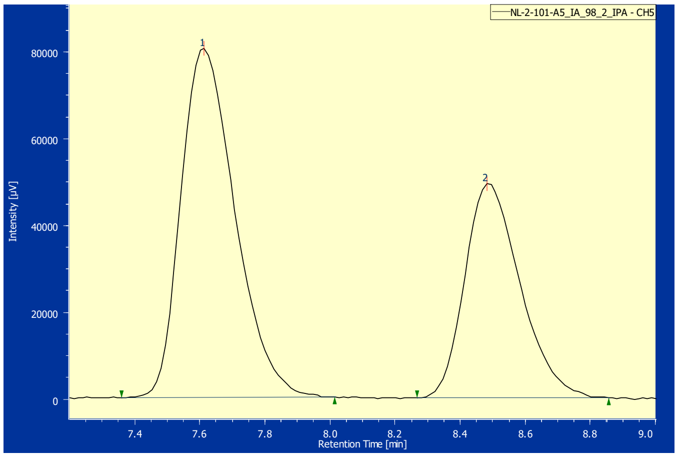

➢ Chiral SFC analysis of racemic **6i** product (using CYP119 F153P, A209G, T213A, V254A, C317S):

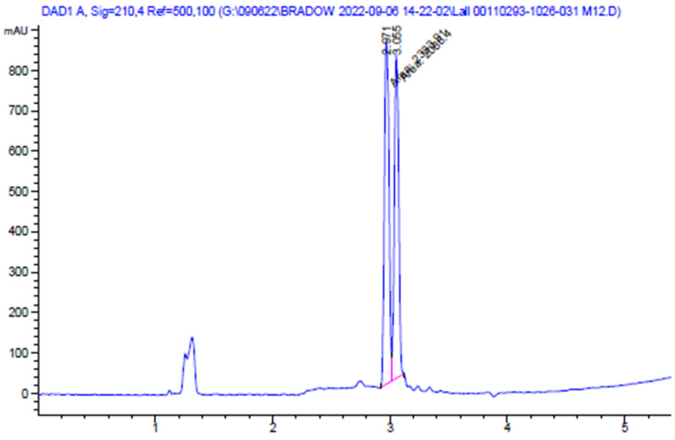

➢ Chiral SFC analysis of **6k** (using CYP119 L205T, A209G, T213A, V254W, C317S):

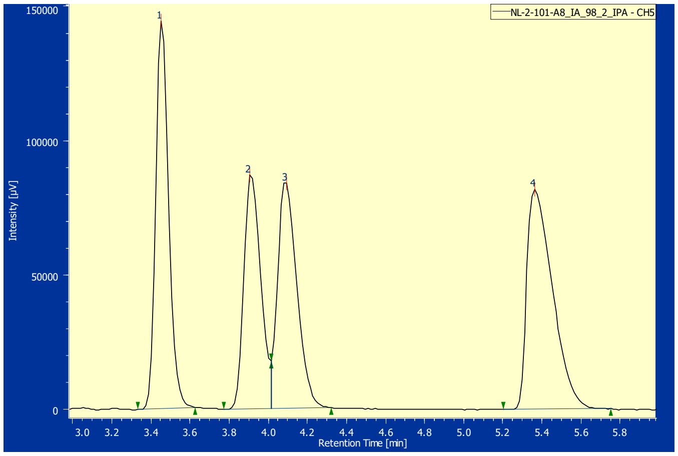

➢ Chiral SFC analysis of racemic **6m** (using CYP119 F153G, T213A, V254T, C317S; *top*), enantioenriched **6m** product (using CYP119 A209G, T213G, V254A, C317S; *middle*) and enantioenriched **7m** product (using CYP119 A209G, G210T, T213A, V254A, C317S; *bottom*):

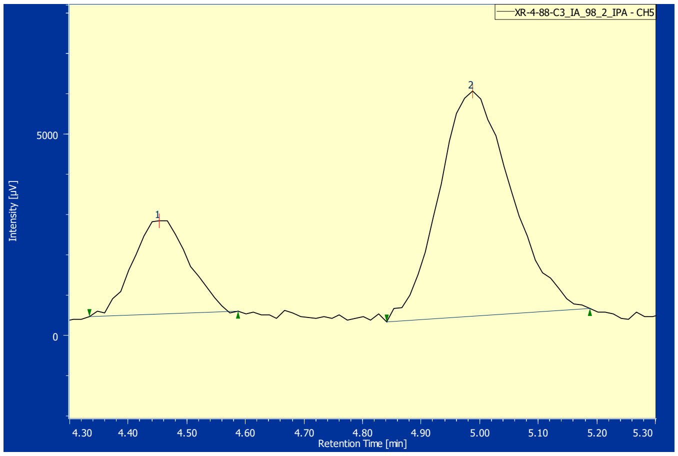

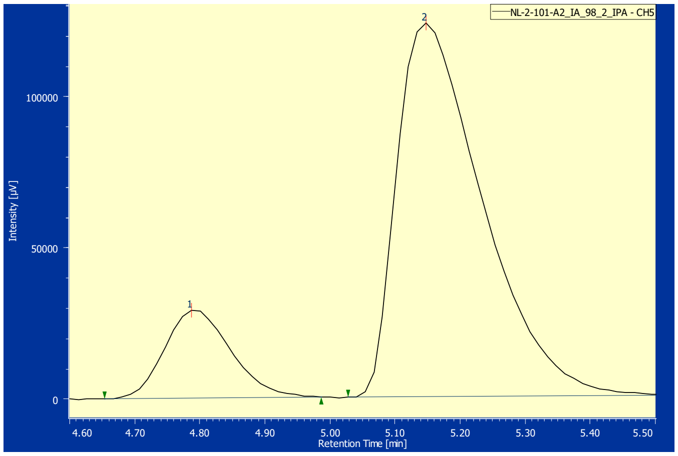

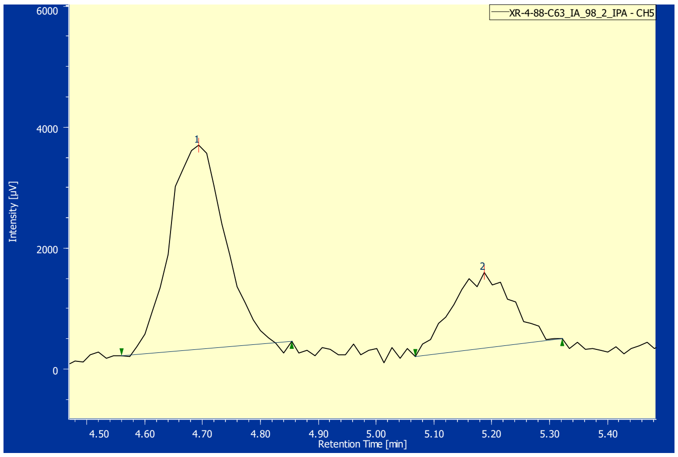

➢ Chiral HPLC analysis of enantioenriched **6n** product (using CYP119 A209W, G210S, T213G, V254A, C317S)

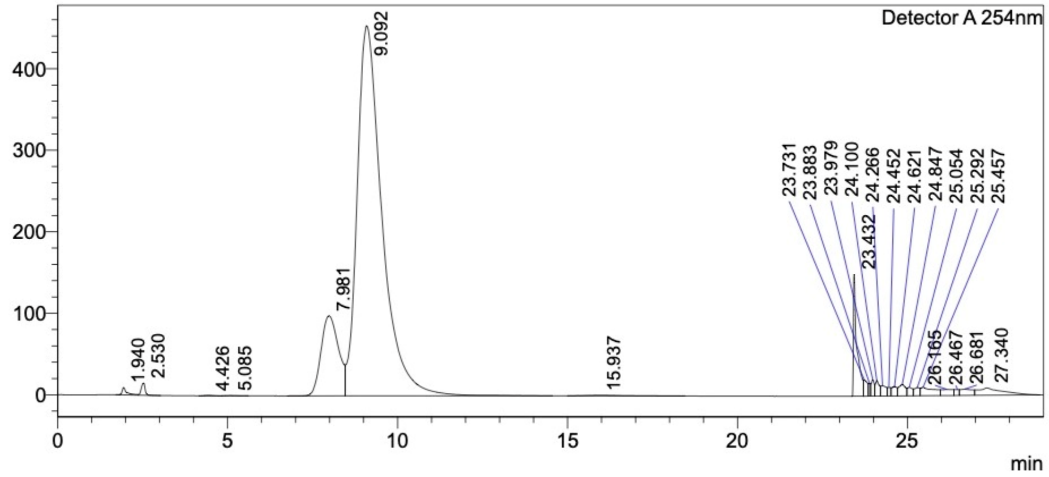

Figure S6. Chiral SFC analysis for the determination of diastereomeric and enantiomeric excess in the CYP119-catalyzed intermolecular dual C–H insertion reactions with EDA (**1b**). Reference racemic (or scalemic) samples were prepared with alternative engineered CYP119 variants as described in the following figures. Stereochemical assignment of the major enantiomer from the C-H insertion reaction with EDA was made by analogy with the corresponding reactions with diazoacetone.

- Chiral SFC analysis of **8a** using CYP119(F153G, T213A, V254A, C317S); referred to as CHI-EDA:

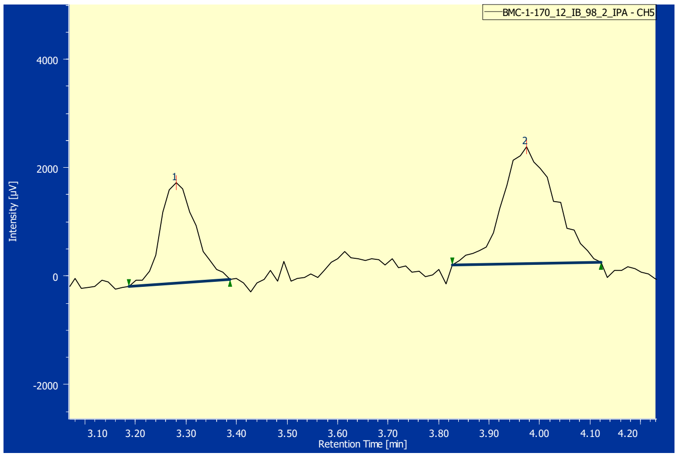

- Chiral SFC analysis of ***cis*(*S*,*R*)-8a** using CYP119(T213A, C317S); referred to as CHI-EDA$_{cis}$:

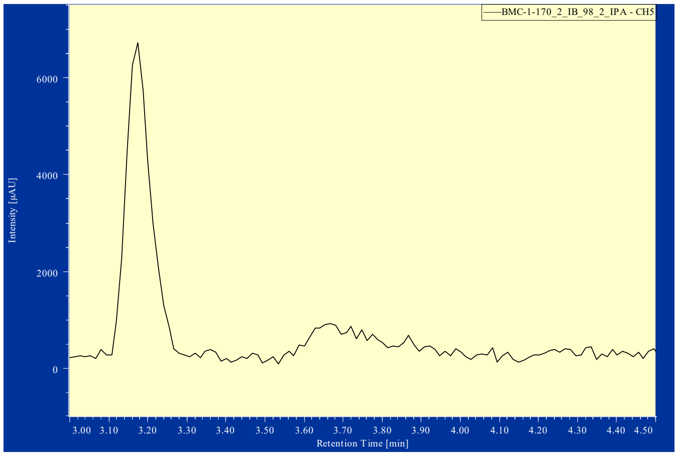

- ➢ Chiral SFC analysis of ***trans*(*S*,*S*)-8a** using CYP119(F153Y, T213G, V254A, C317S); referred to as CHI-EDA*trans-SS*:

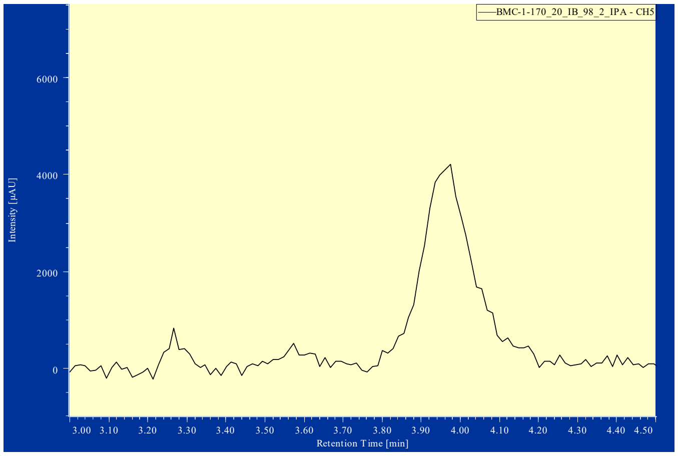

- ➢ Chiral SFC analysis of ***trans*(*R*,*R*)-8a** using CYP119(F153A, T213G, V254A, C317S); referred to as CHI-EDA*trans-RR*:

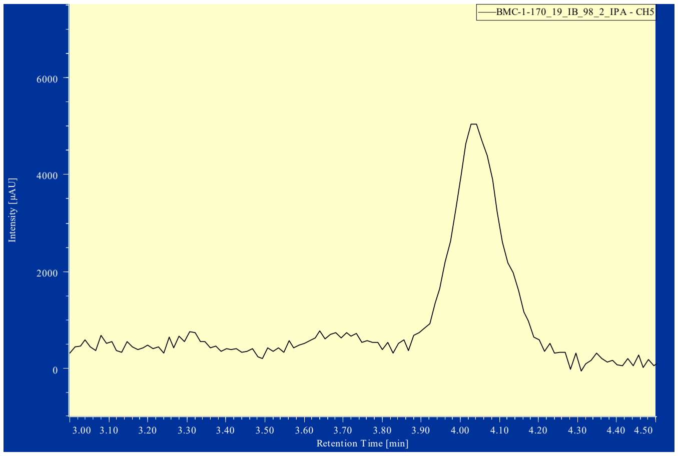

Figure S7. Chiral GC analysis for determination of enantiomeric excess in the CYP119-catalyzed intermolecular dual C–H insertion reactions with diazoacetone (**1a**) and EDA (**1b**). The reference racemic samples were prepared with engineered CYP119 variant as described in the following figures.

- Chiral GC analysis of enantioenriched **9a** using CYP119(L205T, A209G, T213A, V254A, C317S) (*top*), and enantioenriched **10a** product using CYP119(T213G, C317S) (*bottom*):

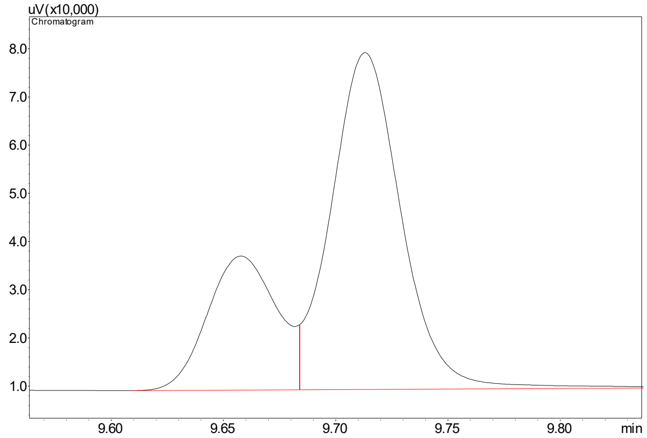

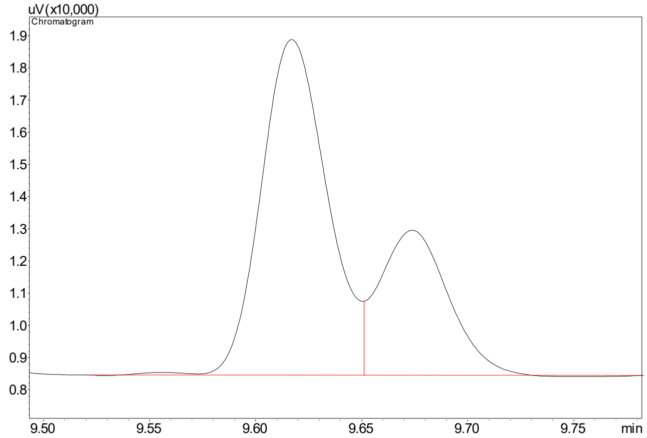

Figure S8. Chiral HPLC analysis for determination of enantiomeric excess in the CYP119-catalyzed late-stage C–H insertion of Sitagliptin core with EDA (**1b**).

- Chiral HPLC analysis of enantioenriched **12** prepared using CYP119(T213A, C317S):

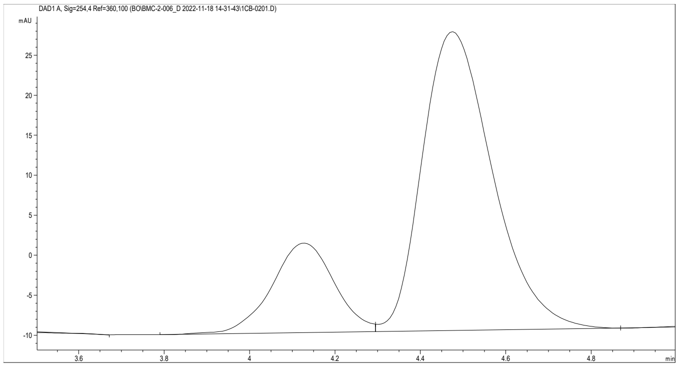

- Chiral HPLC analysis of enantioenriched **13** prepared using CYP119(T213A, C317S):

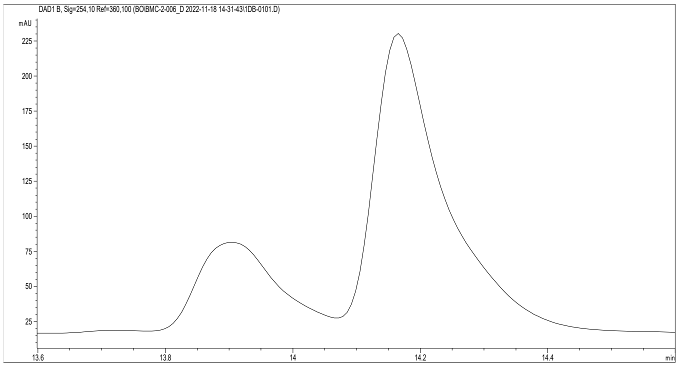

X-Ray Crystallographic Analyses

Data Collection. A crystal (0.533 x 0.197 x 0.12 mm3) was placed onto a thin glass optical fiber or a nylon loop and mounted on a Rigaku XtaLAB Synergy-S Dualflex diffractometer equipped with a HyPix-6000HE HPC area detector for data collection at 100.00(10) K. A preliminary set of cell constants and an orientation matrix were calculated from a small sampling of reflections. A short pre-experiment was run, from which an optimal data collection strategy was determined. The full data collection was carried out using a PhotonJet (Cu) X-ray source with frame times of 0.05 and 0.06 seconds and a detector distance of 34.0 mm. Series of frames were collected in 0.50º steps in ω at different 2θ, κ, and ϕ settings. After the intensity data were corrected for absorption, the final cell constants were calculated from the xyz centroids of 13955 strong reflections from the actual data collection after integration. See Table S7 for additional crystal and refinement information.

Structure solution and Refinement. The structure was solved using SHELXT and refined using SHELXL. The space group $P2_1$ was determined based on systematic absences and intensity statistics. Most or all non-hydrogen atoms were assigned from the solution. Full-matrix least squares / difference Fourier cycles were performed which located any remaining non-hydrogen atoms. All non-hydrogen atoms were refined with anisotropic displacement parameters. All hydrogen atoms were placed in ideal positions and refined as riding atoms with relative isotropic displacement parameters. The final full matrix least squares refinement converged to $R1 = 0.0368$ (F^2, $I > 2\sigma(I)$) and $wR^2 = 0.0999$ (F^2, all data).

Table S7. Crystal data and structure refinement for 3e. Cambridge Crystallographic Data Centre (CCDC) entry: 2210524

Identification code	fasnl01	
Empirical formula	C13 H16 F N O	
Formula weight	221.27	
Temperature	100.00(10) K	
Wavelength	1.54184 Å	
Crystal system	monoclinic	
Space group	$P2_1$	
Unit cell dimensions	a = 8.46090(10) Å	α = 90°
	b = 5.13850(10) Å	β = 91.3580(10)°
	c = 13.01250(10) Å	γ = 90°
Volume	565.577(14) $Å^3$	
Z	2	
Density (calculated)	1.299 Mg/m^3	
Absorption coefficient	0.762 mm^{-1}	
F(000)	236	
Crystal color, morphology	colourless, needle	
Crystal size	0.533 x 0.197 x 0.12 mm^3	
Theta range for data collection	3.397 to 79.913°	
Index ranges	$-10 \le h \le 10$, $-6 \le k \le 5$, $-16 \le l \le 16$	
Reflections collected	18283	
Independent reflections	2296 [R(int) = 0.0531]	
Observed reflections	2252	
Completeness to theta = 74.504°	100.0%	
Absorption correction	Multi-scan	
Max. and min. transmission	1.00000 and 0.38778	
Refinement method	Full-matrix least-squares on F^2	
Data / restraints / parameters	2296 / 1 / 146	
Goodness-of-fit on F^2	1.098	
Final R indices [I>2sigma(I)]	$R1$ = 0.0368, $wR2$ = 0.0995	
R indices (all data)	$R1$ = 0.0372, $wR2$ = 0.0999	
Absolute structure parameter	0.08(11)	
Largest diff. peak and hole	0.172 and -0.255 e.$Å^{-3}$	

Analytical Methods

Chiral SFC Methods. Stereoisomer resolution for compounds **3a-m**, **6a-e**, **6g-k**, **6m,** and **8a** was performed by Supercritical Fluid Chromatography (SFC) analysis, using a JASCO Analytical and Semi-Preparative SFC instrument equipped with a column oven (35 °C), photodiode array detector, a backpressure regulator (~14.0 MPa), a carbon dioxide pump and a sample injection volume of 3 µL. Daicel Chiralpak IA, IB, or AD-H column (0.46 cm ID × 25 cm L) were used for separation of enantiomers. All samples were eluted using an isocratic solvent system with the indicated modifier (see table below) in liquid CO_2 at an elution rate of 4 mL/min and detected at λ = 220 nm. Total run time was 10.2 min. Modifier solvent percentages and retention times (t_R) for chiral SFC analyses of samples **3a-m**, **6a-e**, **6g-k**, **6m,** and **8a** are reported in the tables below:

**Product**	**Column**	**Modifier Solvent**	**t_R for 1st isomer (min)**	**t_R for 2nd isomer (min)**
**3a**	B	5% IPA	3.5	4.0
**3b**	A	2% IPA	7.6	8.0
**3c**	A	2% IPA	5.6	6.2
**3d**	B	2% IPA	3.9	4.2
**3e**	A	2% IPA	5.6	6.2
**3f**	A	5% IPA	6.3	6.7
**3g**	B	10% IPA	3.0	4.8
**3h**	A	5% IPA	4.6	5.3
**3i**	A	5% IPA	4.8	5.2
**3j**	B	5% IPA	4.4	4.9
**3k**	A	2% IPA	5.1	6.6
**3l**	B	2% IPA	5.6	7.0
**3m**	B	2% IPA	6.1	6.9

Product	Column	Modifier Solvent	t_R for 1st isomer (min)	t_R for 2nd isomer (min)
**6a**	B	1% IPA	5.1	4.6
**6b**	A	2% IPA	6.0	6.9
**6c**	A	2% IPA	4.3	5.3
**6d**	B	2% IPA	3.4	3.5
**6e**	A	2% IPA	4.2	5.1
**6g**	B	5% IPA	4.2	4.6
**6h**	A	2% IPA	7.6	8.5
**6i**	AD-H	7.5% MeOH	3.0	3.1
**6k**	A	2% IPA	3.4	3.9
**6m**	A	2% IPA	4.7	5.2

Product	Column	Modifier Solvent	t_R for 1st isomer (min)	t_R for 2nd isomer (min)	t_R for 3rd isomer (min)
**8a**	B	2% IPA	3.2	3.9	4.1

Chiral HPLC Methods. Stereoisomer resolution for compound **6f**, **6n**, **12**, and **13** was performed by High Performance Liquid Chromatography (HPLC) analysis, using a Shimadzu HPLC-2030 iSeries Plus, equipped with column oven (25 °C), UV/PDA detector and a sample injection volume of 5 µL. Daicel Chiralpak IB, IF or AD-H column (0.46 cm ID × 15 cm L) was used for separation of enantiomers. All samples were eluted using an isocratic solvent system with the indicated modifier (see table below) in hexanes at an elution rate of 1 mL/min and detected at 254 nm. Total run time was 20 min.

Product	Column	Modifier Solvent	t_R for 1st isomer (min)	t_R for 2nd isomer (min)
**6f**	IF	4% IPA	7.2	7.6
**6n**	AD-H	-	8.0	9.1
**12**	IB	5% IPA	4.1	4.5

13	IB	5% IPA	13.9	14.2

GC Methods. Gas chromatography (GC) analysis were carried out using a Shimadzu GC-2010 gas chromatograph equipped with a FID detector, and a Cyclosil-B column (30 m x 0.25 mm x 0.25 μm film). The following GC methods were used for TON analysis: 1 μL injection, injector temp.: 200 ºC, detector temp: 300 ºC. Method: column temperature set at 80 ºC for 2 min, then to 245 ºC at 40 ºC/min, then held at 245 ºC for 6.5 min. Total run time was 12.63 min.

Additional Synthetic Procedures

Synthesis of diazoacetone (1a)

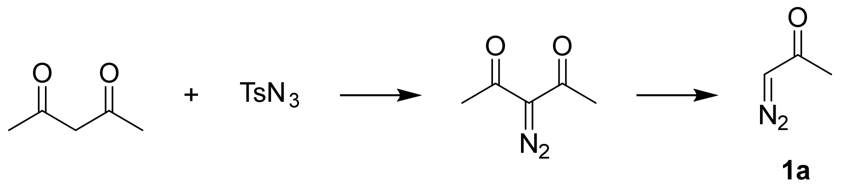

1a

Diazoacetone (**1a**) was prepared in accordance to a reported procedure (Abid *et al.*, *J. Org. Chem.* **2015**, 80, 9980-9988). Isolated yield: 81%. NMR data matched the reported data.

Synthesis of substituted N-phenylpyrrolidine derivatives

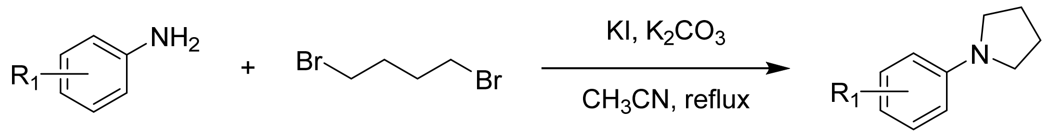

2b-d

Substrates **2b-2d** were synthesized according to a reported procedure (Rao *et al., Synlett*, **2015**, 26(16), 2231-2236). To a stirring solution of K_2CO_3 (5.5g, 40 mmol), KI (0.17g, 5 mol%) in MeCN (20 mL) under nitrogen, 1,4-dibromobutane (20 mmol) and aryl amines (25 mmol) were added. The reaction mixture was heated at reflux overnight. After removing the solvent by rotary evaporation, ethyl acetate (40 mL) and water (15 mL) was added. The organic layer was separated, and the aqueous layer was extracted by ethyl acetate (3 x 20 mL). The combined organic layer was washed with brine, dried over $MgSO_4$, filtered, and purified in silica column using hexane as

eluent. Products are colorless to light yellow oil. Isolated yield: 76-88%. NMR data matched the reported data.

Synthesis of MOM protected Januvia core (11a)

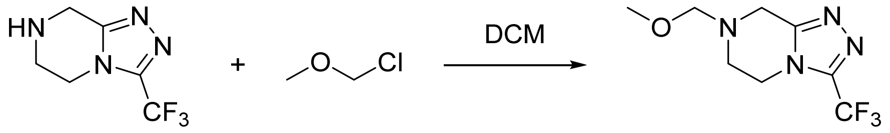

To a stirred solution of 3-(trifluoromethyl)-5,6,7,8-tetrahydro-[1,2,4]triazolo[4,3-*a*]pyrazine (200 mg, 1.04 mmol) in DCM (5 mL) cooled to 0°C, chloro(methoxy)methane (316 μL, 4.16 mmol, 4 equiv.) was added dropwise over 5 minutes. The reaction was allowed to stir at 0°C for 20 minutes before the solid was filtered and washed will cooled DCM. The product was isolated as a light yellow solid (isolated yield: 92% (**11a)**).

Compound characterization

(*S*)-1-(1-phenylpyrrolidin-2-yl)propan-2-one (3a)

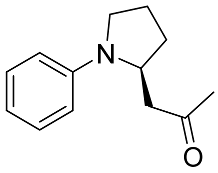

(*S*)-1-(1-phenylpyrrolidin-2-yl)propan-2-one was prepared according to the general procedure A. GC-MS m/z (% relative intensity): 203(9.6), 147(11.2), 146(100), 104(8.7), 77(12.0); 1H NMR (400 MHz, $CDCl_3$) δ 7.22 (dd, J = 8.0, 7.2 Hz, 2H), 6.68 (t, J = 7.2 Hz, 1H), 6.55 (d, J = 8.0 Hz, 2H), 4.23 (dd, J = 9.5, 7.5 Hz, 1H), 3.42 (dt, J = 10.1, 7.2 Hz, 1H), 3.24 – 3.12 (m, 1H), 2.89 (dd, J = 17.1, 2.5 Hz, 1H), 2.44 (dd, J = 17.0, 10.2 Hz, 1H), 2.21 – 2.14 (m, 3H), 2.14 – 2.04 (m, 1H), 2.01 – 1.95 (m, 1H), 1.76 (dd, J = 16.3, 3.9 Hz, 1H), 1.62 – 1.56 (m, 1H). 13C NMR (101 MHz, $CDCl_3$) δ 208.4, 146.6, 129.5, 115.9, 112.0, 54.1, 48.0, 46.6, 31.5, 31.1, 23.2.

(*S*)-1-(1-(p-tolyl)pyrrolidin-2-yl)propan-2-one (3b)

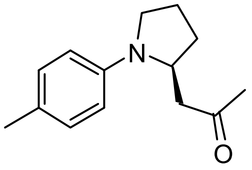

(*S*)-1-(1-(p-tolyl)pyrrolidin-2-yl)propan-2-one was prepared according to the general procedure A. GC-MS m/z (% relative intensity): 217(12.4), 161(12.6), 160(100), 158(6.2), 118(13.1), 91(27.0), 65(9.3); ^1H NMR (500 MHz, $CDCl_3$) δ 7.04 (d, *J* = 8.2 Hz, 2H), 6.48 (d, *J* = 8.2 Hz, 2H), 4.18 (t, *J* = 8.7 Hz, 1H), 3.39 (t, *J* = 9.6 Hz, 1H), 3.15 (q, *J* = 8.2 Hz, 1H), 2.86 (t, *J* = 15.7 Hz, 1H), 2.42 (dd, *J* = 16.8, 10.1 Hz, 1H), 2.25 (s, 3H), 2.16 (s, 3H), 2.13 – 2.03 (m, 2H), 2.00 (d, *J* = 12.3 Hz, 2H), 1.77 – 1.68 (m, 1H). ^{13}C NMR (126 MHz, $CDCl_3$) δ 130.0, 112.1, 54.3, 48.2, 46.7, 31.7, 31.5, 23.3, 22.8. Quaternary carbons not observed.

(*S*)-1-(1-(m-tolyl)pyrrolidin-2-yl)propan-2-one (3c)

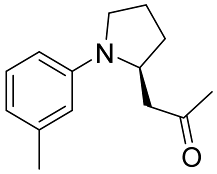

(*S*)-1-(1-(m-tolyl)pyrrolidin-2-yl)propan-2-one was prepared according to the general procedure A. GC-MS m/z (% relative intensity): 217(12.2), 161(12.2), 160(100), 158(5.9), 118(11.0), 117(13.6), 91(30.2), 65(9.9); 1H NMR (500 MHz, $CDCl_3$) δ 7.10 (dd, J = 10.1, 6.3 Hz, 1H), 6.49 (d, J = 7.4 Hz, 1H), 6.35 (d, J = 5.0 Hz, 2H), 4.23 – 4.16 (m, 1H), 3.38 (td, J = 8.0, 3.8 Hz, 1H), 3.15 (q, J = 8.5 Hz, 1H), 2.87 (dd, J = 17.2, 2.6 Hz, 1H), 2.41 (dd, J = 16.9, 10.2 Hz, 1H), 2.29 (s, 3H), 2.16 (s, 3H), 2.13 – 1.92 (m, 3H), 1.73 (d, J = 11.9 Hz, 1H). 13C NMR (126 MHz, $CDCl_3$) δ 209.1, 139.9, 130.0, 121.5, 117.5, 113.3, 109.8, 54.7, 48.6, 47.2, 32.1, 31.7, 23.8, 22.6.

(*S*)-1-(1-(o-tolyl)pyrrolidin-2-yl)propan-2-one (3d)

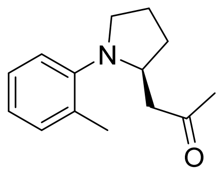

(*S*)-1-(1-(o-tolyl)pyrrolidin-2-yl)propan-2-one was prepared according to the general procedure A. GC-MS m/z (% relative intensity): 217(7.1), 176(12.7), 161(10.5), 160(100), 158(10.4), 132(8.8), 131(8.1), 130(8.7), 120 (6.0), 118(32.5), 117(13.6), 91(36.2), 89(5.8), 77(6.8), 65(16.2); ^{1}H NMR (400 MHz, $CDCl_3$) δ 7.2 – 7.1 (m, 2H), 7.0 – 6.9 (m, 2H), 4.1 – 3.9 (m, 1H), 3.5 (dt, *J* = 9.0, 7.2 Hz, 1H), 2.8 (td, *J* = 8.7, 4.7 Hz, 1H), 2.7 (dd, *J* = 16.6, 3.5 Hz, 1H), 2.4 – 2.3 (m, 2H), 2.3 (s, 3H), 2.0 (s, 3H), 2.0 – 1.8 (m, 2H), 1.5 (dq, *J* = 12.3, 8.6 Hz, 1H). ^{13}C NMR (126 MHz, $CDCl_3$) δ 208.6, 147.9, 132.6, 131.5, 126.5, 122.2, 118.7, 55.9, 53.0, 47.9, 31.8, 31.0, 23.8, 19.5.

(*S*)-1-(1-(4-fluorophenyl)pyrrolidin-2-yl)propan-2-one (3e)

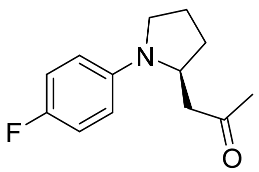

(*S*)-1-(1-(4-fluorophenyl)pyrrolidin-2-yl)propan-2-one was prepared according to the general procedure A. GC-MS m/z (% relative intensity): 221(11.2), 165(10.6), 164(100), 162(6.4), 122(29.4), 109(8.1), 95(31.2), 75(9.0); ^{1}H NMR (400 MHz, $CDCl_3$) δ 6.94 (dd, *J* = 8.8, 8.8 Hz, 2H), 6.50 – 6.40 (m, 2H), 4.16 (dddd, *J* = 9.7, 7.7 Hz, 1H), 3.37 (ddd, *J* = 12.0, 7.5 Hz, 1H), 3.13 (dd, *J* = 16.4, 8.3 Hz, 1H), 2.83 (dd, *J* = 17.0, 2.2 Hz, 1H), 2.44 (dd, *J* = 17.0, 10.1 Hz, 1H), 2.17 (s, 3H), 2.15 – 2.06 (m, 1H), 2.06 – 1.96 (m, 2H), 1.79 – 1.70 (m, 1H). ^{13}C NMR (101 MHz, $CDCl_3$) δ 208.3, 143.3, 115.9 (d, *J* = 23.7 Hz), 112.5, 54.6, 48.5, 46.6, 31.4, 31.2, 23.3. F-Carbon not observed ^{19}F NMR (376 MHz, $CDCl_3$) δ -130.5.

(*S*)-1-(1-(4-chlorophenyl)pyrrolidin-2-yl)propan-2-one (3f)

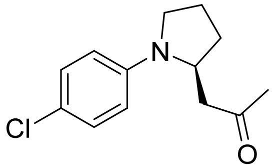

(*S*)-1-(1-(4-chlorophenyl)pyrrolidin-2-yl)propan-2-one was prepared according to the general procedure A. GC-MS m/z (% relative intensity): 237(10.8), 182(31.8), 181(11.6), 180(100), 140(8.9), 138(18.1), 117(7.1), 113(6.4), 111(24.7), 75(11.9); ^{1}H NMR (400 MHz, $CDCl_3$) δ 7.2 – 7.1 (m, 2H), 6.5 – 6.4 (m, 2H), 4.2 (ddt, *J* = 9.9, 7.6, 2.1 Hz, 1H), 3.4 – 3.3 (m, 1H), 3.1 (td, *J* = 8.9, 7.4 Hz, 1H), 2.8 (dd, *J* = 17.2, 2.6 Hz, 1H), 2.4 (dd, *J* = 17.1, 10.1 Hz, 1H), 2.2 (s, 3H), 2.1 – 1.9 (m, 3H), 1.8 – 1.7 (m, 1H). ^{13}C NMR (101 MHz, $CDCl_3$) δ 207.9, 145.0, 129.1, 120.5, 112.8, 79.5, 77.3, 77.0, 76.7, 54.1, 47.9, 46.1, 31.4, 30.9, 23.1.

(*S*)-1-(1-(4-methoxyphenyl)pyrrolidin-2-yl)propan-2-one (3g)

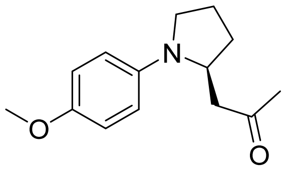

(*S*)-1-(1-(4-methoxyphenyl)pyrrolidin-2-yl)propan-2-one was prepared according to the general procedure A. GC-MS m/z (% relative intensity): 233(16.3), 177(12.3), 176(100), 161(6.5), 134(11.0), 132(5.3), 120(7.0), *77(9.9); ^{1}H NMR (500 MHz, $CDCl_3$) δ 6.86 – 6.74 (m, 2H), 6.54 – 6.47* (m, 2H), 4.13 (dd, *J* = 9.7, 7.7 Hz, 1H), 3.73 (s, 3H), 3.39 – 3.32 (m, 1H), 3.11 (q, *J* = 8.2 Hz, 1H), 2.83 (dd, *J* = 16.7, 2.1 Hz, 1H), 2.41 (dd, *J* = 16.8, 10.0 Hz, 1H), 2.14 (s, 3H), 2.12 – 1.92 (m, 3H), 1.75 – 1.67 (m, 1H). ^{13}C NMR (126 MHz, $CDCl_3$) δ 208.52, 151.13, 141.61, 115.33, 113.01, 56.09, 54.65, 48.66, 46.86, 31.59, 31.12, 23.35.

(*S*)-1-(1-(3-bromophenyl)pyrrolidin-2-yl)propan-2-one (3h)

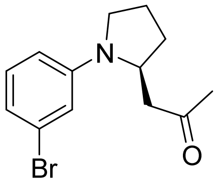

(*S*)-1-(1-(3-bromophenyl)pyrrolidin-2-yl)propan-2-one was prepared according to the general procedure A. GC-MS m/z (% relative intensity): 283(12.9), 281(14.3), 227(10.1), 226(93.1), 225(11.6), 224(100), 184(10.5), 182(8.9), 157(9.5), 155(8.5), 145(8.3), 144(5.7), 130(5.9), 117(6.4), 76(6.3); ^{1}H NMR (500 MHz, $CDCl_3$) δ 7.03 (dd, *J* = 8.1, 8.1 Hz, 1H), 6.76 (d, *J* = 7.6 Hz, 1H), 6.64 (s, 1H), 6.43 (d, *J* = 7.8 Hz, 1H), 4.22 – 4.13 (m, 1H), 3.41 – 3.30 (m, 1H), 3.13 (dd, *J* = 16.6, 8.9 Hz, 1H), 2.81 (dd, *J* = 17.4 Hz, 1H), 2.43 (dd, *J* = 17.2, 10.4 Hz, 1H), 2.16 (s, 3H), 2.14 – 2.04 (m, 1H), 2.03 – 1.95 (m, 2H), 1.78 – 1.71 (m, 1H). ^{13}C NMR (126 MHz, $CDCl_3$) δ 207.94, 147.72, 130.68, 123.73, 118.75, 114.76, 110.66, 54.19, 48.07, 46.21, 31.49, 31.08, 23.13.

(*S*)-2-(2-(2-oxopropyl)pyrrolidin-1-yl)benzonitrile (3i)

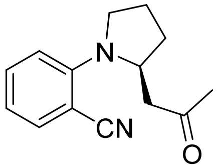

(*S*)-2-(2-(2-oxopropyl)pyrrolidin-1-yl)benzonitrile was prepared according to the general procedure A. GC-MS m/z (% relative intensity): 228(9.6), 185(5.9), 172(15.4), 171(100), 154(14.8), 129(16.8), 102(11.3); ^{1}H NMR (500 MHz, $CDCl_3$) δ 7.45 (dd, *J* = 7.9, 1.8 Hz, 1H), 7.32 (ddd, *J* = 8.9, 7.3, 1.7 Hz, 1H), 6.69 (t, *J* = 7.5 Hz, 1H), 6.65 (d, *J* = 8.7 Hz, 1H), 4.50 – 4.41 (m, 1H), 3.90 (dt, *J* = 9.8, 7.1 Hz, 1H), 3.46 (ddd, *J* = 9.8, 7.7, 5.3 Hz, 1H), 2.93 (dd, *J* = 17.3, 2.7 Hz, 1H), 2.44 (dd, *J* = 17.3, 9.9 Hz, 1H), 2.26 (dq, *J* = 13.5, 6.8 Hz, 1H), 2.16 (s, 3H), 2.01 – 1.79 (m, 2H), 1.69 (ddd, *J* = 10.7,

7.1, 5.5 Hz, 1H). ^{13}C NMR (126 MHz, $CDCl_3$) δ 208.07, 150.21, 136.61, 134.25, 121.62, 117.78, 115.83, 97.61, 55.59, 52.26, 47.68, 32.70, 32.32. 24.76

(*S*)-1-(1-(pyridin-2-yl)pyrrolidin-2-yl)propan-2-one (3j)

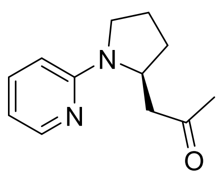

(*S*)-1-(1-(pyridin-2-yl)pyrrolidin-2-yl)propan-2-one was prepared according to the general procedure A. GC-MS m/z (% relative intensity): 204(15.1), 162(5.9), 161(52.3), 148(9.7), 147(100), 145(9.8), 133(10.1), 132(12.0), 131(9.3), 120(7.1), 119(25.7), 107(17.2), 79(20.9), 78(65.7), 52(10.2), 51(14.0); ^{1}H NMR (500 MHz, $CDCl_3$) δ 8.10 (dd, *J* = 5.2, 1.9 Hz, 1H), 7.38 (ddd, *J* = 8.9, 7.0, 2.0 Hz, 1H), 6.49 (dd, *J* = 7.1, 4.9 Hz, 1H), 6.30 (d, *J* = 8.5 Hz, 1H), 4.45 (ddd, *J* = 9.9, 6.5, 3.7 Hz, 1H), 3.50 – 3.42 (m, 1H), 3.27 (q, *J* = 8.5 Hz, 1H), 3.06 (dd, *J* = 16.0, 3.3 Hz, 1H), 2.40 (dd, *J* = 16.1, 9.5 Hz, 1H), 2.15 (s, 3H), 2.15 – 2.05 (m, 1H), 2.08 – 1.93 (m, 2H), 1.81 – 1.72 (m, 1H). ^{13}C NMR (126 MHz, $CDCl_3$) δ 208.48, 156.78, 148.31, 137.10, 111.64, 106.68, 53.84, 47.37, 31.19, 30.75, 23.43.

1-((2*S*)-5-methyl-1-phenylpyrrolidin-2-yl)propan-2-one (3k)

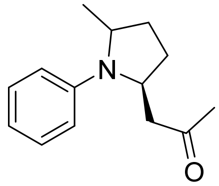

1-((2*S*)-5-methyl-1-phenylpyrrolidin-2-yl)propan-2-one was prepared according to the general procedure A. GC-MS m/z (% relative intensity): 217(17.0), 202(8.5), 161(12.9), 160(100), 145(5.7), 144(24.3), 130(7.5), 119(12.1), 118(23.4), 117(9.0), 104(25.0), 91(6.5), 77(46.1); ^{1}H NMR (500 MHz, $CDCl_3$) δ 7.24 – 7.18 (m, 2H), 6.68 (t, *J* = 7.3 Hz, 1H), 6.59 (d, *J* = 8.1 Hz, 2H), 4.33 (ddd, *J* = 9.9, 7.4, 2.3 Hz, 1H), 3.99 (p, *J* = 6.3 Hz, 1H), 3.03 (dd, *J* = 16.9, 3.0 Hz, 1H), 2.48 (dd, *J* = 16.9, 10.0 Hz, 1H), 2.34 – 2.22 (m, 1H), 2.17 (s, 3H), 1.77 – 1.66 (m, 3H), 1.26 (d, *J* = 6.1 Hz, 3H). ^{13}C NMR (101 MHz, $CDCl_3$) δ 208.5, 208.1, 146.6, 144.5, 129.3, 129.2, 115.9, 115.3, 113.3, 112.0, 77.3, 77.0, 76.7, 56.1, 55.7, 53.2, 52.5, 49.1, 45.1, 32.1, 31.0, 30.8, 30.6, 30.2, 28.7, 21.6, 18.2.

1-(1,3-dihydroisobenzofuran-1-yl)propan-2-one (3l)

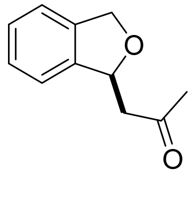

1-(1,3-dihydroisobenzofuran-1-yl)propan-2-one was prepared according to the general procedure A. GC-MS m/z (% relative intensity): 176(4.8), 161(31), 133(12.5), 119(90.4), 118(100), 103(14.6), 91(90.7), 90(31.7), 89(20.8),

77(24.5), 65(32.1), 63(19), 51(16.4). ^{1}H NMR (500 MHz, $CDCl_3$) δ 7.3 – 7.2 (m, 2H), 7.2 – 7.1 (m, 2H), 5.7 (s, 1H), 5.1 (dd, *J* = 12.4, 2.6 Hz, 1H), 5.0 (d, *J* = 12.2 Hz, 1H), 2.9 – 2.8 (m, 2H), 2.2 (s, 3H). ^{13}C NMR (126 MHz, $CDCl_3$) δ 207.42, 141.81, 139.85, 128.22, 121.91, 121.80, 80.61, 73.36, 51.06, 31.65.

(*S*)-1-(1-phenylpiperidin-2-yl)propan-2-one (3m)

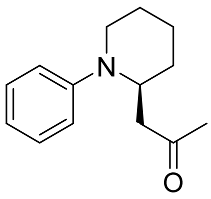

(*S*)-1-(1-phenylpiperidin-2-yl)propan-2-one was prepared according to the general procedure A. GC-MS m/z (% relative intensity): 218(12.3), 217(59.8), 174(7.6), 161(63.0), 160(100), 159(11.9), 158(18.1), 144(8.7), 133(7.6), 132(57.2), 130(21.9), 119(13.9), 118(27.0), 117(12.2), 106(16.7), 105(11.3), 104(45.7), 91(13.8), 78(8.5), 77(57.6). ^{1}H NMR (500 MHz, $CDCl_3$) δ 7.2 (dd, *J* = 8.8, 7.2 Hz, 3H), 6.9 (d, *J* = 8.1 Hz, 2H), 6.8 (t, *J* = 7.4 Hz, 1H), 4.3 (dd, *J* = 9.3, 4.5 Hz, 1H), 3.3 – 3.3 (m, 1H), 2.9 – 2.8 (m, 1H), 2.7 (dd, *J* = 16.3, 9.6 Hz, 1H), 2.4 (dd, *J* = 16.2, 3.7 Hz, 1H), 2.0 (s, 3H), 1.9 – 1.8 (m, 1H), 1.7 (d, *J* = 12.3 Hz, 1H), 1.7 – 1.6 (m, 4H). ^{13}C NMR (126 MHz, $CDCl_3$) δ 208.8, 151.2, 130.0, 129.9, 129.3, 120.2, 117.8, 115.7, 78.0, 77.8, 77.5, 52.8, 51.5, 45.6, 45.5, 42.3, 31.4, 29.6, 26.3, 25.9, 23.2, 20.1.

1,1'-(1-phenylpyrrolidine-2,5-diyl)bis(propan-2-one) (5a)

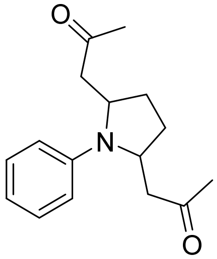

1,1'-(1-phenylpyrrolidine-2,5-diyl)bis(propan-2-one) was prepared according to general procedure B. GC-MS m/z (% relative intensity): 259(10.6), 203(7.4), 202(50.7), 158(14.2), 145(11.8), 144(100), 120(5.2), 119(5.2), 118(13.2), 117(6.7), 104(10.0), 91(6.7), 77(25.5), 51(5.9). ^{1}H NMR (500 MHz, $CDCl_3$) δ 7.21 (t, *J* = 7.2 Hz, 1H), 6.67 (t, *J* = 7.2 Hz, 1H), 6.51 (d, *J* = 8.1 Hz, 1H), 4.36 – 4.29 (m, 1H), 2.88 (d, *J* = 17.3 Hz, 1H), 2.33 (dd, *J* = 17.4, 10.1 Hz, 1H), 2.13 (s, 3H), 1.72 (d, *J* = 6.3 Hz, 1H), 1.27 (d, *J* = 13.0 Hz, 1H). ^{13}C NMR (126 MHz, $CDCl_3$) δ 208.4, 143.9, 129.7, 116.2, 113.4, 53.1, 45.3, 30.9, 28.8.

Ethyl (*S*)-2-(1-phenylpyrrolidin-2-yl)acetate (6a)

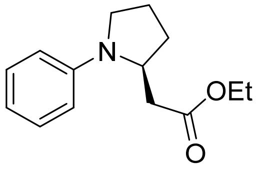

Ethyl (*S*)-2-(1-phenylpyrrolidin-2-yl)acetate was prepared according to the general procedure A. GC-MS m/z (% relative intensity): 233(9.9), 147(11.1), 146(100), 104(8.6), 77(11.8). ^{1}H NMR (500 MHz, $CDCl_3$) δ 7.24

(d, J = 8.1 Hz, 2H), 6.69 (t, J = 7.0 Hz, 1H), 6.62 (d, J = 7.6 Hz, 2H), 4.17 (q, J = 7.1 Hz, 3H), 3.46 – 3.39 (m, 1H), 3.23 – 3.14 (m, 1H), 2.79 (dd, J = 15.8, 7.9 Hz, 1H), 2.22 (dd, J = 14.5, 10.8 Hz, 1H), 2.10 – 1.99 (m, 3H), 1.94 – 1.86 (m, 1H), 1.29 (t, J = 7.2 Hz, 3H). ^{13}C NMR (126 MHz, $CDCl_3$) δ 172.16, 146.61, 129.50, 116.05, 112.03, 60.61, 55.46, 53.56, 48.02, 37.83, 31.06, 23.14, 14.40.

Ethyl 2-(1-(p-tolyl)pyrrolidin-2-yl)acetate (6b/7b)

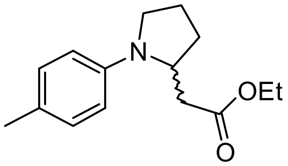

Ethyl 2-(1-(p-tolyl)pyrrolidin-2-yl)acetate was prepared according to the general procedure A. GC-MS m/z (% relative intensity): 247(9.9), 161(12.1), 160(100), 158(5.2), 130(5.1), 118(12), 91(20.7), 65(8.1). ^{1}H NMR (500 MHz, $CDCl_3$) δ 7.05 (d, J = 8.0 Hz, 2H), 6.54 (d, J = 8.1 Hz, 2H), 4.16 (q, J = 7.2 Hz, 3H), 3.40 (s, 1H), 3.19 – 3.12 (m, 1H), 2.78 (dd, J = 14.9, 3.1 Hz, 1H), 2.25 (s, 3H), 2.23 – 2.00 (m, 4H), 1.87 (s, 1H), 1.28 (t, J = 7.2 Hz, 3H). ^{13}C NMR (126 MHz, $CDCl_3$) δ 130.60, 112.70, 100.00, 61.17, 56.23, 48.85, 38.55, 31.65, 23.78, 20.97, 15.00.

Ethyl (*S*)-2-(1-(m-tolyl)pyrrolidin-2-yl)acetate (6c)

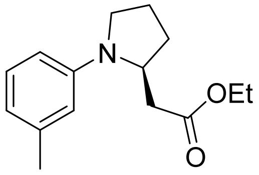

Ethyl (*S*)-2-(1-(m-tolyl)pyrrolidin-2-yl)acetate was prepared according to the general procedure A. GC-MS m/z (% relative intensity): 247(9.4), 161(12.4), 160(100), 118(8.6), 91(22.9), 65(7.2). ^{1}H NMR (500 MHz, $CDCl_3$) δ 7.13 (t, J = 8.0 Hz, 1H), 6.52 (d, J = 7.5 Hz, 1H), 6.44 (d, J = 7.3 Hz, 2H), 4.18 (q, J = 7.0 Hz, 3H), 3.45 – 3.38 (m, 1H), 3.18 (q, J = 8.3 Hz, 1H), 2.80 (dd, J = 15.1, 3.0 Hz, 1H), 2.32 (s, 3H), 2.21 (dd, J = 15.0, 10.4 Hz, 1H), 2.04 (q, J = 6.7 Hz, 3H), 1.88 (s, 1H), 1.29 (t, J = 7.1 Hz, 3H). ^{13}C NMR (126 MHz, $CDCl_3$) δ 172.83, 147.28, 139.82, 129.97, 117.62, 113.33, 109.85, 61.19, 56.05, 48.65, 38.51, 31.64, 23.72, 22.63, 15.00.

Ethyl (*S*)-2-(1-(o-tolyl)pyrrolidin-2-yl)acetate (6d)

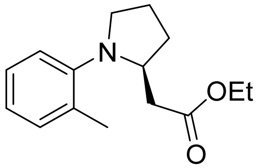

Ethyl (*S*)-2-(1-(o-tolyl)pyrrolidin-2-yl)acetate was prepared according to the general procedure A. GC-MS m/z (% relative intensity): 247(5.7), 161(11.5), 160(100), 130(6.3), 118(16.4), 117(5.9), 91(22.7), 65(7.6). ^{1}H NMR (500 MHz, $CDCl_3$) δ 7.14 (t, J = 8.1 Hz, 2H), 7.03 (d, J = 7.8 Hz, 1H), 6.92 (t, J = 7.3 Hz, 1H), 4.03 (dq, J = 13.8, 7.3 Hz, 3H), 3.51 (dt, J = 9.5, 7.2 Hz, 1H), 2.81 (td, J

= 8.6, 4.7 Hz, 1H), 2.53 (dd, J = 15.0, 4.1 Hz, 1H), 2.34 – 2.22 (m, 1H), 2.27 (s, 3H), 2.13 (dd, J = 14.9, 9.3 Hz, 1H), 2.00 – 1.79 (m, 2H), 1.69 (dq, J = 12.2, 8.3 Hz, 1H), 1.20 (t, J = 7.2 Hz, 3H). ^{13}C NMR (126 MHz, $CDCl_3$) δ 173.09, 148.39, 133.33, 131.98, 127.05, 122.86, 119.64, 60.92, 57.37, 53.65, 39.75, 32.27, 24.34, 20.04, 14.90.

Ethyl (*S*)-2-(1-(4-fluorophenyl)pyrrolidin-2-yl)acetate (6e)

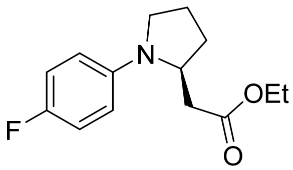

Ethyl (*S*)-2-(1-(4-fluorophenyl)pyrrolidin-2-yl)acetate was prepared according to the general procedure A. GC-MS m/z (% relative intensity): 251(8.9), 165(11.5), 164(100), 122(18.2), 109(4.9), 95(19.4). ^{1}H NMR (500 MHz, $CDCl_3$) δ 6.95 (t, J = 8.6 Hz, 2H), 6.54 – 6.50 (m, 2H), 4.16 (q, J = 7.1 Hz, 2H), 4.10 (s, 1H), 3.40 – 3.37 (m, 1H), 3.16 – 3.11 (m, 1H), 2.73 (dd, J = 14.9, 3.0 Hz, 1H), 2.23 – 2.20 (m, 1H), 2.15 – 1.98 (m, 3H), 1.91 – 1.87 (m, 1H), 1.27 (q, J = 6.3 Hz, 3H). ^{13}C NMR (126 MHz, $CDCl_3$) δ 172.69, 116.47 (d, J = 21.7 Hz), 113.10, 61.26, 49.13, 38.50, 31.77, 23.84, 14.99. ^{19}F NMR (376 MHz, $CDCl_3$) δ -131.05.

Ethyl 2-(1-(4-chlorophenyl)pyrrolidin-2-yl)acetate (6f/7f)

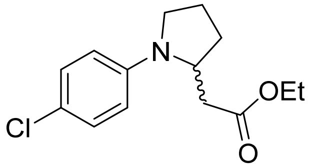

Ethyl 2-(1-(4-chlorophenyl)pyrrolidin-2-yl)acetate was prepared according to the general procedure A. GC-MS m/z (% relative intensity): 267(9.7), 182(31), 181(12.9), 180(100), 140(9.1), 138(16.1), 117(5.8), 111(16.5), 75(6.9). ^{1}H NMR (500 MHz, $CDCl_3$) δ 7.18 (d, J = 8.7 Hz, 2H), 6.57 – 6.53 (m, 2H), 4.15 (dt, J = 16.7, 8.3 Hz, 3H), 3.40 (s, 1H), 3.16 (t, J = 8.4 Hz, 1H), 2.72 (dd, J = 14.9, 3.0 Hz, 1H), 2.24 (s, 1H), 2.06 (s, 3H), 1.90 (s, 1H), 1.28 (t, J = 7.2 Hz, 3H). ^{13}C NMR (126 MHz, $CDCl_3$) δ 172.53, 129.90, 113.84, 61.34, 38.22, 31.71, 23.75, 15.00.

Ethyl (*S*)-2-(1-(4-methoxyphenyl)pyrrolidin-2-yl)acetate (6g)

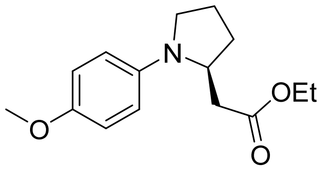

Ethyl (*S*)-2-(1-(4-methoxyphenyl)pyrrolidin-2-yl)acetate was prepared according to the general procedure A. GC-MS m/z (% relative intensity): 263(12.1), 177(13.6), 176(100), 134(6.6), 120(5.2), 92(5.4), 77(7.5). ^{1}H NMR (500 MHz, $CDCl_3$) δ 6.91 – 6.82 (m, 2H), 6.61 – 6.54 (m, 2H), 4.16 (q, J = 7.1 Hz, 2H), 4.10 (ddd, J = 10.4, 7.5, 3.3 Hz, 1H), 3.76 (s, 3H), 3.39 (td, J = 8.0, 3.5 Hz, 1H), 3.13 (q, J = 8.2 Hz, 1H), 2.76 (dd, J = 15.0, 3.0 Hz, 1H), 2.20 (dd, J = 14.9, 10.4

Hz, 1H), 2.11 – 1.97 (m, 3H), 1.87 (dd, *J* = 10.7, 5.7 Hz, 1H), 1.28 (t, *J* = 7.2 Hz, 3H). ^{13}C NMR (126 MHz, $CDCl_3$) δ 172.89, 151.75, 142.16, 115.92, 113.55, 61.17, 56.70, 56.56, 49.25, 38.73, 31.75, 23.87, 15.01.

Ethyl (*S*)-2-(1-(3-bromophenyl)pyrrolidin-2-yl)acetate (6h)

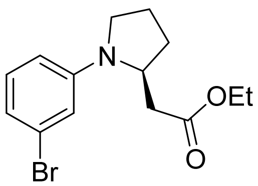

Ethyl (*S*)-2-(1-(3-bromophenyl)pyrrolidin-2-yl)acetate was prepared according to the general procedure A. GC-MS m/z (% relative intensity): 313(10.2), 311(10.5), 227(10.8), 226(93.5), 225(12.6), 224(100), 184(8.4), 182(7.9), 157(7.9), 155(6.9), 145(7.8). ^{1}H NMR (500 MHz, $CDCl_3$) δ 7.11 (s, 1H), 6.87 (s, 2H), 6.64 (s, 1H), 4.16 (q, *J* = 7.3 Hz, 3H), 3.50 – 3.47 (m, 1H), 3.23 – 3.18 (m, 1H), 2.74 (dd, *J* = 15.4, 3.3 Hz, 1H), 2.33 – 2.29 (m, 1H), 2.11 – 2.07 (m, 2H), 1.96 – 1.92 (m, 1H), 1.77 – 1.46 (m, 1H), 1.28 (t, *J* = 7.2 Hz, 3H). ^{13}C NMR (126 MHz, $CDCl_3$) δ 131.45, 124.40, 61.48, 37.89, 31.51, 23.53, 14.94.

Ethyl (*S*)-2-(1-(2-cyanophenyl)pyrrolidin-2-yl)acetate (6i)

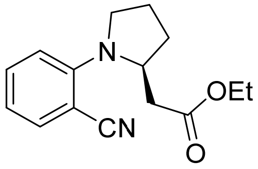

Ethyl (*S*)-2-(1-(2-cyanophenyl)pyrrolidin-2-yl)acetate was prepared according to the general procedure A. GC-MS m/z (% relative intensity): 258(7.6), 172(12.8), 171(100), 169(3.2), 155(2.5), 154(11.3), 144(2.5), 131(2.1), 130(2.1), 129(12.7), 116(2.1), 104(2.2), 103(3.3), 102(8.3). ^{1}H NMR (500 MHz, $CDCl_3$) δ 7.46 (dd, *J* = 7.8, 1.4 Hz, 1H), 7.35 (dd, *J* = 8.6, 1.6 Hz, 1H), 6.75 (d, *J* = 8.7 Hz, 1H), 6.71 (t, *J* = 8.4 Hz, 1H), 4.44 – 4.36 (m, 1H), 4.14 (q, *J* = 7.1 Hz, 2H), 3.99 (dt, *J* = 9.6, 7.0 Hz, 1H), 3.51 (ddd, *J* = 9.6, 7.7, 5.4 Hz, 1H), 2.77 (dd, *J* = 15.5, 3.0 Hz, 1H), 2.32 – 2.25 (m, 1H), 2.27 – 2.20 (m, 1H), 2.03 (dp, *J* = 12.2, 6.6 Hz, 1H), 1.93 (td, *J* = 13.4, 7.2 Hz, 1H), 1.85 (tt, *J* = 12.5, 5.9 Hz, 1H), 1.25 (t, *J* = 7.1 Hz, 3H). ^{13}C NMR (126 MHz, $CDCl_3$) δ 172.12, 150.22, 136.58, 134.20, 121.54, 117.87, 115.93, 100.00, 97.82, 61.36, 56.54, 52.32, 38.68, 32.29, 24.78, 14.93.

Ethyl 2-((2*S*)-5-methyl-1-phenylpyrrolidin-2-yl)acetate (6k)

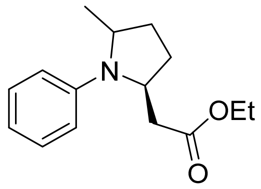

Ethyl 2-((2*S*)-5-methyl-1-phenylpyrrolidin-2-yl)acetate was prepared according to the general procedure A. GC-MS m/z (% relative intensity): 247(8.1), 232(8.5), 161(12), 160(100), 144(16.2), 130(5.2), 118(11.3), 117(5.4), 104(17), 91(6.2), 77(25.6), 51(5.8). ^{1}H NMR (400 MHz, $CDCl_3$) δ 7.2 (dddd, *J* = 12.1, 7.0, 3.6, 1.3 Hz, 2H), 6.7 – 6.6 (m, 1H), 6.6 – 6.5 (m, 2H), 4.2 – 4.1 (m, 1H), 3.8 – 3.7 (m, 1H), 3.0 (dd, *J* = 16.9, 2.9 Hz, 1H), 2.5 (dd, *J* = 16.9, 10.0 Hz, 1H), 2.4 – 2.3 (m, 1H), 2.2 (d, *J* = 17.3 Hz, 4H), 2.1 – 2.0 (m, 1H), 1.7 (tt, *J* = 15.6, 5.6 Hz, 2H), 1.3 (d, *J* = 6.1 Hz, 2H), 1.1 (d, *J* = 6.1 Hz, 1H). ^{13}C NMR (126 MHz, $CDCl_3$) δ 173.05, 172.66, 147.39, 145.17, 130.07, 130.00, 116.76, 116.19, 114.16, 112.80, 61.19, 61.16, 58.27, 56.32, 55.38, 53.39, 41.01, 37.08, 32.91, 30.94, 30.87, 29.02, 22.32, 18.96, 15.01.

Ethyl 2-(1-phenylpiperidin-2-yl)acetate (6m/7m)

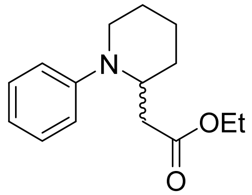

Ethyl 2-(1-phenylpiperidin-2-yl)acetate was prepared according to the general procedure A. GC-MS m/z (% relative intensity): 247(7.3), 161(12.3), 160(100), 132(6.6), 104(9.4), 77(11.4). ^{1}H NMR (500 MHz, $CDCl_3$) δ 7.23 (d, *J* = 7.5 Hz, 2H), 6.95 (d, *J* = 8.1 Hz, 2H), 6.81 (t, *J* = 7.3 Hz, 1H), 4.38 – 4.31 (m, 1H), 4.03 (q, *J* = 7.1 Hz, 2H), 3.35 (d, *J* = 12.3 Hz, 1H), 2.91 (t, *J* = 11.3 Hz, 1H), 2.53 (dd, *J* = 14.7, 9.4 Hz, 1H), 2.45 (dd, *J* = 14.8, 5.0 Hz, 1H), 1.94 – 1.81 (m, 1H), 1.80 – 1.73 (m, 1H), 1.71 – 1.60 (m, 4H), 1.20 (t, *J* = 7.1 Hz, 3H). ^{13}C NMR (126 MHz, $CDCl_3$) δ 172.60, 150.49, 129.30, 119.38, 117.05, 60.51, 53.29, 44.14, 32.98, 28.89, 25.65, 19.37, 14.30.

Ethyl (*R*)-2-(4-phenylmorpholin-3-yl)acetate (6n)

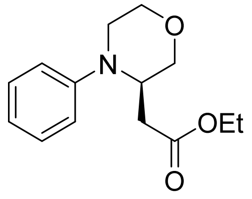

Ethyl (*R*)-2-(4-phenylmorpholin-3-yl)acetate was prepared according to the general procedure A. GC-MS m/z (% relative intensity): 249(11.5), 163(11.3), 162(100), 134(5.3), 132(7.4), 119(9.1), 118(8.3), 117(8.9), 106(10.8), 105(18.4), 104(65.2), 91(13.6), 78(5.4), 77(46.2), 51(6.7). ^{1}H NMR (500 MHz, $CDCl_3$) δ 7.30 – 7.26 (m, 2H), 6.89 (d, *J* = 8.2 Hz, 2H), 6.86 (t, *J* = 7.3 Hz, 1H), 4.16 (d, *J* = 9.5 Hz, 1H), 4.05 (qd, *J* = 7.1, 2.9 Hz, 2H), 4.00 (d, *J* = 10.7 Hz, 1H), 3.91 (d, *J* = 11.5 Hz, 1H), 3.85 (d, *J* = 11.4 Hz, 1H), 3.71 (td, *J* = 11.3, 3.2 Hz, 1H), 3.18 (d, *J* = 11.9 Hz, 1H), 3.10 (td, *J* = 11.8, 3.5 Hz, 1H), 2.86 (dd, *J* = 15.8, 10.2 Hz, 1H), 2.35 (d, *J* = 15.7 Hz, 1H), 1.21 (t, *J* =

7.1 Hz, 3H). ^{13}C NMR (126 MHz, $CDCl_3$) δ 173.00, 149.64, 130.14, 120.45, 116.29, 70.16, 67.64, 61.28, 52.87, 43.82, 30.89, 14.87.

Diethyl 2,2'-(1-phenylpyrrolidine-2,5-diyl)diacetate (8a-c)

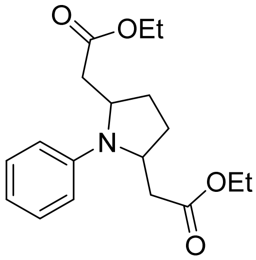

Diethyl 2,2'-(1-phenylpyrrolidine-2,5-diyl)diacetate was prepared according to general procedure B. GC-MS m/z (% relative intensity): 319(6.3), 233(15.5), 232(100), 158(10.3),145(6.4), 144(55.3), 118(5.3), 104(13.8), 77(18.2); ^{1}H NMR (500 MHz, $CDCl_3$) δ 7.2 (ddd, J = 9.3, 7.2, 1.9 Hz, 2H), 6.7 – 6.6 (m, 2H), 4.2 – 4.1 (m, 6H), 2.9 (dd, J = 15.1, 3.4 Hz, 1H), 2.3 – 2.1 (m, 2H), 2.0 (s, 3H), 1.9 – 1.8 (m, 2H), 1.6 (q, J = 5.4 Hz, 1H), 1.3 – 1.2 (m, 24H), 0.9 (d, J = 6.6 Hz, 1H). ^{13}C NMR (126 MHz, $CDCl_3$) δ 130.2, 123.4, 117.5, 113.0, 61.3, 61.2, 58.0, 40.8, 37.1, 32.3, 30.9, 23.4, 21.8, 15.0, 14.9, 14.9.

Ethyl 2-(5-(2-oxopropyl)-1-phenylpyrrolidin-2-yl)acetate (9a/10a)

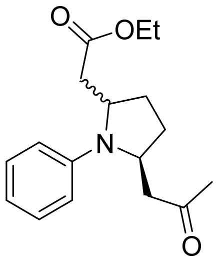

Ethyl 2-(5-(2-oxopropyl)-1-phenylpyrrolidin-2-yl)acetate was prepared according to detailed procedure for (**9a/10a**). GC-MS m/z (% relative intensity): 289(9.4), 233(6.9), 232(42.8), 202(34.9), 158(13.7), 156(5.7), 145(10.9), 144(100), 143(6.7), 130(5.5), 118(11.8), 117(8.1), 104(14.9), 91(7.9), 77(29.7), 51(6), 143(6.7), 130(5.5), 118(11.8), 117(8.1), 104(14.9), 91(7.9), 77(29.7), 51(6.0). ^{1}H NMR (500 MHz, $CDCl_3$) δ 7.3 – 7.2 (m, 3H), 6.7 (dt, J = 20.8, 7.3 Hz, 1H), 6.6 (dd, J = 17.4, 8.2 Hz, 2H), 4.2 – 4.1 (m, 4H), 2.3 (t, J = 7.4 Hz, 3H), 2.3 – 2.2 (m, 5H), 2.2 – 2.1 (m, 1H), 2.1 – 2.0 (m, 1H), 1.7 (tt, J = 14.5, 6.4 Hz, 2H), 1.3 (d, J = 22.9 Hz, 6H), 1.3 – 1.2 (m, 28H), 1.1 (t, J = 6.2 Hz, 1H), 0.9 (td, J = 6.9, 2.9 Hz, 4H). ^{13}C NMR (126 MHz, $CDCl_3$) δ 130.2, 117.5, 113.0, 78.0, 77.7, 77.5, 61.3, 58.2, 56.7, 49.7, 40.9, 31.3, 30.8, 30.4, 30.2, 30.1, 30.0, 29.8, 29.5, 25.5, 23.4, 16.5, 15.0, 14.8.

Ethyl 2-(3-(trifluoromethyl)-5,6,7,8-tetrahydro-[1,2,4]triazolo[4,3-a]pyrazin-8-yl)acetate (12)

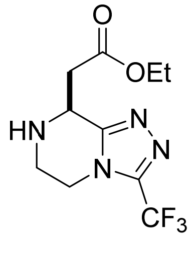

Diethyl 2,2'-(1-phenylpyrrolidine-2,5-diyl)diacetate was prepared according to general procedure A. GC-MS m/z (% relative intensity): 278(11.4), 259)8.5), 249(5.6), 234(28.3), 232(14.3), 219(10.5), 205(14.6), 204(56.6), 192(12.6), 191(64.9), 177(6.3), 176(6.7), 165(7.7), 96(5.1), 70(16.5), 69(26.7), 68(11.8), 67(9.8), 59(100), 56(15.3), 55(7.8), 54(20.4); ^{1}H NMR (500 MHz, $CDCl_3$) δ 4.1 – 4.0 (m, 4H), 2.9 (dd, J = 15.8, 7.2 Hz, 1H), 2.7 (dd, J = 15.9, 8.0 Hz, 1H), 2.3 (td, J = 7.5, 2.1 Hz, 1H), 1.7 – 1.6 (m, 2H), 1.3 (d, J = 2.2 Hz, 3H). ^{19}F NMR (376 MHz, $CDCl_3$) δ -64.0. E.r. of the product was determined via chiral HPLC but its absolute configuration was not determined.

Diethyl 2,2'-(3-(trifluoromethyl)-5,6-dihydro-[1,2,4]triazolo[4,3-a]pyrazine-7,8(8H)-diyl)diacetate (13)

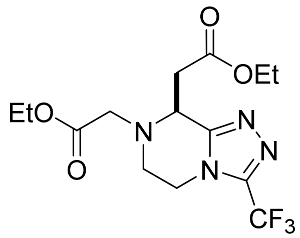

Diethyl 2,2'-(3-(trifluoromethyl)-5,6-dihydro-[1,2,4]triazolo[4,3-a]pyrazine-7,8(8H)-diyl)diacetate was prepared according to general procedure B using 3-(trifluoromethyl)-5,6,7,8-tetrahydro-[1,2,4]triazolo[4,3-*a*]pyrazine as the substrate. GC-MS m/z (% relative intensity): 364(6.8), 292(13.1), 291(100), 277(23.5), 245(31.3), 219(21.3), 218(7.4), 217(13.9), 192(6.4), 191(53.2), 176(8.6), 174(6.5), 150(8.8), 128(6.1), 82(8.3), 69(12.4), 68(8.8), 55(11.9), 54(12.7). ^{1}H NMR (500 MHz, $CDCl_3$) δ 4.3 – 4.2 (m, 5H), 4.2 – 4.0 (m, 7H), 3.3 (dt, J = 12.8, 5.2 Hz, 1H), 3.1 (ddd, J = 12.3, 7.0, 4.5 Hz, 1H), 2.9 (dd, J = 15.8, 7.2 Hz, 1H), 2.7 (dd, J = 15.9, 8.0 Hz, 1H), 1.6 (d, J = 7.6 Hz, 2H), 0.9 (td, J = 6.9, 3.1 Hz, 4H), 0.6 (s, 1H), 0.5 (td, J = 8.3, 4.0 Hz, 1H). ^{13}C NMR (126 MHz, $CDCl_3$) δ 146.8, 130.3, 130.2, 117.5, 113.0, 78.0, 77.8, 77.5, 61.3, 58.2, 56.7, 49.7, 40.9, 34.7, 32.7, 31.7, 31.3, 30.9, 30.8, 30.4, 30.2, 30.1, 30.0, 29.8, 29.7, 29.5, 29.3, 29.0, 25.5, 23.4, 16.5, 15.0, 14.8, 11.7, 0.7. ^{19}F NMR (376 MHz, $CDCl_3$) δ -63.5. E.r. of the product was determined via chiral HPLC but its absolute configuration was not determined.

Ethyl 2-(3-(trifluoromethyl)-5,6-dihydro-[1,2,4]triazolo[4,3-a]pyrazin-7(8H)-yl)acetate (14)

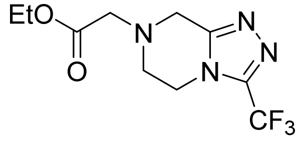

Ethyl 2-(3-(trifluoromethyl)-5,6-dihydro-[1,2,4]triazolo[4,3-a]pyrazin-7(8H)-yl)acetate was prepared according to general procedure A using 3-(trifluoromethyl)-5,6,7,8-tetrahydro-[1,2,4]triazolo[4,3-*a*]pyrazine as the substrate. GC-MS m/z (% relative intensity): 278(9.1), 230(3.3), 205(8.9), 205(100), 191(33.3),

177(12.9), 149(9.3), 69(12.0), 56(11.0), 54(79.4); ^{1}H NMR (500 MHz, $CDCl_3$) δ 4.2 (q, *J* = 7.1 Hz, 2H), 4.2 (t, *J* = 5.5 Hz, 2H), 4.1 (s, 2H), 3.5 (s, 2H), 3.2 – 3.1 (m, 2H), 1.3 (t, *J* = 7.2 Hz, 3H). ^{13}C NMR (126 MHz, $CDCl_3$) δ 170.2, 152.5, 120.3, 78.0, 77.8, 77.5, 61.9, 58.1, 49.2, 48.7, 44.3, 14.9. ^{19}F NMR (376 MHz, $CDCl_3$) δ -63.4.

(*S*)-1-(1-phenylpyrrolidin-2-yl)propan-2-one (3a)

^{1}H NMR (400 MHz, $CDCl_3$)

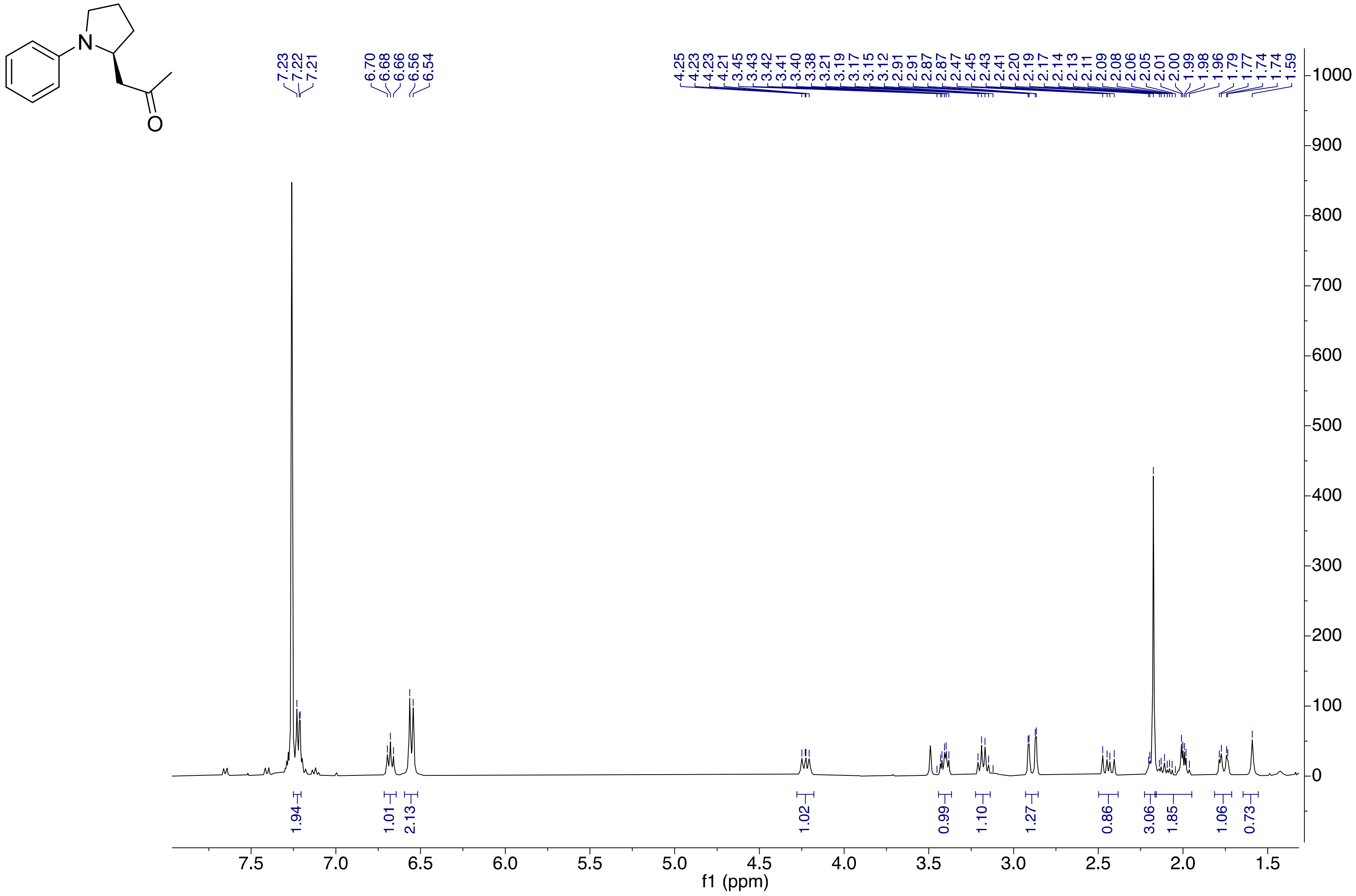

(*S*)-1-(1-phenylpyrrolidin-2-yl)propan-2-one (3a)

^{13}C NMR (101 MHz, $CDCl_3$)

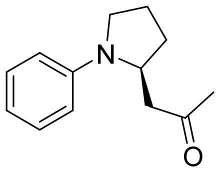

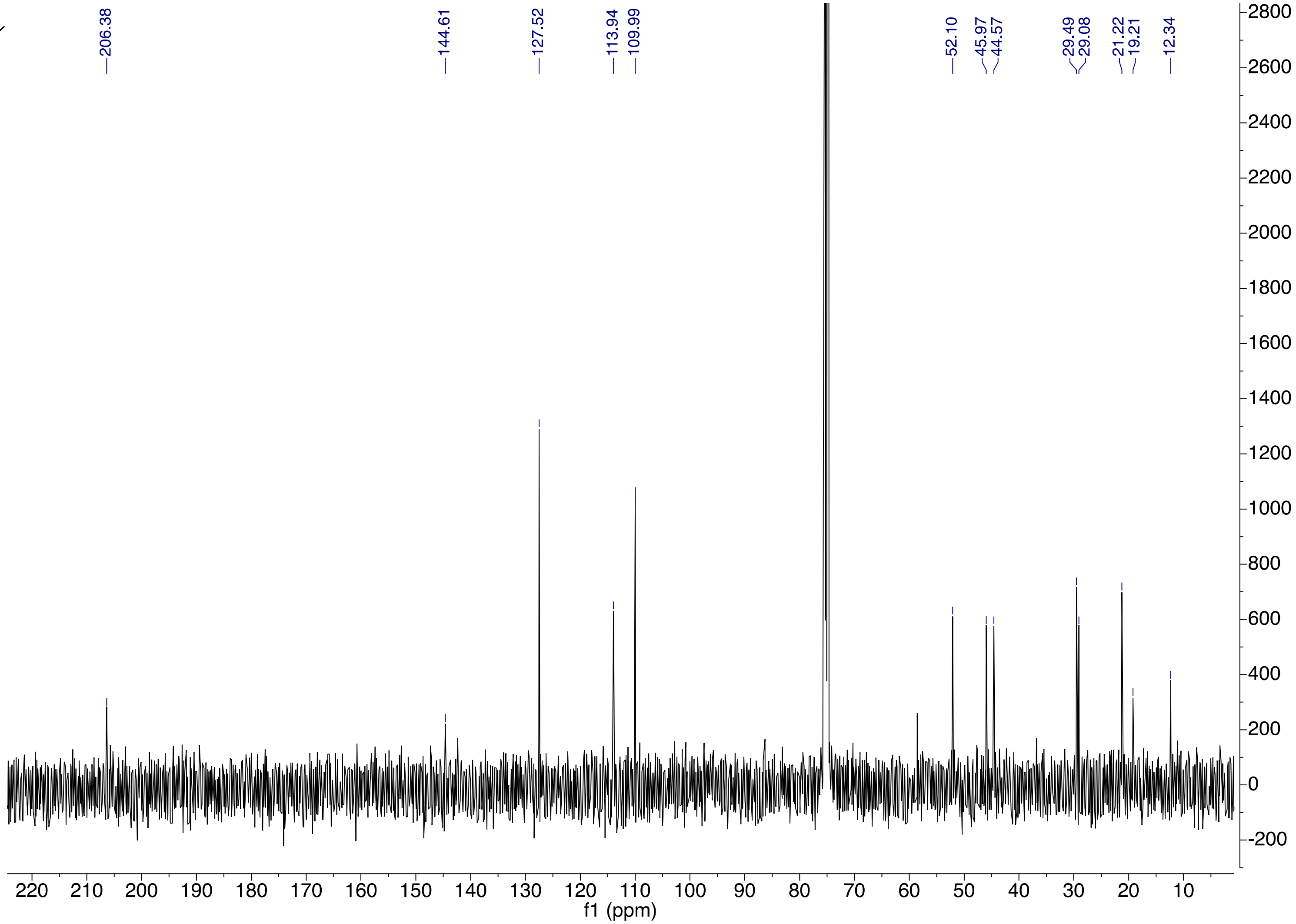

(*S*)-1-(1-(p-tolyl)pyrrolidin-2-yl)propan-2-one (3b)

^{1}H NMR (500 MHz, $CDCl_3$)

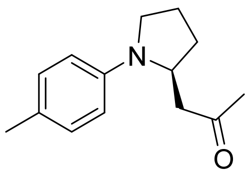

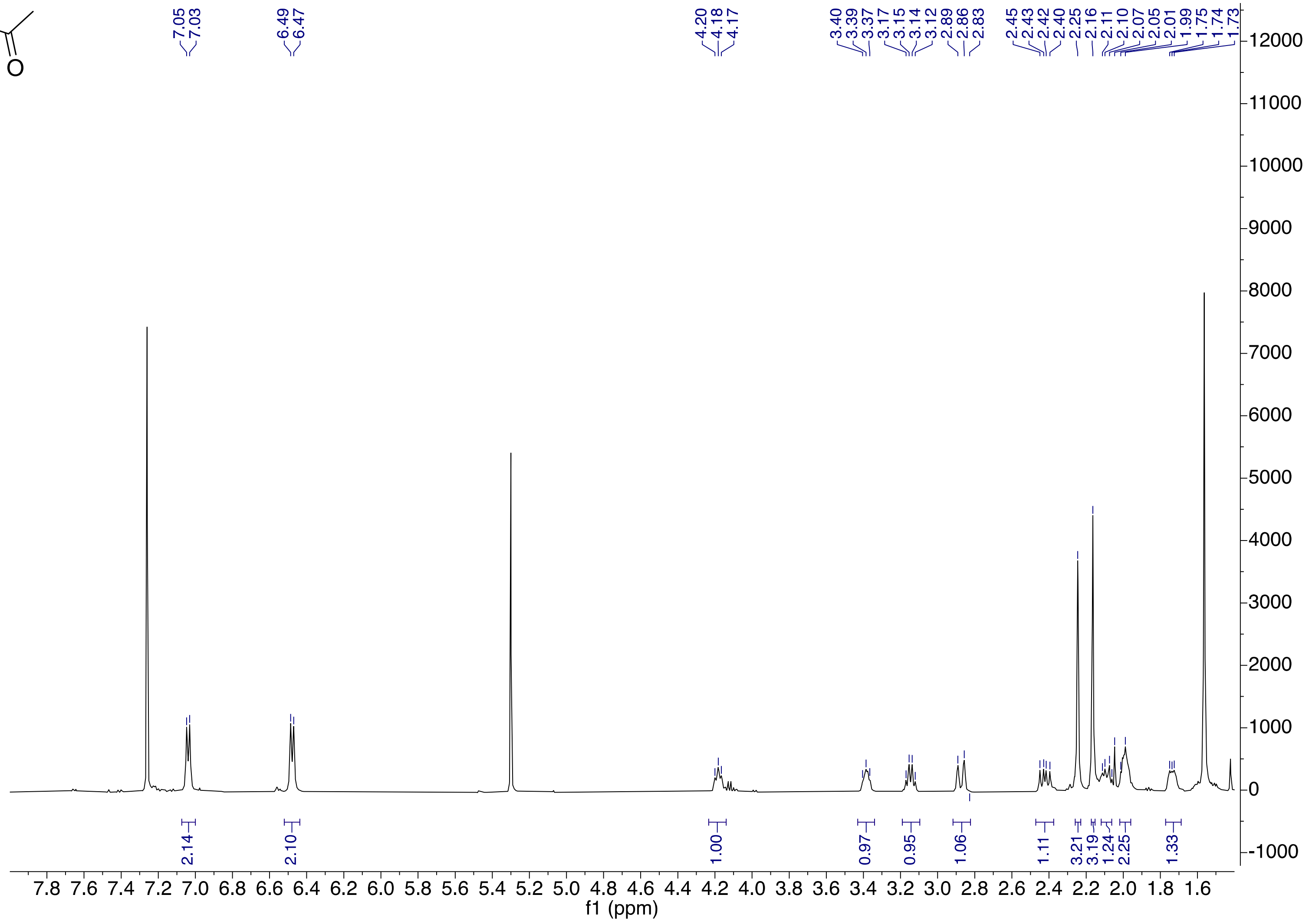

(*S*)-1-(1-(p-tolyl)pyrrolidin-2-yl)propan-2-one (3b)

^{13}C NMR (126 MHz, $CDCl_3$)

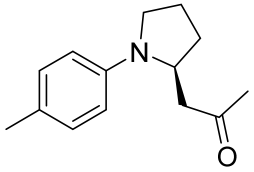

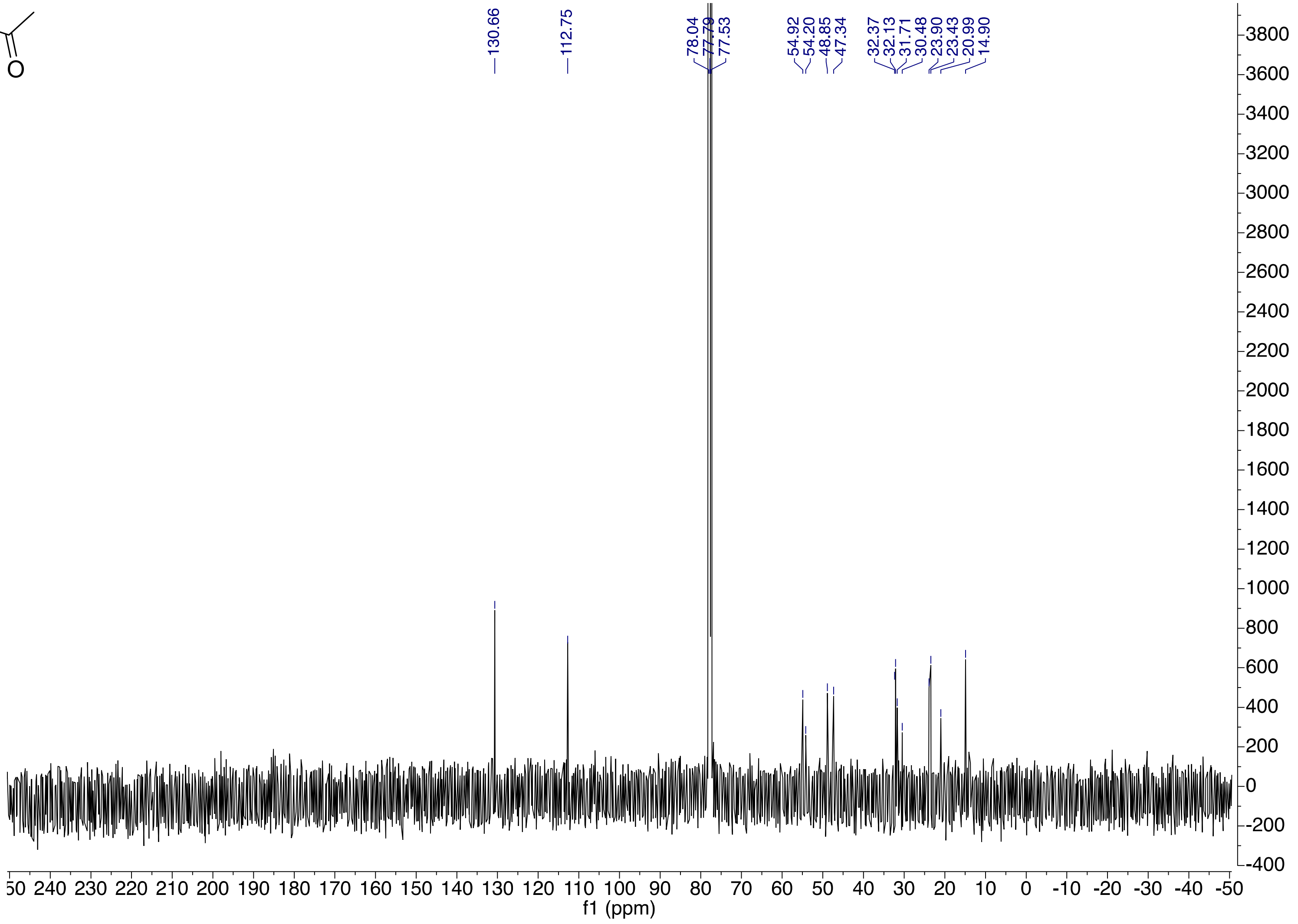

(*S*)-1-(1-(m-tolyl)pyrrolidin-2-yl)propan-2-one (3c)

^{1}H NMR (500 MHz, $CDCl_3$)

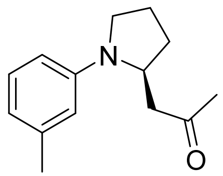

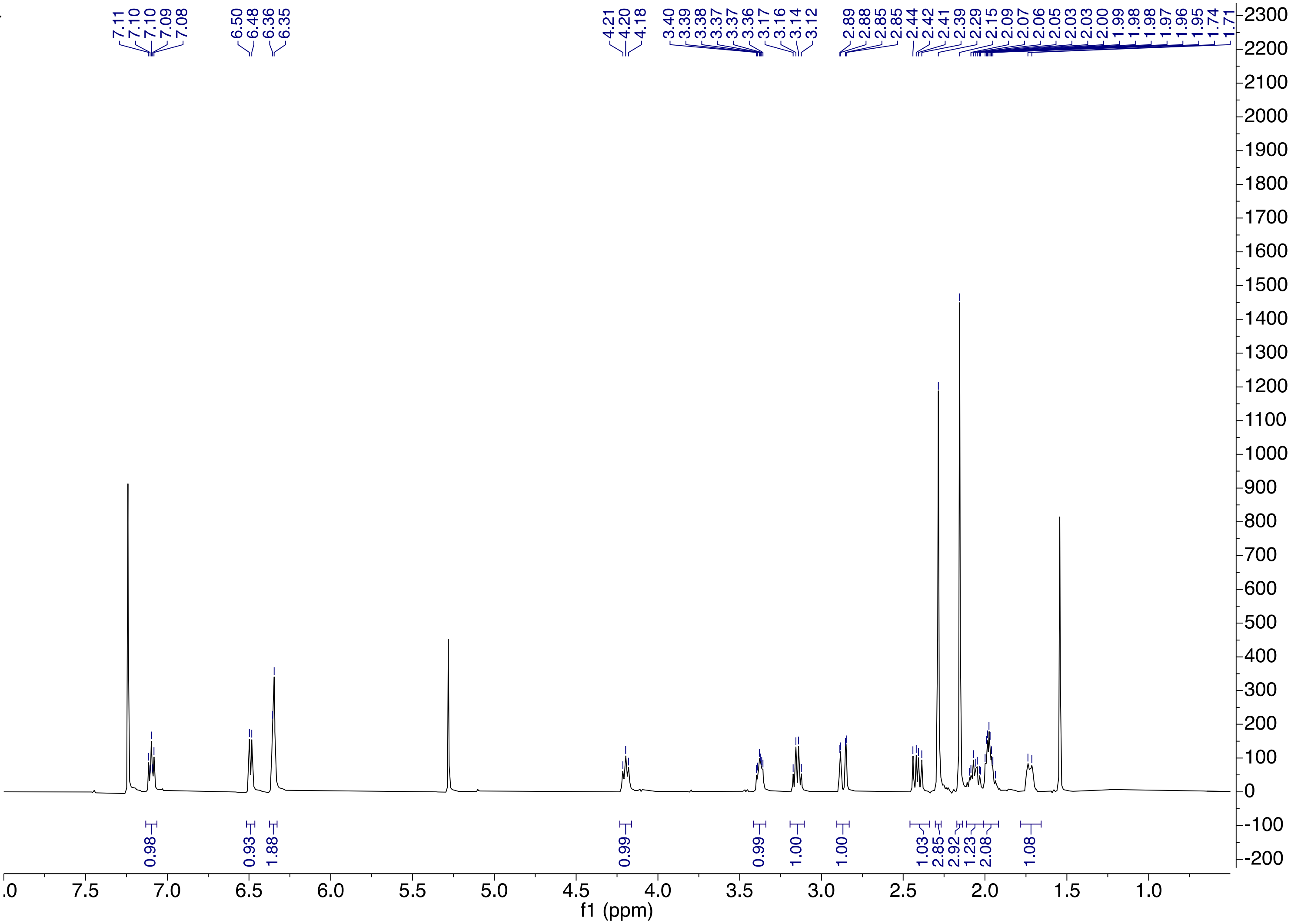

(*S*)-1-(1-(m-tolyl)pyrrolidin-2-yl)propan-2-one (3c)

^{13}C NMR (126 MHz, $CDCl_3$)

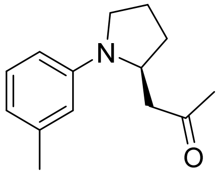

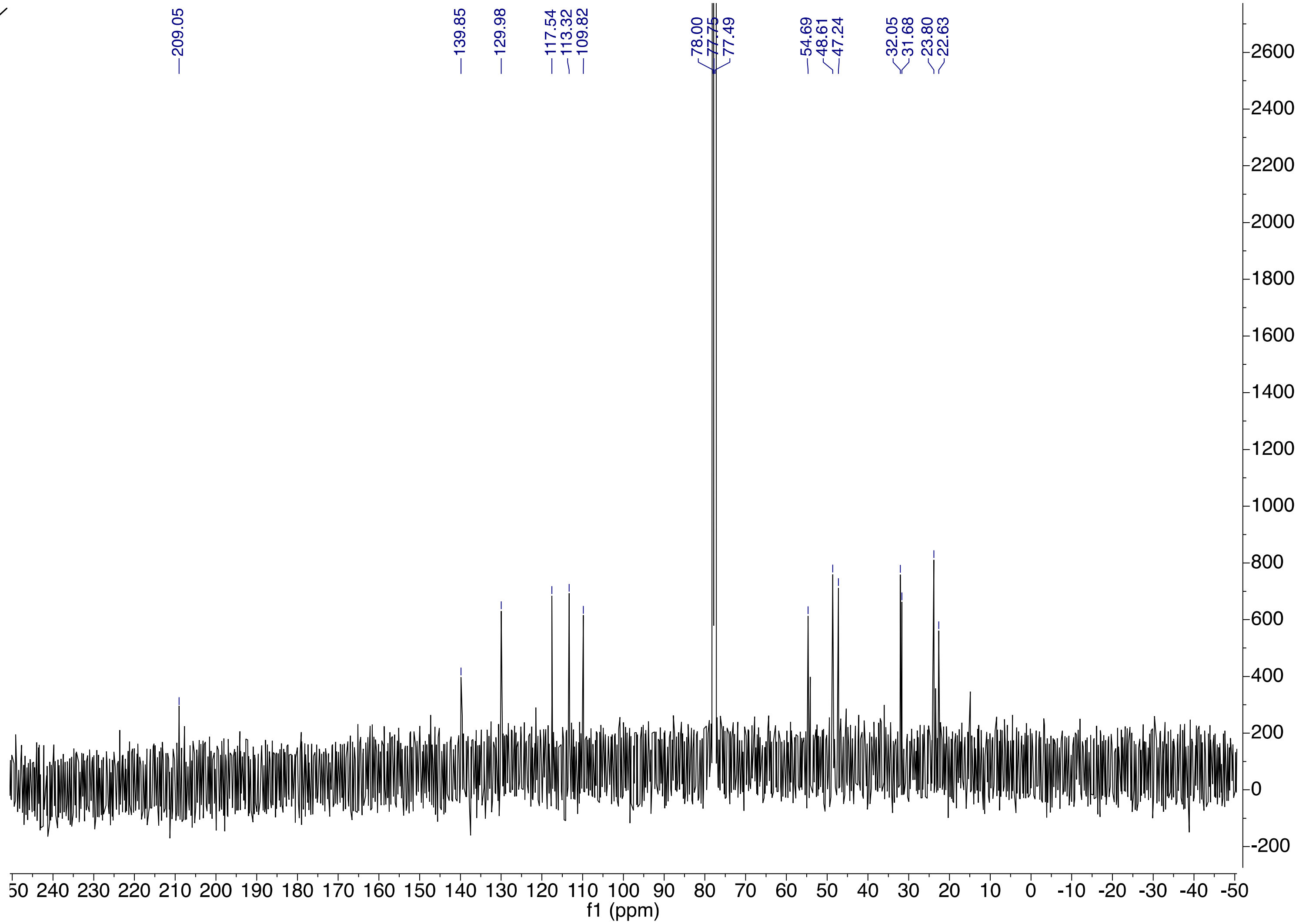

(*S*)-1-(1-(o-tolyl)pyrrolidin-2-yl)propan-2-one (3d)

^{1}H NMR (500 MHz, $CDCl_3$)

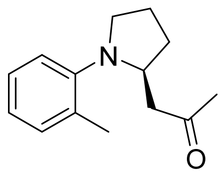

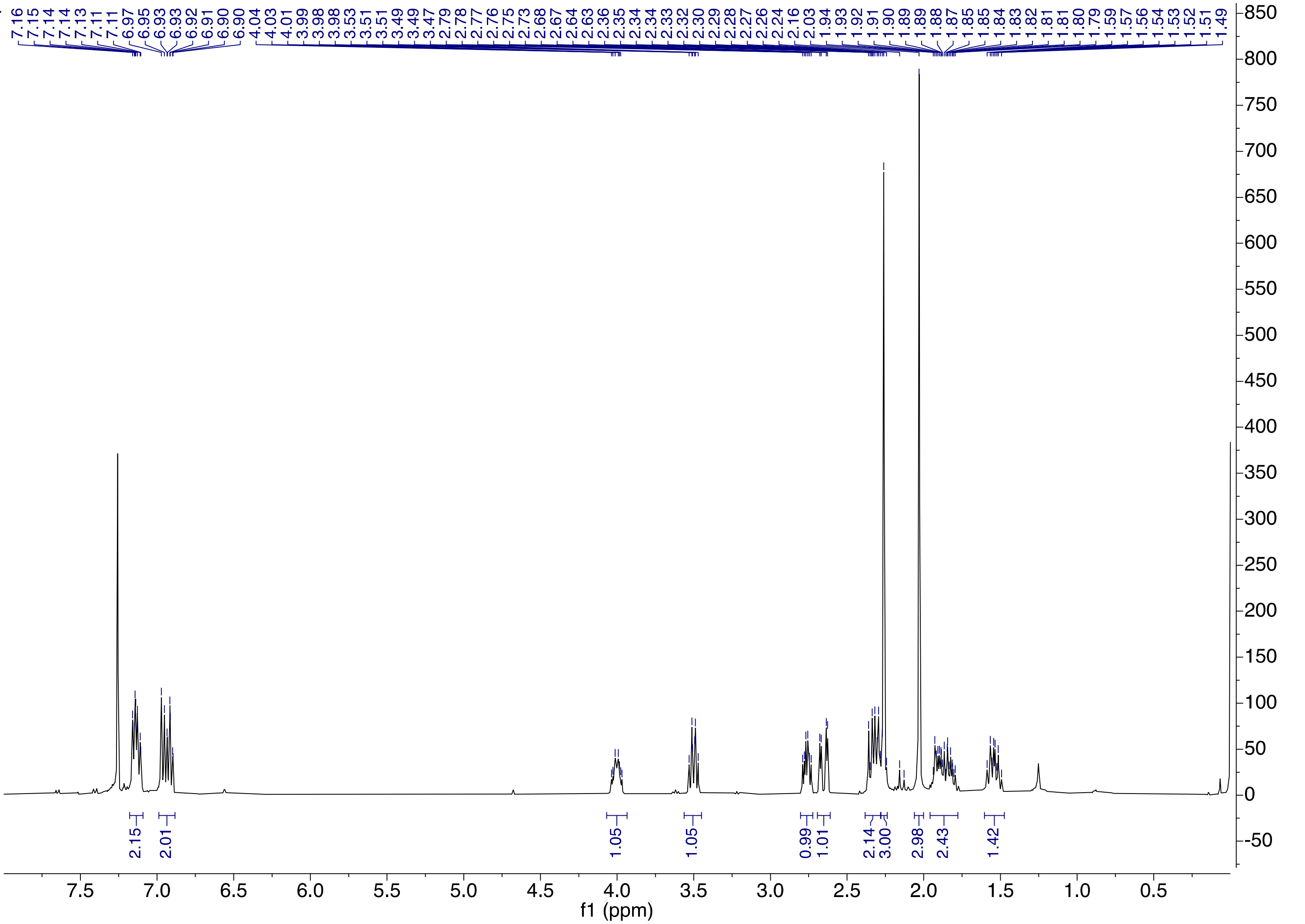

(*S*)-1-(1-(4-fluorophenyl)pyrrolidin-2-yl)propan-2-one (3e)

^{1}H NMR (400 MHz, $CDCl_3$)

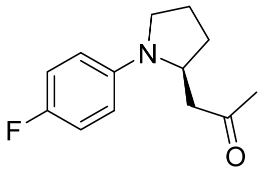

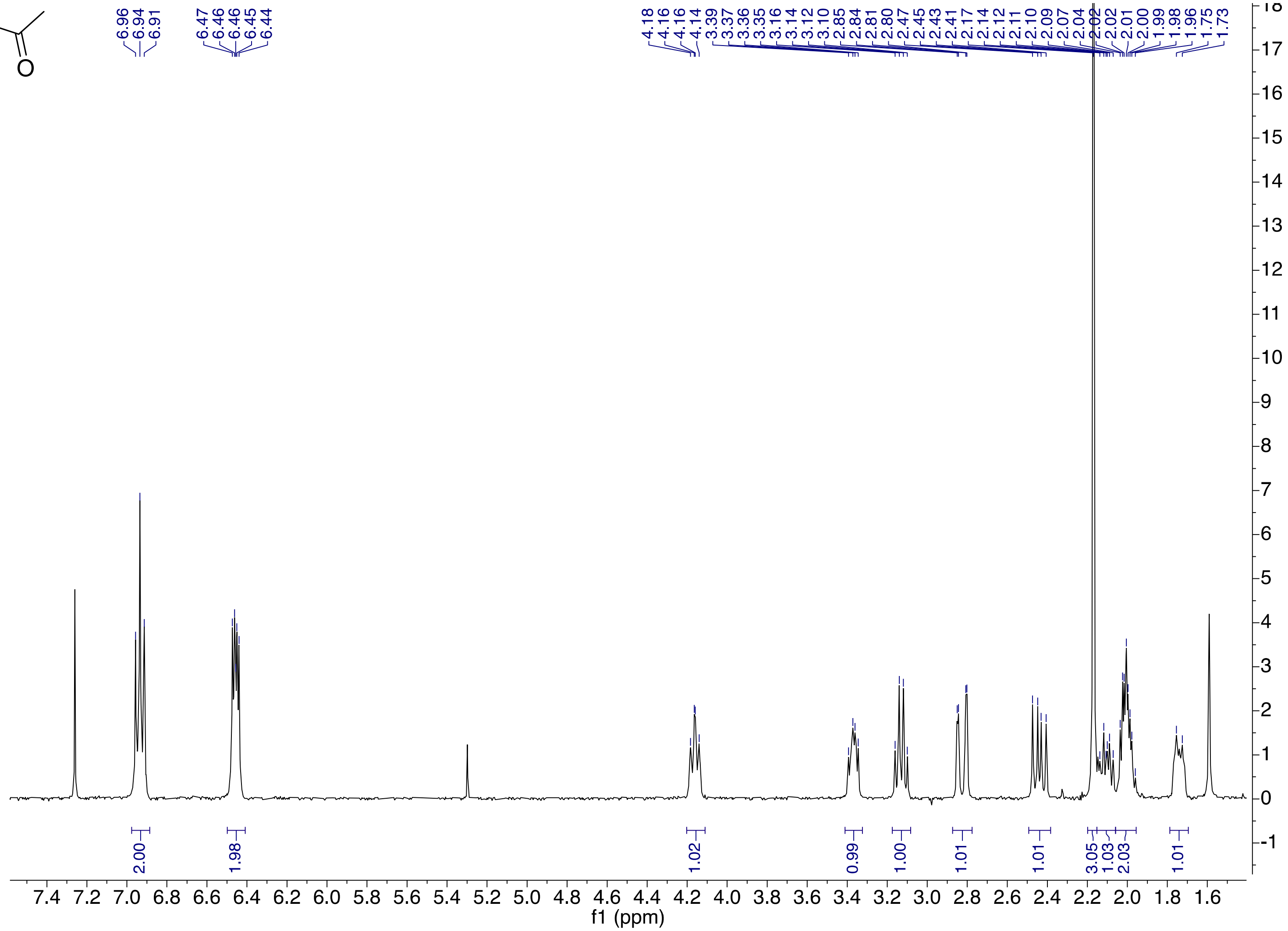

(*S*)-1-(1-(4-fluorophenyl)pyrrolidin-2-yl)propan-2-one (3e)

^{13}C NMR (101 MHz, $CDCl_3$)

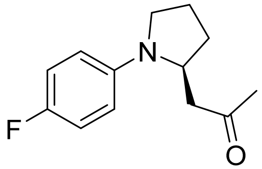

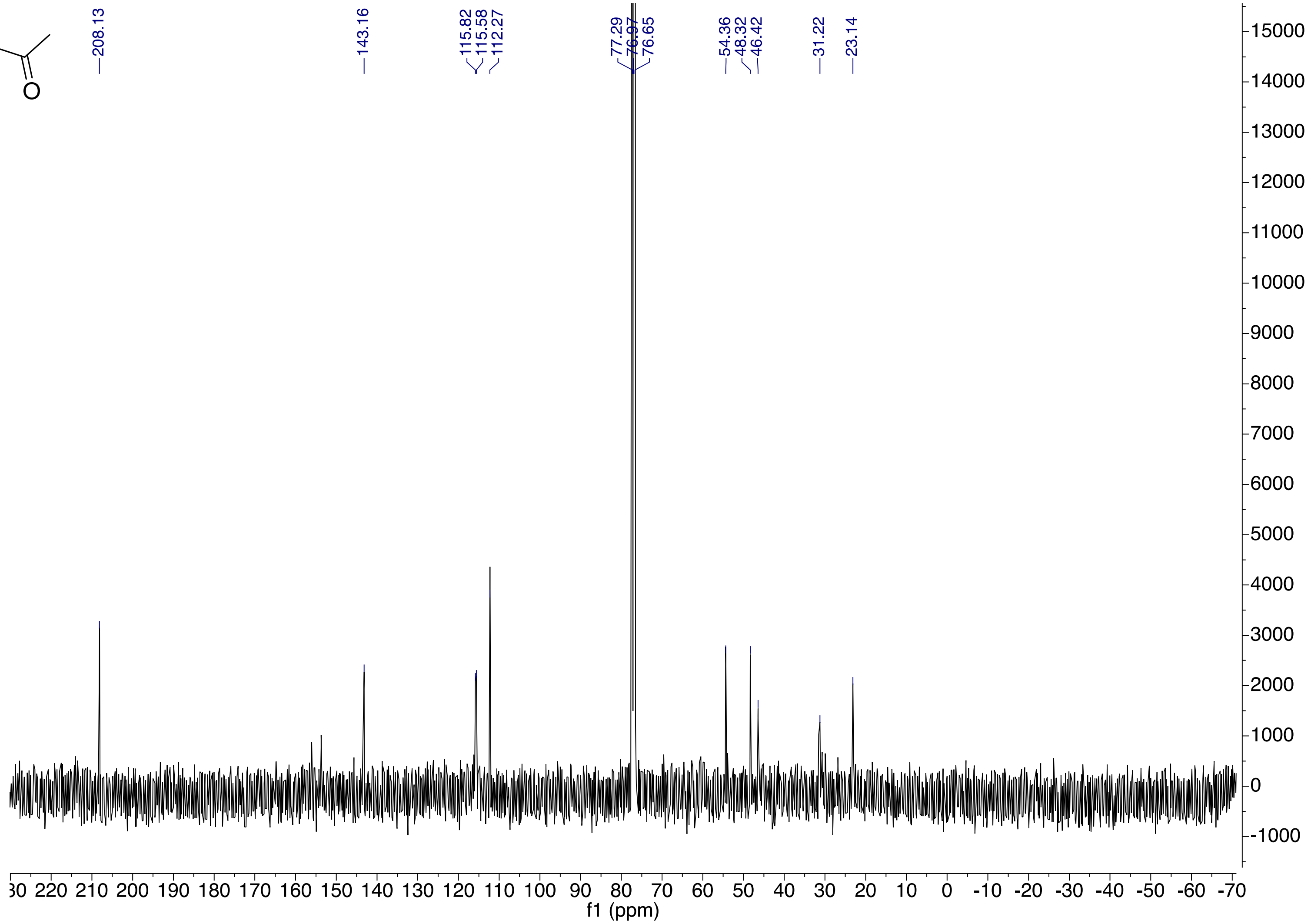

(*S*)-1-(1-(4-fluorophenyl)pyrrolidin-2-yl)propan-2-one (3e)

^{19}F NMR (376 MHz, $CDCl_3$)

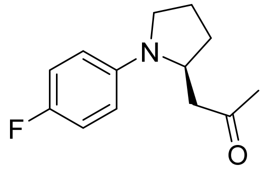

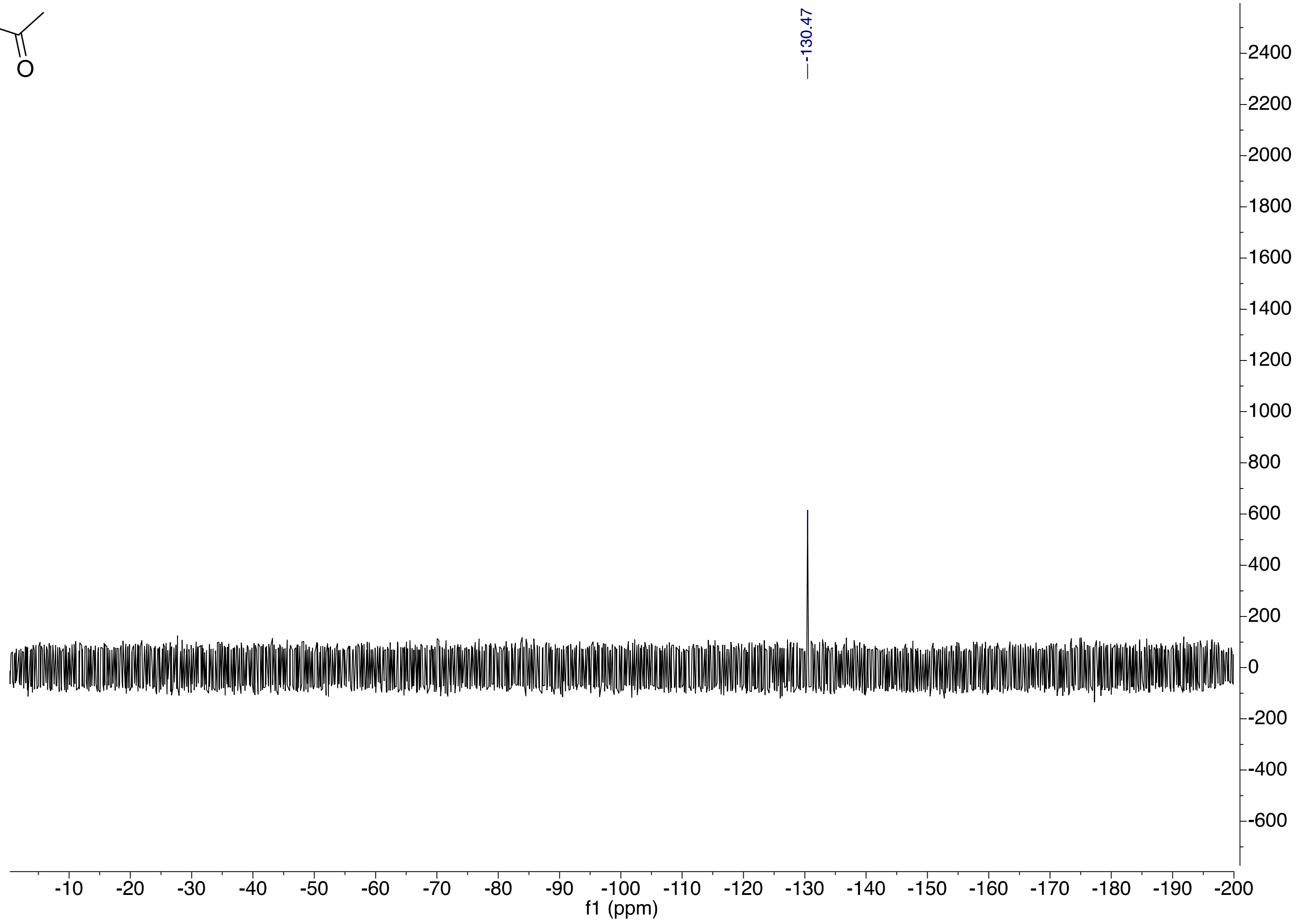

(*S*)-1-(1-(4-chlorophenyl)pyrrolidin-2-yl)propan-2-one (3f)

^{1}H NMR (500 MHz, $CDCl_3$)

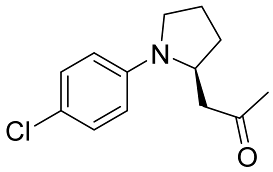

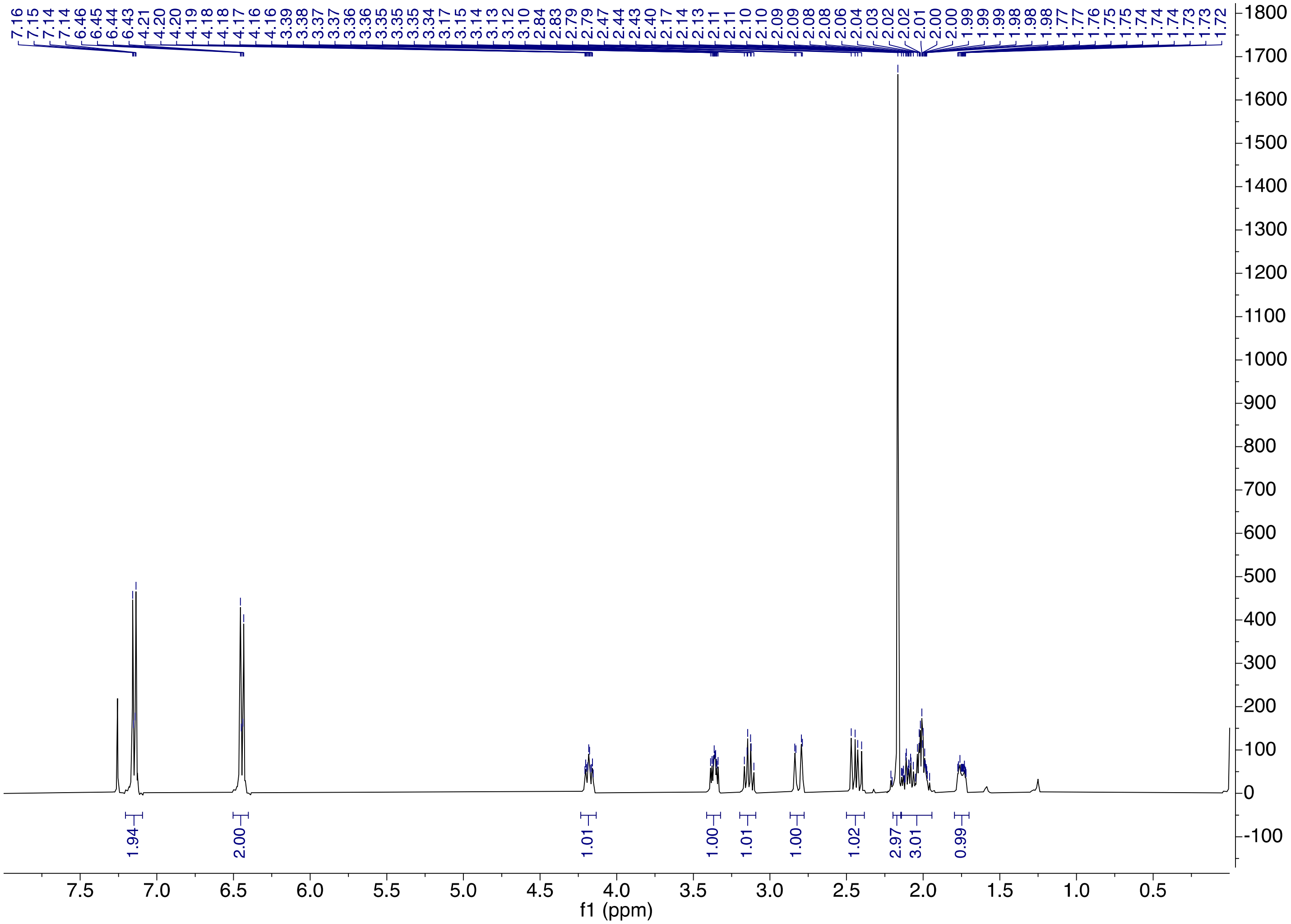

(*S*)-1-(1-(4-chlorophenyl)pyrrolidin-2-yl)propan-2-one (3f)

^{13}C NMR (126 MHz, $CDCl_3$)

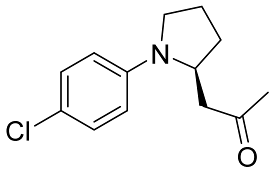

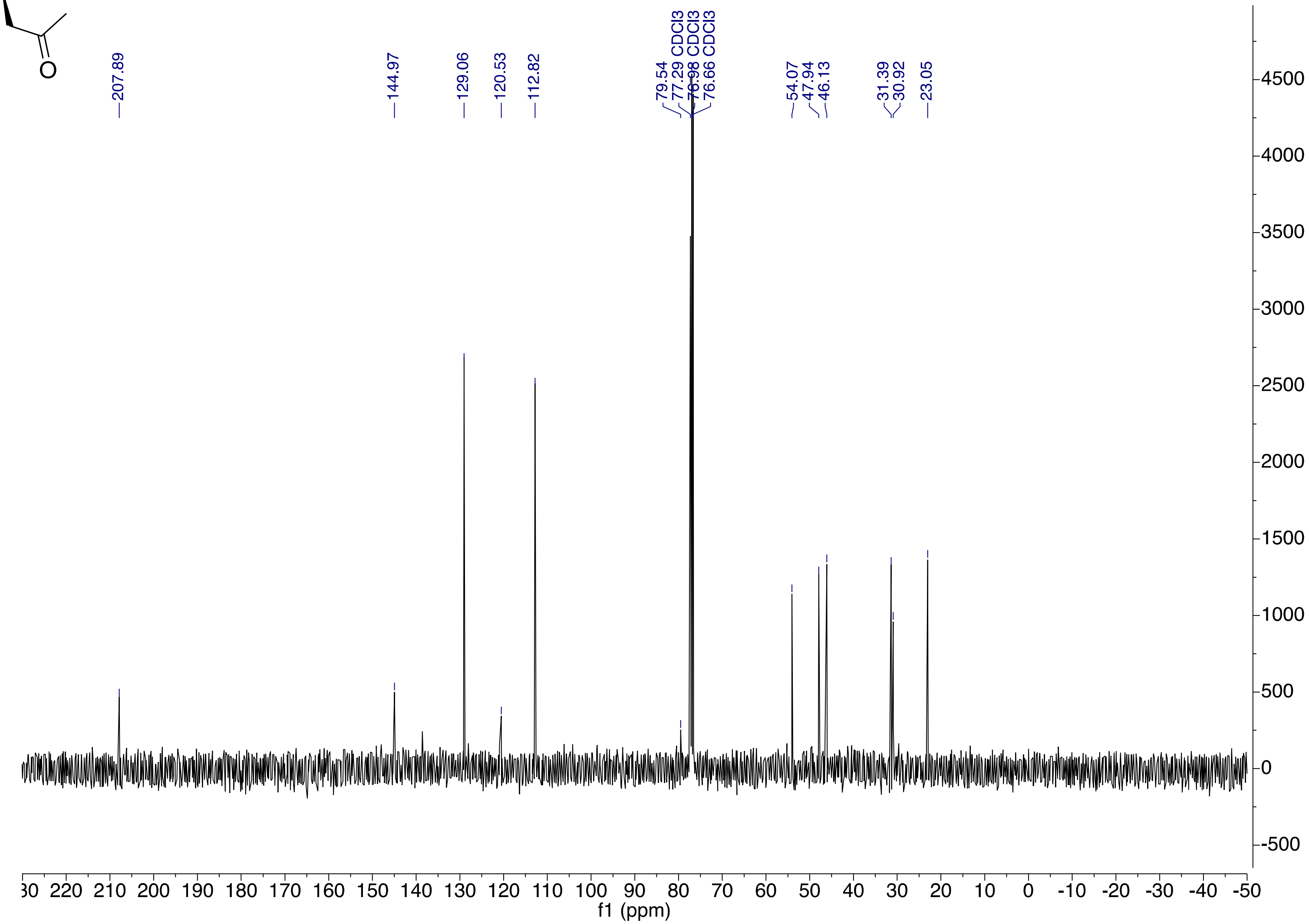

(*S*)-1-(1-(4-methoxyphenyl)pyrrolidin-2-yl)propan-2-one (3g)

^{1}H NMR (500 MHz, $CDCl_3$)

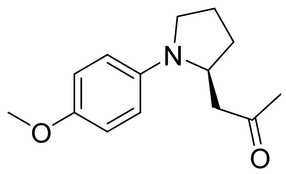

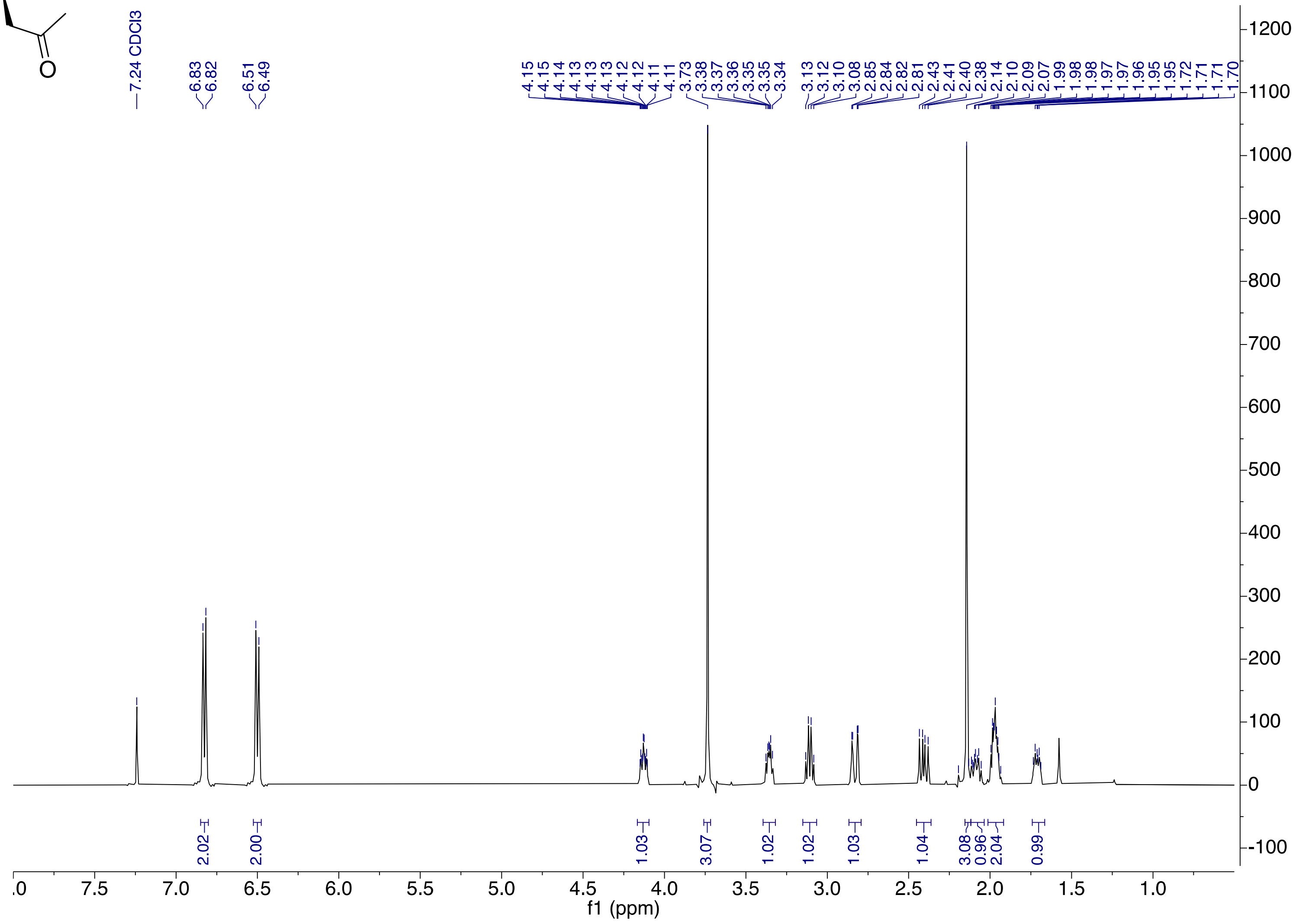

(*S*)-1-(1-(4-methoxyphenyl)pyrrolidin-2-yl)propan-2-one (3g)

^{13}C NMR (126 MHz, CDCl$_3$)

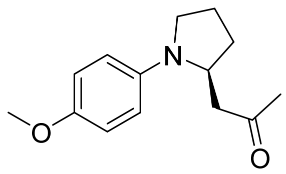

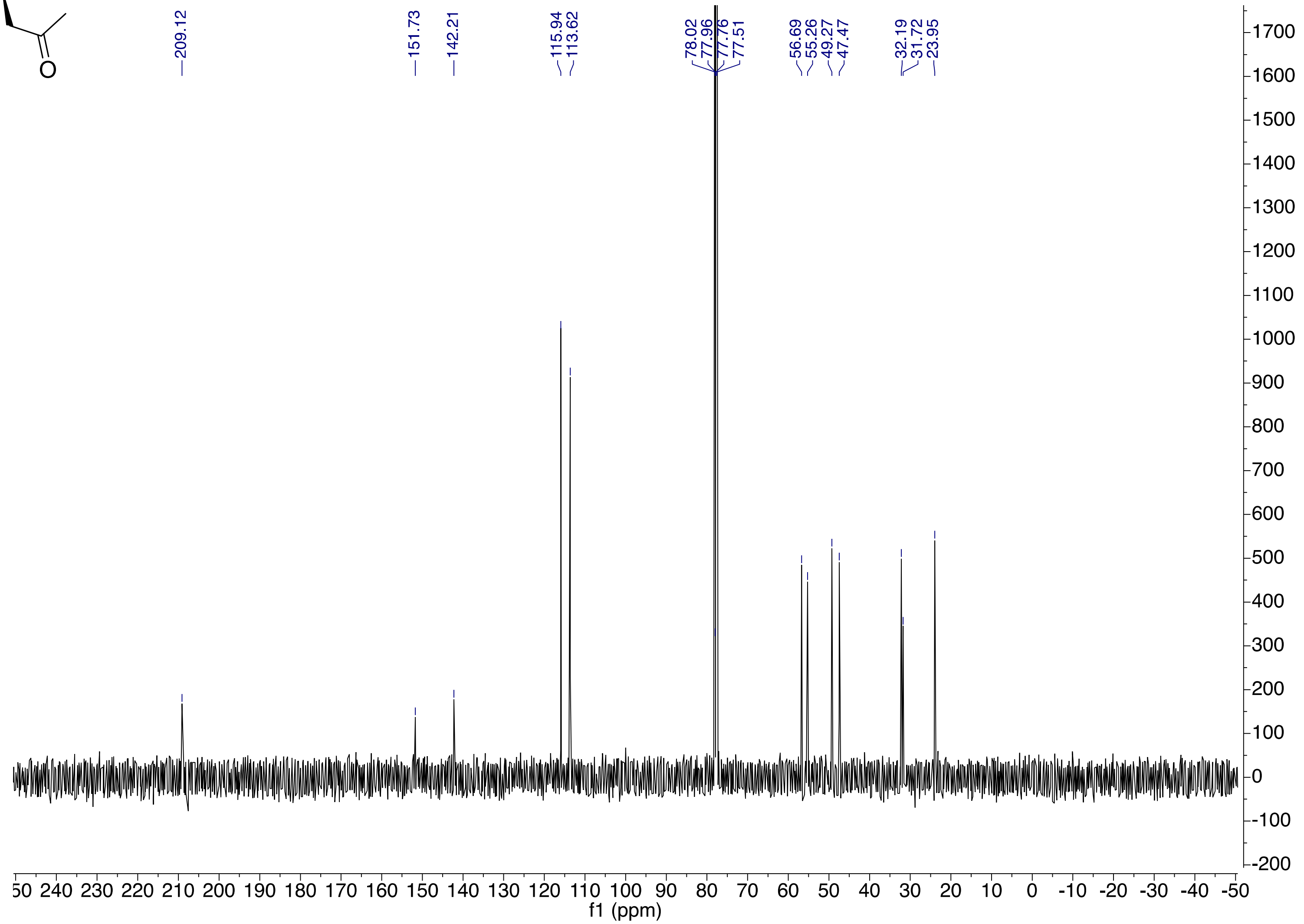

(*S*)-1-(1-(3-bromophenyl)pyrrolidin-2-yl)propan-2-one (3h)

^{1}H NMR (500 MHz, $CDCl_3$)

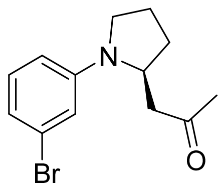

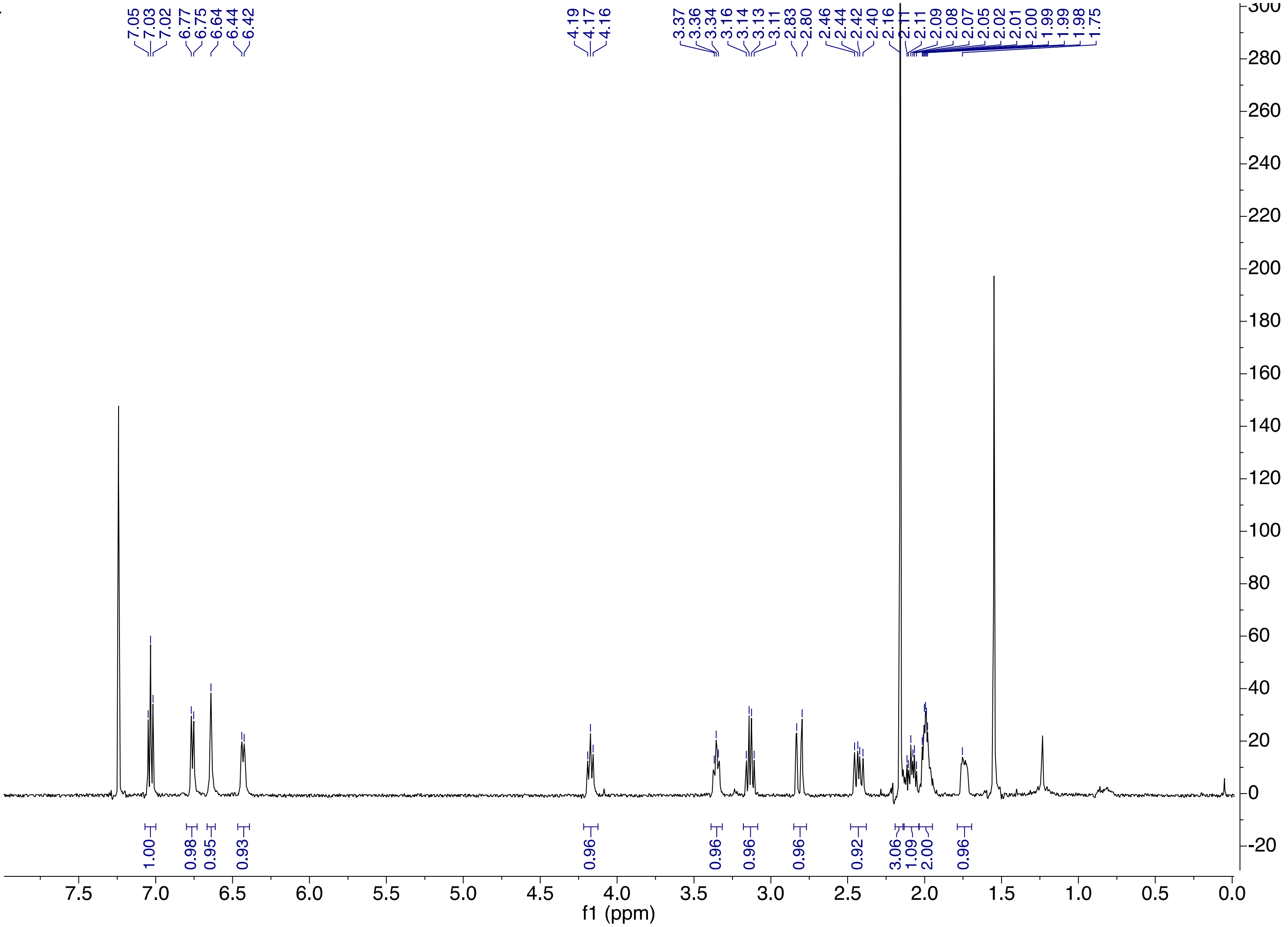

(*S*)-1-(1-(3-bromophenyl)pyrrolidin-2-yl)propan-2-one (3h)

^{13}C NMR (126 MHz, $CDCl_3$)

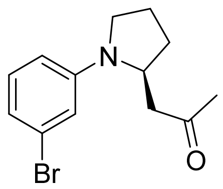

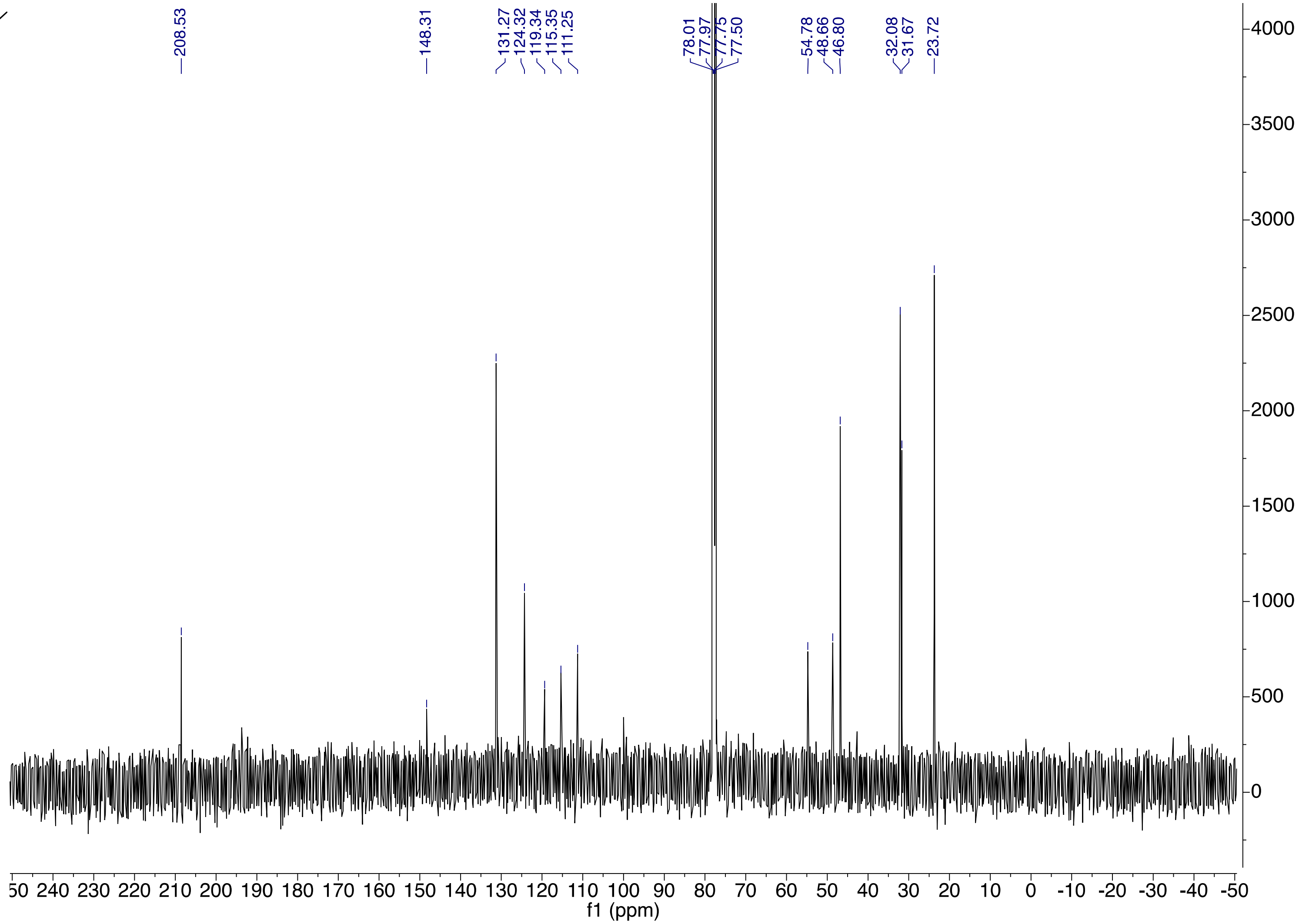

(*S*)-2-(2-(2-oxopropyl)pyrrolidin-1-yl)benzonitrile (3i)

^{1}H NMR (500 MHz, $CDCl_3$)

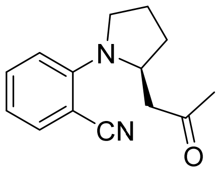

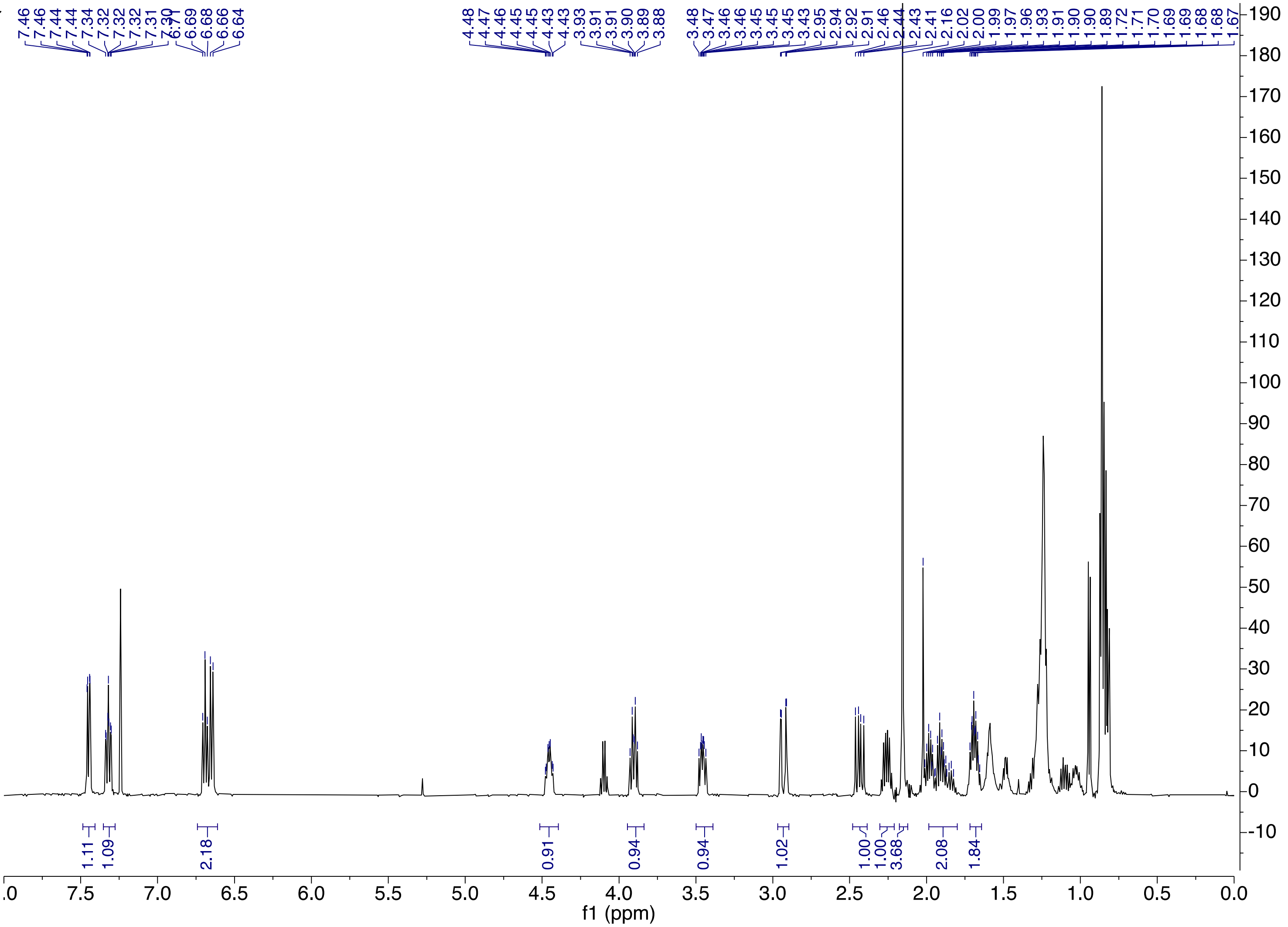

(*S*)-2-(2-(2-oxopropyl)pyrrolidin-1-yl)benzonitrile (3i)

^{13}C NMR (126 MHz, $CDCl_3$)

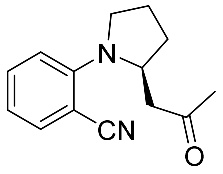

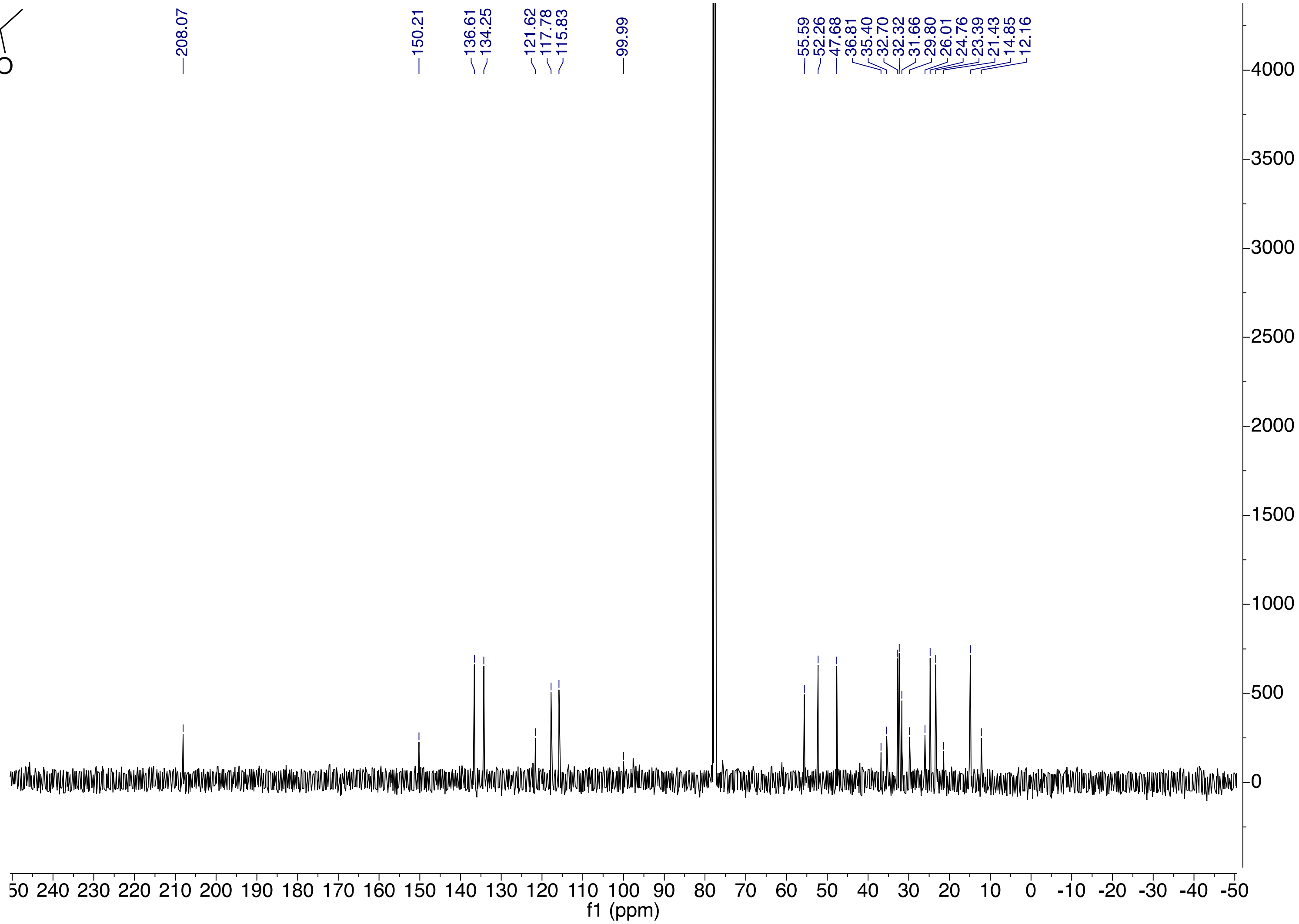

(*S*)-1-(1-(pyridin-2-yl)pyrrolidin-2-yl)propan-2-one (3j)

^{1}H NMR (500 MHz, $CDCl_3$)

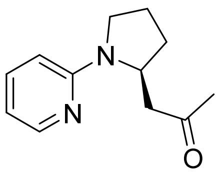

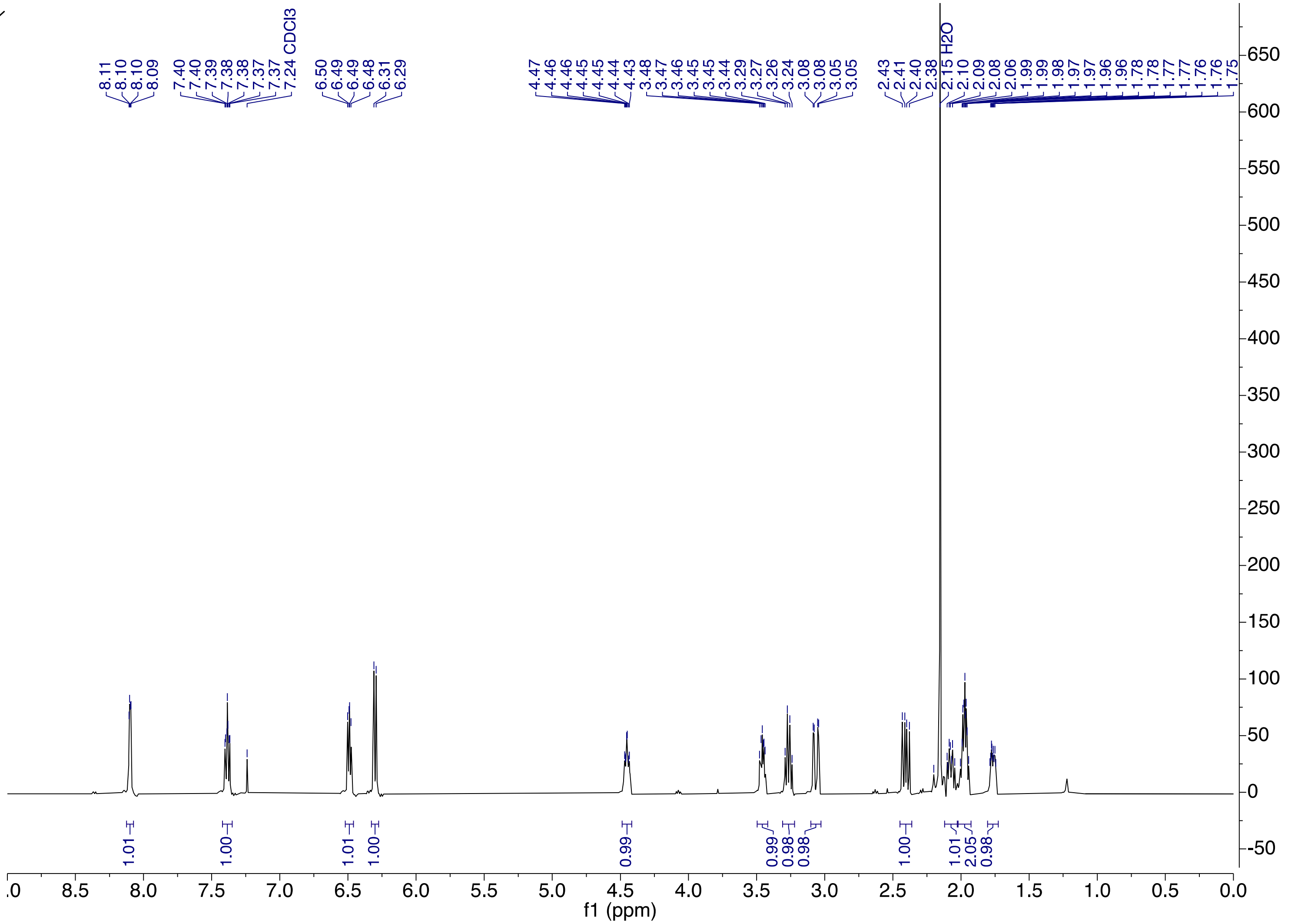

(*S*)-1-(1-(pyridin-2-yl)pyrrolidin-2-yl)propan-2-one (3j)

^{13}C NMR (126 MHz, $CDCl_3$)

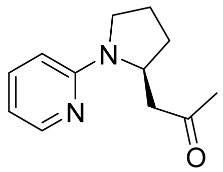

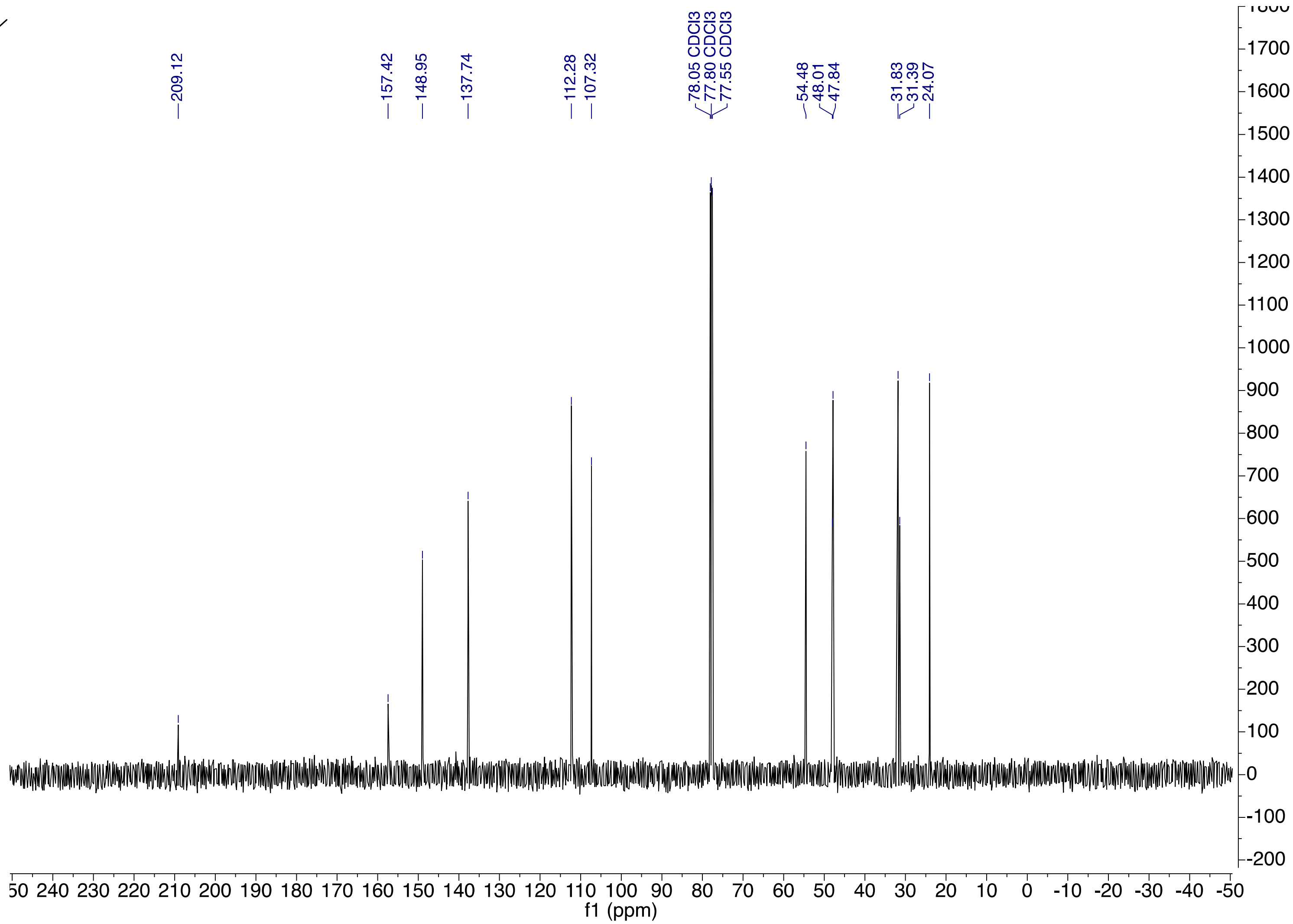

1-((2*S*)-5-methyl-1-phenylpyrrolidin-2-yl)propan-2-one (3k)

^{1}H NMR (500 MHz, $CDCl_3$)

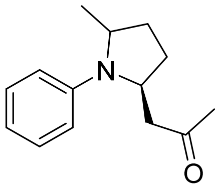

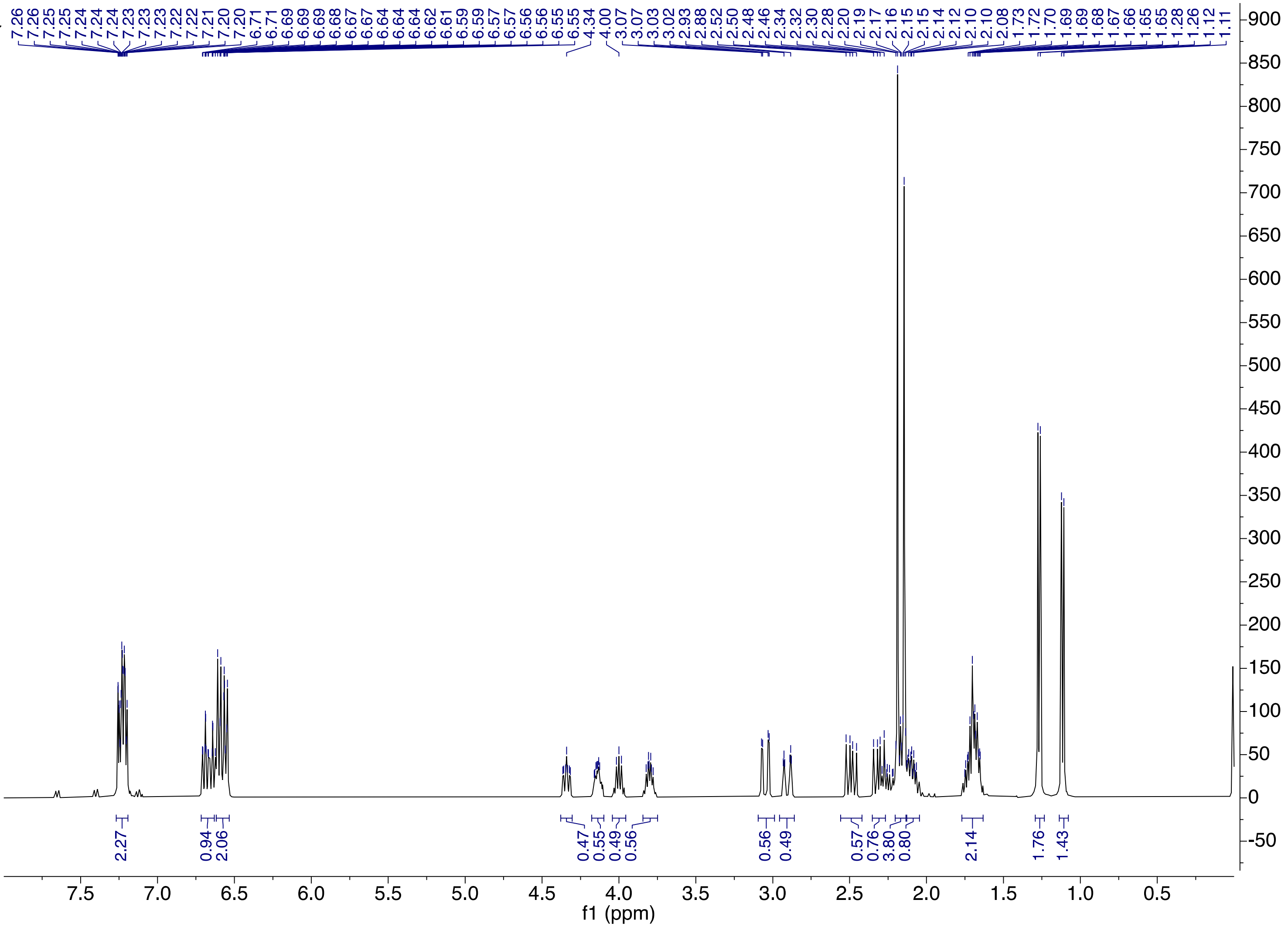

1-((2*S*)-5-methyl-1-phenylpyrrolidin-2-yl)propan-2-one (3k)

^{13}C NMR (126 MHz, $CDCl_3$)

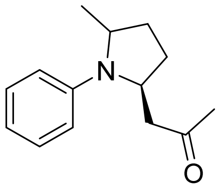

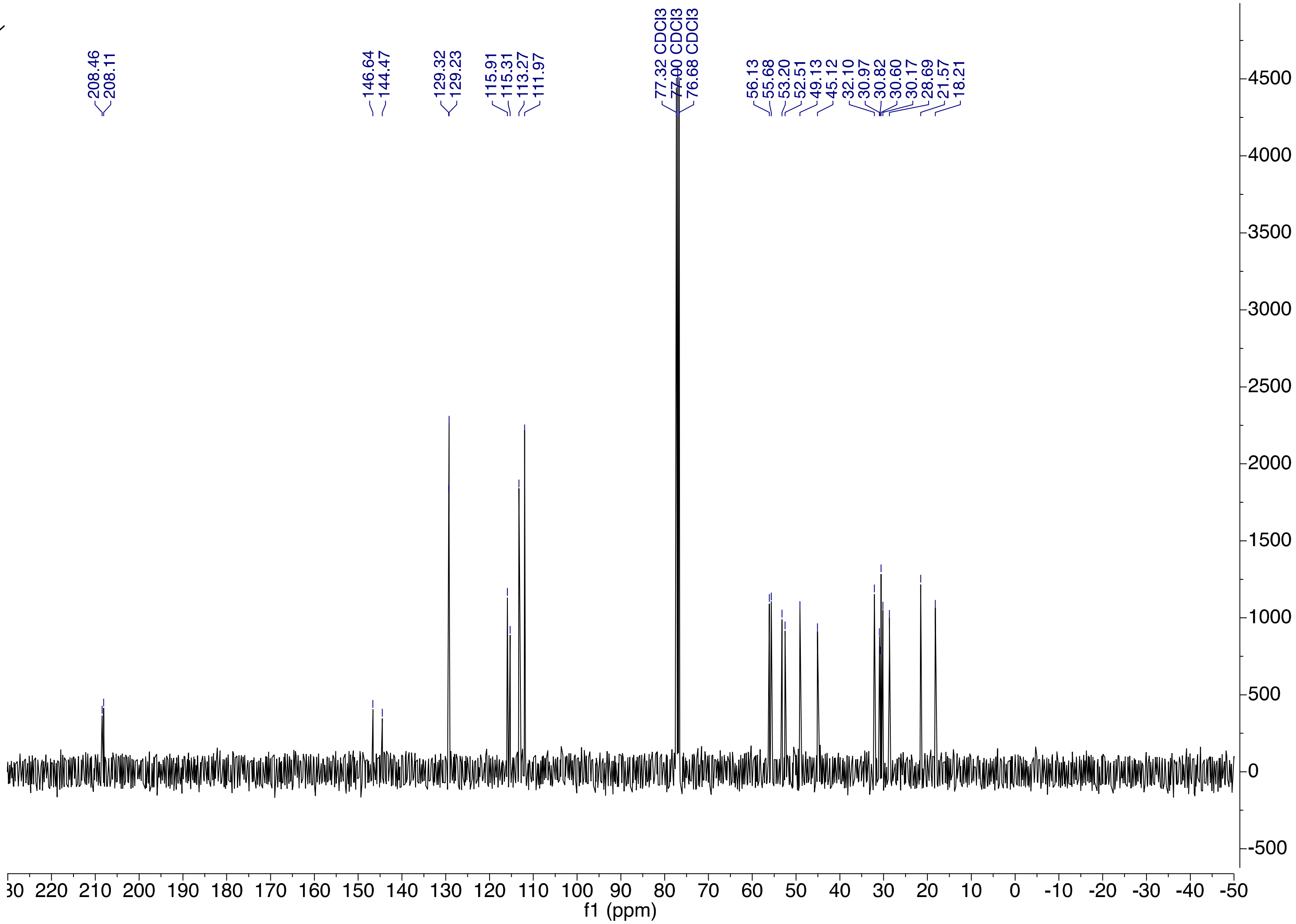

1-(1,3-dihydroisobenzofuran-1-yl)propan-2-one (3l)

^{1}H NMR (500 MHz, $CDCl_3$)

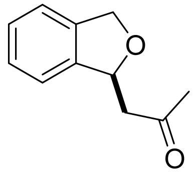

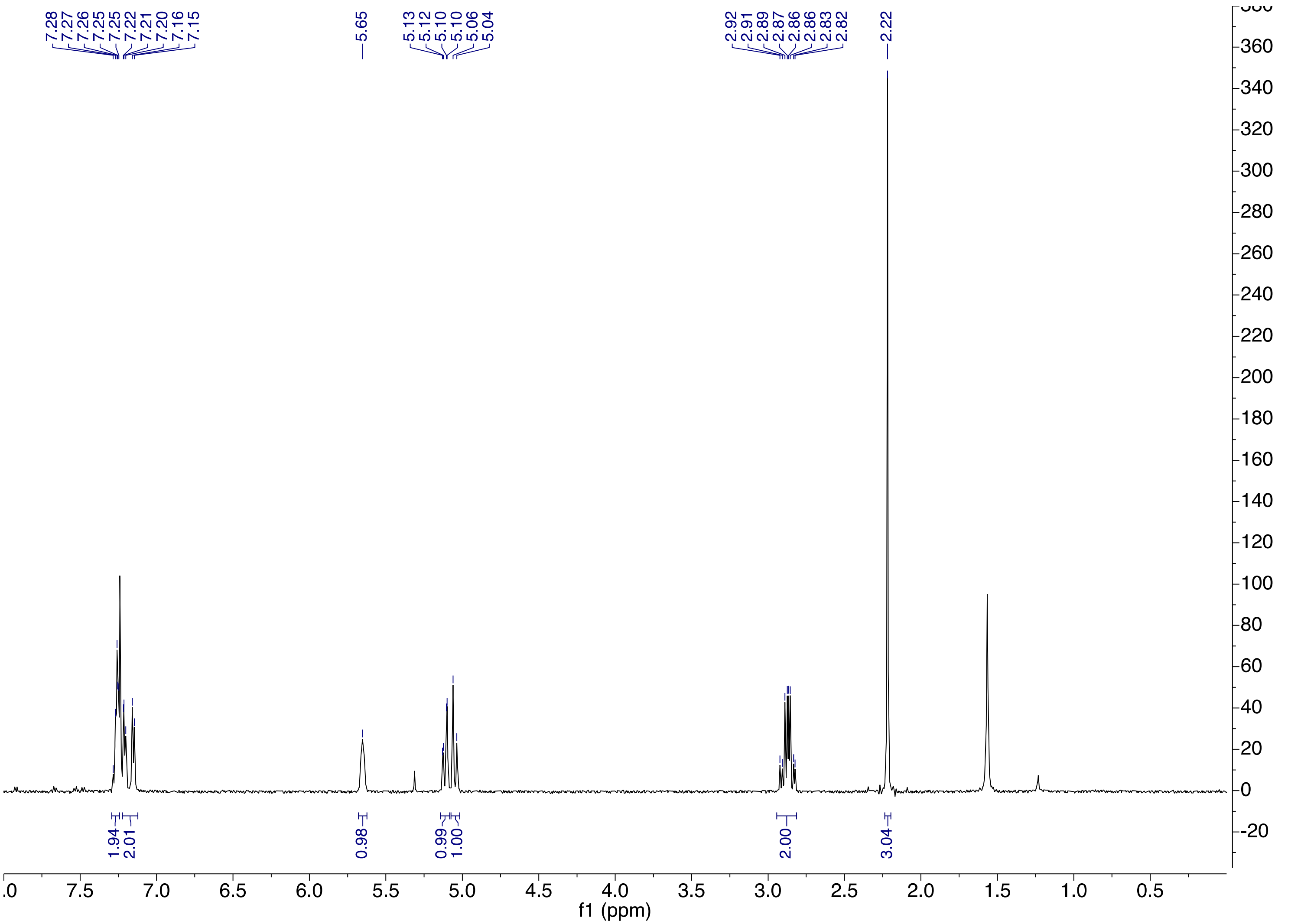

1-(1,3-dihydroisobenzofuran-1-yl)propan-2-one (3l)

^{13}C NMR (126 MHz, $CDCl_3$)

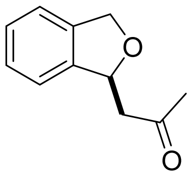

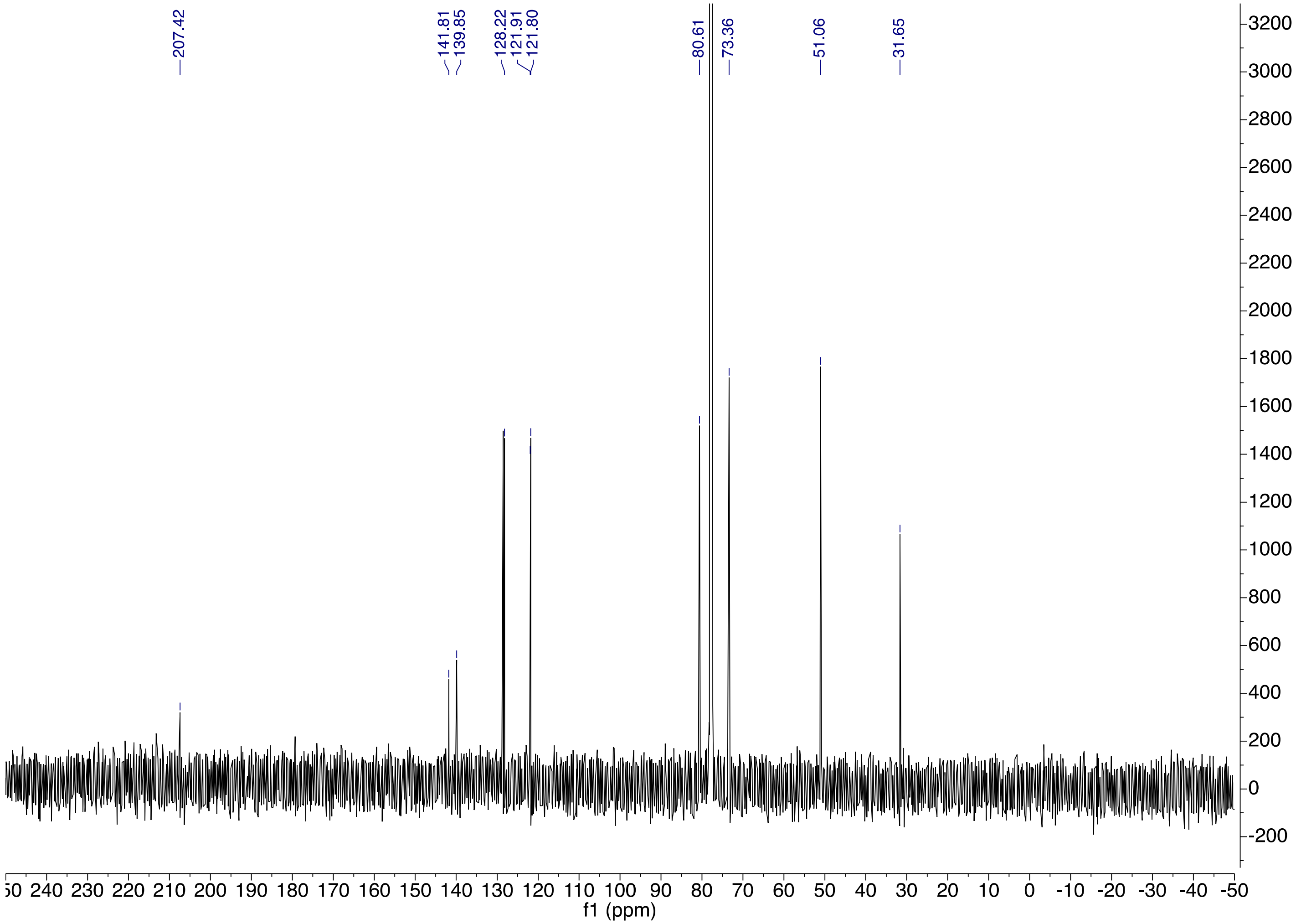

(*S*)-1-(1-phenylpiperidin-2-yl)propan-2-one (3m)

^{1}H NMR (500 MHz, $CDCl_3$)

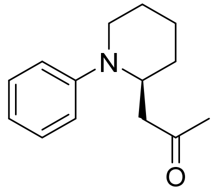

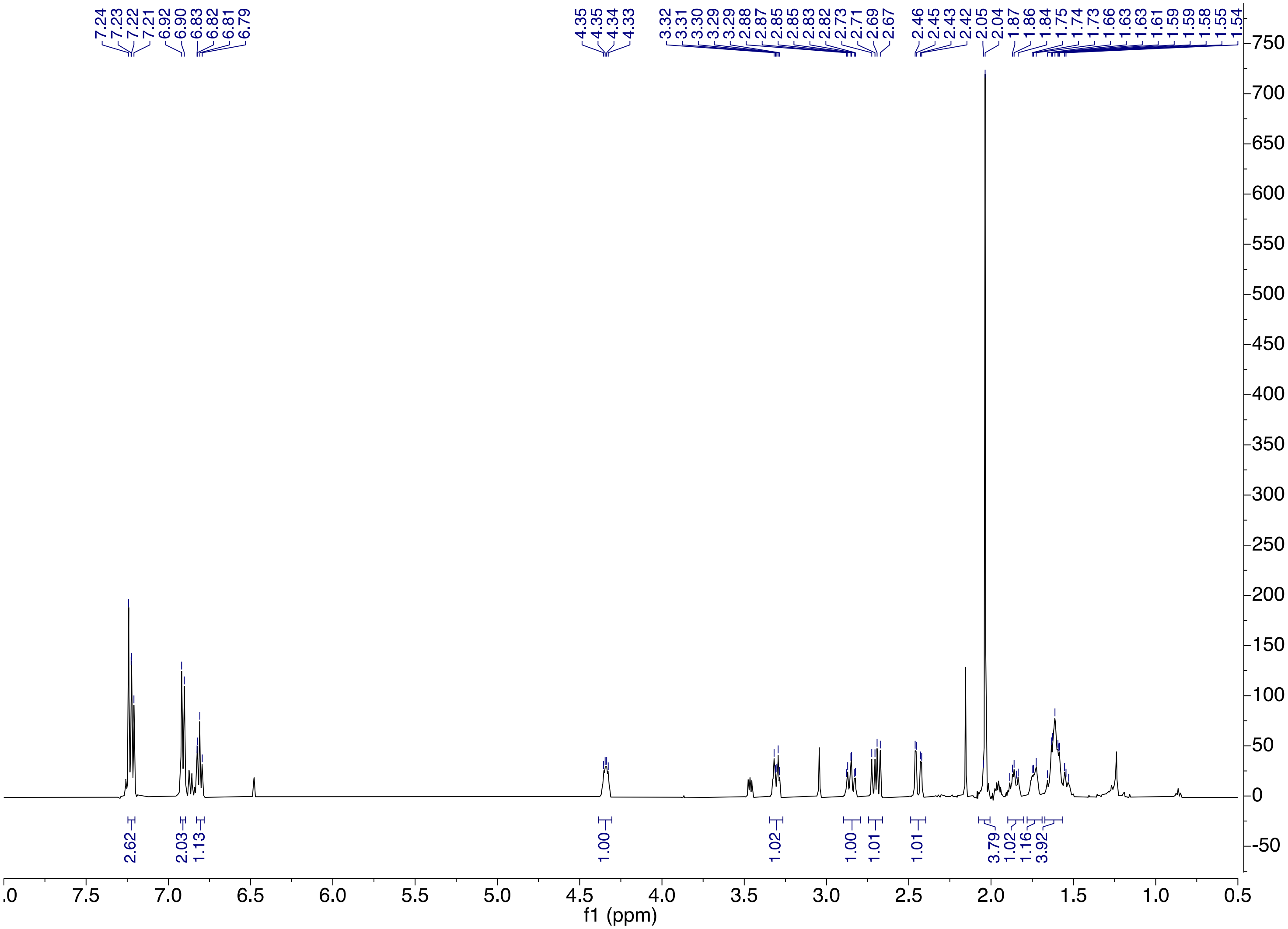

(*S*)-1-(1-phenylpiperidin-2-yl)propan-2-one (3m)

^{13}C NMR (126 MHz, $CDCl_3$)

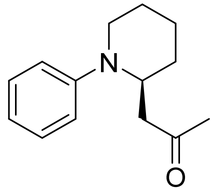

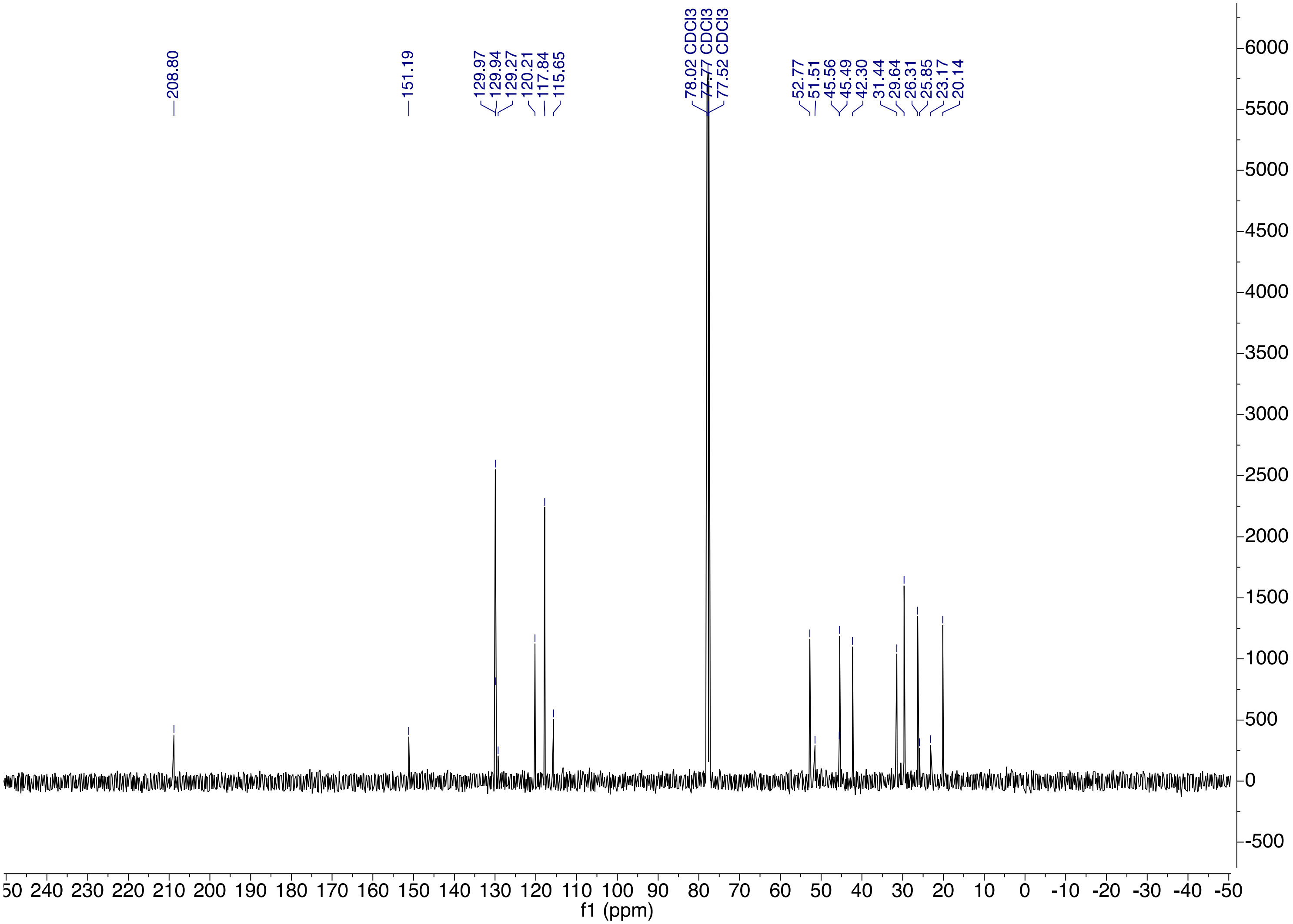

1,1'-(1-phenylpyrrolidine-2,5-diyl)bis(propan-2-one) (5a)

^{1}H NMR (500 MHz, $CDCl_3$)

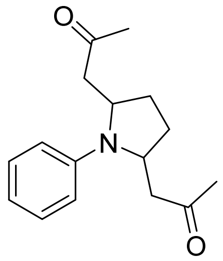

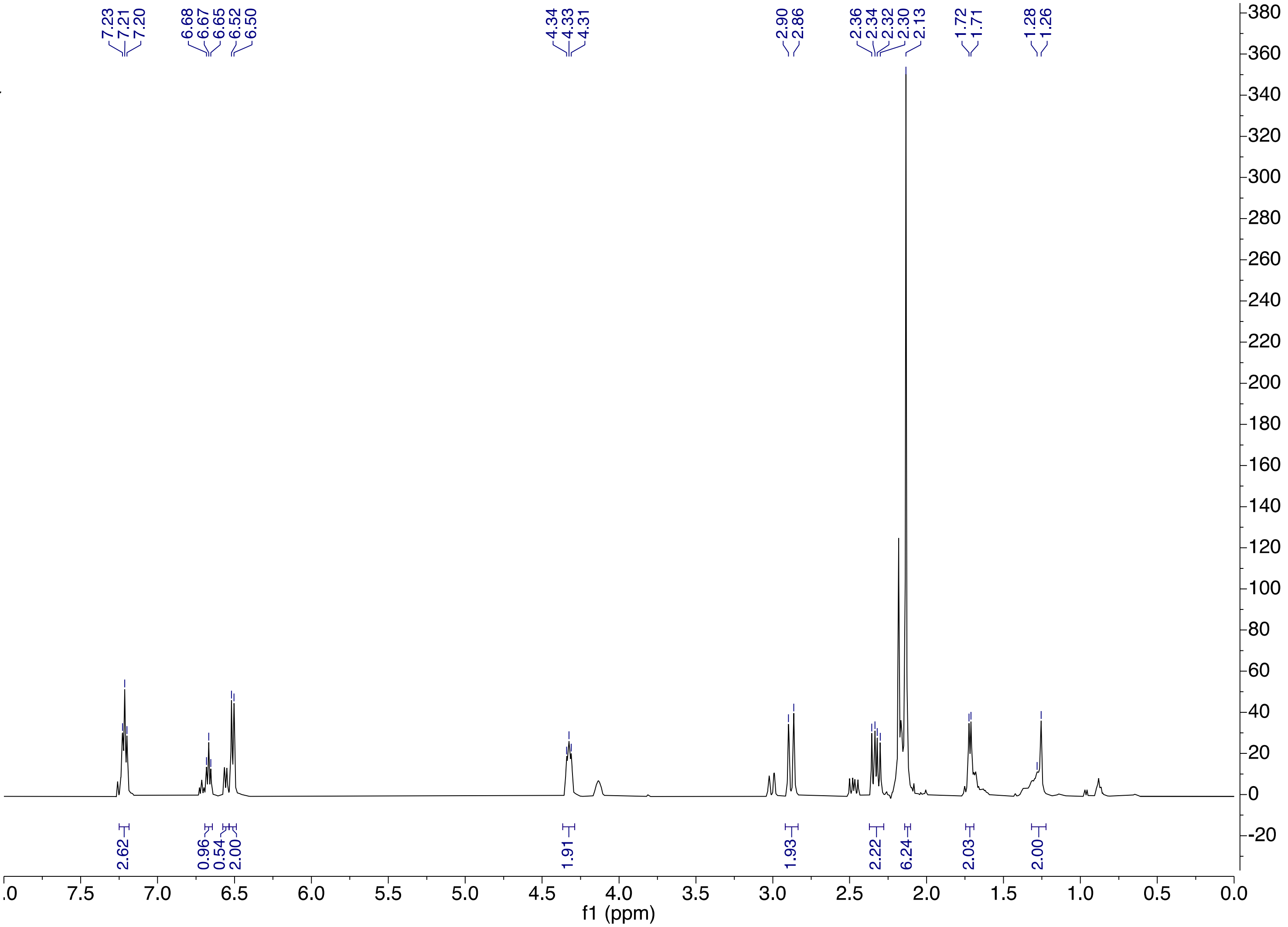

1,1'-(1-phenylpyrrolidine-2,5-diyl)bis(propan-2-one) (5a)

^{13}C NMR (126 MHz, $CDCl_3$)

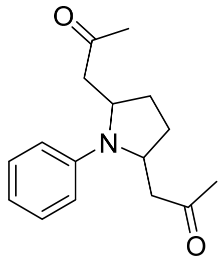

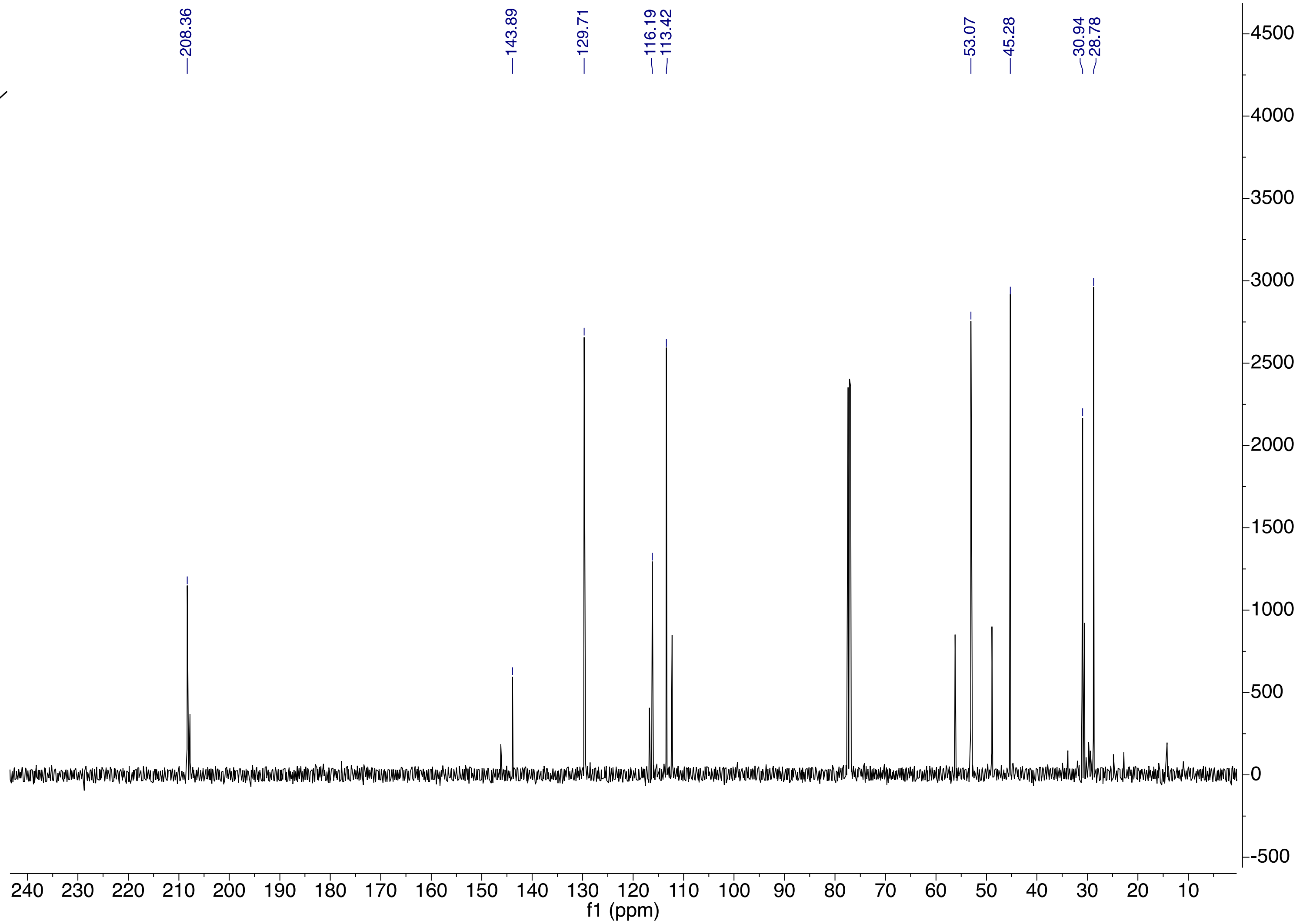

Ethyl (*S*)-2-(1-phenylpyrrolidin-2-yl)acetate (6a)

^{1}H NMR (500 MHz, $CDCl_3$)

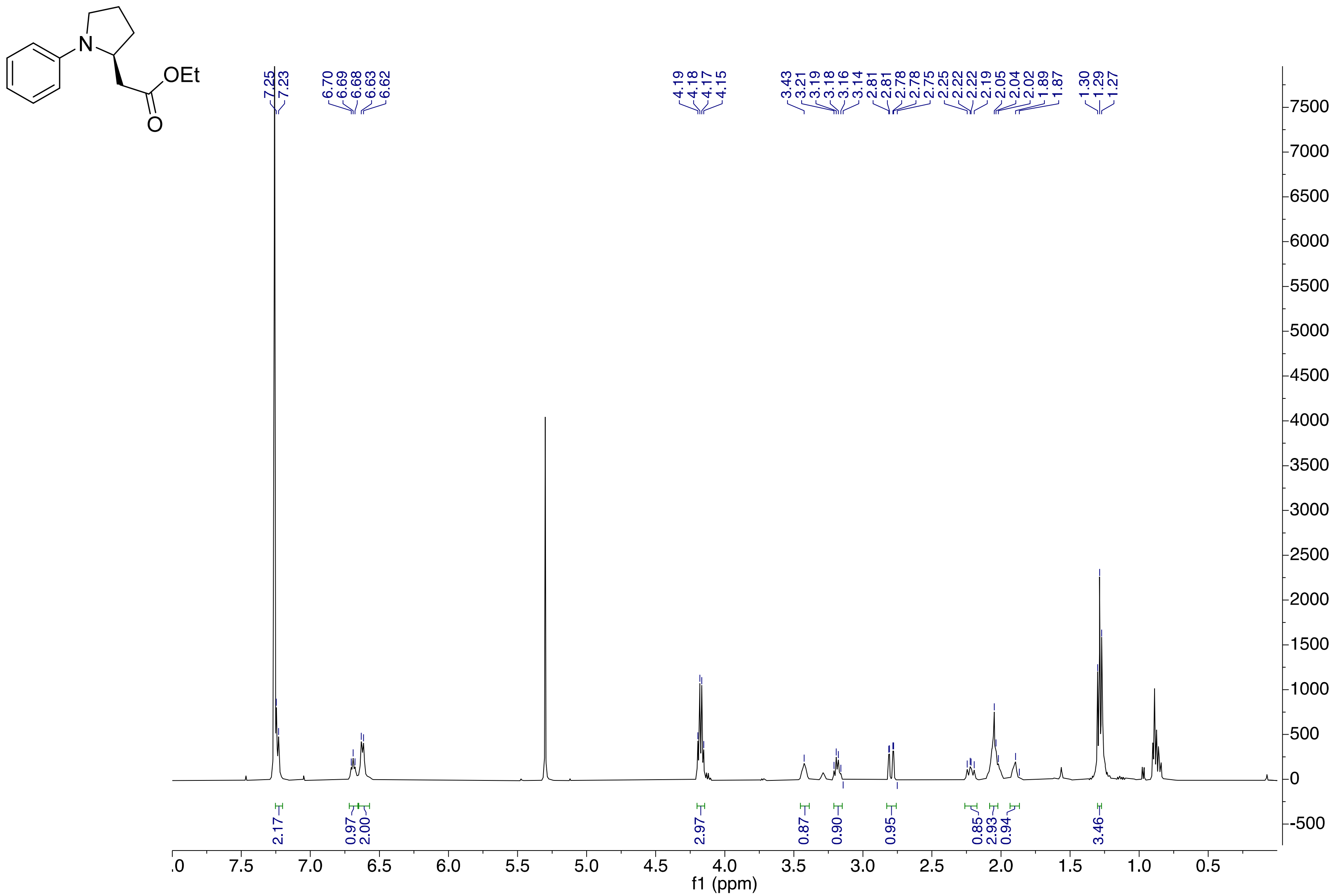

Ethyl (*S*)-2-(1-phenylpyrrolidin-2-yl)acetate (6a)

^{13}C NMR (126 MHz, $CDCl_3$)

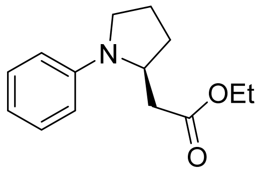

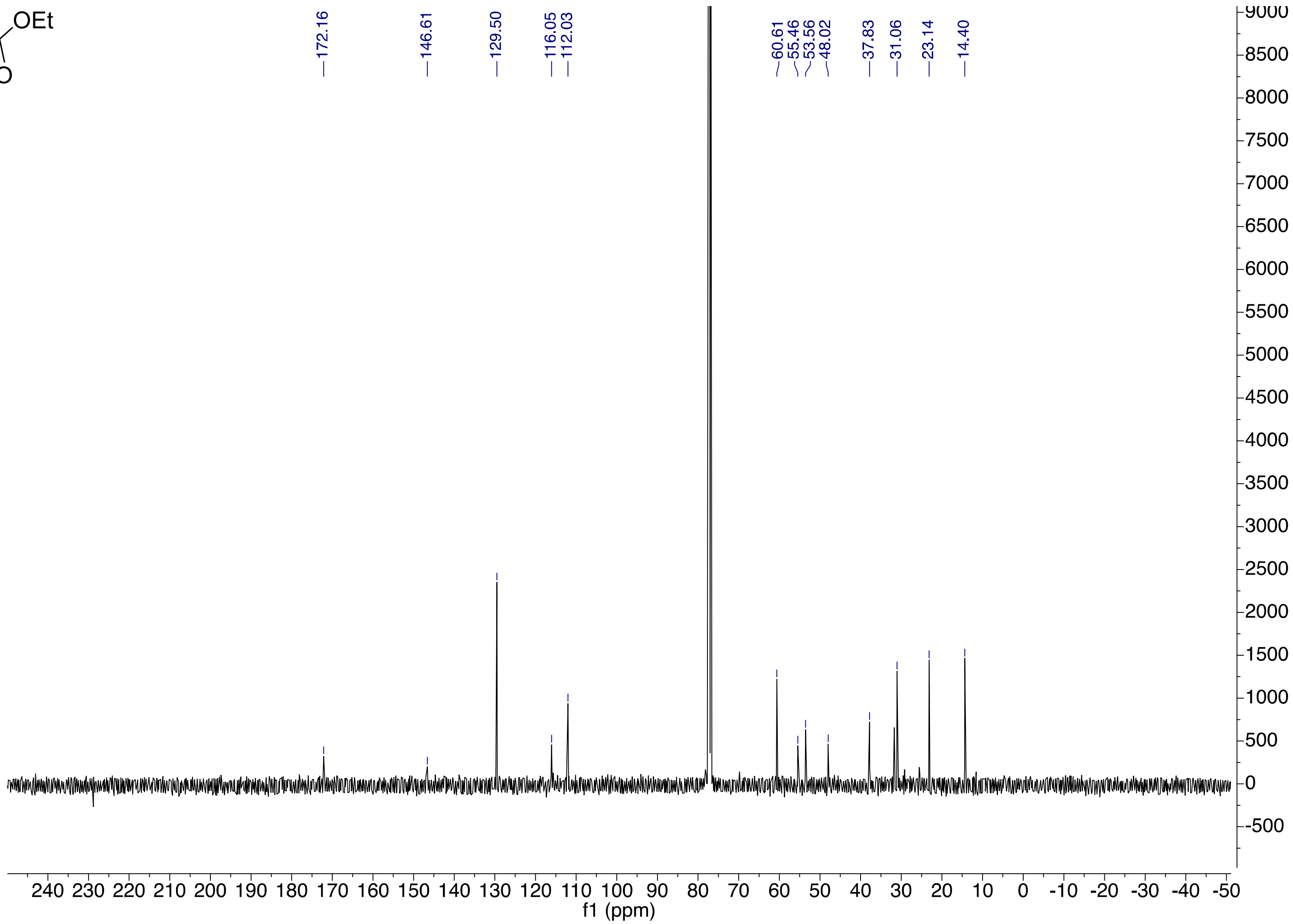

Ethyl 2-(1-(p-tolyl)pyrrolidin-2-yl)acetate (6b/7b)

^{1}H NMR (500 MHz, $CDCl_3$)

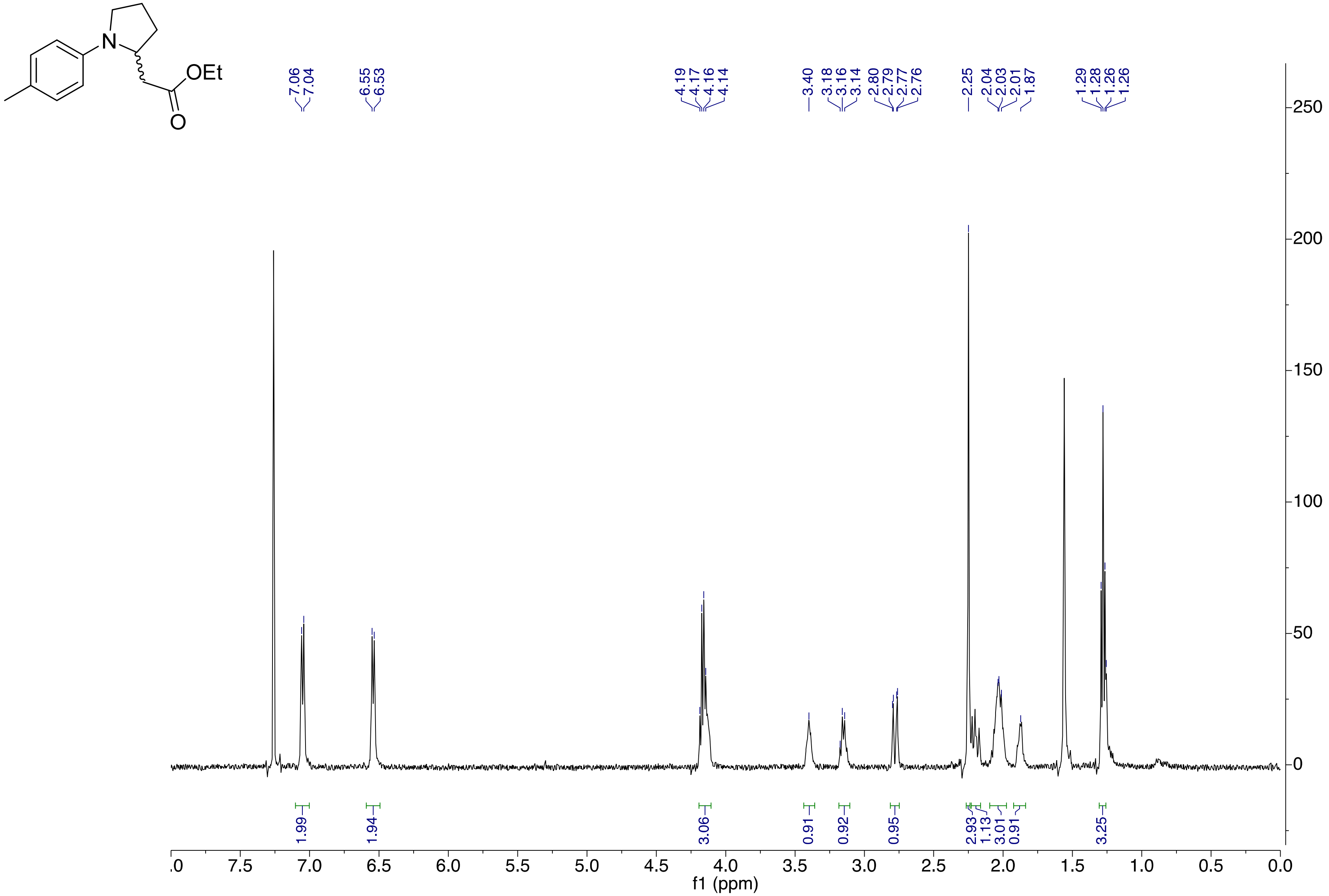

Ethyl 2-(1-(p-tolyl)pyrrolidin-2-yl)acetate (6b/7b)

^{13}C NMR (126 MHz, $CDCl_3$)

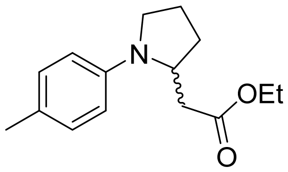

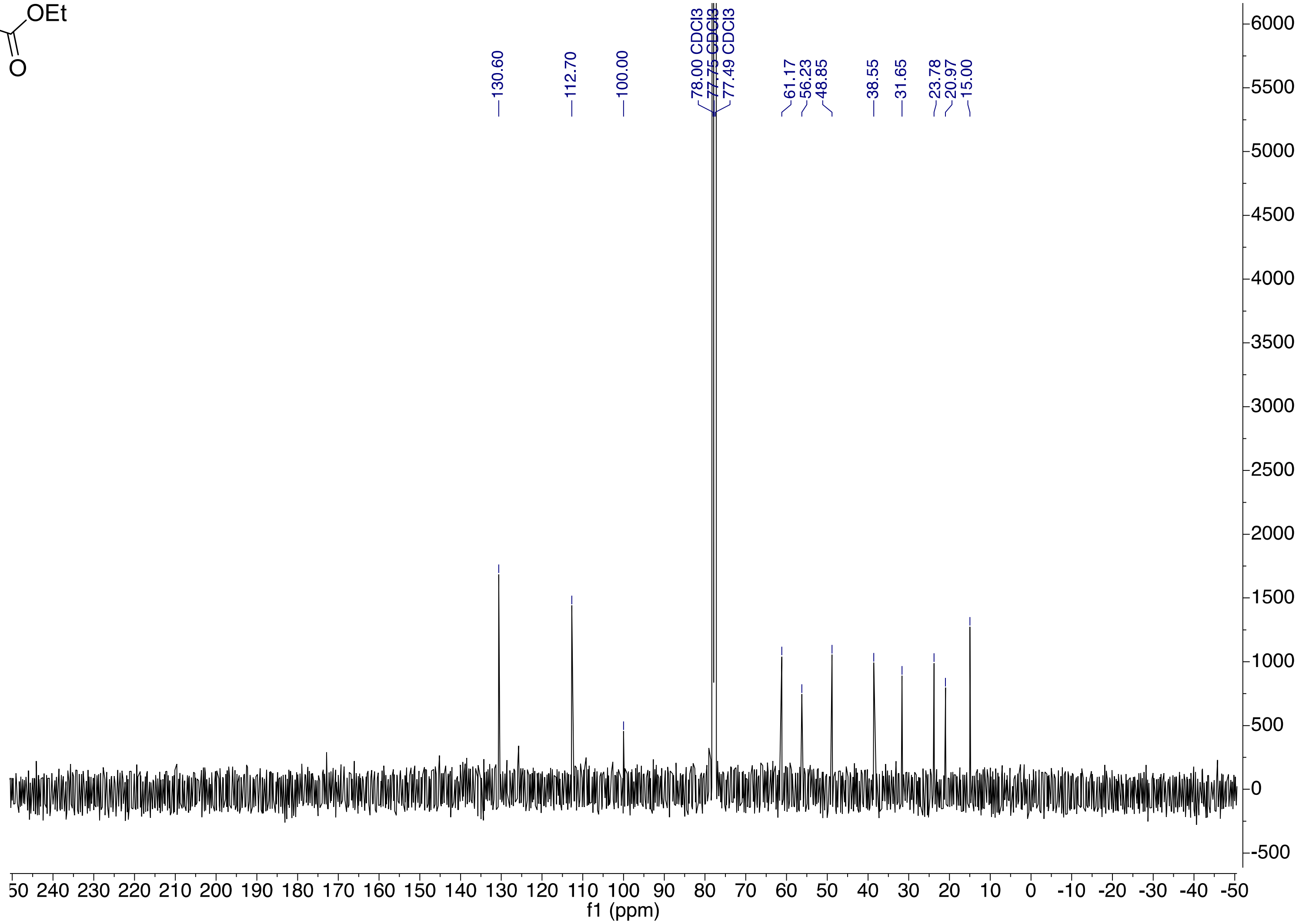

Ethyl (*S*)-2-(1-(m-tolyl)pyrrolidin-2-yl)acetate (6c)

^{1}H NMR (500 MHz, $CDCl_3$)

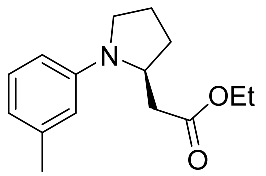

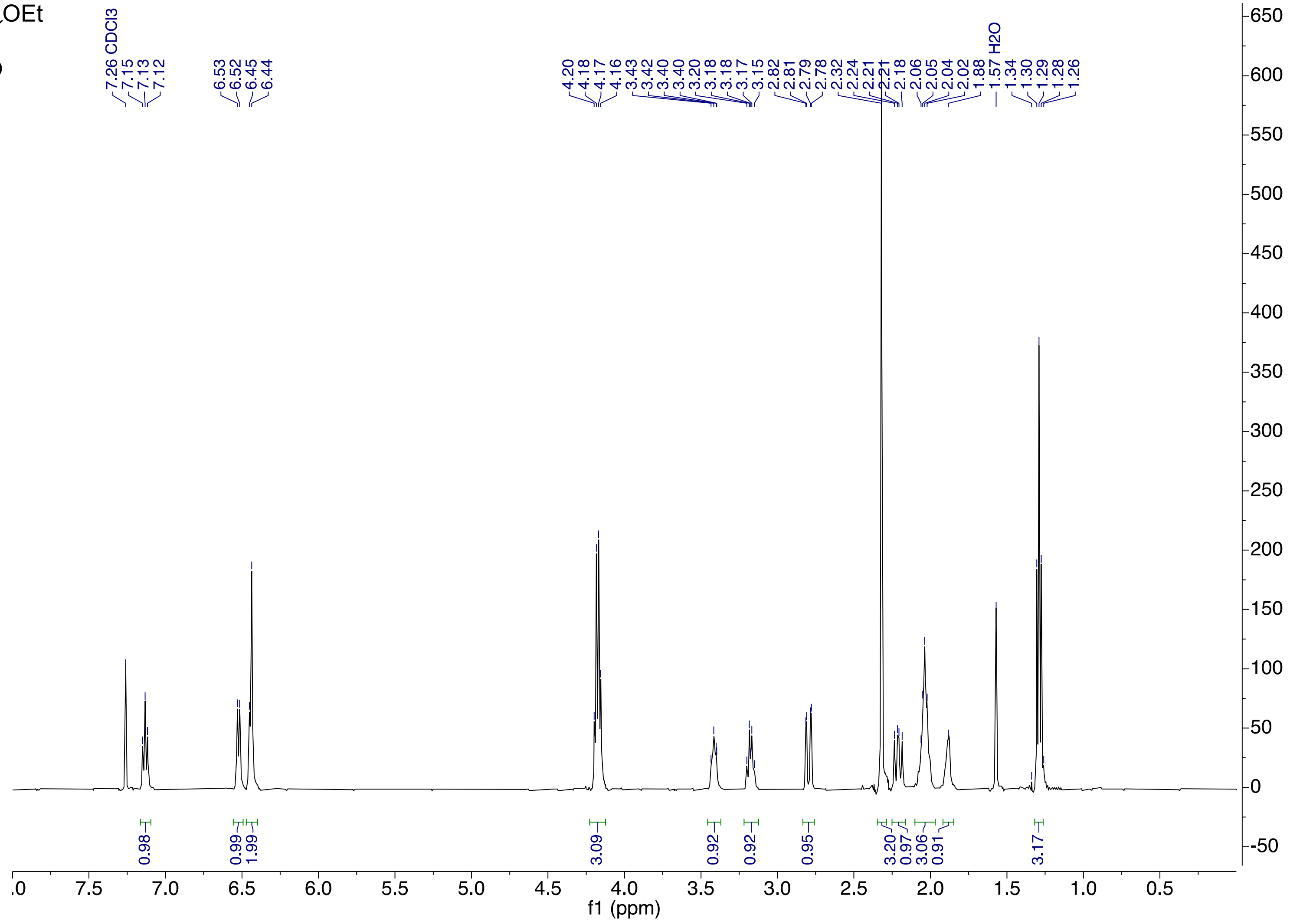

Ethyl (*S*)-2-(1-(m-tolyl)pyrrolidin-2-yl)acetate (6c)

^{13}C NMR (126 MHz, $CDCl_3$)

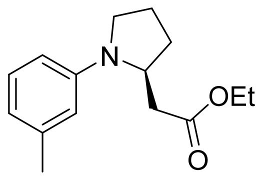

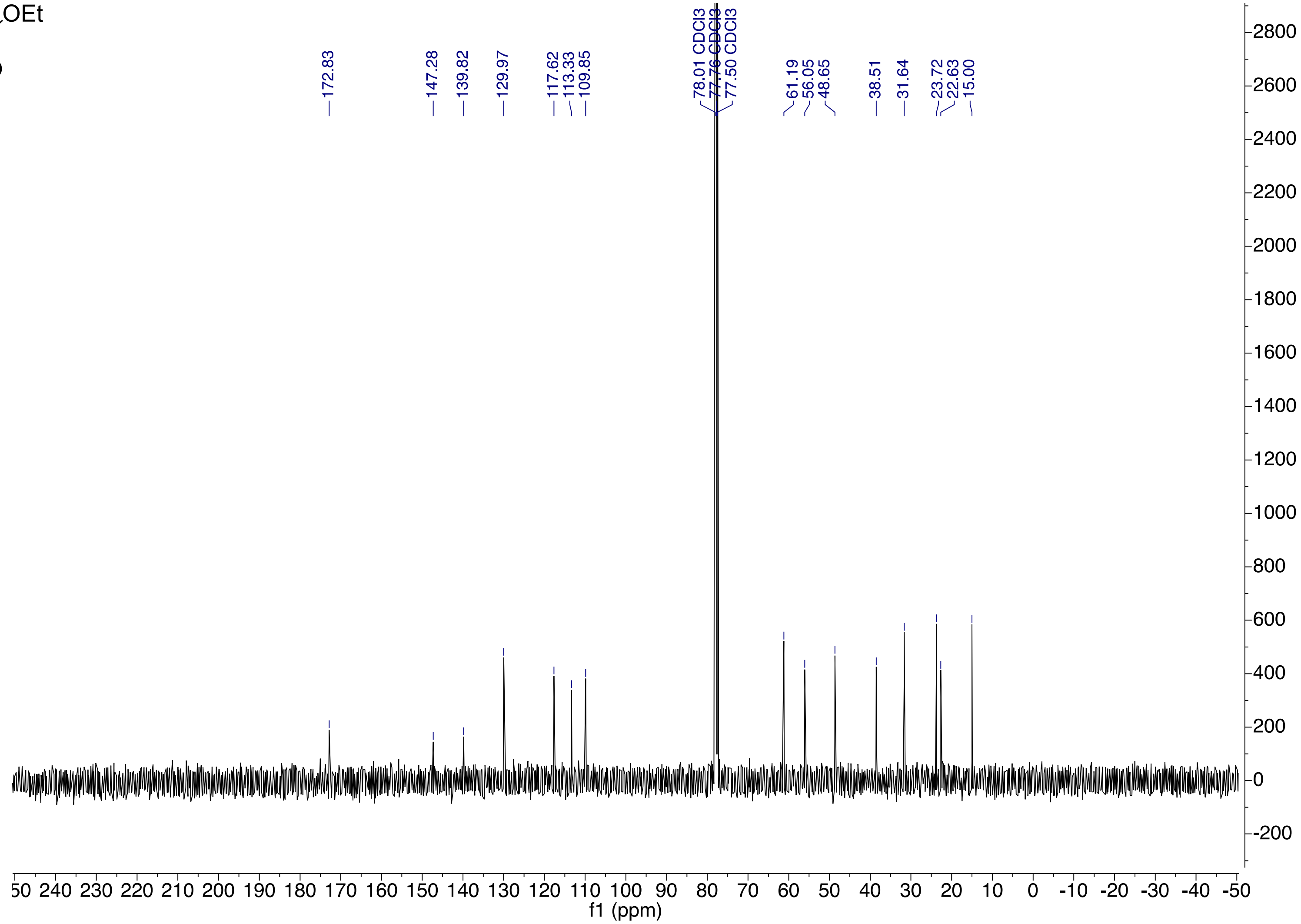

Ethyl (*S*)-2-(1-(o-tolyl)pyrrolidin-2-yl)acetate (6d)

^{1}H NMR (500 MHz, $CDCl_3$)

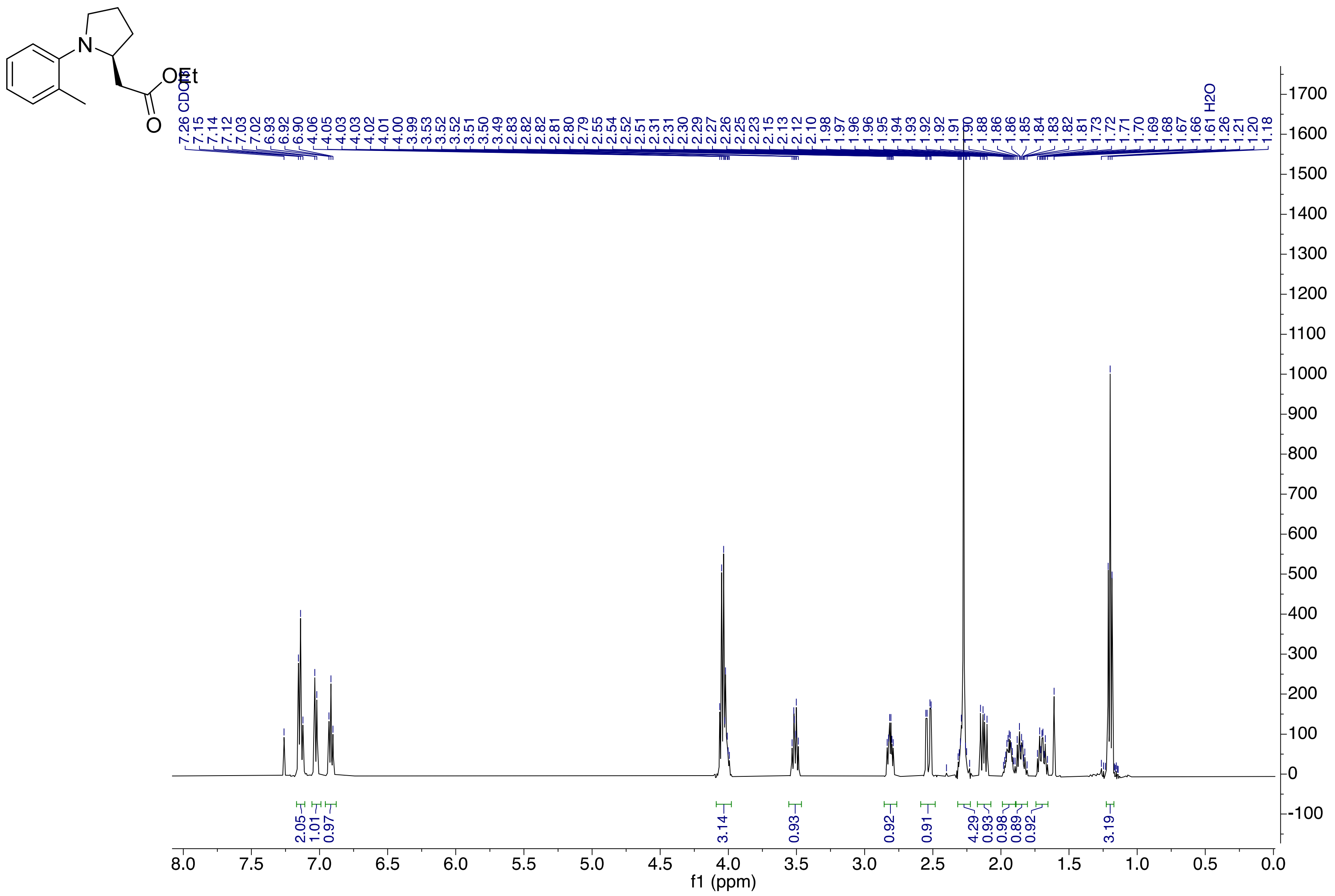

Ethyl (*S*)-2-(1-(o-tolyl)pyrrolidin-2-yl)acetate (6d)

^{13}C NMR (126 MHz, $CDCl_3$)

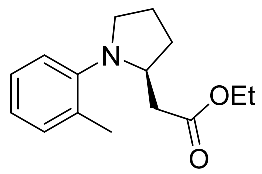

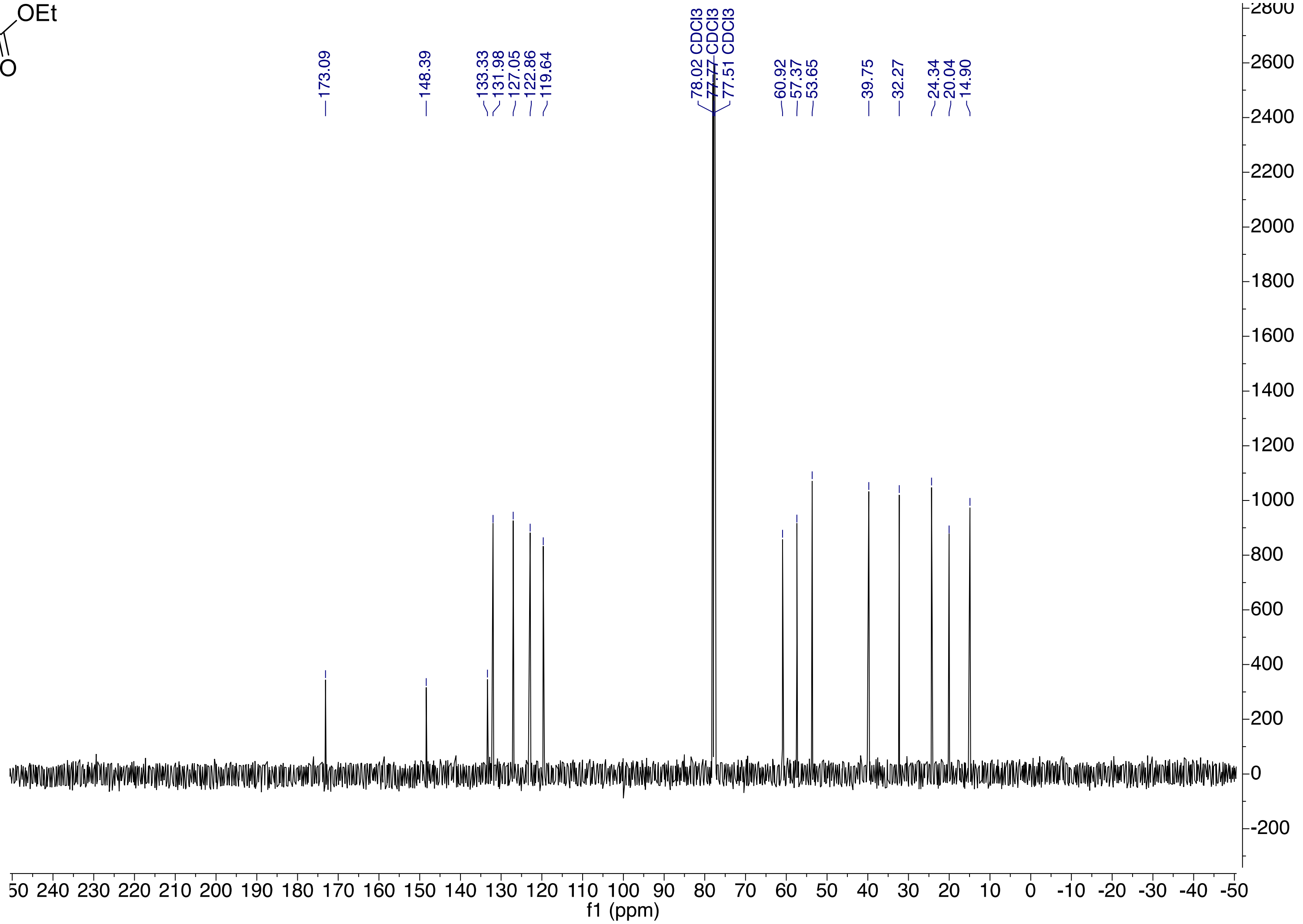

Ethyl (*S*)-2-(1-(4-fluorophenyl)pyrrolidin-2-yl)acetate (6e)

^{1}H NMR (500 MHz, $CDCl_3$)

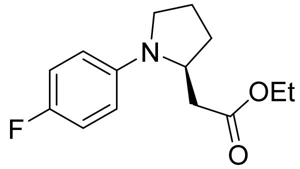

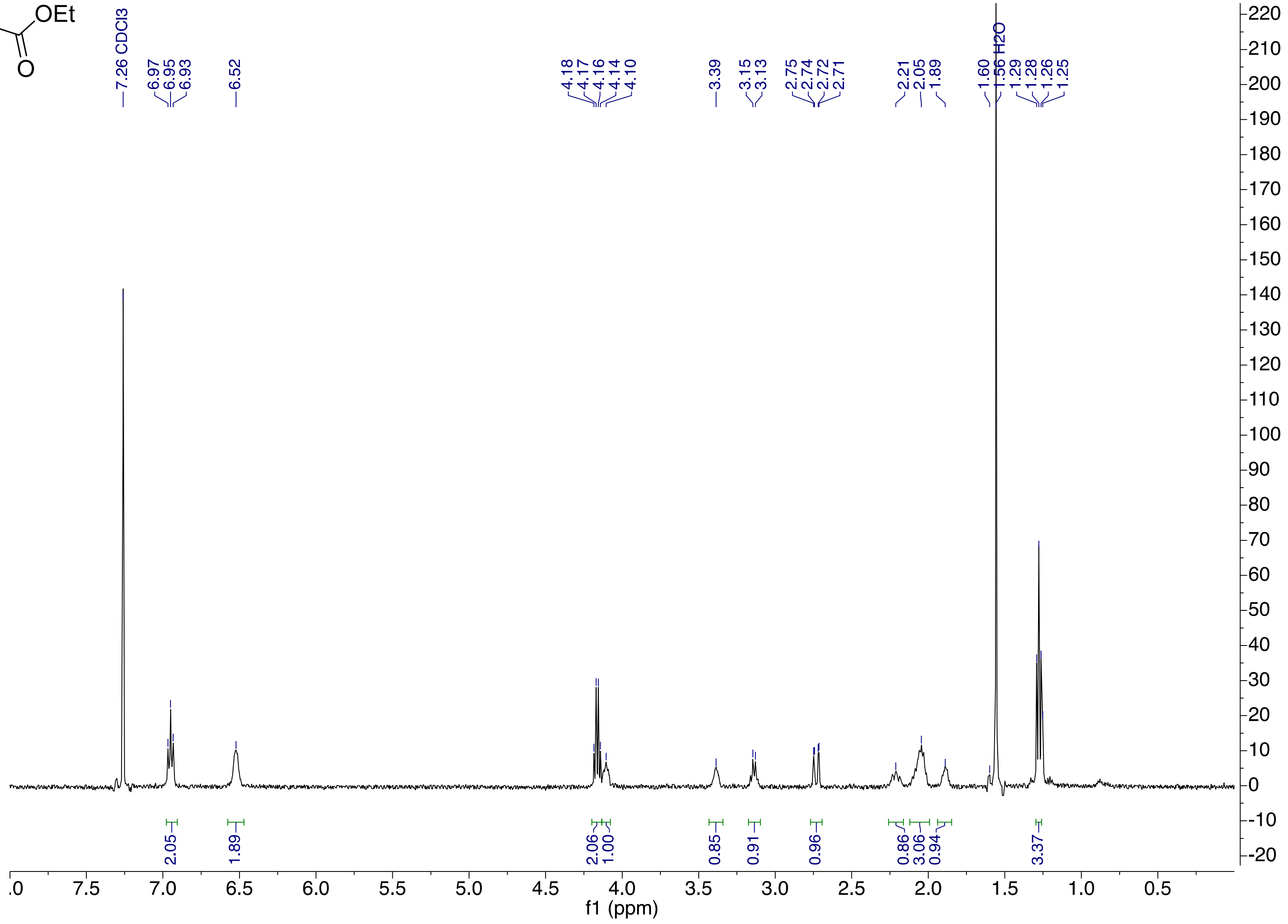

Ethyl (*S*)-2-(1-(4-fluorophenyl)pyrrolidin-2-yl)acetate (6e)

^{13}C NMR (126 MHz, $CDCl_3$)

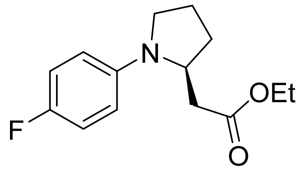

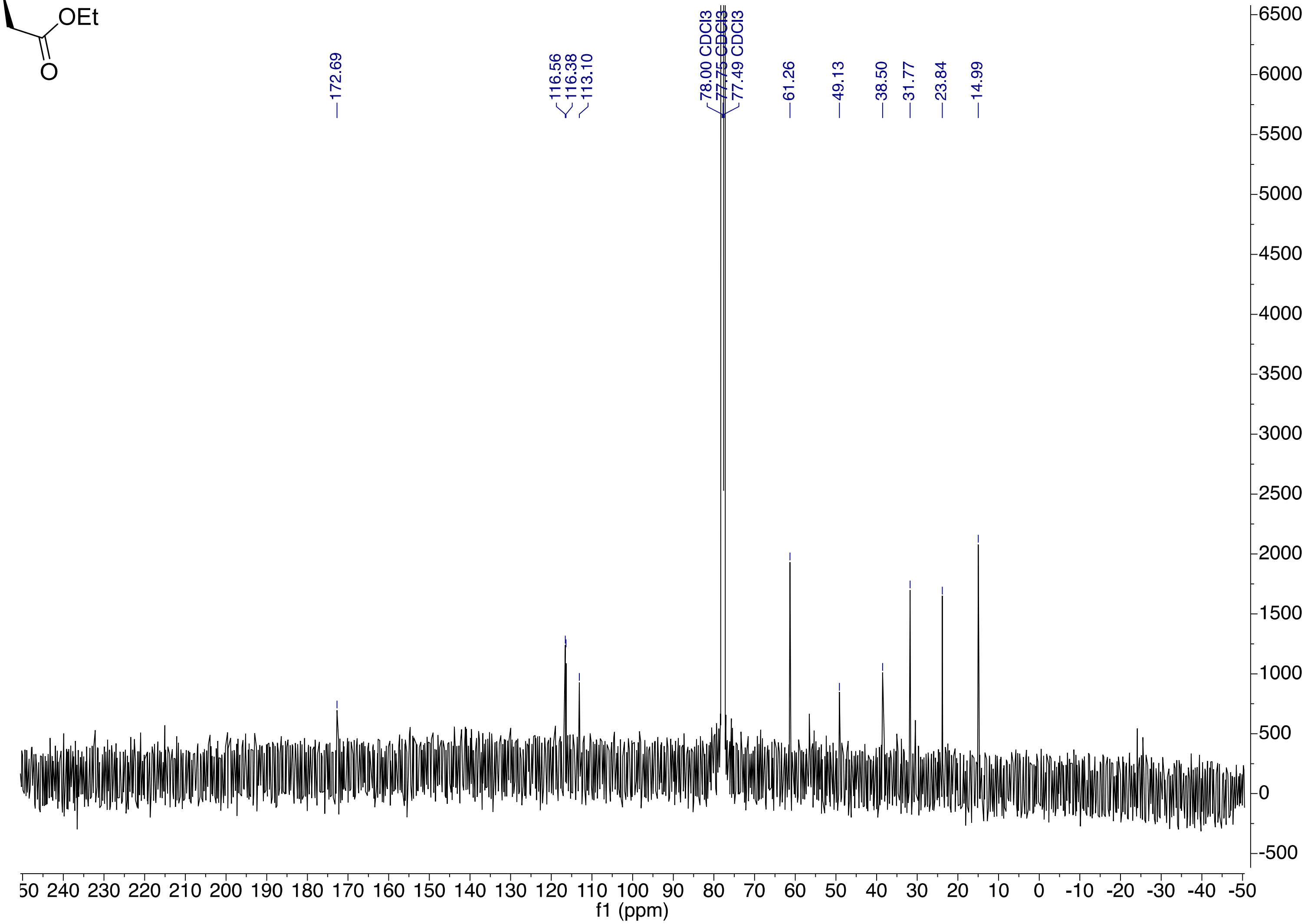

Ethyl (*S*)-2-(1-(4-fluorophenyl)pyrrolidin-2-yl)acetate (6e)

^{19}F NMR (376 MHz, $CDCl_3$)

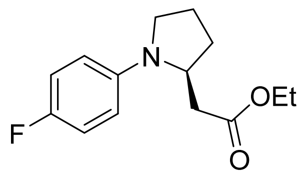

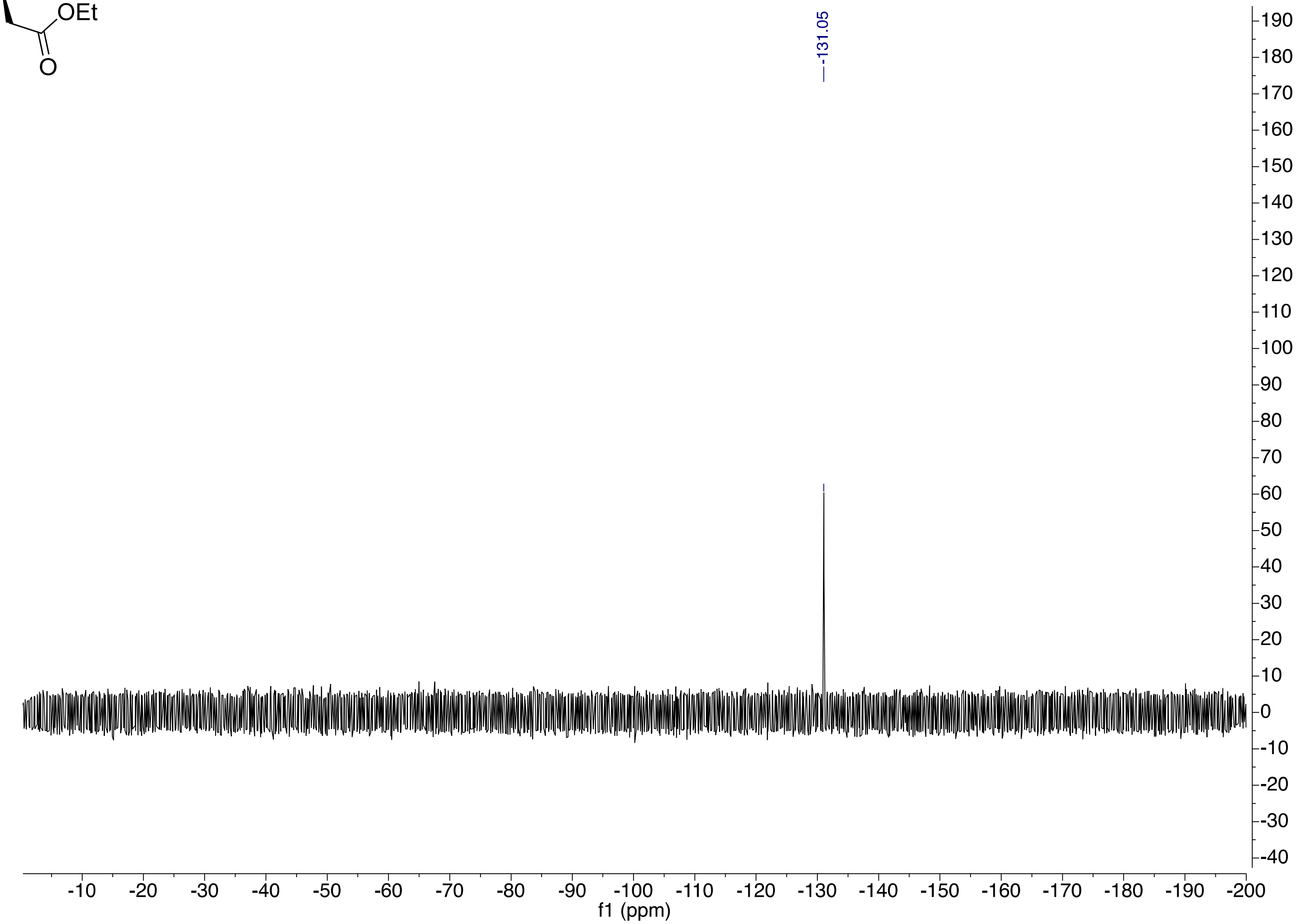

Ethyl 2-(1-(4-chlorophenyl)pyrrolidin-2-yl)acetate (6f/7f)

^{1}H NMR (500 MHz, $CDCl_3$)

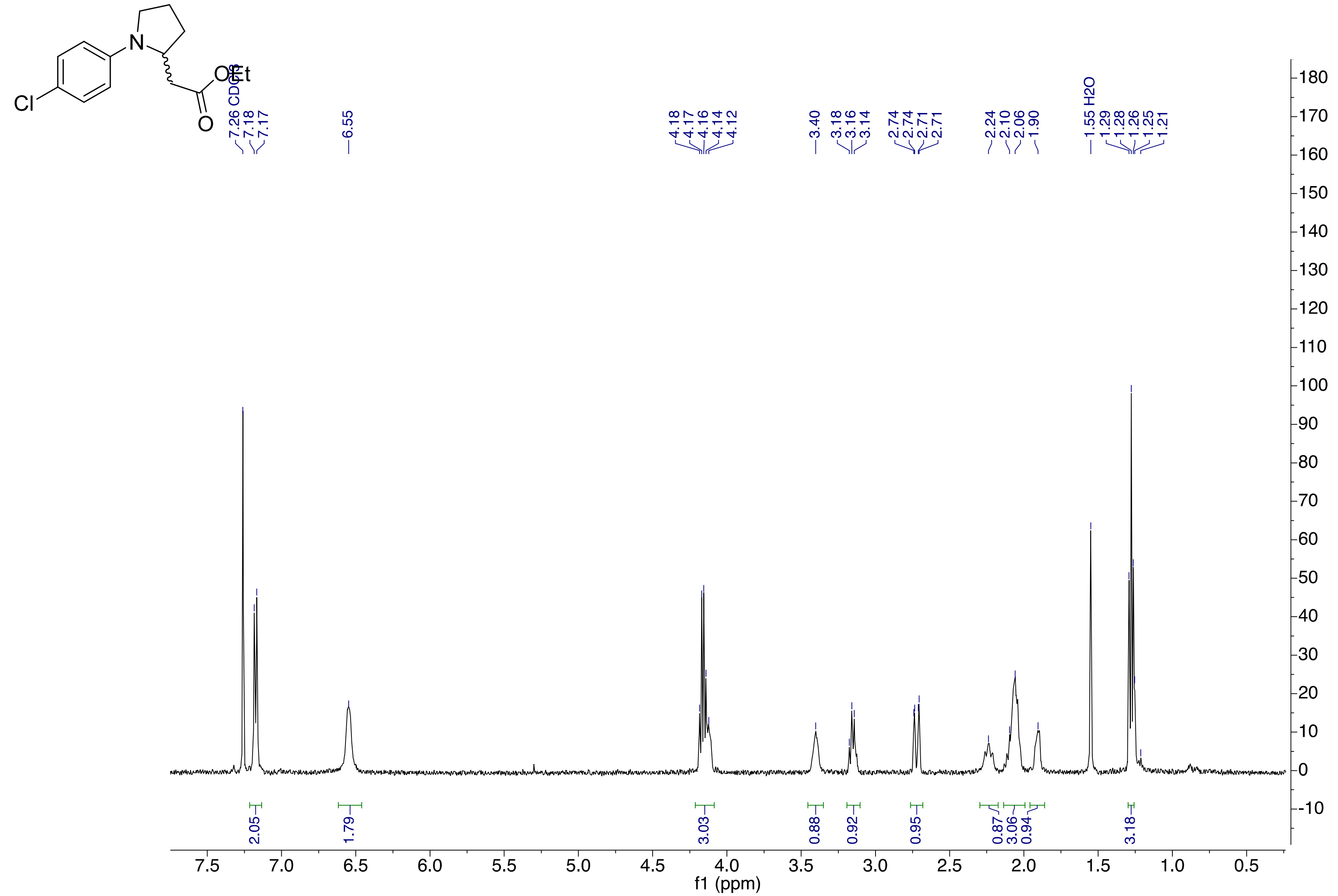

Ethyl 2-(1-(4-chlorophenyl)pyrrolidin-2-yl)acetate (6f/7f)

^{13}C NMR (126 MHz, $CDCl_3$)

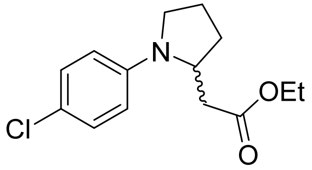

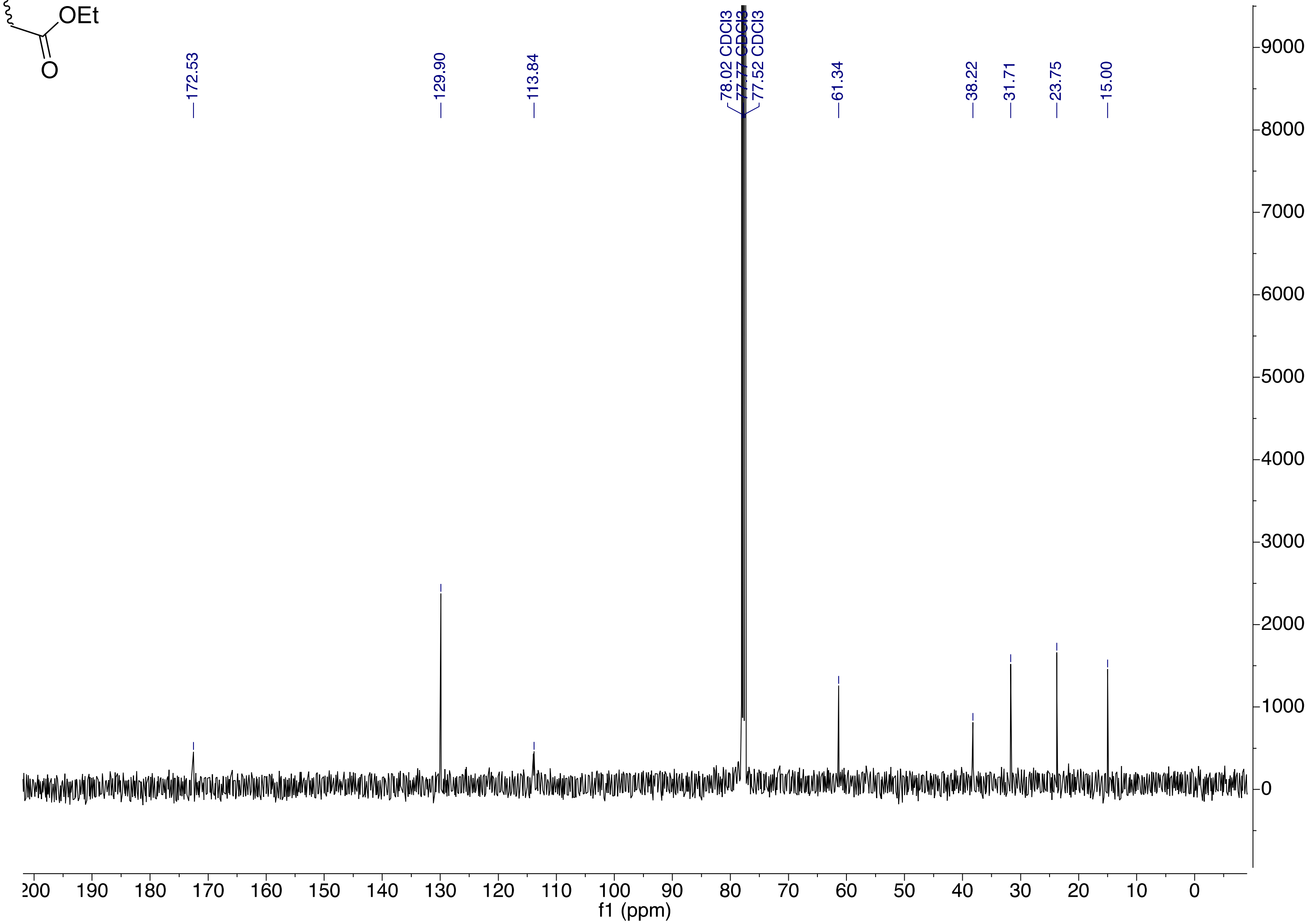

Ethyl (*S*)-2-(1-(4-methoxyphenyl)pyrrolidin-2-yl)acetate (6g)

^{1}H NMR (500 MHz, $CDCl_3$)

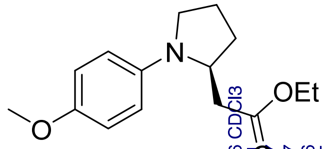

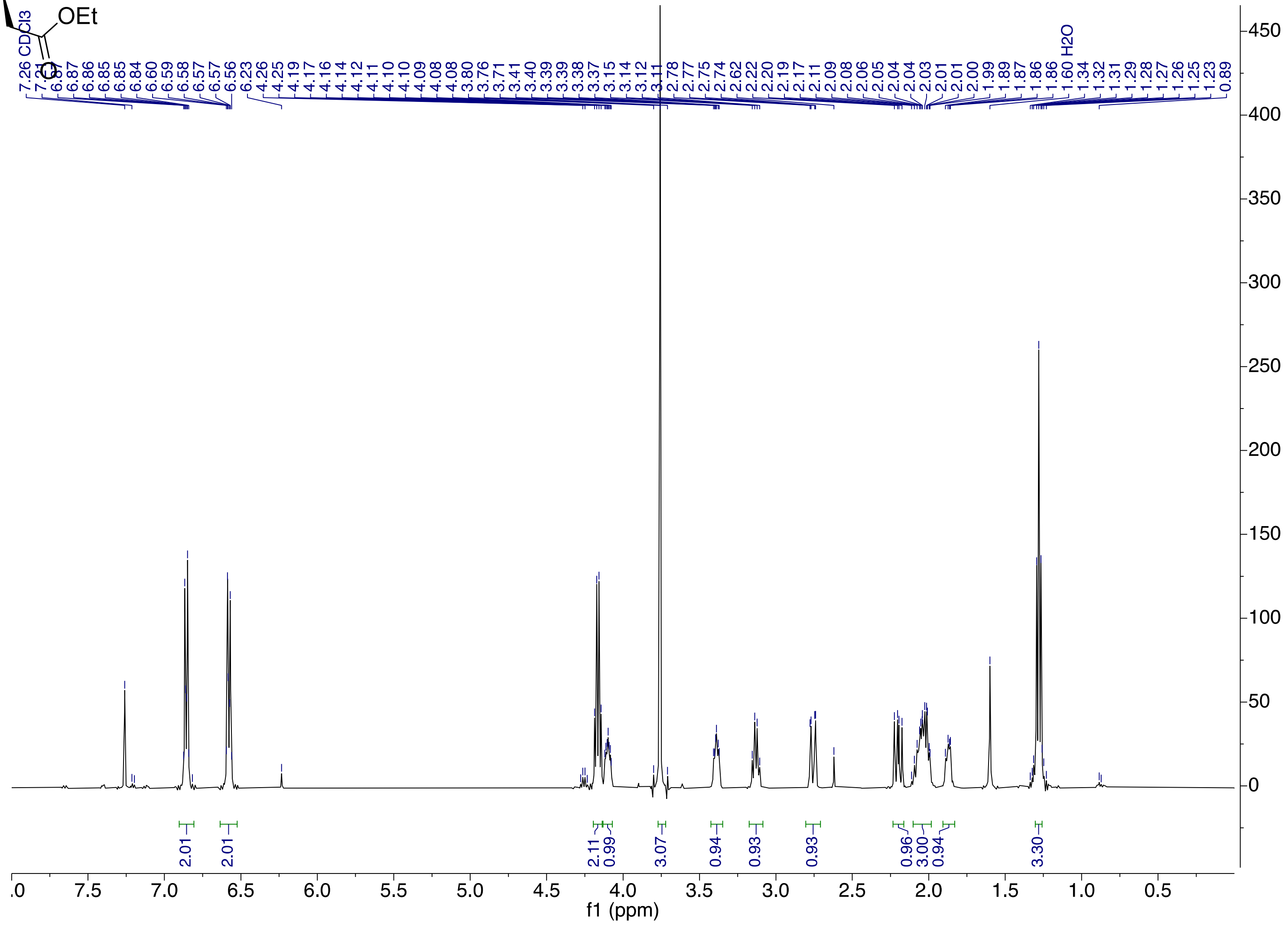

Ethyl (*S*)-2-(1-(4-methoxyphenyl)pyrrolidin-2-yl)acetate (6g)

^{13}C NMR (126 MHz, $CDCl_3$)

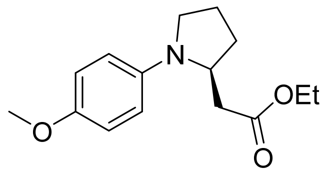

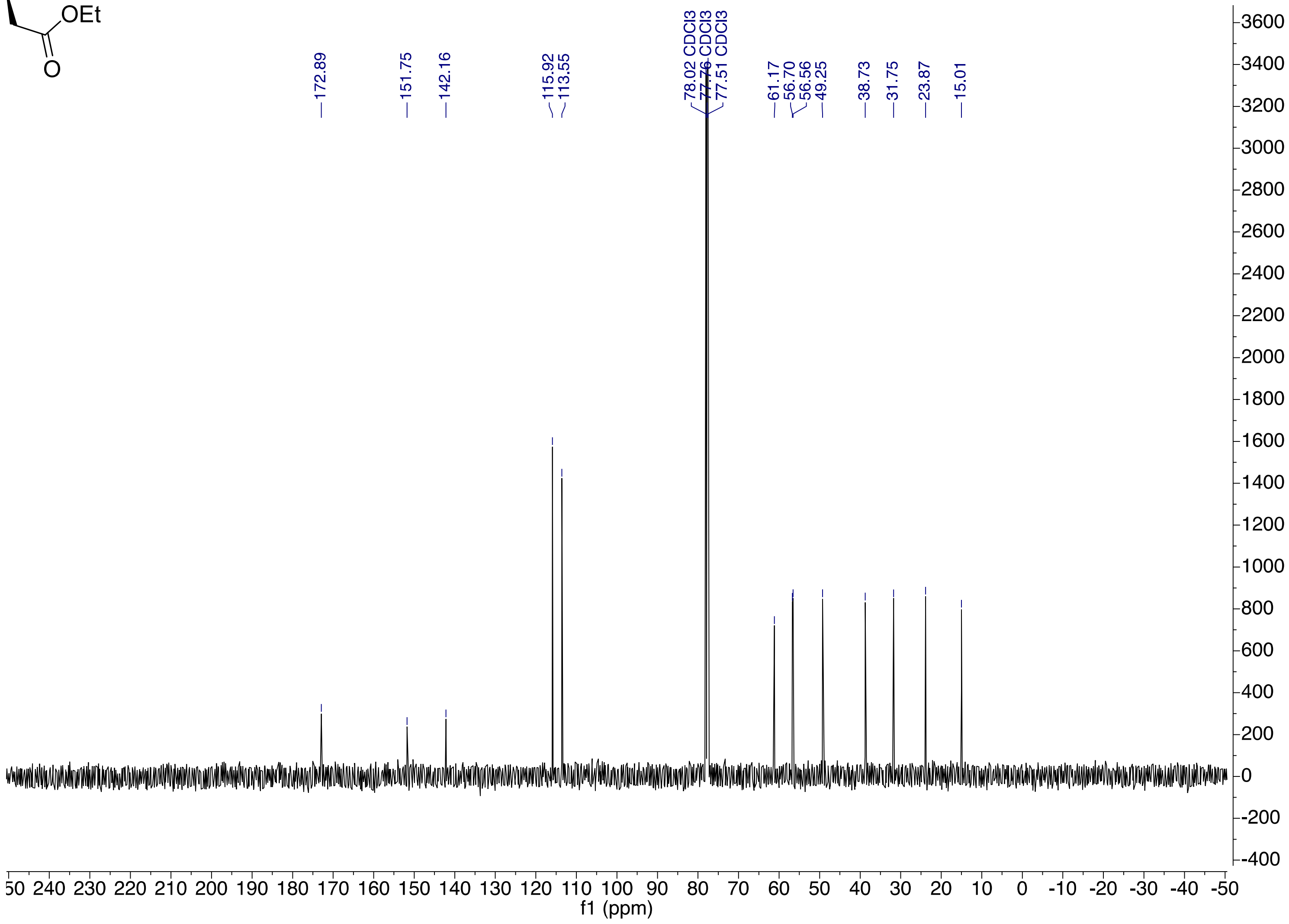

Ethyl (*S*)-2-(1-(3-bromophenyl)pyrrolidin-2-yl)acetate (6h)

^{1}H NMR (500 MHz, $CDCl_3$)

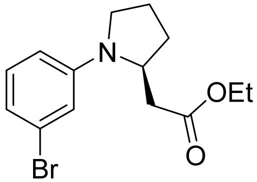

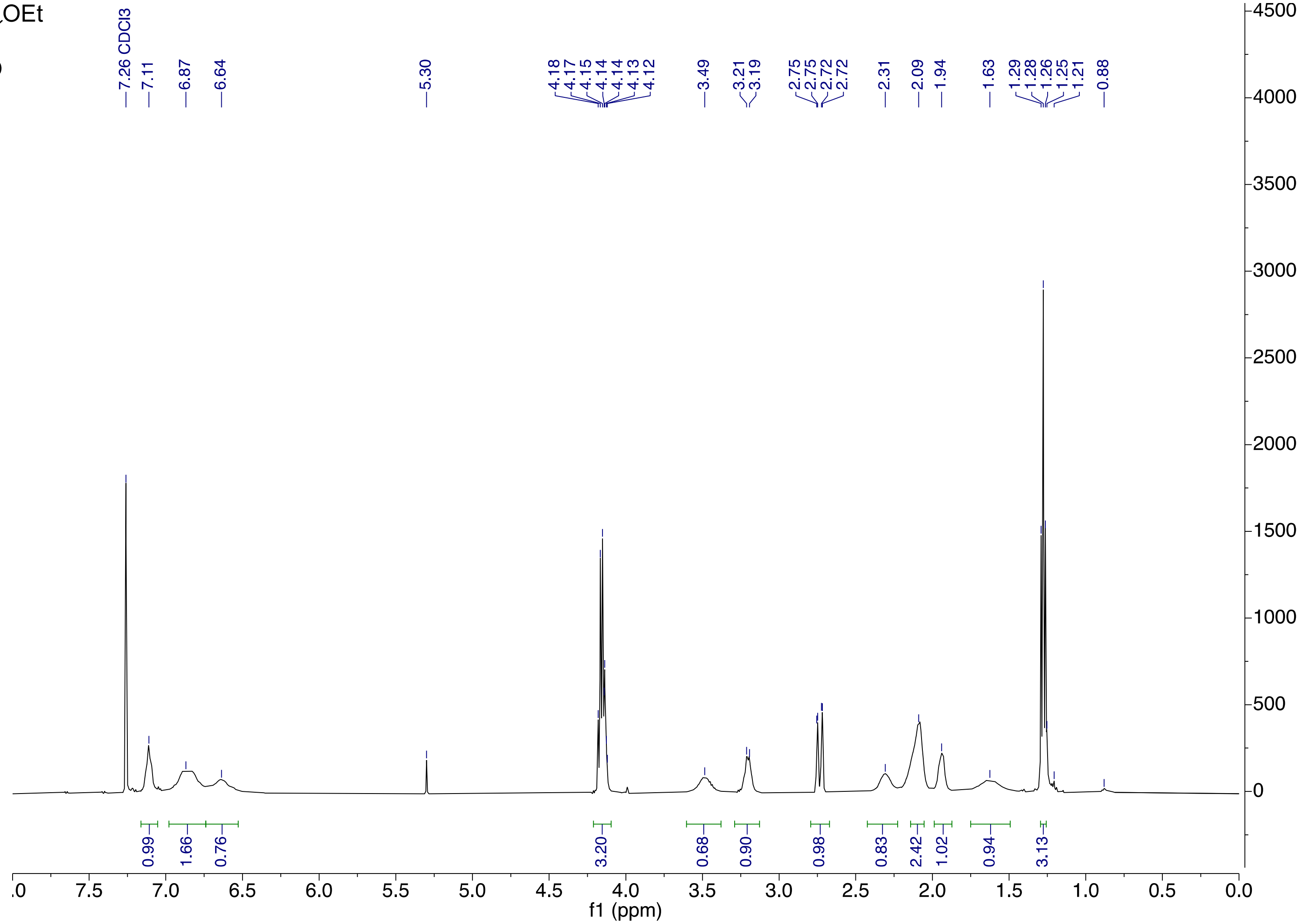

Ethyl (*S*)-2-(1-(3-bromophenyl)pyrrolidin-2-yl)acetate (6h)

^{13}C NMR (126 MHz, $CDCl_3$)

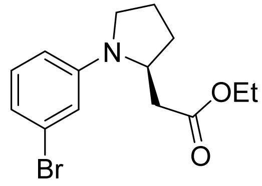

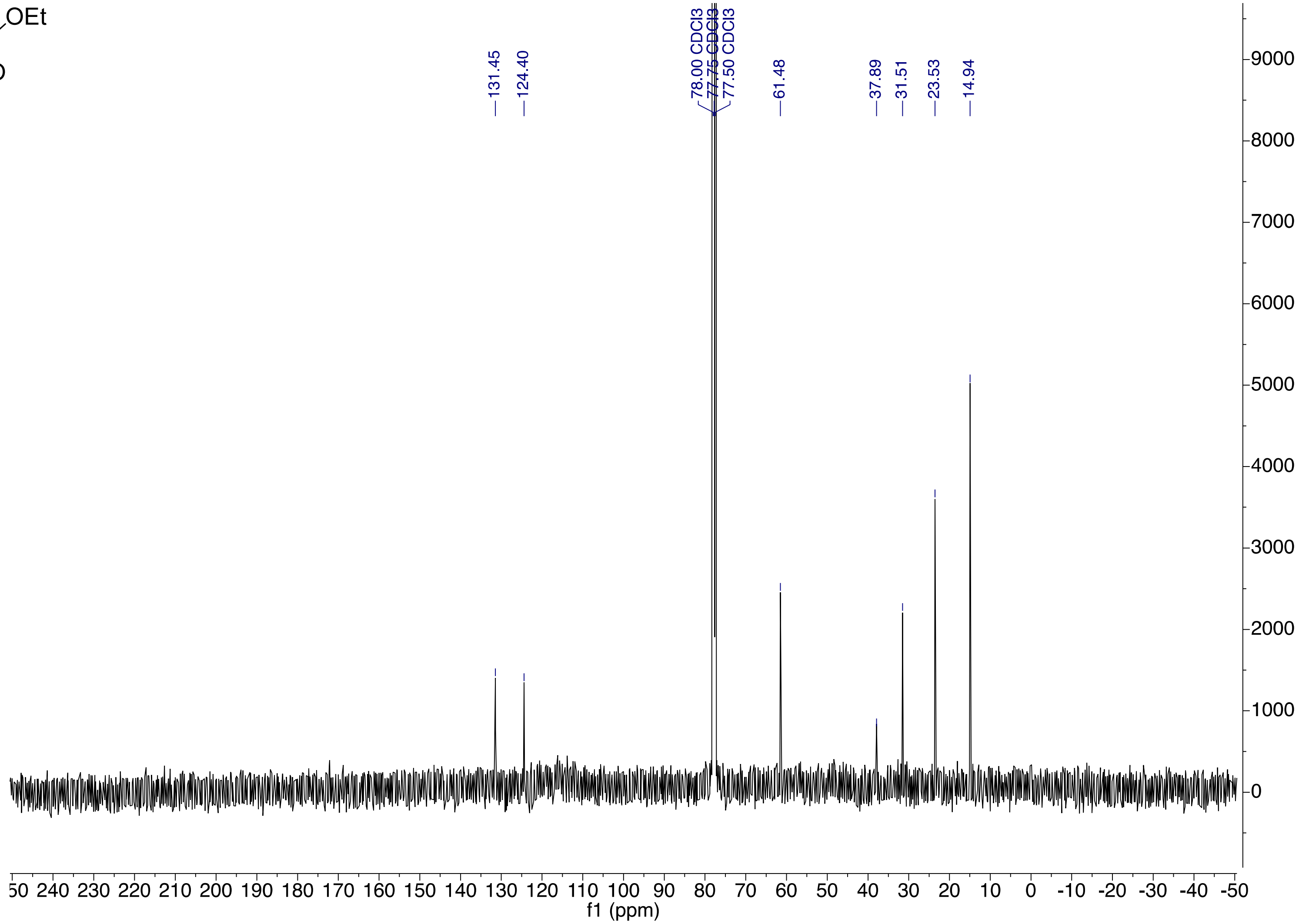

Ethyl (*S*)-2-(1-(2-cyanophenyl)pyrrolidin-2-yl)acetate (6i)

^{1}H NMR (500 MHz, $CDCl_3$)

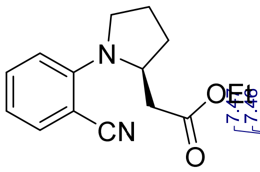

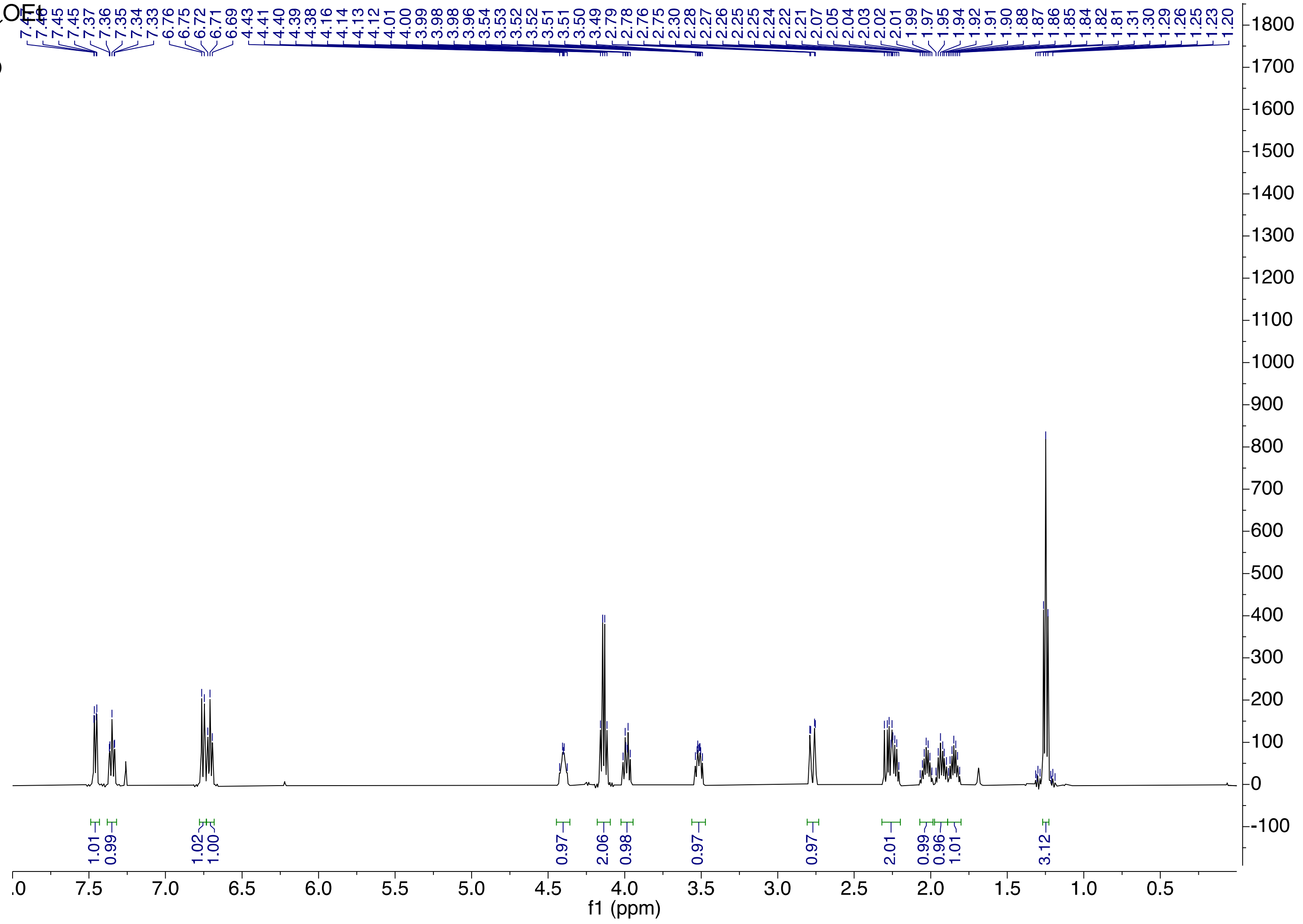

Ethyl (*S*)-2-(1-(2-cyanophenyl)pyrrolidin-2-yl)acetate (6i)

^{13}C NMR (126 MHz, $CDCl_3$)

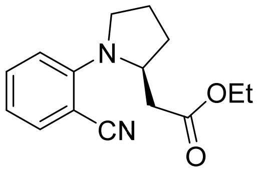

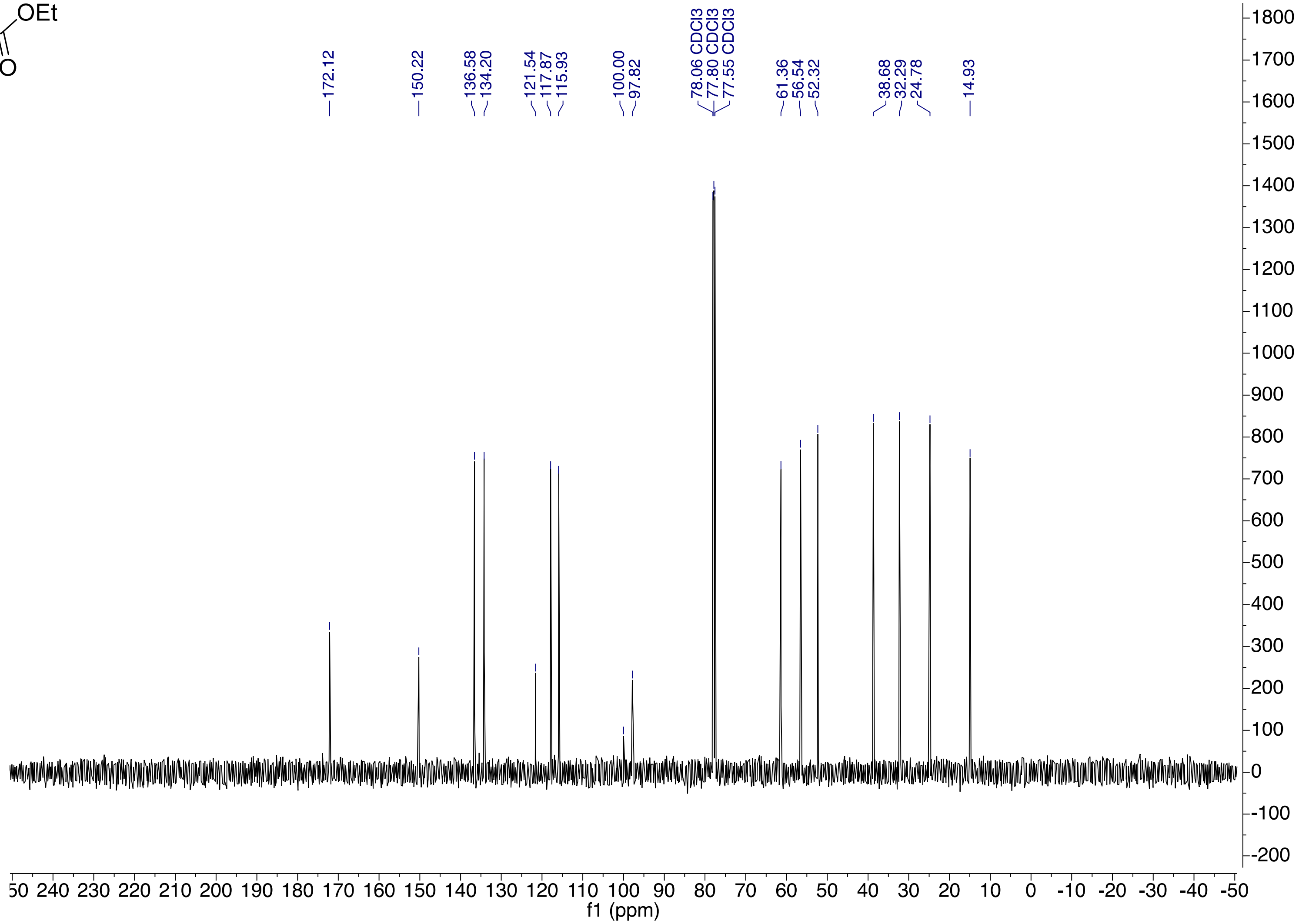

Ethyl 2-((2*S*)-5-methyl-1-phenylpyrrolidin-2-yl)acetate (6k)

^{1}H NMR (500 MHz, $CDCl_3$)

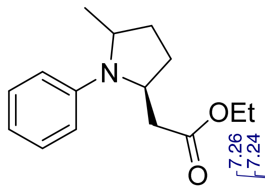

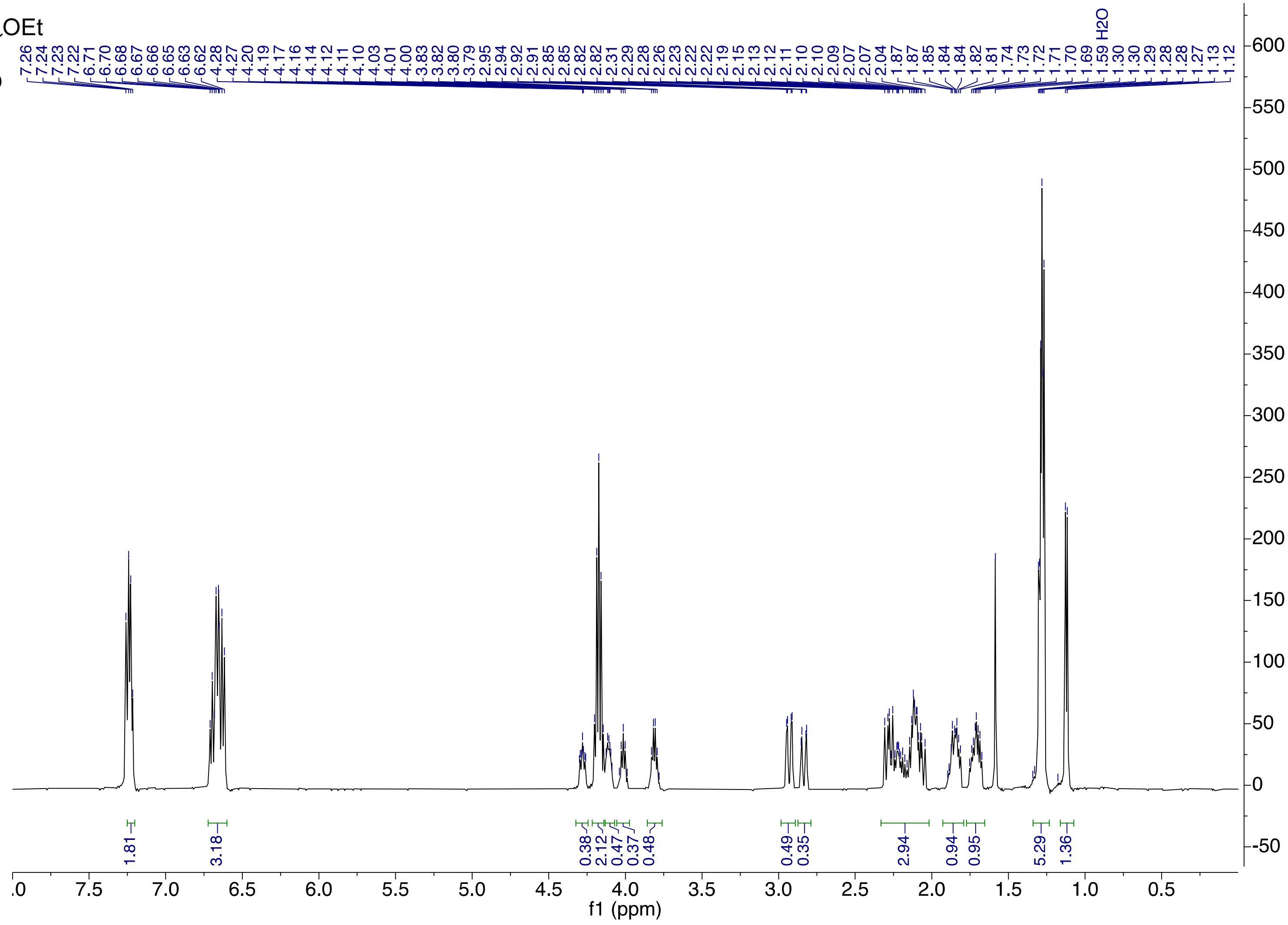

Ethyl 2-((2*S*)-5-methyl-1-phenylpyrrolidin-2-yl)acetate (6k)

^{13}C NMR (126 MHz, $CDCl_3$)

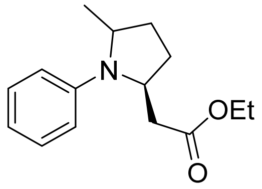

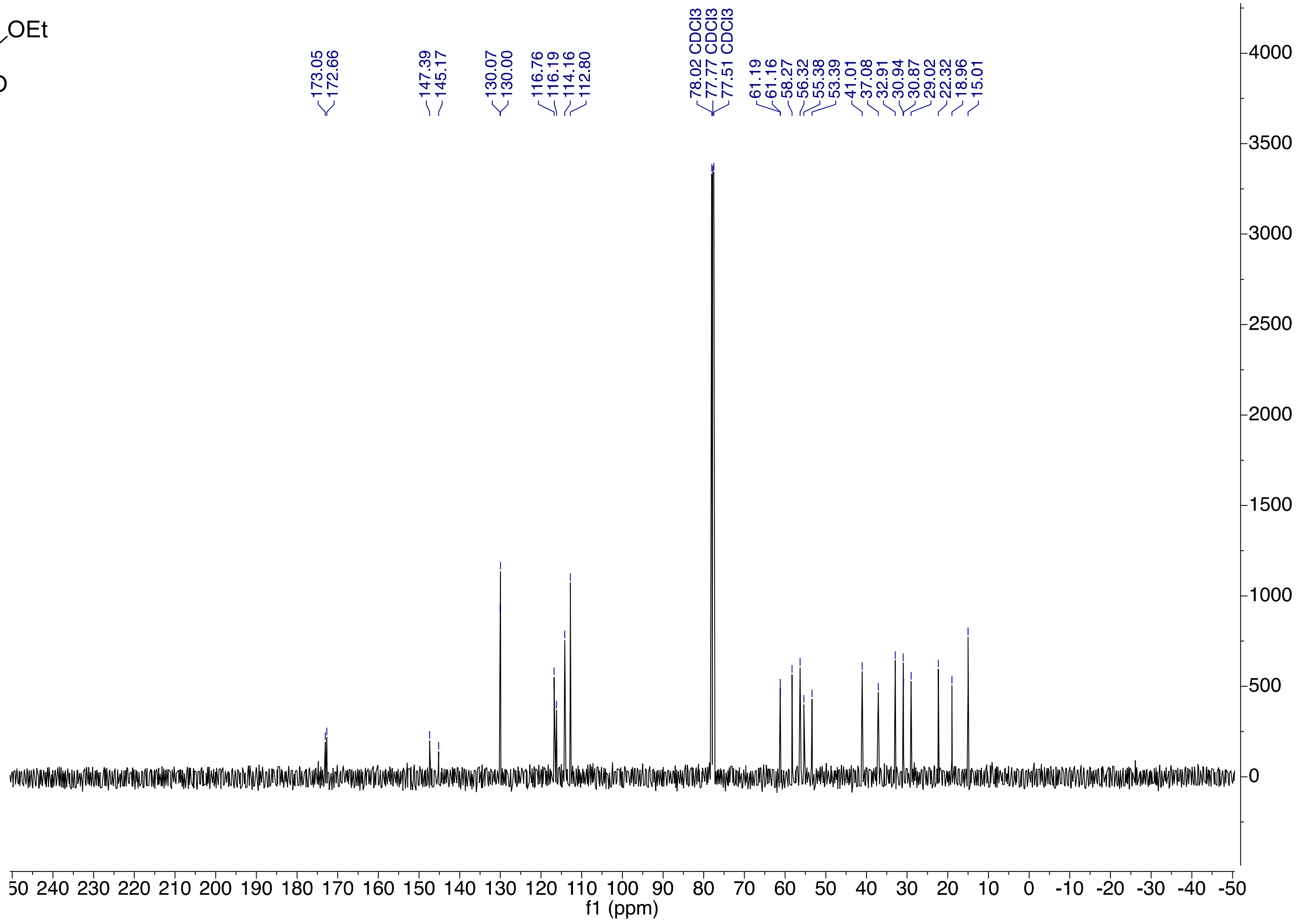

Ethyl 2-(1-phenylpiperidin-2-yl)acetate (6m/7m)

^{1}H NMR (500 MHz, $CDCl_3$)

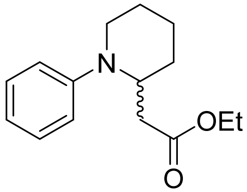

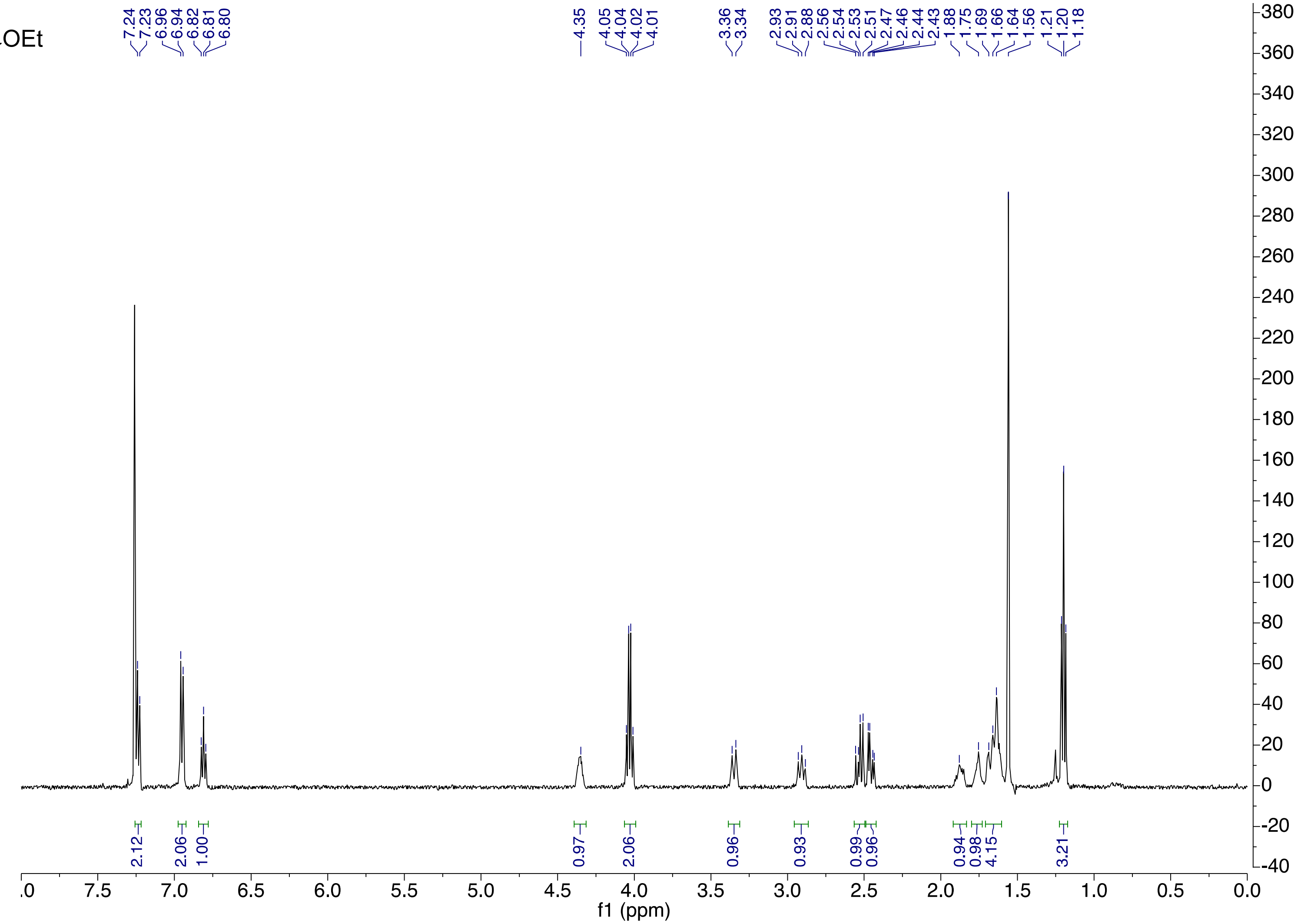

Ethyl 2-(1-phenylpiperidin-2-yl)acetate (6m/7m)

^{13}C NMR (126 MHz, $CDCl_3$)

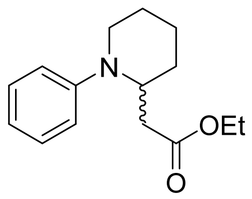

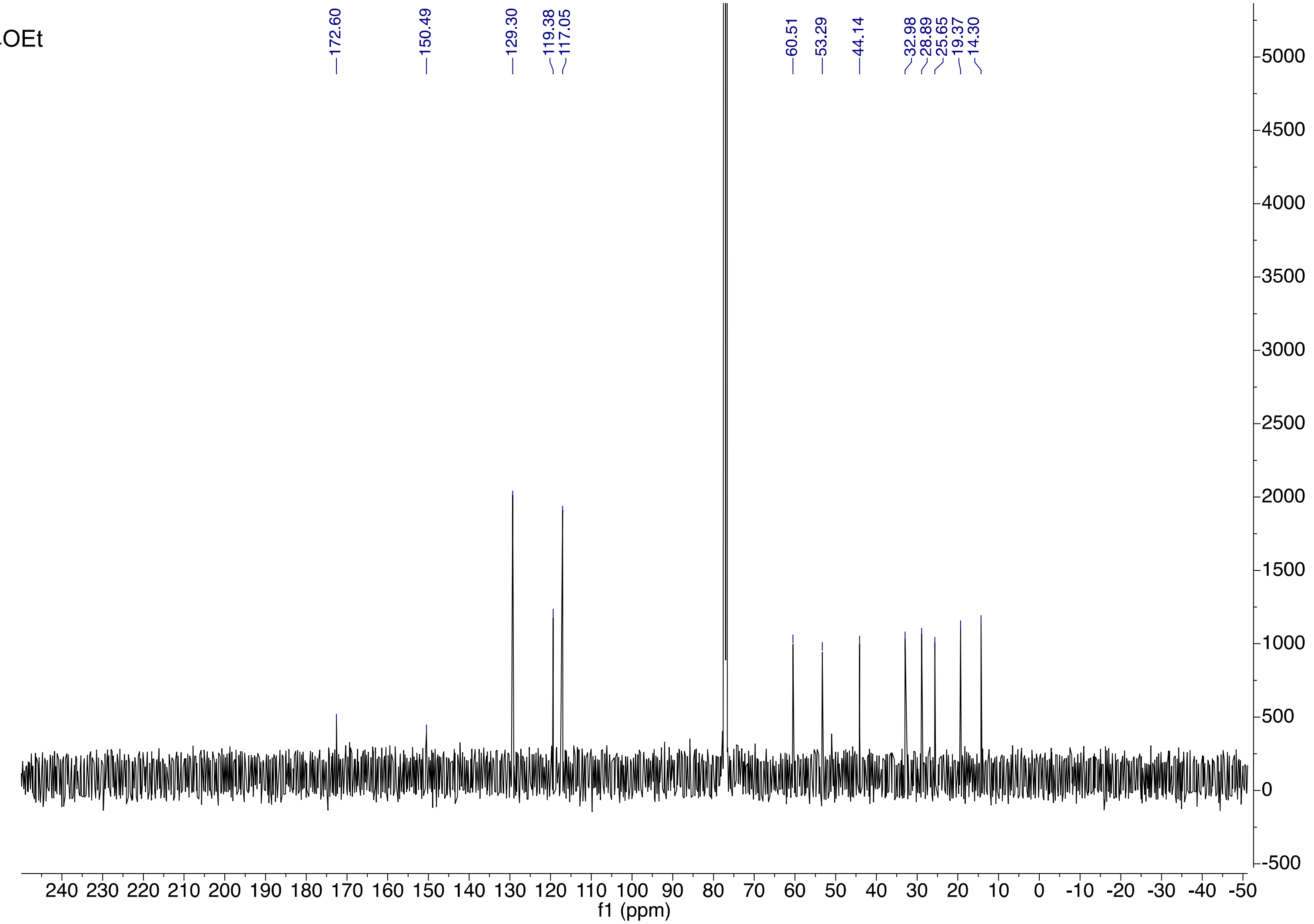

Ethyl (*R*)-2-(4-phenylmorpholin-3-yl)acetate (6n)

^{1}H NMR (500 MHz, $CDCl_3$)

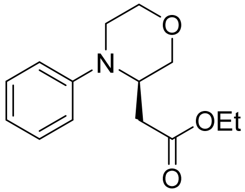

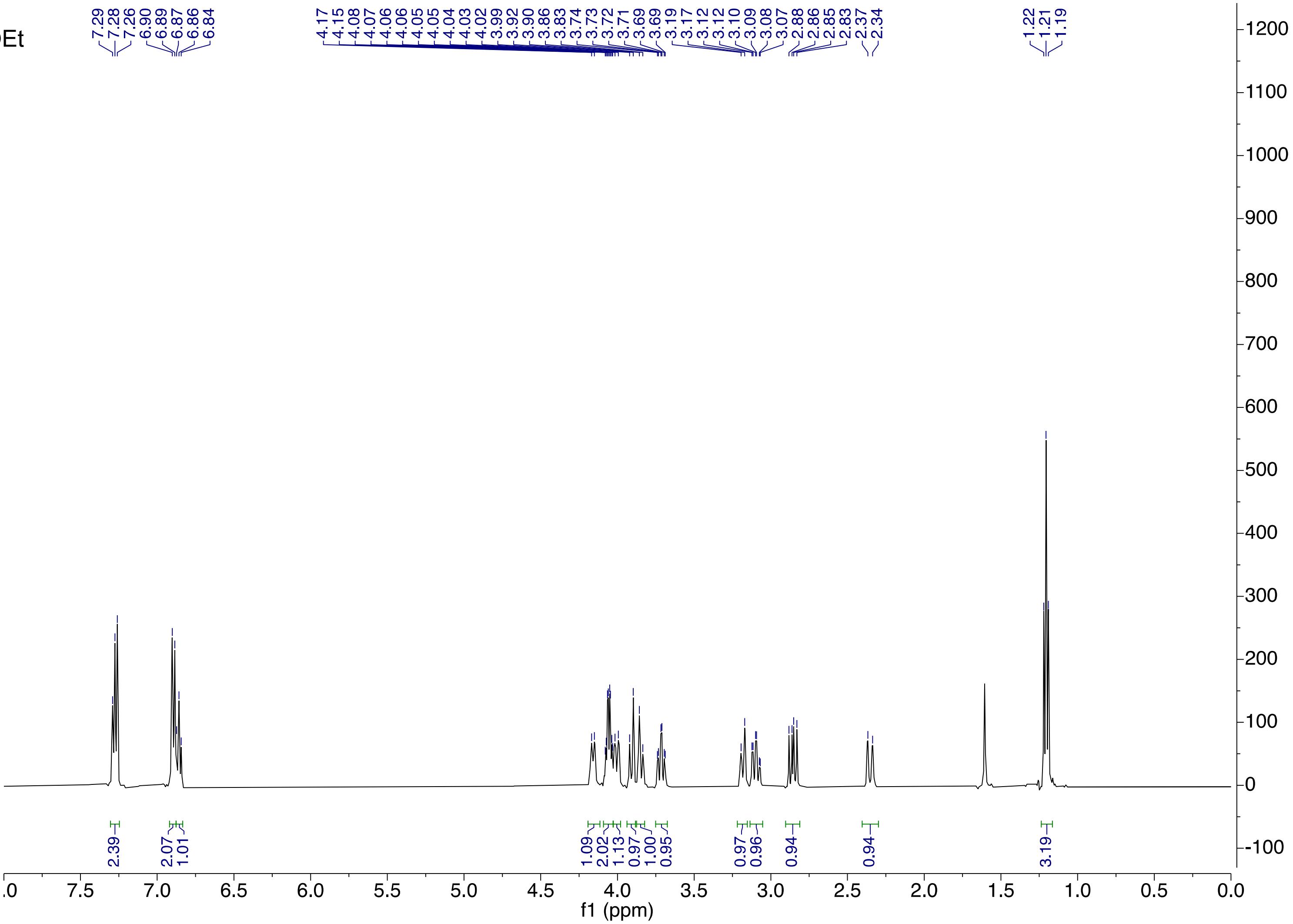

Ethyl (*R*)-2-(4-phenylmorpholin-3-yl)acetate (6n)

^{13}C NMR (126 MHz, $CDCl_3$)

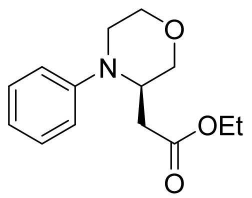

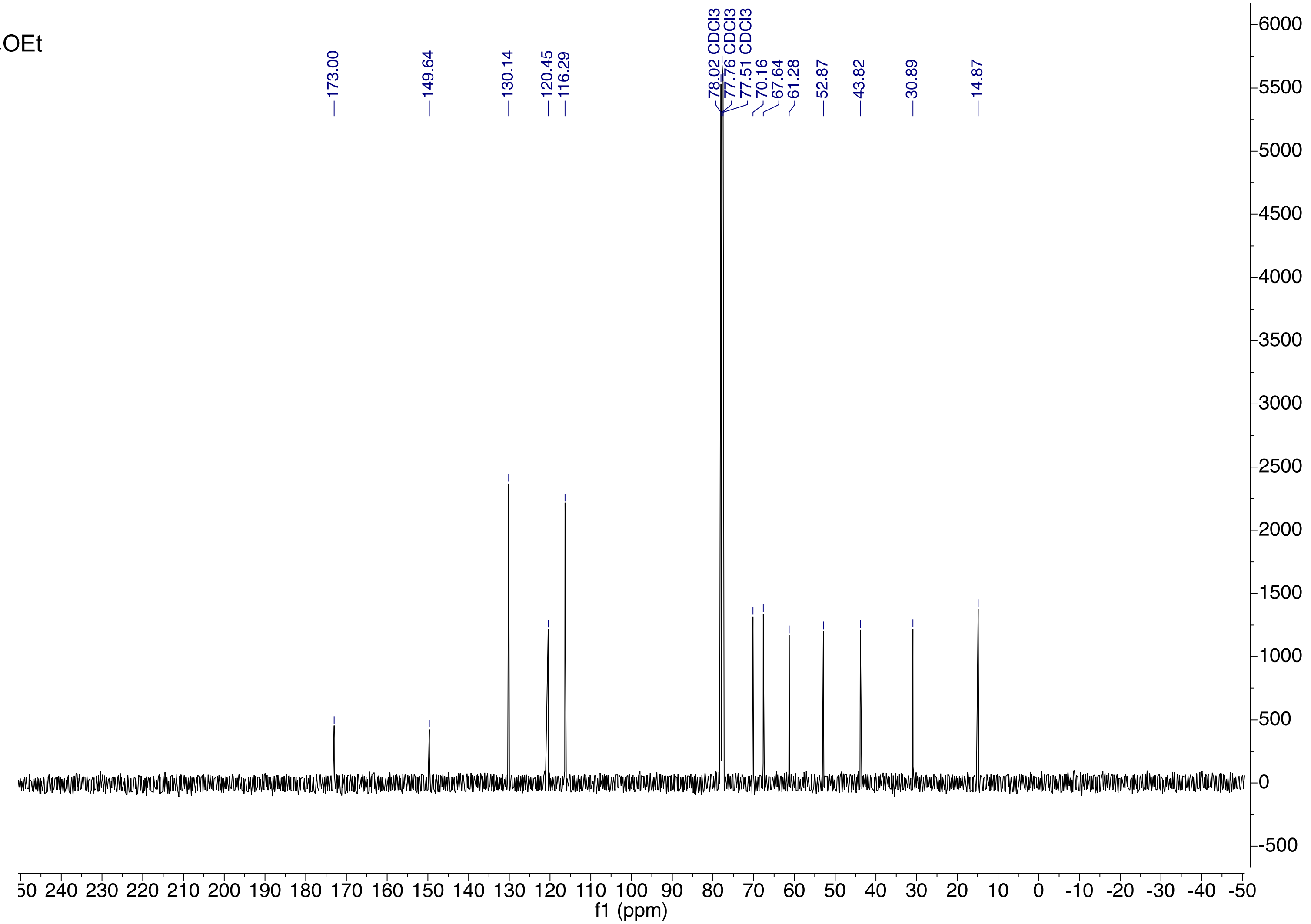

Diethyl 2,2'-(1-phenylpyrrolidine-2,5-diyl)diacetate (8a-c)

^{1}H NMR (500 MHz, $CDCl_3$)

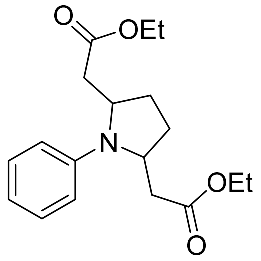

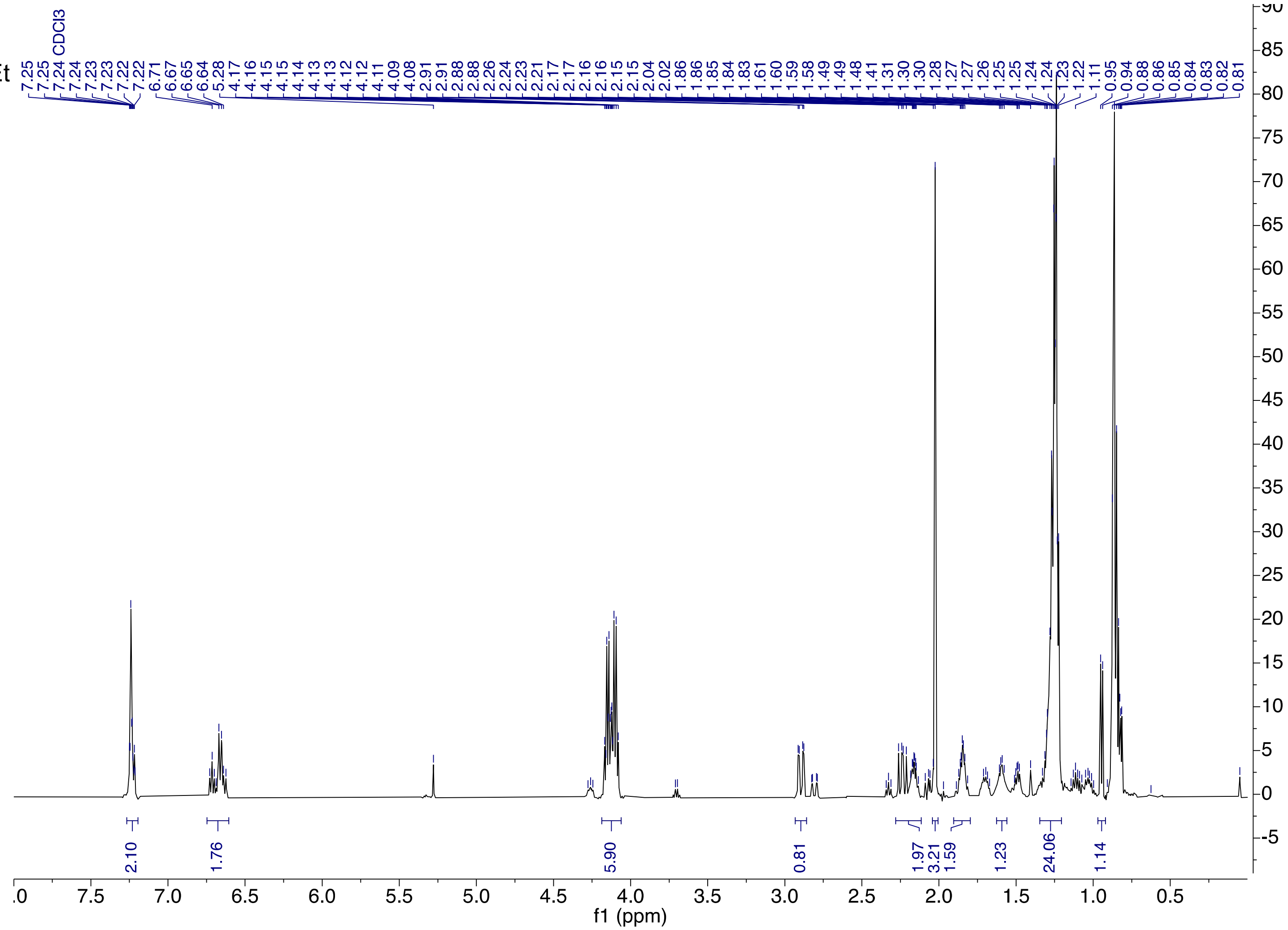

Diethyl 2,2'-(1-phenylpyrrolidine-2,5-diyl)diacetate (8a-c)

^{13}C NMR (126 MHz, $CDCl_3$)

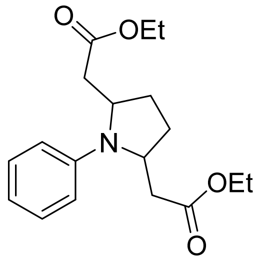

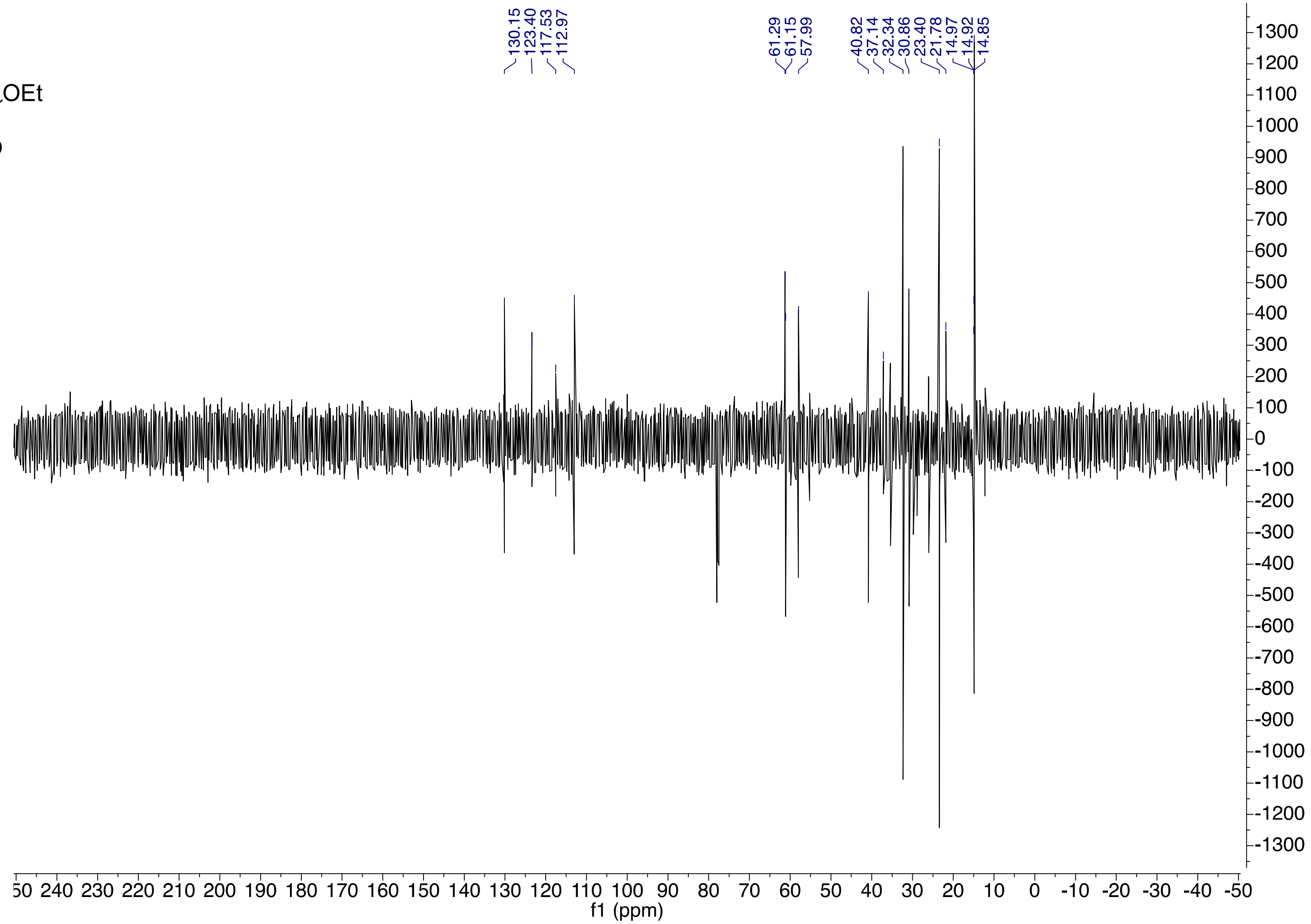

Ethyl 2-(5-(2-oxopropyl)-1-phenylpyrrolidin-2-yl)acetate (9a/10a)

^{1}H NMR (500 MHz, $CDCl_3$)

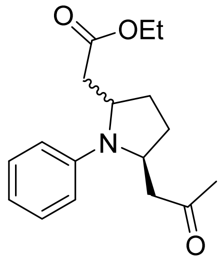

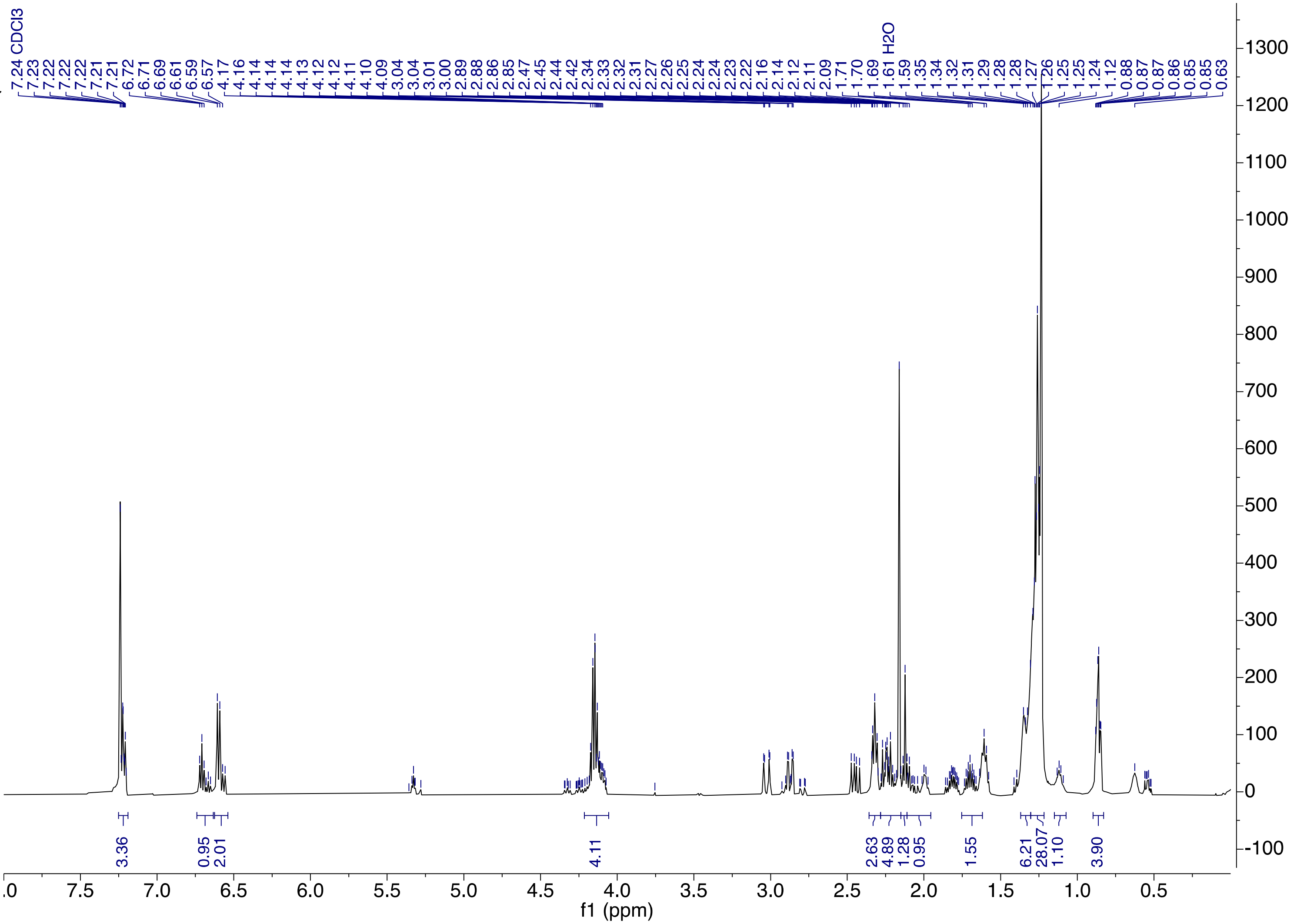

Ethyl 2-(5-(2-oxopropyl)-1-phenylpyrrolidin-2-yl)acetate (9a/10a)

^{13}C NMR (126 MHz, $CDCl_3$)

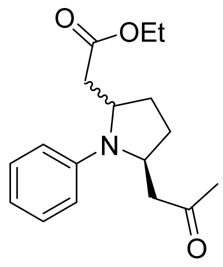

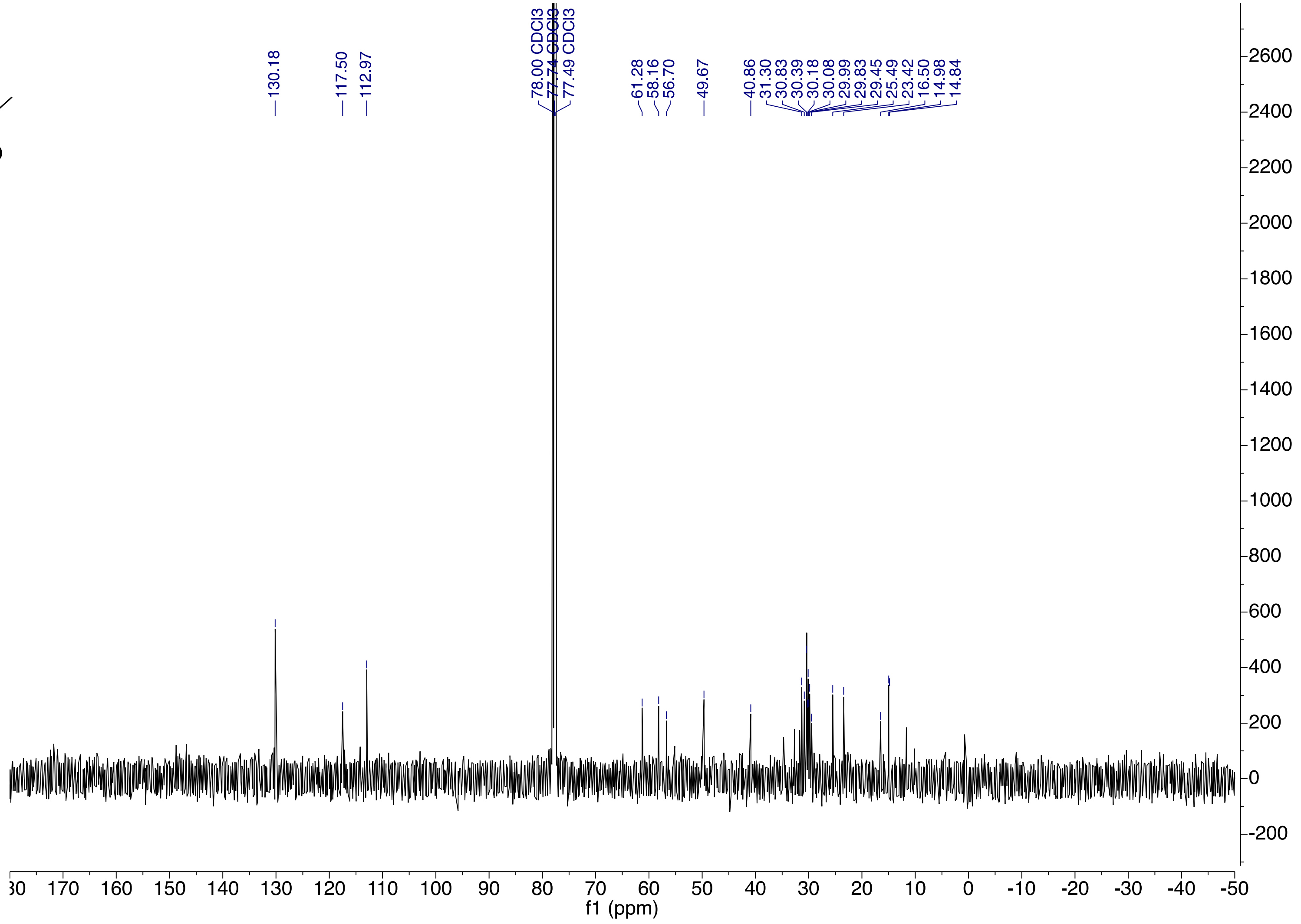

Ethyl 2-(3-(trifluoromethyl)-5,6,7,8-tetrahydro-[1,2,4]triazolo[4,3-a]pyrazin-8-yl)acetate (12)

^{1}H NMR (400 MHz, $CDCl_3$)

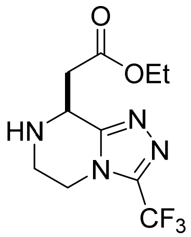

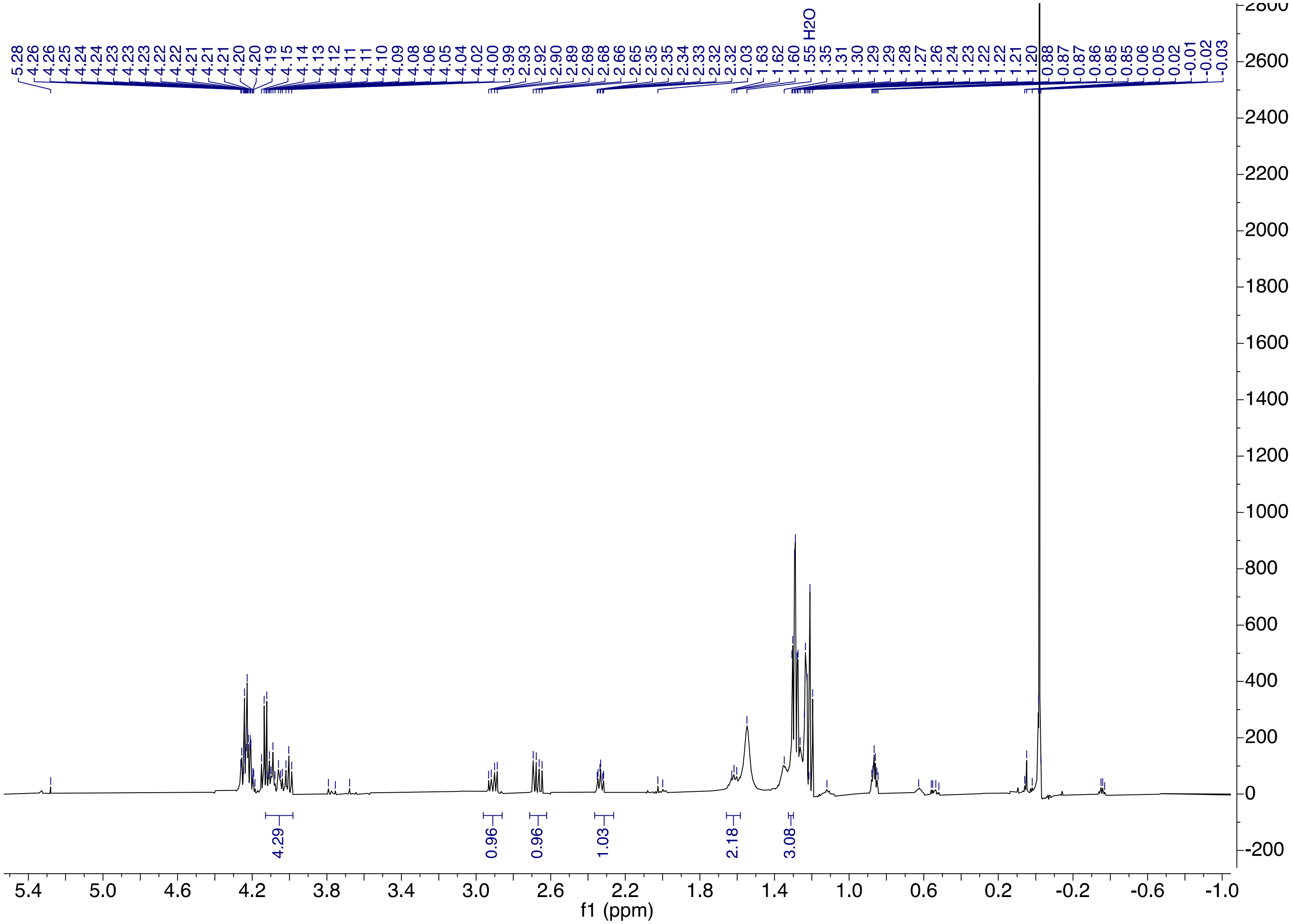

Ethyl 2-(3-(trifluoromethyl)-5,6,7,8-tetrahydro-[1,2,4]triazolo[4,3-a]pyrazin-8-yl)acetate (12)

^{19}F NMR (376 MHz, $CDCl_3$)

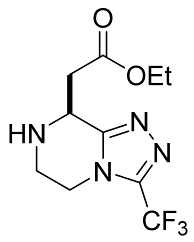

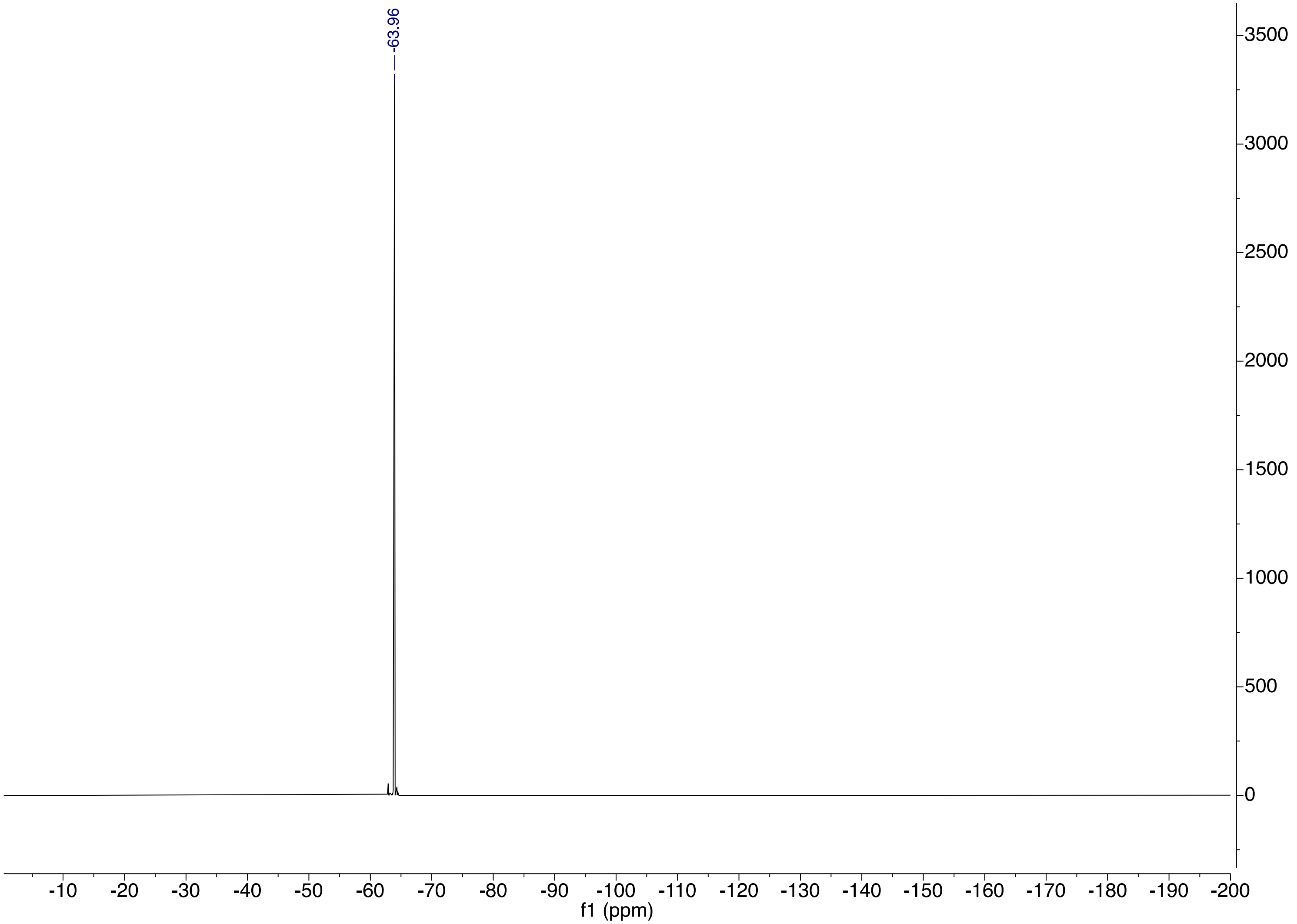

Diethyl 2,2'-(3-(trifluoromethyl)-5,6-dihydro-[1,2,4]triazolo[4,3-a]pyrazine-7,8(8H)-diyl)diacetate (13)

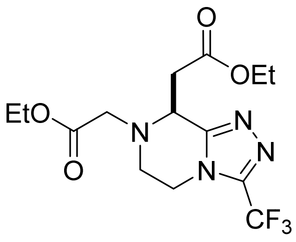

^1H NMR (500 MHz, $CDCl_3$)

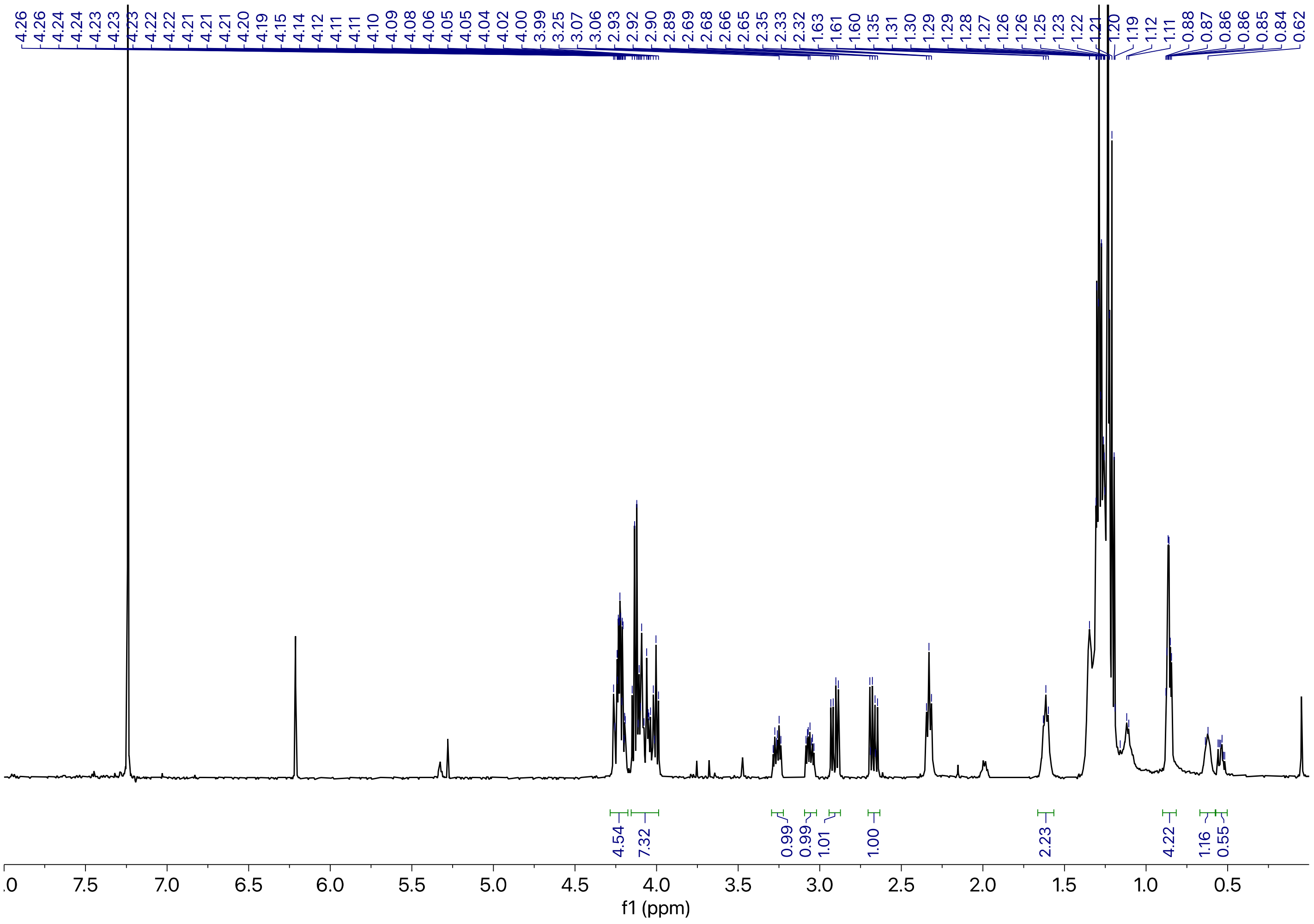

Diethyl 2,2'-(3-(trifluoromethyl)-5,6-dihydro-[1,2,4]triazolo[4,3-a]pyrazine-7,8(8H)-diyl)diacetate (13)

^{13}C NMR (126 MHz, $CDCl_3$)

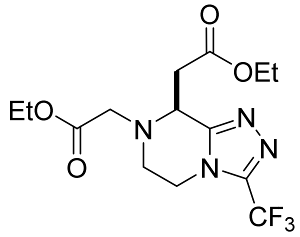

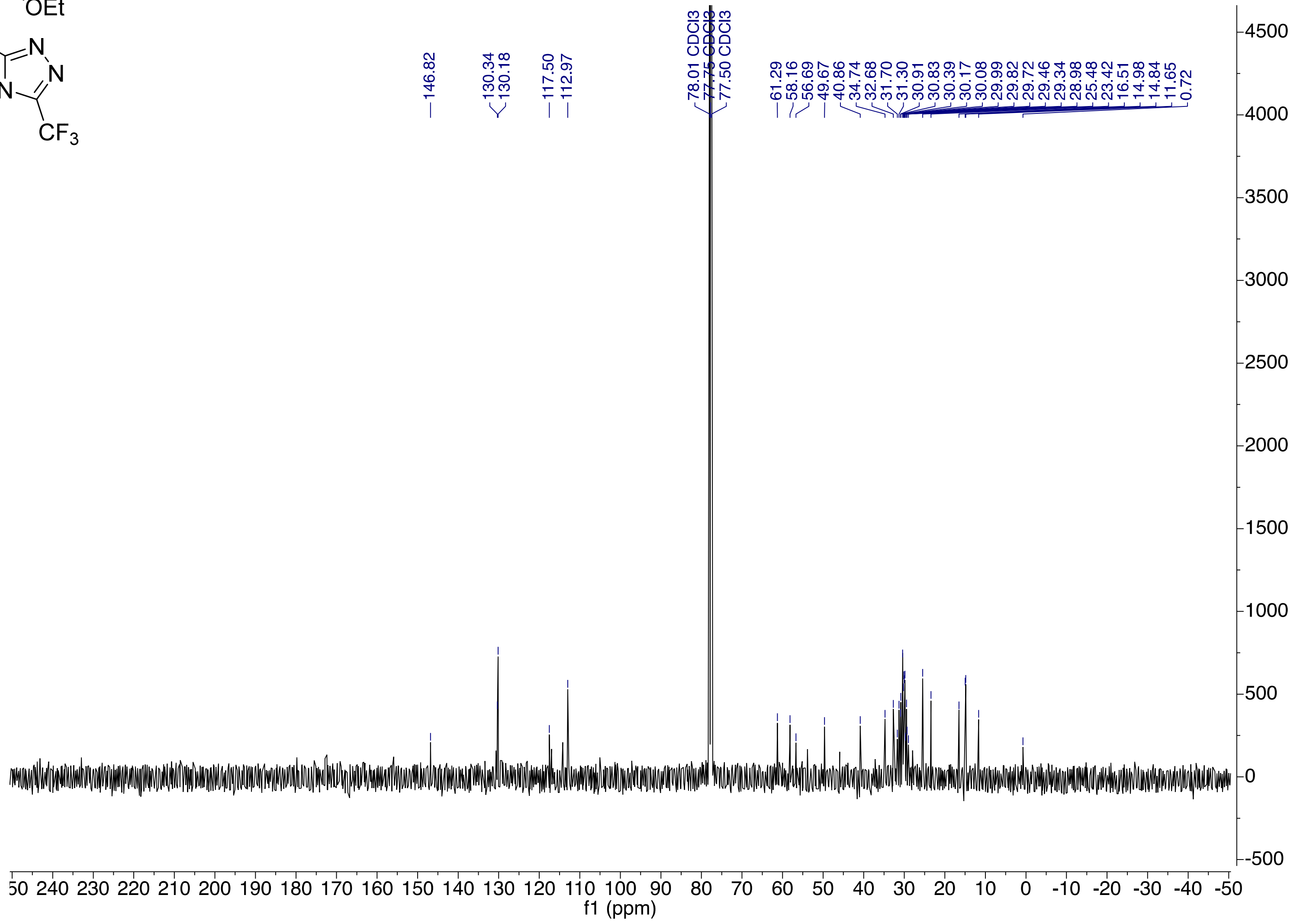

Diethyl 2,2'-(3-(trifluoromethyl)-5,6-dihydro-[1,2,4]triazolo[4,3-a]pyrazine-7,8(8H)-diyl)diacetate (13)

^{19}F NMR (376 MHz, $CDCl_3$)

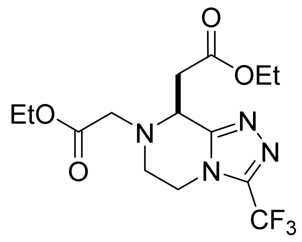

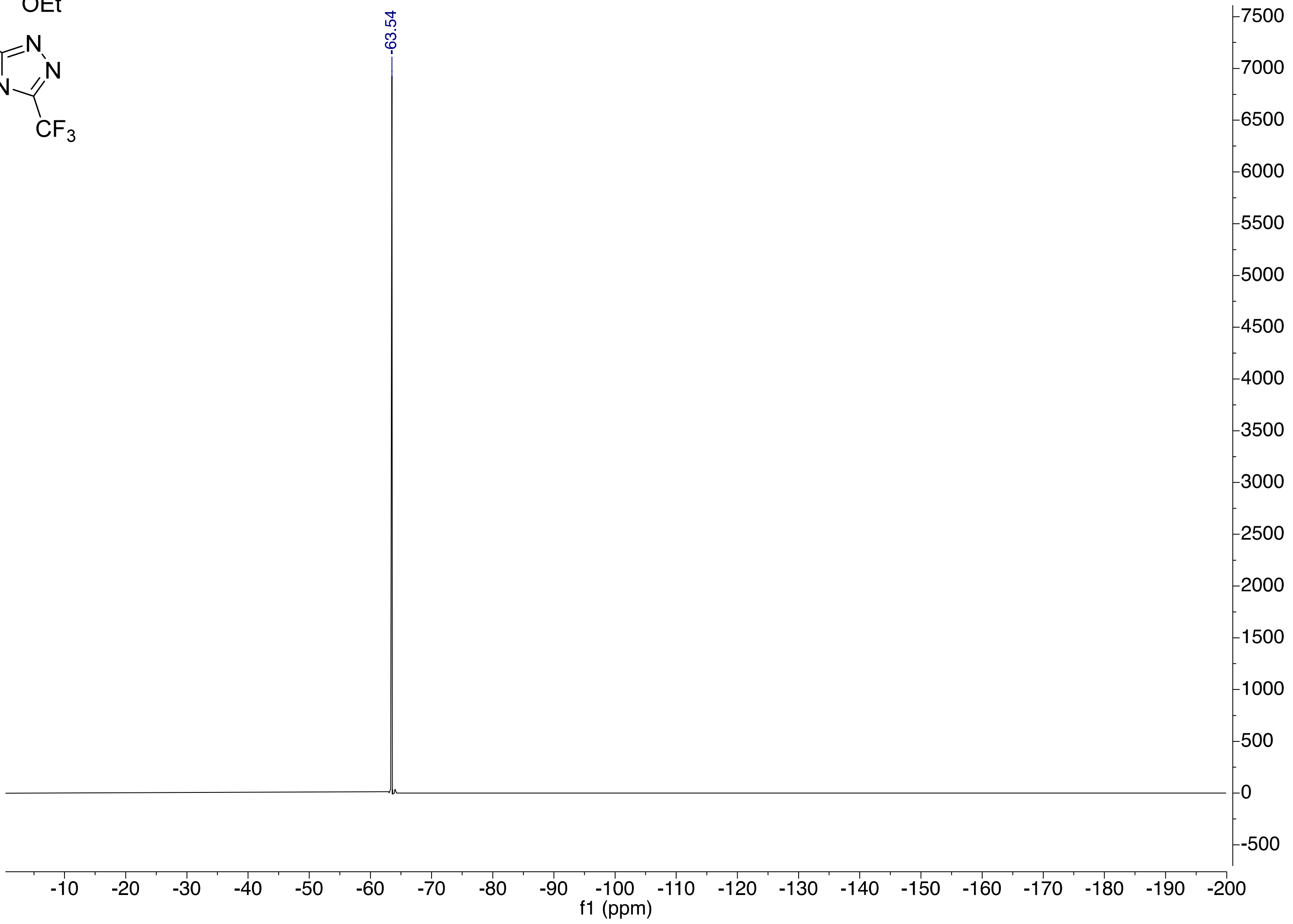

Ethyl 2-(3-(trifluoromethyl)-5,6-dihydro-[1,2,4]triazolo[4,3-a]pyrazin-7(8H)-yl)acetate (14)

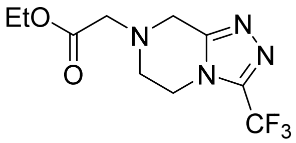

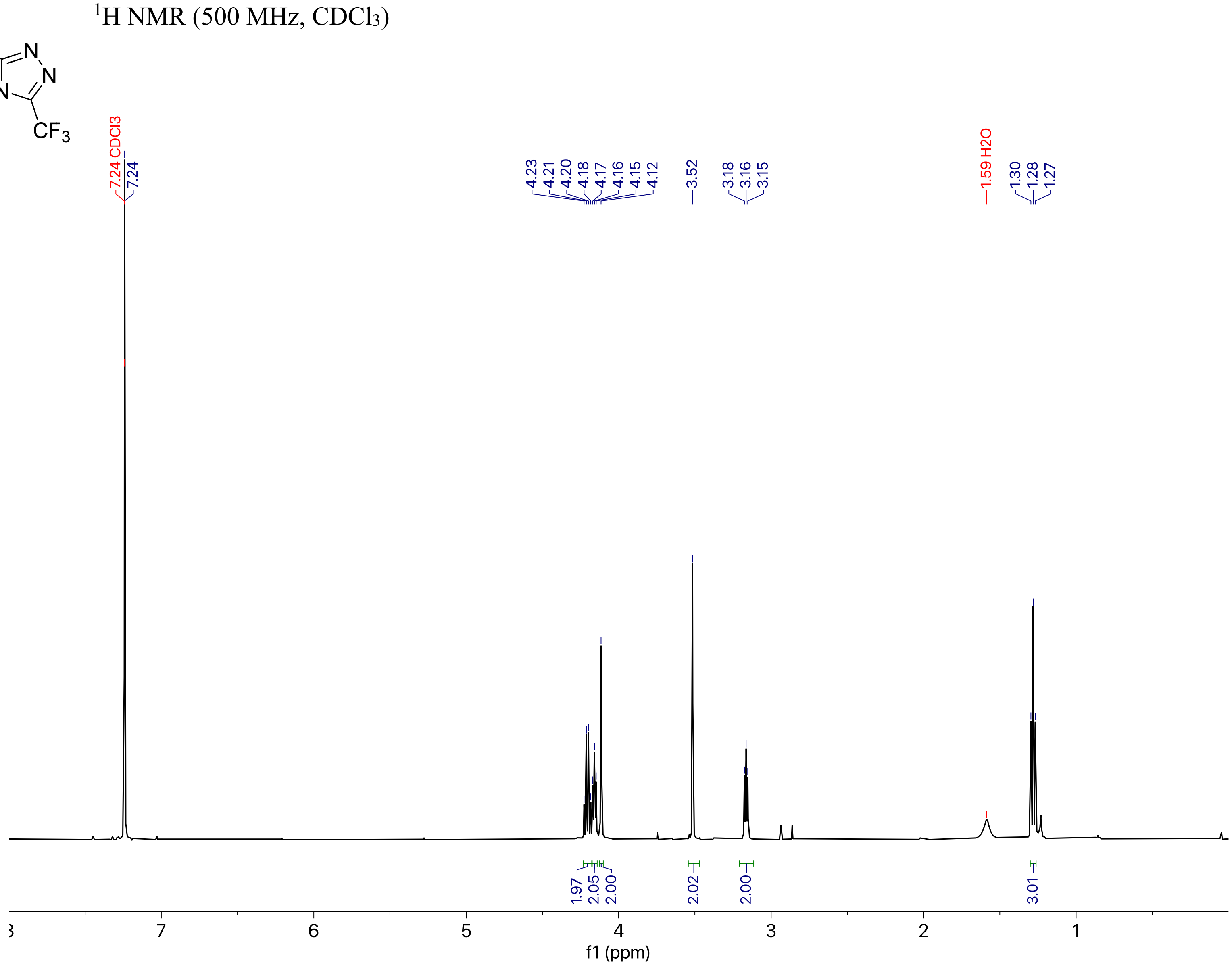

Ethyl 2-(3-(trifluoromethyl)-5,6-dihydro-[1,2,4]triazolo[4,3-a]pyrazin-7(8H)-yl)acetate (14)

^{13}C NMR (101 MHz, $CDCl_3$)

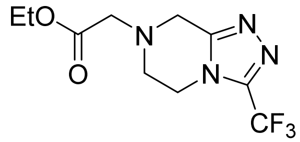

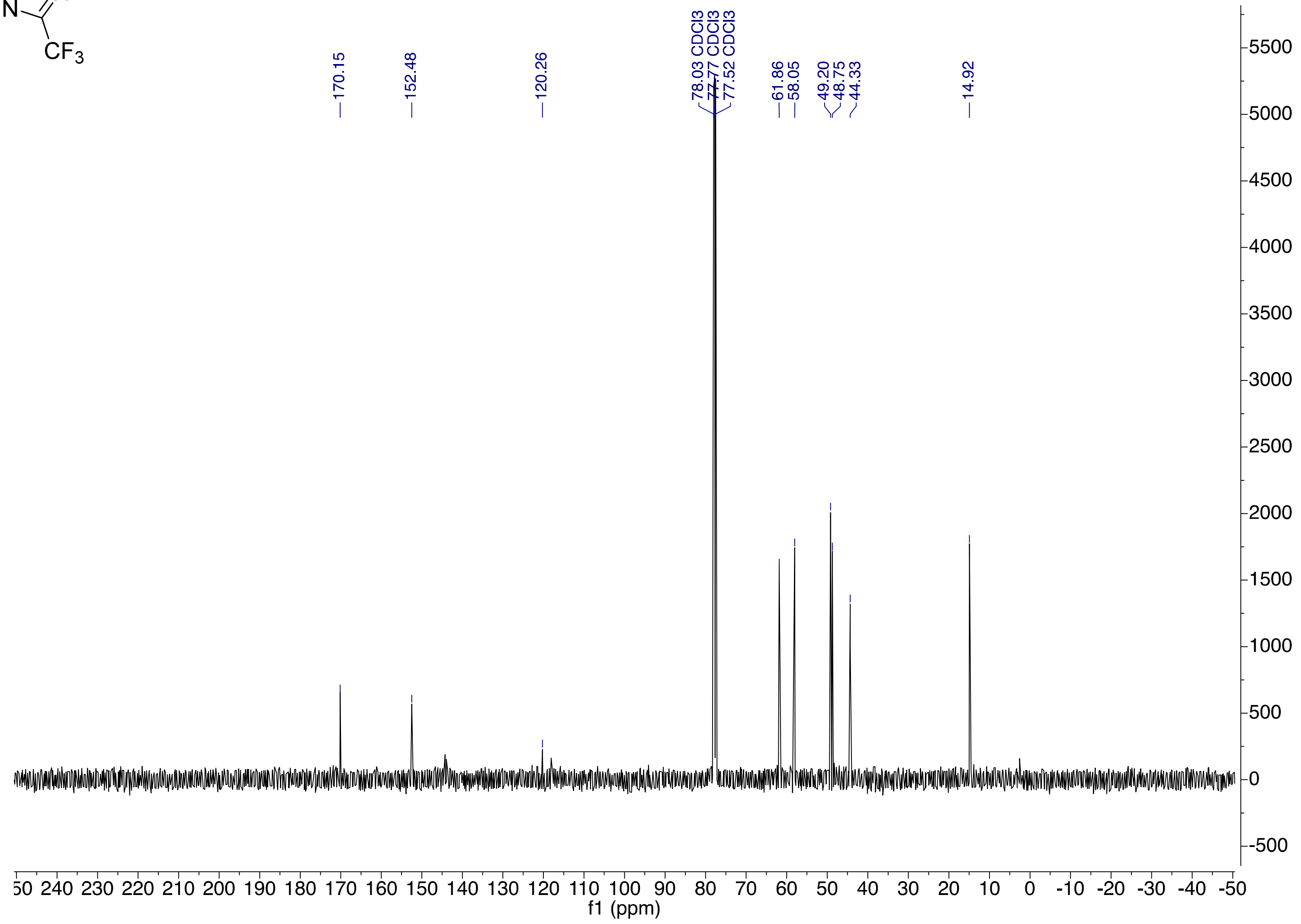

Ethyl 2-(3-(trifluoromethyl)-5,6-dihydro-[1,2,4]triazolo[4,3-a]pyrazin-7(8H)-yl)acetate (14)

^{19}F NMR (376 MHz, $CDCl_3$)

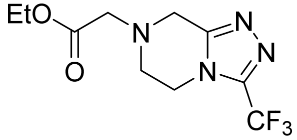

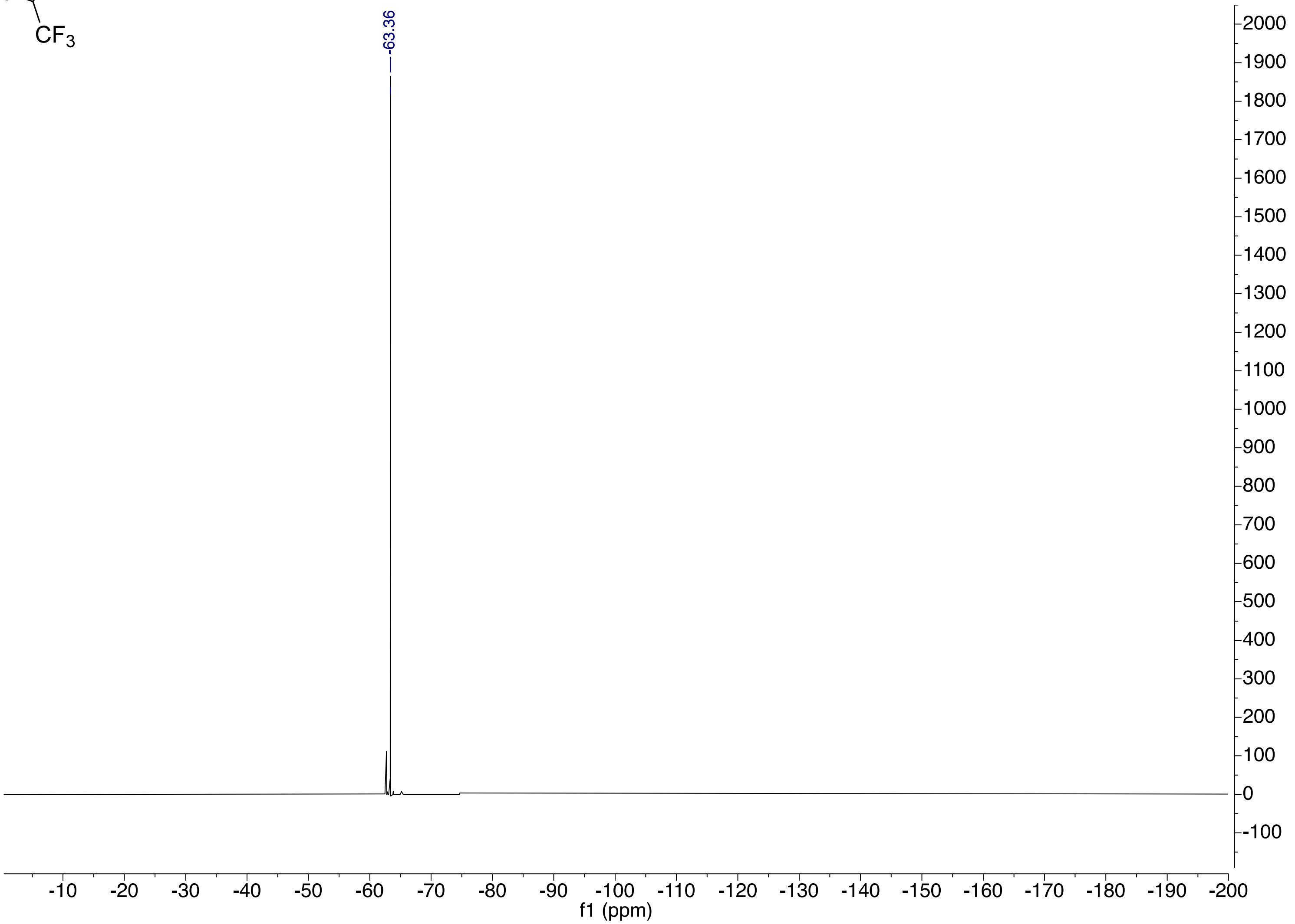

Supplement: Supplementary file 1 — ja2c10775_si_001.pdf [file ja2c10775_si_001.pdf]
